# Identification, Structure–Activity Relationship, and Biological Characterization of 2,3,4,5-Tetrahydro-1*H*-pyrido[4,3-*b*]indoles as a Novel Class of CFTR Potentiators

**DOI:** 10.1021/acs.jmedchem.0c01050

**Published:** 2020-09-18

**Authors:** Nicoletta Brindani, Ambra Gianotti, Simone Giovani, Francesca Giacomina, Paolo Di Fruscia, Federico Sorana, Sine Mandrup Bertozzi, Giuliana Ottonello, Luca Goldoni, Ilaria Penna, Debora Russo, Maria Summa, Rosalia Bertorelli, Loretta Ferrera, Emanuela Pesce, Elvira Sondo, Luis J. V. Galietta, Tiziano Bandiera, Nicoletta Pedemonte, Fabio Bertozzi

**Affiliations:** † D3-PharmaChemistry, Istituto Italiano di Tecnologia (IIT), 16163 Genova, Italy; ‡ UOC Genetica Medica, IRCCS Istituto Giannina Gaslini, 16147 Genova, Italy; § Analytical Chemistry and Translational Pharmacology, Istituto Italiano di Tecnologia (IIT), 16163 Genova, Italy; ∥ Telethon Institute of Genetics and Medicine (TIGEM), 80078 Pozzuoli, Italy; ⊥ Department of Translational Medical Sciences (DISMET), University of Naples Federico II, 80138 Naples, Italy

## Abstract

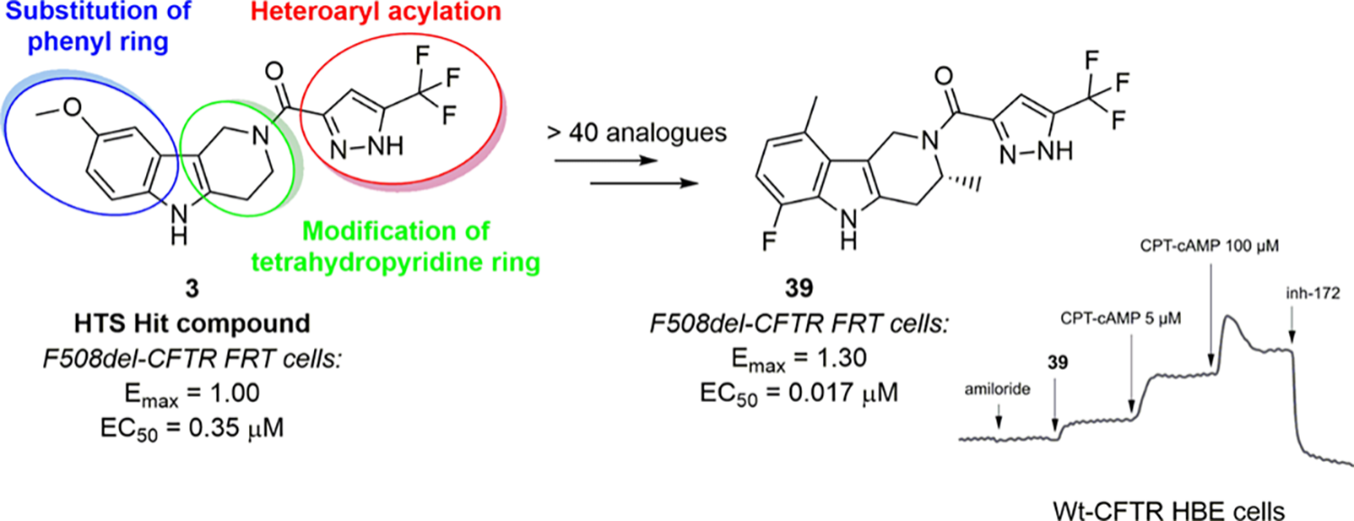

Cystic fibrosis (CF) is a life-threatening autosomal recessive disease, caused by mutations in the CF transmembrane conductance regulator (CFTR) chloride channel. CFTR modulators have been reported to address the basic defects associated with CF-causing mutations, partially restoring the CFTR function in terms of protein processing and/or channel gating. Small-molecule compounds, called potentiators, are known to ameliorate the gating defect. In this study, we describe the identification of the 2,3,4,5-tetrahydro-1*H*-pyrido[4,3-*b*]indole core as a novel chemotype of potentiators. In-depth structure–activity relationship studies led to the discovery of enantiomerically pure **39** endowed with a good efficacy in rescuing the gating defect of F508del- and G551D-CFTR and a promising *in vitro* druglike profile. The *in vivo* characterization of γ-carboline **39** showed considerable exposure levels and good oral bioavailability, with detectable distribution to the lungs after oral administration to rats. Overall, these findings may represent an encouraging starting point to further expand this chemical class, adding a new chemotype to the existing classes of CFTR potentiators.

## Introduction

**Figure 1:**
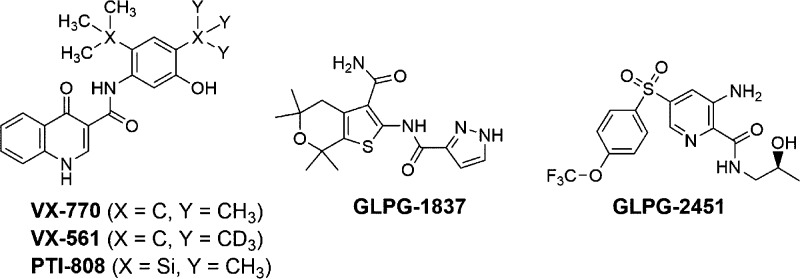
Chemical structure of known CFTR potentiators.

Cystic fibrosis (CF) is the most frequent life-threatening autosomal recessive disease in Caucasians, caused by loss-of-function mutations in the CF transmembrane conductance regulator (*cftr*) gene, encoding for CFTR protein.^1,2^ CFTR is a cAMP-regulated chloride channel expressed at the apical membrane of epithelial cells, where it provides a route for electrogenic anion flux, thus regulating the composition and volume of epithelial secretions.^3,4^ CF is a multiorgan disease, affecting the lungs, pancreas, liver, and other organs.^2^ More than 2000 mutations have been described in the *cftr* gene; however, the pathogenicity has been demonstrated only for approximately 300 mutations.^5^ CF mutations cause the loss of function of CFTR protein by affecting its synthesis, trafficking, or its function as an anion channel.^6^ According to the mechanism causing CFTR dysfunction, CF mutations have been grouped into seven different classes: mutations introducing a premature stop codon (class I), mutations causing protein misfolding (class II), mutations causing defective channel gating (class III), mutations causing defective channel conductance (class IV), mutations leading to aberrant mRNA splicing (class V), mutations causing reduced stability at the plasma membrane (class VI), and mutations resulting in no mRNA expression (class VII).^7^ Despite this clear classification, the majority of CF mutations cause CFTR dysfunction by multiple mechanisms,^8^ as in the case of the deletion of phenylalanine 508 (F508del), the most frequent mutation among CF patients.^9^ Indeed, F508del causes a folding defect, leading to premature protein degradation.^10,11^ In addition, when F508del-CFTR is forced to traffic to the plasma membrane, for example, by rescue maneuvers, the mutant protein shows reduced stability because of the peripheral protein quality control mechanisms^12^ and defective channel gating.^13,14^ Other mutations, however, are associated with a single mechanism of CFTR dysfunction, as in the case of the class III mutation G551D, which causes a severe CFTR channel gating defect.^15^ Druglike small molecules, known as “CFTR modulators”, can target these specific defects caused by CFTR mutations restoring, at least partially, the CFTR function.^16^ The maturation defect can be rescued by small molecules called correctors, such as VX-809^17^ or VX-661,^18^ while the gating defect can be corrected by small molecules called potentiators, such as VX-770 (Figure 1).^19^

Together with VX-770, other small-molecule potentiators have been reported ameliorating gating defects of mutant CFTR.^20−23^ However, only a few have progressed to the stage of evaluation in clinical trials. Among them, the corresponding nona-deuterated (VX-561)^24^ and bis-(trimethylsilyl) (PTI-808)^25^ analogues of VX-770 or AbbVie-Galapagos GLPG-1837^26,27^ and GLPG-2451^27^ compounds have successfully entered clinical trials in CF patients with different gating mutations (Figure 1).

Presently, VX-770 is the only potentiator drug that has been approved for monotherapy of different mutants displaying a gating defect.^28^

Aiming to add new small-molecule compounds to the existing classes of CFTR potentiators, expanding the portfolio of modulators available to CF patients, we embarked on a drug discovery effort to the identification of novel chemotypes endowed with a promising pharmacological profile. After a high-throughput screening (HTS) campaign, few structurally diverse small-molecule hits were identified; among them, the most promising ones shared the 2,3,4,5-tetrahydro-1*H*-pyrido[4,3-*b*]indole (or 1,2,3,4-tetrahydro-γ-carboline) core structure.

In this work, we disclose the identification and an extended structure–activity relationship (SAR) study of tetrahydro-γ-carboline derivatives, which led to the discovery of novel CFTR potentiators, characterized by a nanomolar activity.

## Results and Discussion

A screening collection of 11,334 maximally diverse small-molecule compounds was assembled starting from a set of ca. 300,000 commercially available molecules belonging to the diversity subsets of major vendors. A series of more and more stringent filters were applied in order to discard compounds with suboptimal druglike properties, and those containing chemically reactive moieties, unstable and known cytotoxic groups, and frequent hitters (e.g., PAINS).^29−32^ A subsequent stepwise clustering protocol based on an unweighted pair group method with arithmetic mean (UPGMA) hierarchical agglomerative algorithm^33^ allowed for the selection of the final set of molecules suited for HTS.

**Figure 2:**
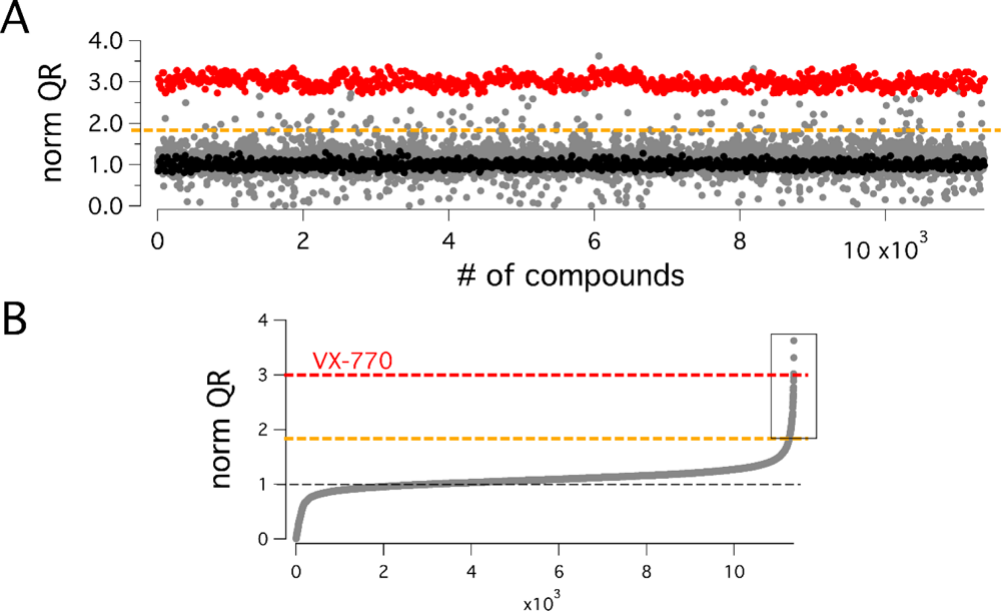
Discovery of mutant CFTR potentiators by HTS. (A) Summary of results obtained by screening the entire chemical library on FRT cells expressing F508del-CFTR and HS-YFP, followed by 24 h of incubation at 32 °C to rescue the trafficking defect. The graphs report the normalized HS-YFP quenching rate (QR), reflecting CFTR-dependent iodide influx, for cells treated with forskolin (10 μM) plus test compounds (5 μM; gray dots), VX-770 (1 μM; red dots), or vehicle [dimethyl sulfoxide (DMSO), black dots]. (B) Ordered distribution of the QR scores measured, for each test compound, in the screening and displayed in (A).

The chemical library was screened in duplicate on a Fischer rat thyroid (FRT) cell line, stably coexpressing F508del-CFTR and the halide-sensitive yellow fluorescent protein, HS-YFP.^23,34,35^ To overcome the F508del trafficking defect, cells were initially incubated for 24 h at 32 °C and then acutely treated (15–30 min) with a single compound (5 μM) in combination with forskolin (10 μM). After stimulation, the F508del-CFTR activity in the plasma membrane was calculated by measuring the rate of fluorescence quenching arising from iodide influx.^34^ This activity was compared to that of wells containing forskolin alone (negative control) or forskolin plus VX-770 (1 μM, as a positive control) (Figure 2A). Analysis of screening performance with the *Z*′ method gave a score of 0.6, which can be considered optimal for this type of assay. The activity scores for each compound were then plotted as an ordered distribution (Figure 2B). All compounds whose average activity calculated from the two rounds of screening was greater than 185% with respect to the negative control (i.e., forskolin alone) were considered as primary hits. The screening detected 104 putative potentiators, with some compounds showing a promising initial activity comparable to VX-770 (Figure 2B).

**Figure 3:**
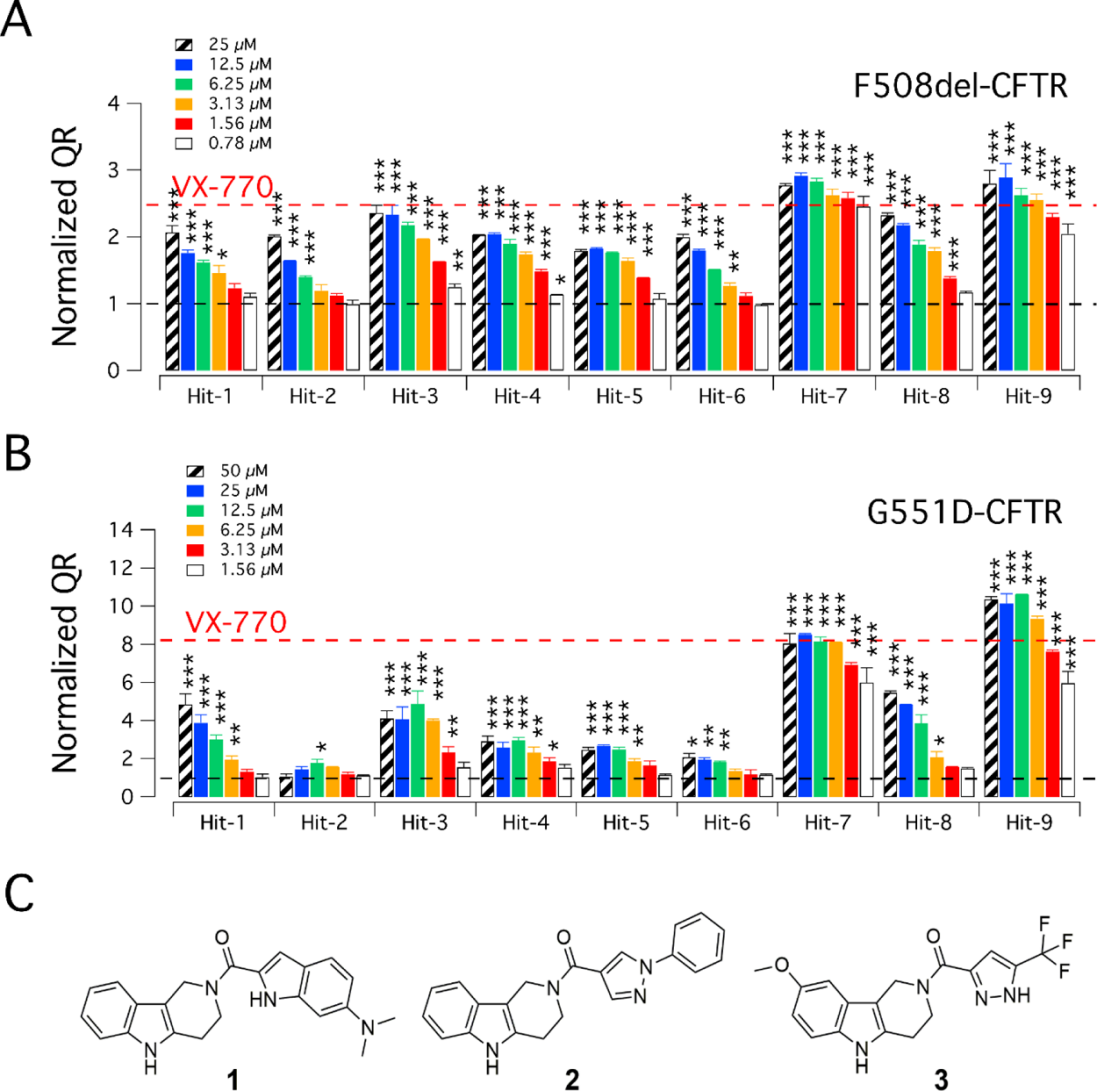
Identification of novel potentiators of mutant CFTR. Dose–response relationships for selected compounds on (A) F508del-CFTR FRT cells rescued at 32 °C for 24 h and (B) on G551D-CFTR FRT cells, compared to VX-770 (1 μM for F508del and 5 μM for G551D). (C) Structures of potentiators *Hit-7* (**1**), *Hit-8* (**2**), and *Hit-9* (**3**), selected for further SAR evolution. The data are expressed as mean ± standard deviation (SD) (*n* = 9; from three independent experiments, each one having three biological replicates). Statistical significance was tested by parametric analysis of variance (ANOVA), followed by the Dunnett multiple comparisons test (all groups against the control group). Symbols indicate statistical significance vs control (DMSO-treated): ***p < 0.001, **p < 0.01, and *p < 0.05.

The primary hits were confirmed by testing them at different concentrations, in order to extrapolate the dose–response relationship. First, we focused on the F508del mutant. The compounds were tested in the low micromolar range, in the presence of forskolin (10 μM), on F508del-CFTR FRT cells following rescue of the trafficking defect by low-temperature incubation, as done for the primary screening. The data for selected test compounds are shown in Figure 3A. The most interesting hits were subsequently tested for their ability to overcome the more severe gating defect of pure class III mutant using FRT cells stably expressing G551D-CFTR (Figure 3B). Interestingly, some compounds were effective on both types of mutants with a strong increase in CFTR activity that in some cases approached the effect of VX-770 (Figure 3A,B).

Notably, three compounds (*Hit-7* (**1**), *Hit-8* (**2**), and *Hit-9* (**3**), Figure 3C) sharing the same chemical scaffold (i.e., 2,3,4,5-tetrahydro-1*H*-pyrido[4,3-*b*]indole) were confirmed as hits on both cell types, when retested with the HS-YFP assay to extrapolate the dose–response relationship.

This finding, besides being a promising preliminary data in this screening campaign, confirmed that this specific chemotype could be considered a reliable starting point to further evolve a newly identified chemical class. In particular, derivatives **1** and **3** showed a good efficacy and an interesting sub-micromolar potency (Figure 3).

**Figure 4:**
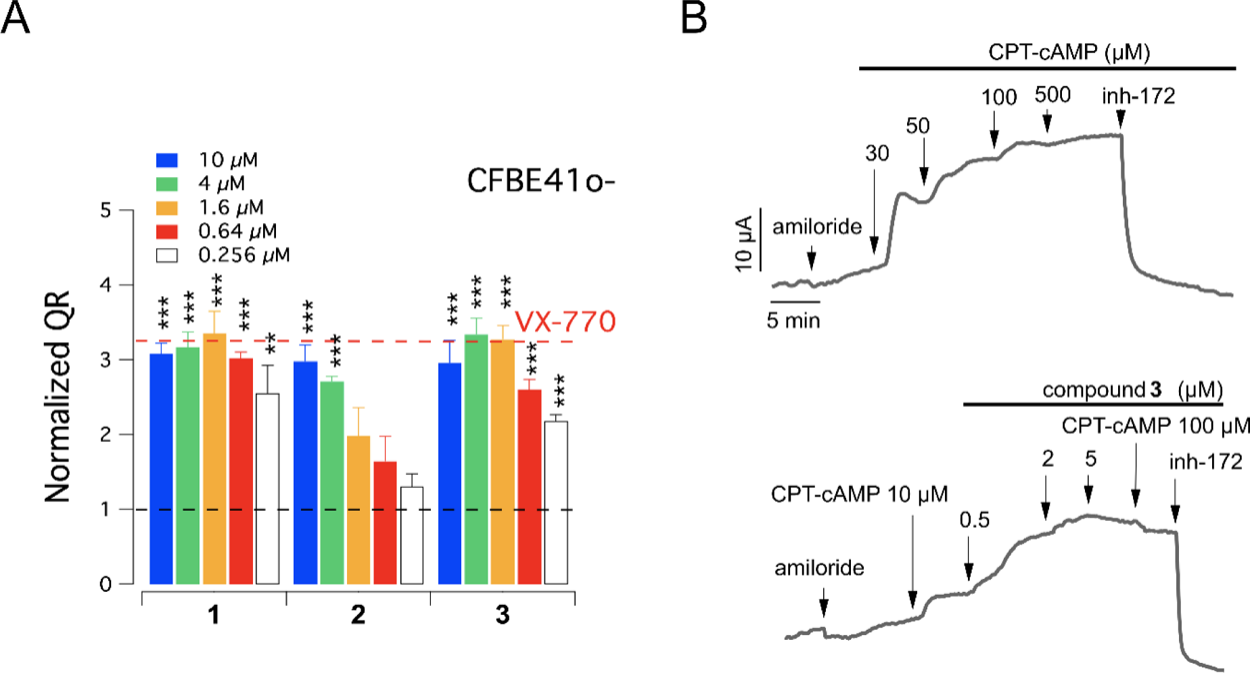
CFTR activation by tetrahydro-γ-carbolines on bronchial epithelial cells. (A) Dose–response relationships for selected compounds, compared to VX-770 (1 μM), on F508del-CFTR CFBE41o-cells rescued at 32 °C for 24 h. The data are expressed as mean ± SD (*n* = 9; from three independent experiments, each one having three biological replicates). Statistical significance was tested by parametric ANOVA, followed by the Dunnett multiple comparisons test (all groups against the control group). Symbols indicate statistical significance vs control (DMSO-treated): ***p < 0.001 and **p < 0.01. (B) Representative traces show the response of CFTR to stimulation with the indicated concentrations of CPT-cAMP and compound **3**. The currents stimulated by compound **3** and CPT-cAMP were blocked by the selective CFTR inhibitor-172. Each experimental condition was tested in three independent experiments, each one performed with three biological replicates.

The preliminary activity of these three hits was further evaluated in secondary screening assays. Although featuring the same common scaffold, in order to possibly discriminate the most interesting hit for further structural investigations, the compounds belonging to the family of tetrahydro-γ-carbolines were tested on more relevant cell models. First, the hits were tested on an immortalized bronchial epithelial cell line (CFBE41o-) stably expressing F508del-CFTR and HS-YFP. Acute stimulation with the compounds, in the presence of forskolin to increase the intracellular cAMP content, resulted in a dose-dependent activation of mutant CFTR (Figure 4A), with compounds **1** and **3** being the most effective.

Subsequently, the ability of compounds to elicit CFTR-mediated chloride secretion was verified on primary human bronchial epithelial (HBE) cells, from non-CF individuals, in short-circuit current experiments. In this respect, it should be noticed that potentiators are also able to stimulate wild-type CFTR, provided that a submaximal cAMP-dependent stimulation is applied.^20,22^ Accordingly, cells were stimulated with a submaximal concentration of the cAMP analogue, CPT-cAMP, followed by increasing concentrations of the potentiator hit.^20,22^ In such experimental conditions, compound **3** was able to stimulate CFTR-mediated chloride current of similar amplitude as that elicited by maximal cAMP stimulation, as confirmed by using the specific CFTR inhibitor-172 (Figure 4B).

**Figure 5:**
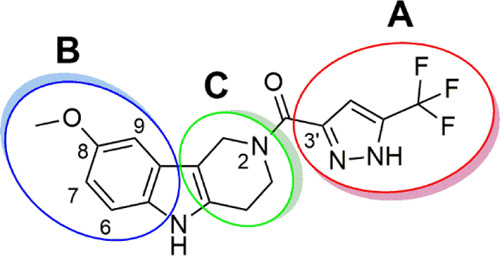
Sites of chemical modifications on the structure of **3** to explore the SAR of the tetrahydro-γ-carboline class.

Based on these initial, promising findings achieved in HBE experiments, compound **3** was selected as the reference molecule to explore the SAR around this chemotype, aiming to improve potency and efficacy of this novel class of CFTR potentiators. The SAR study focused on the investigation of the role of the heteroaromatic carboxylic acid (**A**) acylating the position *N*^2^ of the tetrahydro-γ-carboline, the substitution pattern of the phenyl ring (**B**), and the modification of the tetrahydropyridine portion (**C**) (Figure 5).

The role of the heteroaryl group (**A**) acylating the position 2 of the tetrahydro-γ-carboline was initially explored by modifying the substituent on the pyrazolyl residue and replacing the pyrazole with other rings, while keeping the 8-methoxy carboline moiety unmodified. The activity of each compound is described in terms of normalized maximal efficacy (*E*_max_), the maximum fold increase in the rescue of F508del-CFTR activity with respect to hit **3**, and potency (EC_50_), the concentration producing half-maximal efficacy. The chemical structures and the data of the first set of compounds are reported in Table 1.

**Table 1:**
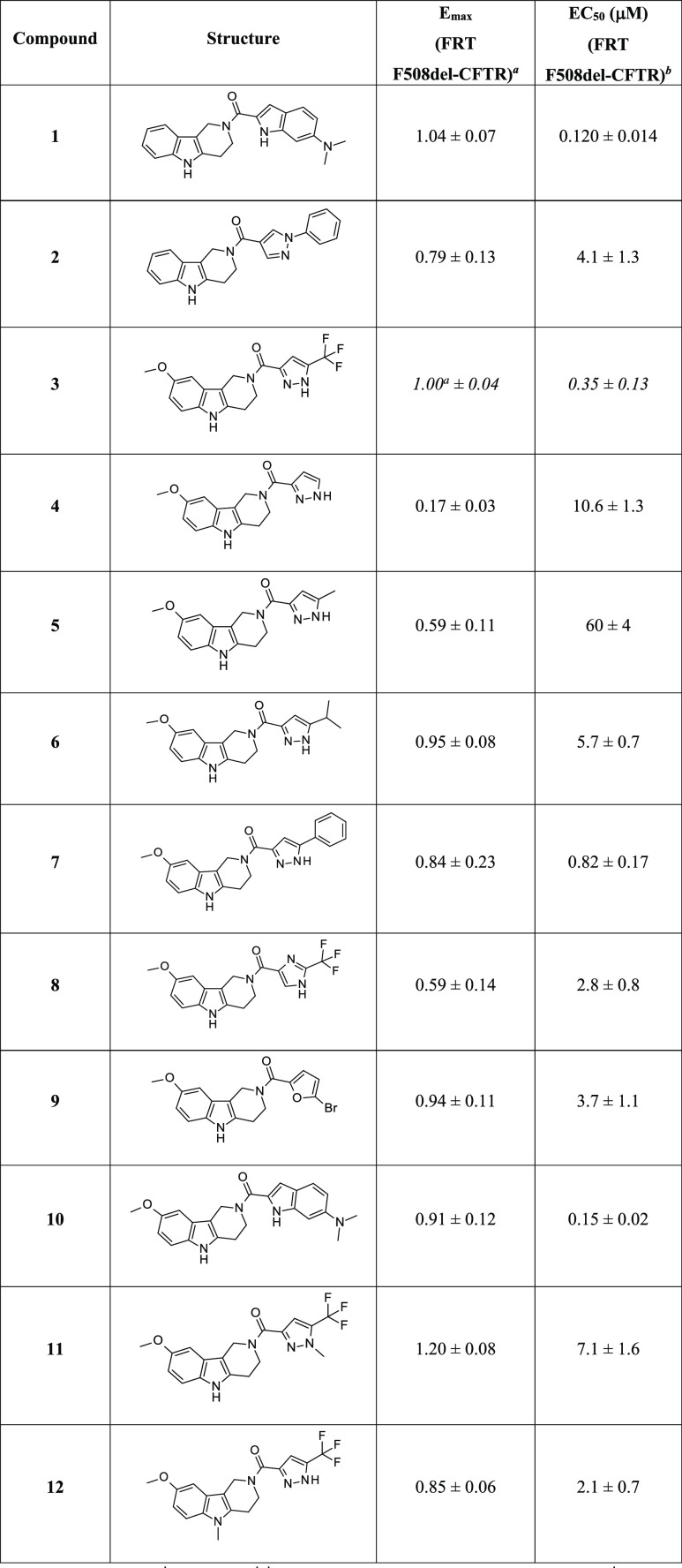
Chemical Structure, Efficacy (*E*_max_), and Potency (EC_50_) Data of Tetrahydro-γ-carbolines **1–12** in F508del-CFTR FRT Cells

a *E*_max_ values (normalized for the activity of analogue **3**).

b EC_50_ values, calculated from experiments performed as in Figure 3A. Data are expressed as mean ± SD (*n* = 3–6).

The removal (**4**, *E*_max_: 0.17; EC_50_: 10.6 μM) or the replacement of the trifluoromethyl moiety in position 5′ of the pyrazolyl residue with a methyl (**5**, *E*_max_: 0.59; EC_50_: 60 μM), isopropyl (**6**, *E*_max_: 0.95; EC_50_: 5.7 μM), and phenyl (**7**, *E*_max_: 0.84; EC_50_: 0.82 μM) group led to a marked loss of activity with respect to compound **3**. Replacing the 5′-trifluoromethyl-pyrazol-3′-yl residue with other heteroaromatic groups, such as a 2′-trifluoromethyl imidazolyl (**8**) or a 5-bromo furanyl (**9**) derivative, resulted in no improvements in activity, showing essentially a marked drop in potency (EC_50_: 2.8 and 3.7 μM, respectively). Not surprisingly, the insertion of a substituted indolyl moiety as in compound **10** allowed to retain a good effect on CFTR function (*E*_max_: 0.91; EC_50_: 0.15 μM), as seen for hit compound **1**. Finally, the alkylation of either *N*^1′^ (**11**) or *N*^5^ (**12**) position with a methyl residue resulted in a marked reduction of activity (**11**, *E*_max_: 1.2; EC_50_: 7.1 μM; **12**, *E*_max_: 0.85; EC_50_: 2.1 μM), suggesting that these hydrogen atoms may be involved in key H-bond interactions with the biological target or insufficient space exists for accommodating the methyl group.

Having demonstrated the importance of the 5′-trifluoromethyl-pyrazol-3′-yl residue as an ideal acylating group of the 8-methoxy-tetrahydro-1*H*-pyrido[4,3-*b*]indole derivative in targeting the gating defects caused by CF mutations, the importance of the substitution pattern on the phenyl ring (**B**, Figure 5) was investigated by the synthesis of a number of phenyl-substituted analogues (Table 2).

**Table 2:**
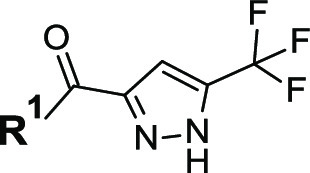

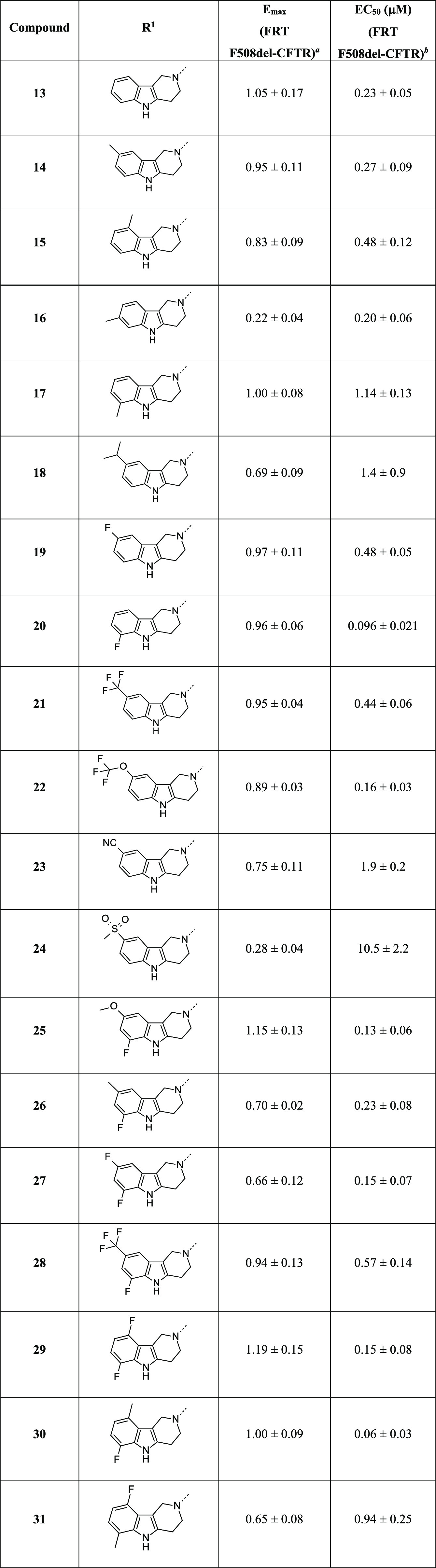
Chemical Structure, Efficacy (*E*_max_), and Potency (EC_50_) Data of Tetrahydro-γ-carbolines **13–31** in F508del-CFTR FRT Cells

a *E*_max_ values (normalized for the activity of analogue **3**).

b EC_50_ values, calculated from experiments performed as in Figure 3A. Data are expressed as mean ± SD (*n* = 3–6).

Initially, the importance of the substituent at position 8 of compound **3** was evaluated by preparing analogues where the methoxy residue was replaced by a hydrogen atom (**13**) or a methyl group (**14**). These changes led to an overall retention in the efficacy (*E*_max_ normalized to **3**), while potency was conserved for compound **14** (EC_50_: 0.27 μM) and slightly increased for analogue **13** (EC_50_: 0.23 μM). Based on these preliminary data achieved for 8-methyl-substituted tetrahydro-γ-carboline **14**, the effect of the position of the methyl substitution on the phenyl ring was investigated. While the efficacy changed progressively from an equal (6-methyl, **17**) to a lower (7-methyl, **16**) via a moderate value (9-methyl, **15**), an opposite trend was observed for potency with EC_50_ values progressively decreasing from 7-methyl (**16**, EC_50_: 0.2 μM) to 8-methyl (**14**, EC_50_: 0.36 μM) to 9-methyl (**15**, EC_50_: 0.48 μM) to 6-methyl (**17**, EC_50_: 1.14 μM) substituted compound (Table 2).

Having demonstrated that position 8 of the γ-carboline moiety could affect positively both efficacy and potency, a number of new analogues of compounds **3** (8-OMe) and **14** (8-Me) were synthesized in order to gain additional information on the SAR of this class. The investigation of the importance of the substituent at position 8 was broadened by preparing derivatives where the methoxy residue was replaced by an isopropyl (**18**), a fluoro (**19**), a trifluoromethyl (**21**), a trifluoromethoxy (**22**), a cyano (**23**), and a methylsulfonyl (**24**) moiety. While compounds **19** and **21** showed potency and efficacy comparable to **3**, a relevant drop in the overall activity was observed with derivatives featuring a sterically demanding isopropyl (**18**) and methylsulfonyl (**24**) groups or the polar linear cyano residue (**23**). On the contrary, the 8-trifluoromethoxy (**22**) derivative showed improved potency (EC_50_: 0.16 μM) and a comparable normalized efficacy (*E*_max_ = 0.89) with respect to **3**. Interestingly, compound **20**, bearing a fluorine atom at position 6, turned out to be the most potent analogue within this small set of mono-substituted γ-tetrahydro-carbolines, displaying a double-digit nanomolar potency (EC_50_ = 0.096 μM), while retaining a similar efficacy (*E*_max_ = 0.96) to analogue **3**.

As a further step in the exploration of SAR within this chemotype, disubstituted phenyl derivatives were also explored. Based on the promising effect shown by **20** in dose–response data in F508del-CFTR FRT cells, a small set of disubstituted compounds bearing a fluorine atom at position 6 was prepared and tested. The introduction of a second substituent on the phenyl ring proved in general to be beneficial, leading to some compounds with efficacy comparable or superior to **3** and potency in the double-digit nanomolar range.

Trying to possibly gain an additive effect by introducing previously identified substituents, the influence on the activity of 6-fluoro (as in **20**) was combined with a 8-methoxy, a 8-methyl, and a 8-fluoro group, as in disubstituted analogues **25**, **26**, and **27** (Table 2). Unfortunately, these modifications turned out to be not so beneficial in terms of overall activity, showing in all cases a significant drop in potency when compared to mono-substituted analogue **20**. A similar effect was displayed by the insertion of a trifluoromethyl group at position 8, as in **28**, which caused a marked 6-fold drop in potency (EC_50_: 0.57 μM) with respect to **20** (Table 2).

While retaining a fluorine atom at position 6, as in **20**, the SAR study around this scaffold was further expanded by modification of the substitution pattern on the phenyl ring of the tetrahydro-γ-carboline core. Accordingly, whereas the insertion of another fluorine in position 9 (**29**) negatively influenced the potency (EC_50_ = 0.15 μM), the replacement of a hydrogen with a methyl group in the same position resulted in a double-digit nanomolar active disubstituted derivative **30** (EC_50_ = 0.06 μM), with more than 6-fold increase in potency and comparable efficacy to hit **3**. To firmly prove that the 6-fluoro-9-methyl disubstitution pattern, as in **30**, was convenient to maintain the activity, we swapped the position of fluorine and methyl group, leading to compound **31**. This modification resulted to be detrimental for efficacy and led to more than 15-fold decrease in potency (EC_50_ = 0.94 μM) (Table 2).

As the next step in the exploration of SAR of the class, modifications in the tetrahydropyridine ring (**C**, Figure 5) were investigated, retaining the optimal 6-fluoro-9-methyl substitution on the phenyl ring. Remarkably, the introduction of a methyl group at position 1 or 3 of the tetrahydropyridine ring, as in racemic **32** and **33**, resulted in a considerable boost in activity, as shown by the good efficacy and low double-digit nanomolar potency (EC_50_ = 0.03 and 0.022 μM, respectively) (Table 3). On the contrary, the insertion of a *gem*-dimethyl moiety at 3-position of the tetrahydropyridine ring, as for compound **34**, negatively affected both potency (EC_50_ = 1.91 μM) and efficacy (*E*_max_ = 0.79) with respect to derivative **30**.

**Table 3:**
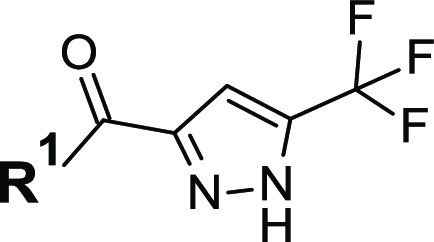

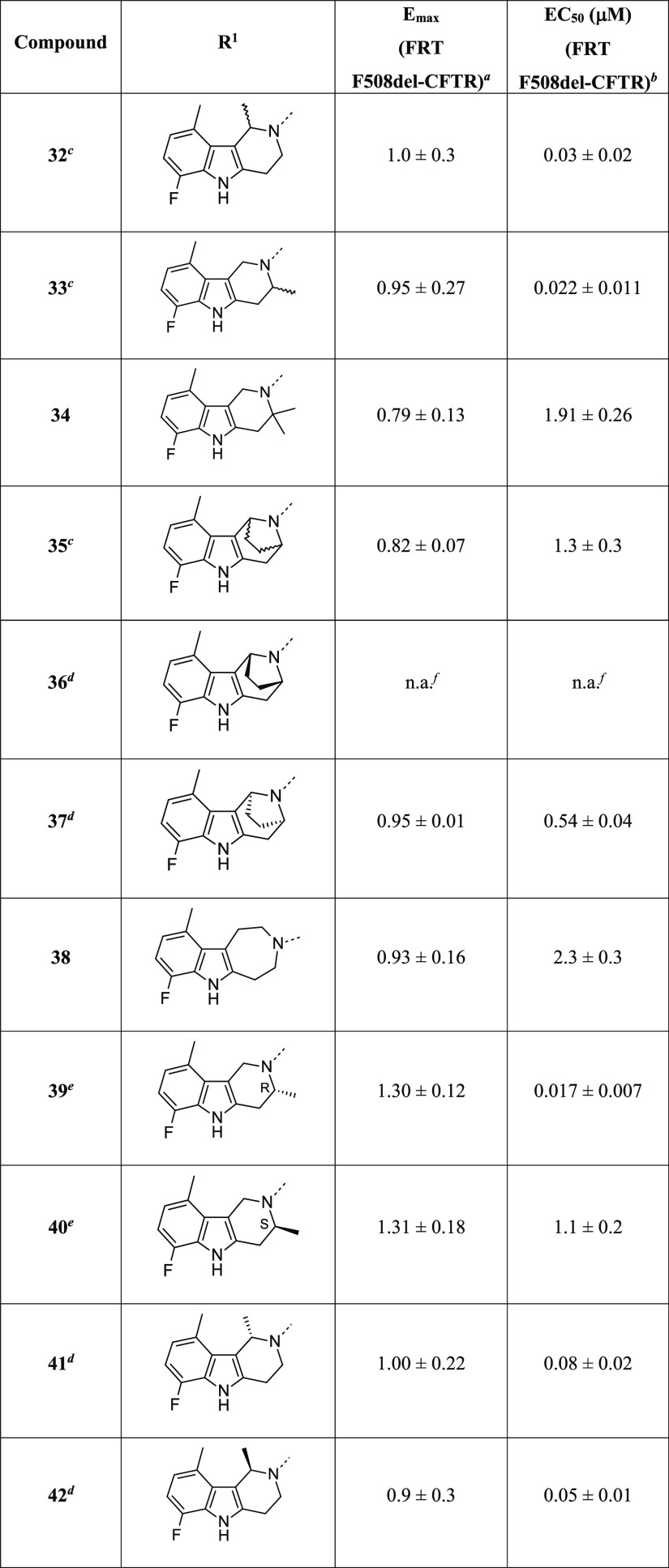
Chemical Structure, Efficacy (*E*_max_), and Potency (EC_50_) Data of Tetrahydro-γ-carbolines **32–42** in F508del-CFTR FRT Cells

a *E*_max_ values (normalized for the activity of analogue **3**).

b EC_50_ values, calculated from experiments performed as in Figure 3A. Data are expressed as mean ± SD (*n* = 3–6).

c Racemic compound.

d Absolute configuration not determined and arbitrary drawn.

e Absolute configuration known.

f n.a.: not active (up to 20 μM).

A more sterically demanding modification of the γ-carboline nucleus was explored by incorporating an ethylene bridge in the tetrahydropyridine ring, as for racemic compound **35**. This modification leads to a more rigid skeleton, possibly resulting in increased affinity at the target binding site.^36,37^ Unfortunately, this structural change was detrimental for both efficacy and potency with respect to **30**. The separation of the racemate into the two pure enantiomers did not result in any improvement because only one of them (**37**) retained some activity, whereas the opposite isomer **36** was completely inactive (Table 3).

Trying to evaluate the effect on the activity of the size of the heterocyclic ring, the corresponding tetrahydro-azepino-indole derivative **38** was synthesized and tested; the compound showed a marked 38-fold drop in potency with respect to the tetrahydropyridine analogue **30**.

Based on the promising data in improving the gating of mutant F508del-CFTR in FRT cells shown by racemic tetrahydro-γ-carbolines **32** and **33**, the corresponding pure enantiomers were synthesized and tested. Notably, a strong difference (>100-fold) in the biological activity was displayed by these chiral analogues. While (*S*)-enantiomer **40** (distomer) resulted in a marked loss in potency but similar efficacy (*E*_max_: 1.31, EC_50_: 1.1 μM) to racemic **33**, the corresponding (*R*)-enantiomer **39** (eutomer) showed a comparable activity with respect to racemate, retaining a low double-digit nanomolar potency (EC_50_: 0.017 μM) (Table 3).

A slightly different pattern of activity was observed with the 1-methyl-substituted enantiomers **41** and **42**, which showed no major differences in terms of efficacy and potency, also when compared to racemic **32**.

Within this small set of alkyl-substituted analogues, the results observed with compounds **41** and **42**, along with the likelihood of 1-alkyl substituted γ-carboline analogues possibly undergoing epimerization in acidic aqueous media,^38^ convinced us to convey our attention primarily on compound **39**.

**Figure 6:**
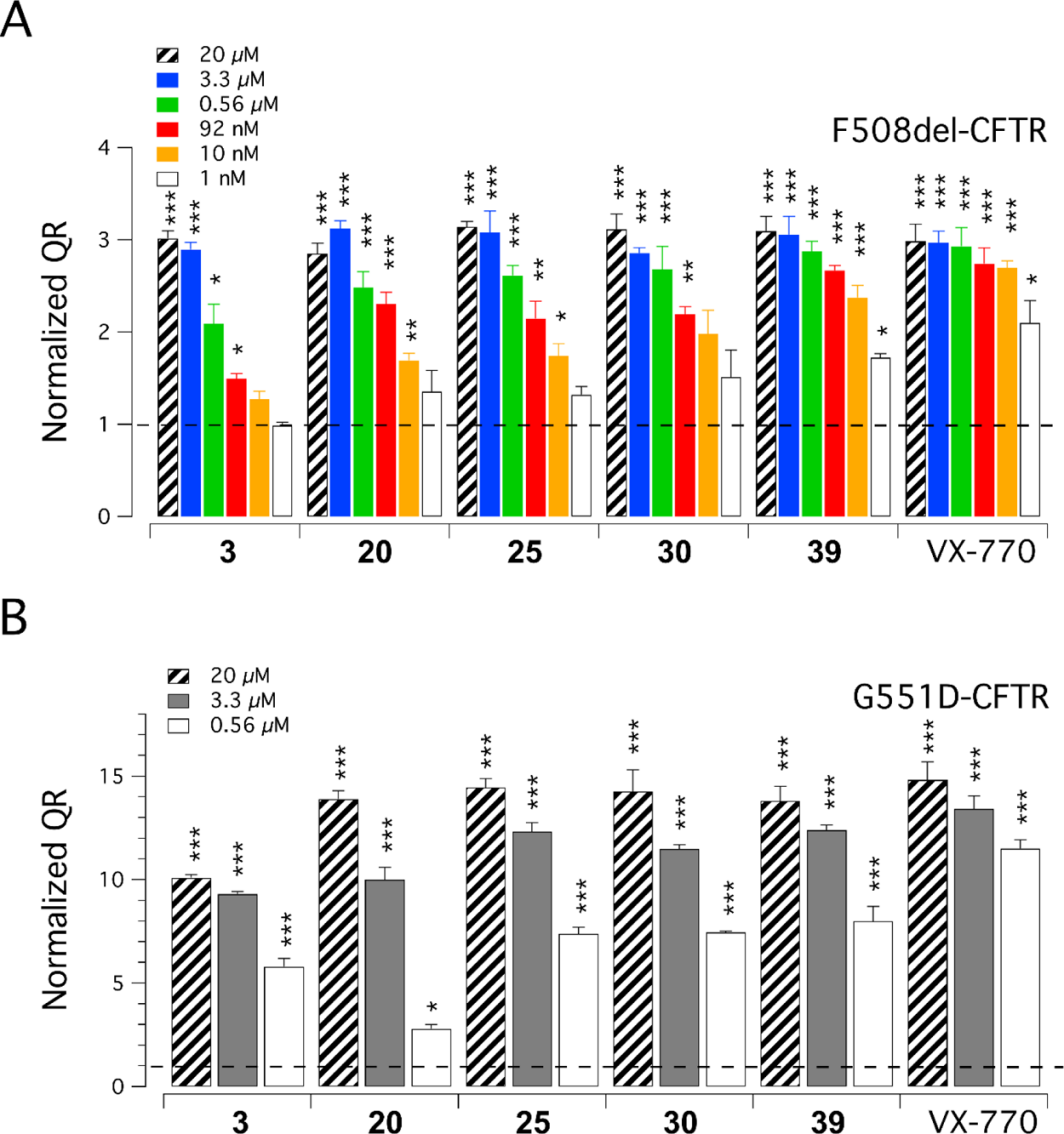
Activity of selected tetrahydro-γ-carboline potentiators (**3**, **20**, **25**, **30**, and **39**) and, for comparison, VX-770 on (A) F508del-CFTR FRT and (B) on G551D-CFTR FRT cells. The data are expressed as mean ± SD (*n* = 9; from three independent experiments, each one having three biological replicates). Statistical significance was tested by parametric ANOVA, followed by the Dunnett multiple comparisons test (all groups against the control group). Symbols indicate statistical significance vs control (DMSO-treated): ***p < 0.001, **p < 0.01, and *p < 0.05.

A small set of selected potentiators with good efficacy and potency on F508del-CFTR FRT cells (Figure 6A) was also tested at three concentrations on G551D-CFTR FRT cells (Figure 6B). With the only exception of compound **3**, the potentiators displayed efficacy comparable to VX-770 at the highest concentration (20 μM), with analogue **39** being the most potent one, although the affinity was lower than that of VX-770.

In F508del-CFTR FRT cells, the selected potentiators displayed efficacy comparable to VX-770. Interestingly, enantiomer **39** resulted to be the most potent analogue within this set of novel tetrahydro-γ-carbolines, although showing a slightly lower activity than VX-770 when tested at 10 and 1 nM (Figure 6A). For these selected compounds, a very similar trend was also observed in G551D-CFTR FRT cells, where **39** showed a comparable pattern of activity at the highest concentrations (20–3.3 μM) and a decrease in activity at 0.56 μM when compared to VX-770 (Figure 6B).

**Figure 7:**
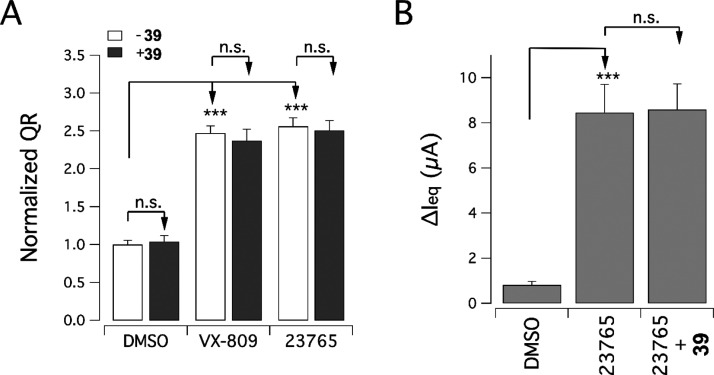
Potentiator **39** does not influence mutant F508del rescue by correctors VX-809 and **ARN23765**. The graphs report the (A) values of normalized QR measured in the YFP-based functional assay on CFBE41o-expressing F508del-CFTR treated for 24 h with VX-809 (1 μM) or **ARN23765** (10 nM) in the absence or presence of compound **39** (5 μM) and (B) equivalent short-circuit current (calculated from TEER/PD measurements) in F508del/F508del bronchial epithelial cells treated for 24 h with VX-809 (1 μM) or **ARN23765** (10 nM) in the absence or presence of compound **39** (0.5 μM). The data are expressed as mean ± SD (*n* = 9; from three independent experiments, each one having three biological replicates). Statistical significance was tested by parametric ANOVA, followed by the Tukey test (for multiple comparisons). Symbols indicate statistical significance: ***p < 0.001, n.s. (not significant) indicates *p* > 0.05.

Recent studies have shown that most potentiators have an undesired activity on F508del-CFTR protein processing/trafficking.^39,40^ According to these studies, chronic incubation with potentiators results in decreased activity of VX-809 as a corrector. To test the effect of our potentiators, we incubated F508del-CFTR CFBE41o-cells for 24 h with tetrahydro-γ-carboline **39** (5 μM) together with VX-809 (1 μM) and **ARN23765** (10 nM), the picomolar affinity corrector recently reported by our research team.^35^ Interestingly, cotreatment with potentiator **39** did not affect the rescue efficacy of VX-809 or **ARN23765** (Figure 7A).

A similar combination study was conducted on primary HBE cells from an F508del/F508del CF patient. The effect of the compound was assessed with the transepithelial electrical resistance and potential difference (TEER/PD) technique.^41^ Epithelia were treated for 24 h with a vehicle, a corrector **ARN23765** alone (10 nM), or **ARN23765** (10 nM) plus the potentiator **39** (0.5 μM). TEER and PD values were taken at resting, after the addition of apical amiloride, after maximal stimulation of F508del-CFTR activity (with forskolin plus genistein), and after blocking with PPQ-102. For each epithelium, we measured the difference in the short-circuit current, *I*_eq_ (calculated from the TEER and PD values), before and after blocking with PPQ-102 (Δ*I*_eq_). The results obtained on primary epithelia confirmed that the rescue of CFTR activity by the corrector **ARN23765** was not affected by coincubation with the novel potentiator **39** (Figure 7B).

**Figure 8:**
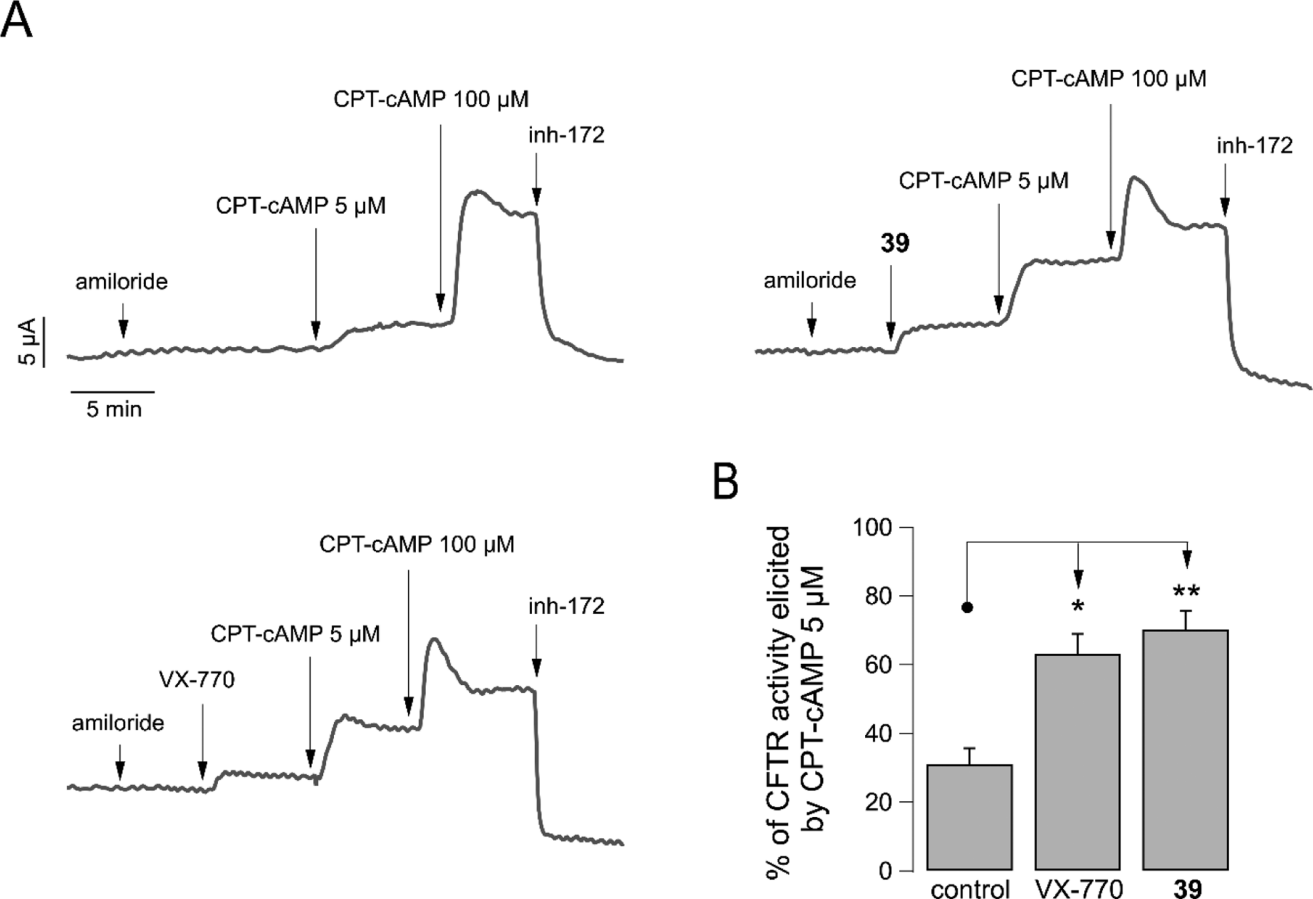
Efficacy of potentiator **39** on primary bronchial epithelial cells from non-CF individuals. (A) Representative traces of short-circuit current measurements showing the response of CFTR to stimulation with the indicated concentrations of CPT-cAMP in the absence or presence of potentiators **39** or VX-770 (1 μM for both compounds). (B) Bar graph showing the currents stimulated by submaximal concentration of CPT-cAMP measured in experiments performed as in (A). The data are expressed as mean ± SD (*n* = 9; from three independent experiments, each one having three biological replicates). Statistical significance was tested by parametric ANOVA, followed by the Dunnett multiple comparisons test (all groups against the control group). Symbols indicate statistical significance: **p < 0.01 and *p < 0.05.

Finally, potentiator **39** and VX-770 were further evaluated in primary HBE cells from non-CF individuals, using short-circuit current measurements, to assess the ability of compounds to maximally activate CFTR function in the presence of a submaximal cAMP stimulation. After the addition of amiloride to inhibit sodium absorption through the ENaC channel, the potentiators were added at the maximal effective concentration (1 μM) after which epithelia were stimulated with a submaximal concentration of CPT-cAMP (5 μM), followed by a maximal concentration of the cAMP analogue (100 μM) (Figure 8). We then measured the extent of CFTR-mediated chloride current elicited by submaximal cAMP stimulation in the presence of a potentiator and compared it to total CFTR-mediated chloride current, determined as the current inhibited by CFTR inhibitor-172. In parallel, other epithelia were stimulated only with the two concentrations of the cAMP analogue, in the absence of a potentiator. Interestingly, the fraction of CFTR-mediated chloride current activated by submaximal cAMP stimulation was significantly increased by more than 2-fold when epithelia were prestimulated with a potentiator (Figure 8). In this respect, VX-770 and compound **39** were similarly effective (Figure 8B).

The most interesting potentiators (**3**, **20**, **25**, **29**, **30**, and **39**), selected on the basis of their efficacy and potency in the HS-YFP assay on F508del-CFTR FRT cells, were profiled *in vitro* for their druglike properties. Kinetic solubility and metabolic stability in rat, dog, and human liver microsomes, in the presence of NADPH and UDPGA (only for human) as cofactors, were assessed along with an indication of potential for hepatotoxicity in HepG2 cells (Table 4).

**Table 4:** Kinetic Solubility, Liver Microsomal Stability, and Cytotoxicity of Selected Compounds

compound	kinetic solubility (μM)^a^	rat LM_NADPH *t*_1/2_ (min)^b^^,^^c^	dog LM_NADPH *t*_1/2_ (min)^b^^,^^c^	human LM_NADPH *t*_1/2_ (min)^b^^,^^c^	human LM_UDPGA *t*_1/2_ (min)^b^^,^^c^	HepG2 (% survival)^d^ (%)
**3**	237 ± 11	30 ± 1	>60 (68)	42 ± 11	n.a.^e^	>80
**20**	9 ± 4	>60 (64)	>60 (85)	>60 (78)	n.a.^e^	>80
**25**	2 ± 1	20 ± 1	>60 (90)	33 ± 1	n.a.^e^	>80
**29**	36 ± 5	50 ± 9	>60 (93)	>60 (60)	n.a.^e^	>80
**30**	<1	32 ± 1	>60 (92)	53 ± 2	>60 (75)	>80
**39**	<1	>60 (70)	>60 (92)	>60 (71)	>60 (79)	>80

a Kinetic solubility (PBS, pH 7.4; *n* = 3).

b 4.6 μM in liver microsomes (LM) with NADPH or UDPGA as cofactors, 0.1% DMSO.

c Data collected as *n* ≥ 3.

d Percentage of survival of HepG2 cells at 20 μM determined by CTG and MTT assays. Viability of HepG2 cells is expressed as percent survival of the vehicle-treated controls (given as 100%). Values are from one experiment, performed in three technical replicates.

e n.a.: not available.

In general, the selected compounds showed a low kinetic solubility (<40 μM), with the exception of the hit **3**, which exhibited high solubility (237 μM). The metabolic stability (phase I metabolism) in the presence of liver microsomes was generally quite good in both dog (*t*_1/2_ > 60 min) and human (*t*_1/2_ > 55 min), with the exception of compounds **3** and **25**, featuring both a methoxy group on the phenyl ring. Indeed, although quite promising in terms of overall activity (Table 2), derivative **25**, along with hit **3**, turned out to be the least stable compounds among the selected novel potentiators. However, their metabolic stability to oxidative metabolism was generally higher in the presence of human liver microsomes compared to rat (Table 4). Tetrahydro-γ-carbolines **30** and **39** turned out to be quite stable to phase II conjugation reactions, showing half-life values (*t*_1/2_ > 60 min) in human liver microsomes, with UDPGA as a cofactor. In addition, a considerable amount (>75%) of parent compound remained at the last time point (Table 4).

In order to assess a possible liver toxicity liability, the selected analogues were also tested in HepG2 cells at two concentrations (2.0 and 20 μM) for 24 h, along with a reference compound (rotenone). A reduction of cell viability to less than 80% was set as the threshold for estimation of cytotoxicity.^42^ None of the compounds induced a decrease in cell viability at the highest dose tested (20 μM), resulting in a cell survival >80% (Table 4).

Based on both its biological profile, showing a good efficacy in primary and secondary assays, and preliminary *in vitro* ADME properties, potentiator **39** was further evaluated in *in vivo* studies. The compound was dosed in Sprague–Dawley rats by intravenous (i.v.) administration, at a dose of 3 mg/kg, and by oral gavage (p.o.), at a dose of 10 mg/kg, to determine its pharmacokinetic profile (Table 5).

**Table 5:** Pharmacokinetic Parameters of **39** Following i.v and p.o. Administration to Male Sprague–Dawley Rats (*n* = 3 per Dose)

	i.v.	p.o.
dose (mg/kg)	3	10
*C*_max_ [ng/mL (μM)]	2792 (7.3)	769 (2.0)
*T*_max_ (min)	5	120
AUC [ng·min/mL (μM·h)]	138,913 (6.1)	139,538 (6.1)
*V*_d_ (L/kg)	2.39	
Cl (mL/min/kg)	19	
half-life (min)		
(elimination phase)	89	212
*F* (%)		30

After i.v. administration, the maximal plasma concentration was ca. 7.3 μM, with a volume of distribution (*V*_d_) of 2.39 L/kg, indicating an overall good distribution to the tissues. The low clearance (19 mL/min/kg) was in accordance with the good stability of **39** as shown in the rat liver microsomal stability assays. After oral administration, the compound reached the maximal plasma concentration at 2 h, and considerable levels of compound (>450 ng/mL, corresponding to a ca. 1.2 μM) were still present at 6 h postadministration, showing a relatively slow elimination phase (see Figure S1, Supporting Information). The exposure (area under the curve, AUC) over the time interval 0–4 h was 6.1 μM·h, resulting in a ca. 30% oral bioavailability, calculated over the same time interval.

Taking into account both the encouraging results in rescuing the gating defect in mutant CFTR in both primary and secondary biological assays, and the promising *in vivo* PK profile, we quantified the amount of compound **39** in rat lung tissue, the main target organ for CF treatment. The lung tissue distribution of potentiator **39** was investigated following administration of a single oral dose of 10 mg/kg to Sprague–Dawley rats. Supported by the data obtained in the pharmacokinetic study after oral administration, two time points (2 and 4 h) were selected for collecting plasma and lung tissue samples. The mean concentration vs. time profiles of compound **39** in plasma and lung tissue are reported in Figure S2 (see the Supporting Information). In this study, while the plasma levels at 2 and 4 h (1026 and 675 ng/mL, respectively) turned out to be in accordance with those observed in the PK experiment, the concentration of **39** in the lungs was quantified to be 4.5 and 2.2 ng/mg tissue at the two selected time points. Overall, this study demonstrated that tetrahydro-γ-carboline **39** distributed to the lung following oral administration, although with a low concentration.

Finally, based on both its biological and pharmacological profile, potentiator **39** could be fairly considered as a lead compound and a valuable starting point for further optimization of 1,2,3,4-tetrahydro-γ-carbolines as novel F508del-CFTR potentiators.

## Chemistry

**Scheme 1:**
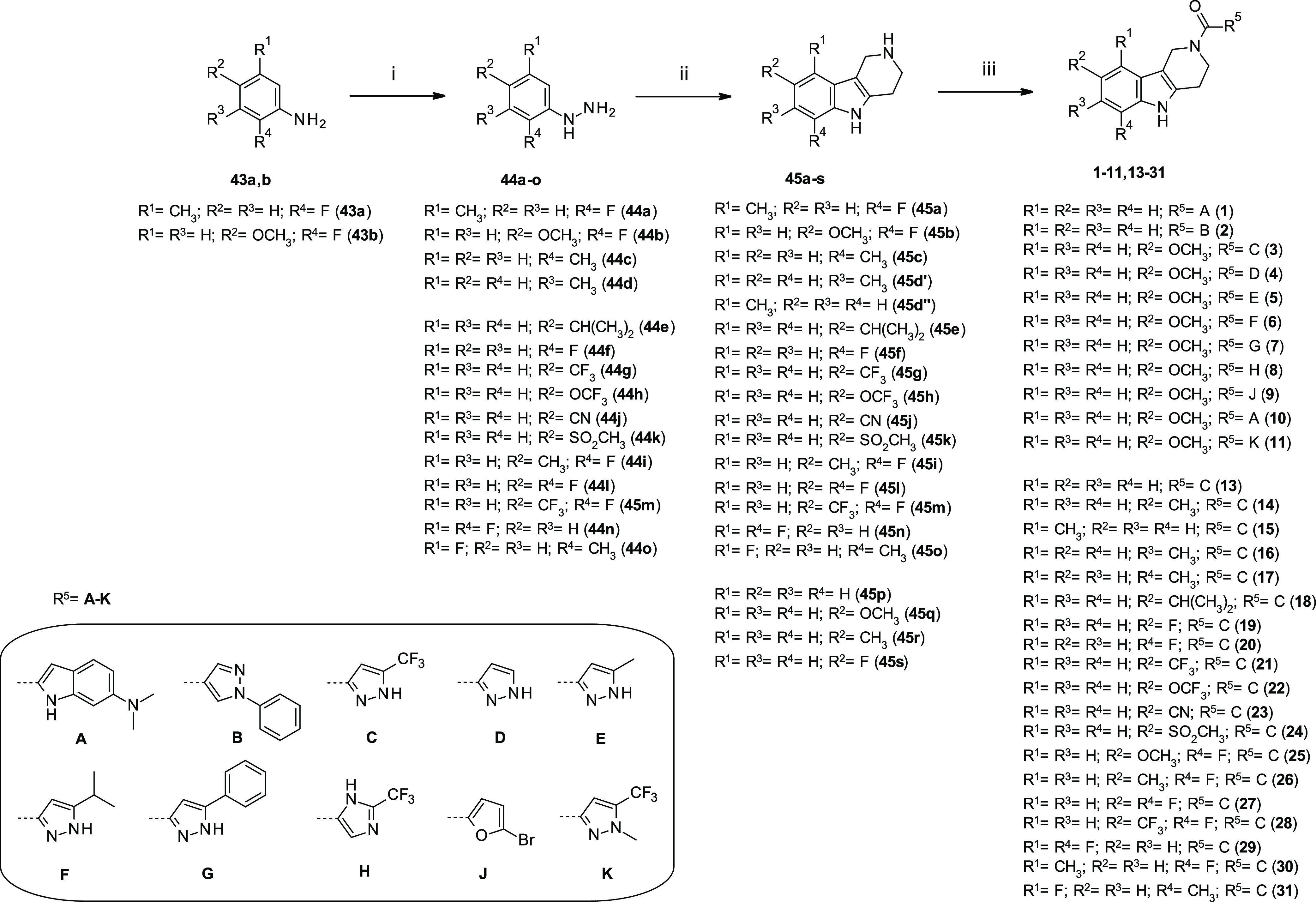
Synthesis of Tetrahydro-γ-carboline Amide Analogues **1–11** and **13–31** Reagents and reaction conditions: (i) HCl (36%), 0 °C, then NaNO_2_, H_2_O, SnCl_2_ in HCl (6 M), 0 °C to r.t., 24 h; (ii) protocol A: *tert*-butyl 4-oxopiperidine-1-carboxylate, HCl (36%), EtOH, 80 °C, and 16 h; protocol B: *tert*-butyl 4-oxopiperidine-1-carboxylate, EtOH, r.t., then 2,4,6-trichloro-1,3,5-triazine, 90 °C, 8 h; protocol C: *tert*-butyl 4-oxopiperidine-1-carboxylate, EtOH, 30 min, r.t., then removal of the solvent and addition of BF_3_·Et_2_O, AcOH, 90 °C, 16 h; (iii) protocol A: carboxylic acid, HATU, DIPEA, DMF, 0 °C, 10–30 min, then **45a,h,j,p–r** r.t., 16 h, 22–95%; protocol B: carboxylic acid, **45a–h,k-m,o–s**, Et_3_N, EDC·HCl, CH_2_Cl_2_, r.t., 16 h, 6–77%.

**Scheme 2:**
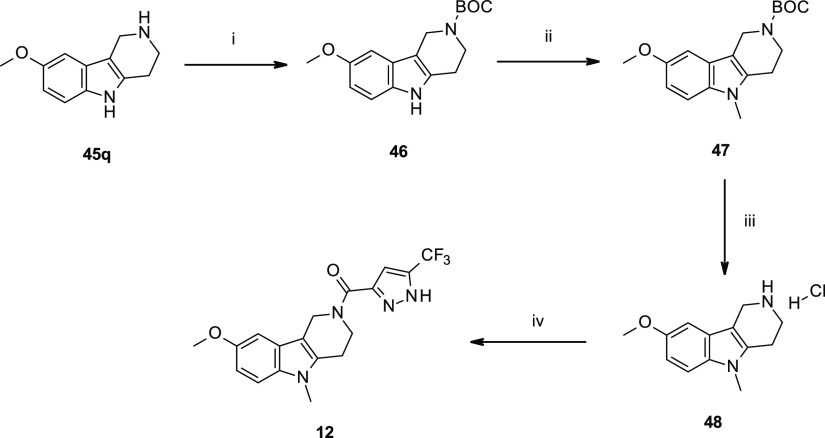
Synthesis of *N*^5^-Methyl-tetrahydro-γ-carboline Analogue **12** Reagents and reaction conditions: (i) (BOC)_2_O, DIPEA, CH_2_Cl_2_, 0 °C to r.t., 1 h; (ii) NaH (60% dispersion in mineral oil), CH_3_I, DMF, 0 °C to r.t., 16 h; (iii) HCl (4.0 M) in dioxane, DCM, r.t., and 20 h; and (iv) 5-trifluoromethyl-1*H*-pyrazole-3-carboxylic acid, HATU, DIPEA, DMF, 10 min, then **48**, r.t., 16 h, and 24%.

**Scheme 3:**
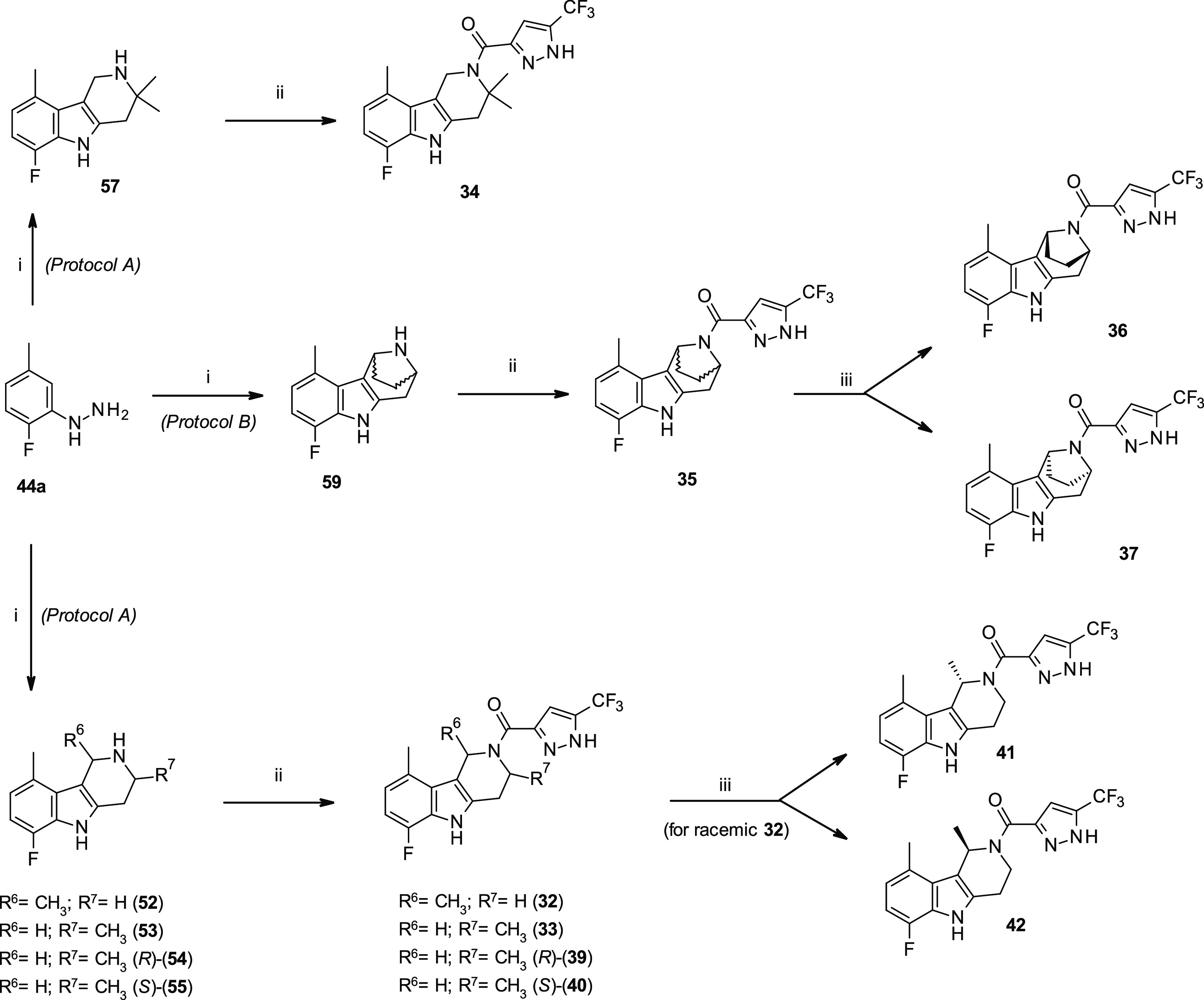
Synthesis of 6-Fluoro-9-methyl-Substituted γ-Carbolines **32–37** and **39–42** with Modified Tetrahydro-pyridine Ring Reagents and reaction conditions: (i) Protocol A: *tert*-butyl 2-methyl-4-oxo-piperidine-1-carboxylate [*rac*-**49**, (*R*)-**50**, and (*S*)-**51**] or *tert*-butyl 2,2-dimethyl-4-oxo-piperidine-1-carboxylate (**56**), toluene, 50 °C, then TsOH, 120 °C, and 24 h; protocol B: *tert*-butyl 3-oxo-8-azabicyclo[3.2.1]octane-8-carboxylate (**58**), HCl (36%), EtOH, 80 °C, and 16 h; (ii) 5-methyl-1*H*-pyrazole-3-carboxylic acid, HATU, DIPEA, DMF, 0 °C, 10–30 min, then **52–55**, **57**, **59**, r.t., 16 h, and 5–47%; (iii) semipreparative chiral separation: ChiralPak AD, *n*-heptane–EtOH (75:25) [for **36**, **37**, **41**, and **42**: absolute configuration not determined and arbitrary drawn].

The chemistry for the preparation of acylated 2,3,4,5-tetrahydro-1*H*-pyrido[4,3-*b*]indoles, as final compounds (**1–42**), is outlined in Schemes 1–3. Treatment of substituted γ-carbolines **45a–s** with heteroaryl carboxylic acids (**A–K**) following different coupling conditions afforded amides (**1–11**, **13–31**) in moderate to high yields (22–95%) (Scheme 1). Substituted tetrahydro-γ-carbolines used to synthesize final compounds were either commercially available (**45p–s**) or synthesized (**45a–o**) in a straightforward manner from the corresponding aryl-hydrazines (**44a–o**) and a suitable Boc-protected 4-piperidone via a Fischer-indole synthesis approach.^43,44^ The synthesis of tetrahydro-carbolines proceeds through an acid-catalyzed rearrangement of the enehydrazine form of an arylhydrazone, via a [3,3]-sigmatropic rearrangement, followed by cyclization and elimination of ammonia. Although simple heating of the two reacting partners in either ethanol/HCl or EtOH/2,4,6-trichloro-1,3,5-triazine gave in general the desired γ-carboline in moderate to high yields (Scheme 1), more forcing conditions (e.g., trifluoroboron etherate complex) were used with electron-poor hydrazines (see the Supporting Information).^45,46^

Two specific hydrazines (**44a,b**) were easily prepared via an *in situ* one-pot diazotation/reduction step from suited aniline (**43a,b**).^47^

The synthesis of *N*^5^-methyl amide **12** was accomplished in 16% overall yield after a four-step protocol by means of an *N*-alkylation of γ-carboline **45q**, as a key synthetic modification (Scheme 2).

Fischer-indole synthesis starting from phenyl hydrazine **44a** in the presence of racemic *N*-*tert*-butoxycarbonyl protected 2-methyl-piperidin-4-one (**49**), 2,2-dimethyl piperidin-4-one (**56**), or 8-azabicyclo[3.2.1]octan-3-one (**58**) led to the corresponding tetrahydro- (**52**, **53**, **57**) and bridged- (**59**)^36,37^ γ-carbolines. Not surprisingly, whereas mono-methyl intermediates **52** (1-methyl) and **53** (3-methyl) were obtained as a 2:8 regioisomeric mixture, 3,3-*gem*-dimethyl analogue **57** was afforded as the only regioisomer. γ-Carbolines **52**, **53**, **57**, and **59** were then converted to final compounds **32–35**, as racemates, by HATU-mediated coupling with 5-methyl-1*H*-pyrazole-3-carboxylic acid (Scheme 3).

Enantiomerically pure analogues **36** and **37** were obtained by semipreparative chiral separation starting from racemic **35**. The reaction with (*R*)- and (*S*)-2-methyl-piperidin-4-ones **50** and **51** afforded the corresponding methyl-substituted tetrahydro-pyridoindoles, as mixtures of 1-methyl and 3-methyl regioisomers. Chromatographic purification allowed to obtain pure regioisomers, which upon amide coupling with pyrazolyl carboxylic acid led to the desired substituted compounds. Whereas 3-methyl analogues (*R*)-**39** and (*S*)-**40** were afforded enantiomerically pure from the corresponding chiral intermediates **54** and **55**, a careful investigation revealed for 1-methyl substituted γ-carbolines a complete racemization of the stereogenic center, probably because of the relatively acidic character of allylic proton in C1-position, allowing a enamine–imine equilibrium during Fischer indole synthesis.^38^ Therefore, the corresponding 1-methyl-substituted compounds (*S*)-**41** and (*R*)-**42** were isolated as enantioenriched compounds after semipreparative chiral separation starting from racemic **32** (Scheme 3).

**Scheme 4:**
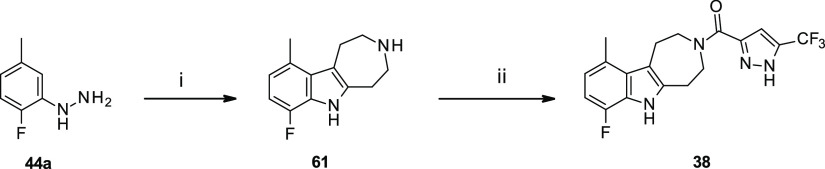
Synthesis of 7-Fluoro-10-methyl-2,4,5,6-tetrahydro-1*H*-azepino[4,5-*b*]indol-3-yl Amide **38** Reagents and reaction conditions: (i) *tert*-butyl 4-oxo-azepane-1-carboxylate (**60**), HCl (36%), EtOH, 80 °C, and 16 h; (ii) 5-methyl-1*H*-pyrazole-3-carboxylic acid, HATU, DIPEA, dry DMF, 0 °C to r.t., 16 h, and 7%.

Compound **38**, featuring a substituted tetrahydroazepino-indole scaffold, was synthesized starting from Boc-protected azepan-4-one **60** with an analogous approach,^48^ as seen for the previously described tetrahydropyridine-indole analogues, though slightly forcing the reaction conditions (Scheme 4). Probably because of conformational constraints, Fischer indole synthesis of hydrazine **44a** with ketone **60** led only to hexahydroazepino-indole **61** with complete regioselectivity. The intermediate **61** underwent classical amide coupling with 5-trifluoromethyl-1*H*-pyrazole-3-carboxylic acid (**C**) to obtain final compound **38**.

## Conclusions

CF, the most frequent autosomal recessive disease, is a multiorgan disease, primarily affecting the lungs. CF mutations cause CFTR protein dysfunction by multiple mechanisms, affecting its expression, stability, or its function as an anion channel. CFTR modulators have been reported to address the basic defects caused by CF mutations restoring, at least partially, the CFTR function. In particular, small-molecule compounds, called potentiators, are known to ameliorate the gating defect.

In the present work, we describe the identification of a novel chemotype of CFTR potentiators. The screening of a library of compounds provided a few hits featuring a common 2,3,4,5-tetrahydro-1*H*-pyrido[4,3-*b*]indole core, which were able to rescue the activity of F508del- and G551D-CFTR in an effective manner.

1,2,3,4-Tetrahydro-γ-carbolines represent a chemical class of well-studied heterocycles, extensively characterized for their chemical and biological properties; therefore, as reported by Ivashchenko,^49^ such tricyclic compounds could be regarded as typical “privileged structures”.^50^ The sustained interest in this scaffold is due to the fact that tetrahydro-γ-carbolines and their derivatives have shown a broad spectrum of biological activities,^49,51−53^ primarily for the treatment of central nervous system diseases.^36,54−56^ Although in the last few years substituted derivatives of tetrahydro-γ-carbolines have been reported to be active toward different biological targets,^52^ to the best of our knowledge, this type of heterocyclic small molecules has never been described as pharmacologically active compounds for the treatment of CF or related conditions.

The initial hits were validated and further explored in SAR studies, leading to the discovery of novel potentiators active in the mid-to-low nanomolar range. Among them, the enantiomerically pure compound **39** turned out to be quite promising being able to rescue the gating defect of both F508del-CFTR (in FRT and CFBE41o-cells) and G551D-CFTR (in FRT cells) with good potency and efficacy, similarly to VX-770. Notably, the tetrahydro-γ-carboline **39** did not affect the rescue efficacy of correctors VX-809 or **ARN23765** in immortalized bronchial CFBE41o-cells and in primary HBE cells from an F508del/F508del CF patient and increased by more than 2-fold the fraction of CFTR-mediated chloride current activated by submaximal cAMP stimulation in HBE cells from non-CF individuals.

Furthermore, potentiator **39** showed good *in vitro* druglike properties and was therefore evaluated *in vivo* for its pharmacokinetic profile in rats. Following oral administration, significant exposure levels were obtained, leading to good oral bioavailability. A subsequent study showed that potentiator **39** distributed to the lung after oral administration to rats, with compound levels also detectable at 4 h postdosing.

To conclude, this study allowed the identification of *N*^2^-acyl-substituted 2,3,4,5-tetrahydro-1*H*-pyrido[4,3-*b*]indoles as novel CFTR potentiators endowed with a good efficacy in rescuing the gating defect of F508del- and G551D-CFTR and a preliminary promising druglike profile. These findings represent a promising starting point to further improve and develop this chemical class, adding a new chemotype to the existing classes of CFTR potentiators, possibly expanding the current portfolio of therapeutic solutions for the treatment of CF.

## Experimental Section

### Chemistry

#### Synthetic Materials and Methods

Solvents and reagents were obtained from commercial suppliers and were used without further purification. Automated column chromatography purifications were performed on a Teledyne ISCO apparatus (CombiFlash Rf) with prepacked silica gel columns of different sizes (RediSep). NMR experiments were run at 300 K on a Bruker AVANCE III 400 system (400.13 MHz for ^1^H and 100.62 MHz for ^13^C), equipped with a BBI probe and *Z*-gradients, and Bruker FT NMR AVANCE III 600 MHz spectrometer equipped with a 5 mm CryoProbe QCI ^1^H/^19^F–^13^C/^15^N–D quadruple resonance, a shielded *Z*-gradient coil and the automatic sample changer SampleJet NMR system (600 MHz for ^1^H, 151 MHz for ^13^C, and 565 MHz for ^19^F). Chemical shifts for ^1^H and ^13^C spectra were reported in parts per million (ppm), calibrating the residual nondeuterated solvent peak for ^1^H and ^13^C, respectively, to 7.26 and 77.16 ppm for CDCl_3_ and 2.50 and 39.52 ppm for DMSO-*d*_6_, whereas spectra in D_2_O were referred to trimethylsilylpropanoic acid peak set at 0.00 ppm. Ultra performance liquid chromatography–mass spectrometry (UPLC/MS) analyses were performed on a Waters ACQUITY UPLC/MS system consisting of a single quadrupole detector (SQD) mass spectrometer equipped with an electrospray ionization interface and a photodiode array detector. Electrospray ionization in positive and negative mode was applied in the mass scan range 100–500 Da. The PDA range was 210–400 nm. The mobile phase was 10 mM NH_4_OAc in H_2_O at pH 5 adjusted with AcOH (A) and 10 mM NH_4_OAc in CH_3_CN–H_2_O (95:5) at pH 5 (B) with 0.5 mL/min as the flow rate. For intermediates, the analyses were run on an ACQUITY UPLC BEH C_18_ column (100 × 2.1 mm ID, particle size 1.7 μm) with a VanGuard BEH C_18_ precolumn (5 × 2.1 mm ID, particle size 1.7 μm). A linear gradient was applied: 0–0.2 min: 5% B; 0.2–2.2 min: 5–95% B; 2.2–2.3 min: 95–100% B; and 2.3–3.0 min: 100% B. For final compounds, a 10 mM DMSO stock solution of test compound was prepared in DMSO-*d*_6_ and further diluted 20-fold in CH_3_CN–H_2_O (1:1) for analysis. The analyses were run on an ACQUITY UPLC BEH C_18_ column (100 × 2.1 mm ID, particle size 1.7 μm) with a VanGuard BEH C_18_ precolumn (5 × 2.1 mm ID, particle size 1.7 μm). A linear gradient was applied: 0–0.2 min: 10% B; 0.2–6.2 min: 10–90% B; 6.2–6.3 min: 90–100% B; and 6.3–7.0 min: 100% B. The purifications by high-performance liquid chromatography–mass spectrometry (HPLC/MS) were performed on a Waters AutoPurification system consisting of a 3100 Single Quadrupole mass spectrometer equipped with an electrospray ionization interface and a 2998 Photodiode Array Detector. The HPLC system included a 2747 Sample Manager, a 2545 Binary Gradient Module, a system fluidic organizer, and a 515 HPLC pump. Electrospray ionization in positive and negative modes was performed in the mass scan range of 100–500 Da. The PDA range was 210–400 nm. The purifications were run on a XBridge Prep C_18_ OBD column (100 × 19 mm ID, particle size 5 μm) with a XBridge Prep C_18_ (10 × 19 mm ID, particle size 5 μm) Guard cartridge with a flow rate of 20 mL/min. The analytical chiral separations were performed on a Waters Alliance HPLC instrument consisting of an e2695 Separation Module and a 2998 Photodiode Array Detector. The PDA range was 210–400 nm. The analyses were run in isocratic mode on Daicel ChiralPak AD column (250 × 4.6 mm ID, particle size 10 μm) with a flow rate of 1.0 mL/min. The semipreparative chiral separations were performed on a Waters Alliance HPLC instrument consisting of a 1525 Binary HPLC Pump, a Waters Fraction Collector III, and a 2998 Photodiode Array Detector. The separations were run in the isocratic mode on a Daicel ChiralPak AD column (250 × 10 mm ID, particle size 10 μm) with a ChiralPak AD Semi-Prep. Guard precolumn (50 × 10 mm ID, particle size 10 μm) at room temperature (r.t.), with a flow rate of 5.0 mL/min. High-resolution mass spectrometry (HRMS) measurements were performed on a Waters SYNAPT G2 Q-ToF mass spectrometer equipped with an electrospray ionization interface and coupled to a Waters ACQUITY UPLC. Leucine enkephalin (2 ng/mL) was used as the lock mass reference compound for spectral recalibration. The analyses were run on an ACQUITY UPLC BEH C_18_ column (100 × 2.1 mm ID, particle size 1.7 μm) with a VanGuard BEH C_18_ precolumn (5 × 2.1 mm ID, particle size 1.7 μm). The mobile phase was H_2_O + 0.1% HCOOH (A) and CH_3_CN + 0.1% HCOOH (B) with 0.5 mL/min as the flow rate. A linear gradient was applied: 0–0.2 min: 10% B; 0.2–6.2 min: 10–90% B; 6.2–6.3 min: 90–100% B; and 6.3–7.0 min: 100% B. The synthesis and characterization of all final compounds **1–42** is reported below. Purity of the initial hits (Hit 1–Hit 6) and final compounds was determined by UPLC/MS and quantitative ^1^H NMR (qNMR, see the Supporting Information) and was equal to or greater than 95% for all of the compounds, except for Hit-6 (80% purity) and analogue **15** (93% purity).

### Synthesis of Phenylhydrazine Hydrochlorides **44a,b**

#### (2-Fluoro-5-methyl-phenyl)hydrazine Hydrochloride (**44a**)

2-Fluoro-5-methyl-aniline (**43a**) (1.0 g, 8.0 mmol, 1.0 equiv) was added at 0 °C to a vigorously stirred aqueous solution of HCl 36% (3.0 mL). To the thus-obtained suspension, a solution of sodium nitrite (1.0 g, 15.2 mmol, 1.9 equiv) and a solution of tin chloride (4.3 g, 19.2 mmol, 2.4 equiv) in HCl 6 M (7.7 mL) were added. The reaction mixture was stirred at r.t. for 24 h and basified with NaOH 12 M. The aqueous phase was extracted with Et_2_O (3 × 20 mL), and the combined organic extracts were dried over Na_2_SO_4_ and filtered. To obtain the hydrazine hydrochloride, a saturated solution of HCl in Et_2_O was added. The salt was filtered and washed with Et_2_O, obtaining the title compound as a white solid (0.6 g, 45%). ^1^H NMR (400 MHz, DMSO-*d*_6_): δ 10.31 (s, 3H), 8.22 (s, 1H), 7.11 (dd, *J* = 11.7, 8.2 Hz, 1H), 7.04 (dd, *J* = 8.3, 2.0 Hz, 1H), 6.77 (dddd, *J* = 8.3, 4.6, 2.1, 0.8 Hz, 1H), 2.25 (s, 3H).

#### (2-Fluoro-4-methoxy-phenyl)hydrazine Hydrochloride (**44b**)

The title compound was afforded following the protocol reported for the synthesis of **44a**, starting from 2-fluoro-3-methoxy-aniline (**43b**) (1.0 g, 7.1 mmol, 1.0 equiv), aq HCl 36% (2.6 mL), sodium nitrite (0.93 g, 13.5 mmol, 1.9 equiv), and tin chloride (3.84 g, 17.0 mmol, 2.4 equiv) in HCl 6 M (6.8 mL). Treatment with HCl in Et_2_O afforded the title compound as a white solid (0.7 g, 51%). ^1^H NMR (400 MHz, DMSO-*d*_6_): δ 10.02 (br s, 3H), 7.79 (s, 1H), 7.22 (dd, *J* = 9.8, 8.9 Hz, 1H), 6.88 (dd, *J* = 13.1, 2.7 Hz, 1H), 6.77 (ddd, *J* = 8.9, 2.8, 1.2 Hz, 1H); UPLC-MS: *t*_R_ = 1.42 min; MS (ESI) *m*/*z*: calcd for C_7_H_10_FN_2_O (M + H)^+^, 157.1; found, 157.3.

Substituted phenylhydrazine hydrochlorides (**44c–o**) were commercially available and used as such without further purification.

### Synthesis of Tetrahydro-γ-carbolines **45a–o**, **48**, **52–55**, **57**, and **59**

#### General Procedures **1a** (Gp1a)

HCl 36% (11 equiv) was added to a solution 0.3 M of suitable hydrazine of type **I** (1.0 equiv) and ketone of type **II** (1.0 equiv) in EtOH. The reaction mixture was heated at 80 °C and stirred for 16 h. The suspension was filtered and washed with Et_2_O to furnish the title compound of type **III**.

#### General Procedure **1b** (Gp1b)

A solution of hydrazine of type **I** (1.0 equiv) and ketone of type **II** (1.0 equiv) in EtOH (0.5 M) was stirred at r.t., until the formation of hydrazone intermediate. 2,4,6-Trichloro-1,3,5-triazine (0.4 equiv) was added and the reaction mixture was heated to 90 °C for 8 h. The reaction mixture was cooled to r.t. and the obtained precipitate was filtered and washed with cold EtOH. The crude product of type **III** was used as such in the next step without further purification.

#### General Procedure **1c** (Gp1c)

Hydrazine of type **I** (1.0 equiv) and ketone of type **II** (1.0 equiv) were dissolved in EtOH (0.2 M) and the reaction mixture was stirred at r.t. for 30 min, until the complete formation of hydrazone. The solvent was removed under reduced pressure and the crude mixture was dissolved in AcOH (0.1 M), followed by addition of trifluoroborate diethyletherate (2.0 equiv). The reaction was stirred at 90 °C for 16 h. The solvent was removed and the crude mixture was poured into an aq 2.0 M NaOH solution and extracted with dichloromethane (DCM), dried over Na_2_SO_4_, filtered, and concentrated *in vacuo* to afford the crude product of type **III**, which was used in the next step without any further purification.

#### General Procedure **1d** (Gp1d)

A suspension of hydrazine of type **I** (1.0 equiv) and ketone of type **II** (1.0 equiv) in toluene (0.2 M) was stirred at 50 °C until the formation of hydrazone intermediate. *p*-Toluenesulfonic acid (1.5 equiv) was added and the reaction mixture was heated to 120 °C for 24 h. The reaction mixture was cooled to r.t. and sat. aq Na_2_CO_3_ solution was added until pH 8. The crude was extracted with DCM, dried over Na_2_SO_4_, filtered, and concentrated *under vacuo*. The compound of type **III** was isolated by chromatography on alumina using DCM/MeOH·NH_3_ (1.0 N) as an eluent.

#### 6-Fluoro-9-methyl-2,3,4,5-tetrahydro-1*H*-pyrido[4,3-*b*]indole Hydrochloride (**45a**)

Following Gp1b, the title compound was obtained from (2-fluoro-5-methyl-phenyl)hydrazine hydrochloride (**44a**) (0.86 g, 4.90 mmol) and *tert*-butyl 4-oxopiperidine-1-carboxylate (0.98 g, 4.90 mmol) as a light orange solid (0.65 g, 95%). UPLC-MS: *t*_R_ = 1.31 min; MS (ESI) *m*/*z*: calcd for C_12_H_14_FN_2_ (M + H)^+^, 205.1; found, 205.8.

#### 6-Fluoro-8-methoxy-2,3,4,5-tetrahydro-1*H*-pyrido[4,3-*b*]indole Hydrochloride (**45b**)

Following Gp1a, the title compound was obtained from (2-fluoro-4-methoxy-phenyl)hydrazine hydrochloride (**44b**) (0.48 g, 2.50 mmol) and *tert*-butyl 4-oxopiperidine-1-carboxylate (0.49 g, 2.50 mmol) in a mixture with trace amounts of 6-chloro-8-methoxy-2,3,4,5-tetrahydro-1*H*-pyrido[4,3-*b*]indole, as a by-product. The crude product was used as such in the next step without further purification. UPLC-MS: *t*_R1_ = 1.39 min; MS (ESI) *m*/*z*: calcd for C_12_H_13_ClN_2_O (M + H)^+^, 237.1; found, 237.4; *t*_R2_ = 1.52 min; MS (ESI) *m*/*z*: calcd for C_12_H_14_FN_2_O (M + H)^+^, 221.1; found, 221.8.

#### 6-Methyl-2,3,4,5-tetrahydro-1*H*-pyrido[4,3-*b*]indole Hydrochloride (**45c**)

Following Gp1a, the title compound was obtained from *o*-tolylhydrazine (**44c**) (0.14 g, 0.86 mmol) and *tert*-butyl 4-oxopiperidine-1-carboxylate (0.17 g, 0.86 mmol) as a white solid (0.07 g, 42%). UPLC-MS: *t*_R_ = 1.31 min; MS (ESI) *m*/*z*: calcd for C_12_H_15_N_2_ (M + H)^+^, 187.1; found, 187.2.

#### 7-Methyl-2,3,4,5-tetrahydro-1*H*-pyrido[4,3-*b*]indole (**45d′**) and 9-Methyl-2,3,4,5-tetrahydro-1*H*-pyrido[4,3-*b*]indole (**45d″**)

Following Gp1a, a mixture of the title compounds was obtained from (3-methyl)phenylhydrazine hydrochloride (**44d**) (1.5 g, 9.46 mmol) and *tert*-butyl 4-oxopiperidine-1-carboxylate (1.88 g, 9.46 mmol) as a white solid (1.04 g, 50% combined). UPLC-MS: *t*_R1_ = 1.27 min, *t*_R2_ = 1.31 min; MS (ESI) *m*/*z*: calcd for C_12_H_15_N_2_ (M + H)^+^, 187.1; found, 187.1.

#### 8-Isopropyl-2,3,4,5-tetrahydro-1*H*-pyrido[4,3-*b*]indole Hydrochloride (**45e**)

Following Gp1a, the title compound was obtained from (4-isopropylphenyl)hydrazine (**44e**) (0.21 g, 1.1 mmol) and *tert*-butyl 4-oxopiperidine-1-carboxylate (0.22 g, 1.1 mmol) as a white solid (0.10 g, 42%). UPLC-MS: *t*_R_ = 1.67 min; MS (ESI) *m*/*z*: calcd for C_14_H_19_N_2_ (M + H)^+^, 215.1; found, 215.2.

#### 6-Fluoro-2,3,4,5-tetrahydro-1*H*-pyrido[4,3-*b*]indole (**45f**)

Following Gp1a, the title compound was obtained from (2-fluorophenyl)hydrazine hydrochloride (**44f**) (0.68 g, 4.2 mmol) and *tert*-butyl 4-oxopiperidine-1-carboxylate (0.84 g, 4.2 mmol), after purification by trituration with chilled ethanol, as the solvent. The solid was partitioned between EtOAc (50 mL) and sat. aq NaHCO_3_ sol. (50 mL). The organic phase was separated, washed with brine (50 mL), dried over anhydrous Na_2_SO_4_, and filtered and the solvent evaporated under reduced pressure. The crude residue was purified by silica gel flash column chromatography, with DCM/MeOH/Et_3_N (9:1:0.1) as the eluent, to afford the title compound as an off-white solid (0.04 g, 5%). ^1^H NMR (400 MHz, DMSO-*d*_6_): δ 11.25 (s, 1H), 7.16 (d, *J* = 7.7 Hz, 1H), 6.96–6.77 (m, 1H), 3.94 (s, 2H), 3.11 (t, *J* = 5.8 Hz, 2H), 2.75 (t, *J* = 5.8 Hz, 2H); UPLC-MS: *t*_R_ = 1.25 min; MS (ESI) *m*/*z*: calcd for C_11_H_12_FN_2_ (M + H)^+^, 191.1; found, 191.1.

#### 8-(Trifluoromethyl)-2,3,4,5-tetrahydro-1*H*-pyrido[4,3-*b*]indole (**45g**)

Following GP1c, the title compound was obtained from (4-trifluoromethylphenyl)hydrazine (**44g**) (0.5 g, 2.35 mmol) and 4-oxopiperidine-1-carboxylate (0.24 g, 2.35 mmol) as a white solid (0.11 g, 20%). ^1^H NMR (400 MHz, DMSO-*d*_6_): δ 7.68 (s, 1H), 7.44 (d, *J* = 8.4 Hz, 1H), 7.27 (d, *J* = 8.6 Hz, 1H), 3.88 (d, *J* = 4.9 Hz, 2H), 3.02 (q, *J* = 5.5 Hz, 2H), 2.70 (t, *J* = 5.7 Hz, 2H). UPLC-MS: *t*_R_ = 1.59 min; MS (ESI) *m*/*z*: calcd for C_12_H_12_F_3_N_2_ (M + H)^+^, 241.1; found, 242.1.

#### 8-(Trifluoromethoxy)-2,3,4,5-tetrahydro-1*H*-pyrido[4,3-*b*]indole (**45h**)

Following Gp1a, the title compound was obtained from (4-trifluoromethoxy)hydrazine hydrochloride (**44h**) (0.67 g, 2.92 mmol) and *tert*-butyl 4-oxopiperidine-1-carboxylate (0.58 g, 2.92 mmol), after purification by trituration with chilled ethanol, as the solvent. The solid was partitioned between EtOAc (50 mL) and sat. aq NaHCO_3_ sol. (50 mL). The organic phase was separated, washed with brine (50 mL), dried over anhydrous Na_2_SO_4_, and filtered, and the solvent was evaporated under reduced pressure. The crude residue was purified by silica gel flash column chromatography, with DCM/MeOH (9:1) as the eluent, to afford the title compound as a yellow solid (0.6 g, 8%). ^1^H NMR (400 MHz, DMSO-*d*_6_): δ 11.11 (s, 1H), 7.41–7.23 (m, 2H), 6.97 (dd, *J* = 8.9, 2.3 Hz, 1H), 3.94 (s, 2H), 3.12 (t, *J* = 5.8 Hz, 2H), 2.76 (t, *J* = 5.8 Hz, 2H); UPLC-MS: *t*_R_ = 1.64 min; MS (ESI) *m*/*z*: calcd for C_12_H_12_F_3_N_2_O (M + H)^+^, 257.1; found, 257.2.

#### 2,3,4,5-Tetrahydro-1*H*-pyrido[4,3-*b*]indole-8-carbonitrile (**45j**)

Following GP1c, the title compound was obtained from 4-hydrazinobenzonitrile hydrochloride (**44j**) (0.07 g, 0.31 mmol) and piperidin-4-one hydrochloride (0.042 g, 0.31 mmol), after purification by trituration with chilled ethanol as the solvent, as a yellow solid (0.045 g, 73%). UPLC-MS: *t*_R_ = 1.10 min; MS (ESI) *m*/*z*: calcd for C_12_H_12_N_3_ (M + H)^+^, 198.1; found, 198.1.

#### 8-Methylsulfonyl-2,3,4,5-tetrahydro-1*H*-pyrido[4,3-*b*]indole (**45k**)

Following Gp1c, the title compound was obtained from (4-methylsulfonylphenyl)hydrazine hydrochloride (**44k**) (0.90, 0.4 mmol) and piperidin-4-one hydrochloride (0.055 g, 0.4 mmol), after purification by trituration with DCM, as the solvent, as a yellow solid (0.1 g, quant.). UPLC-MS: *t*_R_ = 1.08 min; MS (ESI) *m*/*z*: calcd for C_12_H_15_N_2_O_2_S (M + H)^+^, 251.1; found, 251.1.

#### 6-Fluoro-8-methyl-2,3,4,5-tetrahydro-1*H*-pyrido [4,3-*b*]indole Hydrochloride (**45i**)

Following Gp1a, the title compound was obtained from (2-fluoro-4-methyl-phenyl)hydrazine hydrochloride (**44i**) (0.23 g, 1.32 mmol) and *tert*-butyl 4-oxopiperidine-1-carboxylate (0.26 g, 1.32 mmol) as a brown solid (0.17 g, 63%). ^1^H NMR (400 MHz, DMSO-*d*_6_): δ 11.46 (s, 1H), 7.08 (s, 1H), 6.79 (d, *J* = 12.4 Hz, 1H), 4.27 (s, 2H), 3.47 (t, *J* = 6.1 Hz, 2H), 3.00 (t, *J* = 6.0 Hz, 2H), 2.37 (s, 3H). UPLC-MS: *t*_R_ = 1.44 min; MS (ESI) *m*/*z*: calcd for C_12_H_14_FN_2_ (M + H)^+^, 205.1; found, 205.2.

#### 6,8-Difluoro-2,3,4,5-tetrahydro-1*H*-pyrido[4,3-*b*]indole Hydrochloride (**45l**)

Following Gp1a, the title compound was obtained from (2,4-difluoro-phenyl)hydrazine hydrochloride (**44l**) (0.26 g, 1.41 mmol) and *tert*-butyl 4-oxopiperidine-1-carboxylate (0.28 g, 1.41 mmol) as a brown solid (0.15 g, 51%). ^1^H NMR (400 MHz, DMSO-*d*_6_): δ 11.76 (s, 1H), 9.28 (s, 2H), 7.19 (dd, *J* = 9.4, 2.2 Hz, 1H), 6.98 (ddd, *J* = 11.7, 9.8, 2.3 Hz, 1H), 4.26 (s, 2H), 3.46 (t, *J* = 6.1 Hz, 2H), 3.03 (t, *J* = 6.1 Hz, 2H). UPLC-MS: *t*_R_ = 1.35 min; MS (ESI) *m*/*z*: calcd for C_11_H_11_F_2_N_2_ (M + H)^+^, 209.1; found, 209.3.

#### 6-Fluoro-8-(trifluoromethyl)-2,3,4,5-tetrahydro-1*H*-pyrido[4,3-*b*]indole Hydrochloride (**45m**)

Following Gp1a, the title compound was obtained from (2-fluoro-4-trifluoromethylphenyl)hydrazine (**44m**) (0.56 g, 2.42 mmol) and *tert*-butyl 4-oxopiperidine-1-carboxylate (0.48 g, 2.42 mmol) as a brown solid (0.2 g, 32%). UPLC-MS: *t*_R_ = 1.41 min; MS (ESI) *m*/*z*: calcd for C_12_H_11_F_4_N_2_ (M + H)^+^, 259.1; found, 259.1.

#### 6,9-Difluoro-2,3,4,5-tetrahydro-1*H*-pyrido[4,3-*b*]indole Hydrochloride (**45n**)

Following Gp1a, the title compound was obtained from (2,5-difluorophenyl)hydrazine hydrochloride (**44n**) (0.55 g, 3.06 mmol) and *tert*-butyl 4-oxopiperidine-1-carboxylate (0.61 g, 3.06 mmol) as a brown solid (0.14 g, 22%). UPLC-MS: *t*_R_ = 1.69 min; MS (ESI) *m*/*z*: calcd for C_11_H_11_F_2_N_2_ (M + H)^+^, 209.1; found, 209.5.

#### 9-Fluoro-6-methyl-2,3,4,5-tetrahydro-1*H*-pyrido [4,3-*b*]indole Hydrochloride (**45o**)

Following Gp1a, the title compound was obtained from (5-fluoro-2-methyl-phenyl)hydrazine (**44o**) (0.48 g, 2.64 mmol) and *tert*-butyl 4-oxopiperidine-1-carboxylate (0.52 g, 2.64 mmol) as a brown solid (0.14 g, 26%). ^1^H NMR (400 MHz, DMSO-*d*_6_): δ 11.35 (s, 1H), 6.87–6.80 (m, 1H), 6.68 (dd, *J* = 10.8, 7.9 Hz, 1H), 4.38 (s, 2H), 3.46 (t, *J* = 6.1 Hz, 2H), 3.03 (t, *J* = 6.2 Hz, 2H), 2.40 (s, 3H). UPLC-MS: *t*_R_ = 1.44 min; MS (ESI) *m*/*z*: calcd for C_12_H_14_FN_2_ (M + H)^+^, 205.1; found, 205.3.

2,3,4,5-Tetrahydro-1*H*-pyrido[4,3-*b*]indole (**45p**), 8-methoxy-2,3,4,5-tetrahydro-1*H*-pyrido[4,3-*b*]indole (**45q**), 8-methyl-2,3,4,5-tetrahydro-1*H*-pyrido[4,3-*b*]indole (**45r**), and 8-fluoro-2,3,4,5-tetrahydro-1*H*-pyrido[4,3-*b*]indole (**45s**) were commercially available and used as such without further purification.

#### (*rac*)-6-Fluoro-1,9-dimethyl-2,3,4,5-tetrahydro-1*H*-pyrido[4,3-*b*]indole (**52**)

Following Gp1d, starting from (2-fluoro-5-methyl-phenyl)hydrazine hydrochloride (**44a**) (1.35 g, 7.64 mmol) and racemic *tert*-butyl 2-methyl-4-oxo-piperidine-1-carboxylate (**49**) (1.5 g, 7.64 mmol), a crude mixture of regioisomers **52**/**53** in a 20:80 ratio (by ^1^H NMR) was afforded. Purification by neutral alumina column chromatography with DCM/MeOH/NH_3_ (95:5:0.1) afforded the title compound as a pure regioisomer as a brown resin (0.1 g, 6%). ^1^H NMR (400 MHz, DMSO-*d*_6_): δ 11.06 (br s), 6.66 (dd, *J* = 11.1, 7.6 Hz, 1H), 6.58–6.61 (m, 1H), 4.39 (q, *J* = 6.5 Hz, 1H), 3.10 (ddd, *J* = 13.1, 10.2, 4.9 Hz, 1H), 2.97 (ddd, *J* = 12.8, 6.1, 2.2 Hz, 1H), 2.66 (m, 1H), 2.56 (ddd, *J* = 16.0, 4.8, 2.0 Hz, 1H), 2.50 (s, 3H), 1.37 (d, *J* = 6.5 Hz, 3H). UPLC-MS: *t*_R_ = 1.46 min; MS (ESI) *m*/*z*: calcd for C_19_H_17_FN_2_O (M + H)^+^, 218.27; found, 219.4.

#### (*rac*)-6-Fluoro-3,9-dimethyl-2,3,4,5-tetrahydro-1*H*-pyrido[4,3-*b*]indole (**53**)

Following Gp1d, starting from (2-fluoro-5-methyl-phenyl)hydrazine hydrochloride (**44a**) (1.35 g, 7.64 mmol) and racemic *tert*-butyl 2-methyl-4-oxo-piperidine-1-carboxylate (**49**) (1.5 g, 7.64 mmol), a crude mixture of regioisomers **52**/**53** in a 20:80 ratio (by ^1^H NMR) was afforded. Purification by neutral alumina column chromatography with DCM/MeOH/NH_3_ (95:5:0.1) afforded the title compound as a pure regioisomer as a brown resin (0.48 g, 29%). ^1^H NMR (400 MHz, DMSO-*d*_6_): δ 11.03 (br s), 6.64 (dd, *J* = 11.3, 7.6 Hz, 1H), 6.57 (ddd, *J* = 7.9, 4.8, 0.7 Hz, 1H), 4.17 (dd, *J* = 14.8, 1.1 Hz, 1H), 4.08 (*app*-dt, *J* = 14.8, 1.8, 1.8 Hz, 1H), 2.89 (m, 1H), 2.67 (ddd, *J* = 17.5, 3.5, 1.5 Hz, 1H), 2.34 (m, 1H), 2.44 (s, 3H), 1.18 (d, *J* = 6.3 Hz, 3H). UPLC-MS: *t*_R_ = 1.46 min; MS (ESI) *m*/*z*: calcd for C_19_H_17_FN_2_O (M + H)^+^, 218.27; found, 219.4.

#### (*R*)-6-Fluoro-3,9-dimethyl-2,3,4,5-tetrahydro-1*H*-pyrido[4,3-*b*]indole (**54**)

Following Gp1d, starting from (2-fluoro-5-methyl-phenyl)hydrazine hydrochloride (**44a**) and enantiomerically pure (*R*)-*tert*-butyl 2-methyl-4-oxo-piperidine-1-carboxylate (**50**), a crude mixture of regioisomers **52**^a^/**54** in a ca. 20:80 ratio (by ^1^H NMR) was afforded. Purification by neutral alumina column chromatography with DCM/MeOH/NH_3_ (95:5:0.1) afforded the title compound as a pure regioisomer as a brown resin (0.8 g, 30%).

#### (*S*)-6-Fluoro-3,9-dimethyl-2,3,4,5-tetrahydro-1*H*-pyrido[4,3-*b*]indole (**55**)

Following Gp1d, starting from (2-fluoro-5-methyl-phenyl)hydrazine hydrochloride (**44a**) and enantiomerically pure (*S*)-*tert*-butyl 2-methyl-4-oxo-piperidine-1-carboxylate (**51**), a crude mixture of regioisomers **52**^b^/**55** in a ca. 20:80 ratio (by ^1^H NMR) was afforded. Purification by neutral alumina column chromatography with DCM/MeOH/NH_3_ (95:5:0.1) afforded the title compound as a pure regioisomer as a brown resin (0.8 g, 30%).

#### 6-Fluoro-3,3,9-trimethyl-2,3,4,5-tetrahydro-1*H*-pyrido[4,3-*b*]indole (**57**)

Following GP1d, starting from (2-fluoro-5-methyl-phenyl)hydrazine hydrochloride (**44a**) (0.3 g, 1.72 mmol) and *tert*-butyl 2,2-dimethyl-4-oxo-piperidine-1-carboxylate (**56**) (0.34 g, 1.72 mmol), the title compound was obtained as a pure regioisomer, after purification by neutral alumina chromatography with DCM/MeOH/NH_3_ (95:5:0.1), as a brown solid (0.06 g, 15%). ^1^H NMR (400 MHz, CDCl_3_): δ 8.28 (br s, 1H), 6.62–6.72 (m, 2H), 4.28 (s, 2H), 2.52 (s, 2H), 2.35 (s, 3H), 1.42 (s, 6H). UPLC-MS: *t*_R_ = 1.46 min; MS (ESI) *m*/*z*: calcd for C_14_H_17_FN_2_ (M + H)^+^, 233.3; found, 233.5.

#### 4-Fluoro-1-methyl-5,6,7,8,9,10-hexahydro-7,10-epiminocyclohepta[*b*]indole Hydrochloride (**59**)

Following Gp1a, the title compound was obtained from (2-fluoro-5-methyl-phenyl)hydrazine hydrochloride (**44a**) (0.43 g, 2.47 mmol) and *tert*-butyl 3-oxo-8-azabicyclo[3.2.1]octane-8-carboxylate (**58**) (0.53 g, 2.47) as a white solid (0.21 g, 37%). UPLC-MS: *t*_R_ = 1.56 min; MS (ESI) *m*/*z*: calcd for C_14_H_16_FN_2_ (M + H)^+^, 231.1; found, 231.3.

#### 8-Methoxy-5-methyl-2,3,4,5-tetrahydro-1*H*-pyrido[4,3-*b*]indole (**48**)

##### Step 1: *tert*-Butyl 8-Methoxy-1,3,4,5-tetrahydro-2*H*-pyrido[4,3-*b*]indole-2-carboxylate (**46**)

To a cooled suspension of 8-methoxy-2,3,4,5-tetrahydro-1*H*-pyrido[4,3-*b*]indole (**45q**) (0.25 g, 1.24 mmol) in dry DCM (7.0 ml) at 0 °C were added *N*,*N*-diisopropylethylamine (DIPEA) (0.04 ml) and di-*tert*-butyl dicarbonate (0.28 g, 1.30 mmol). The resulting white mixture was stirred at r.t. for 1 h under the nitrogen atmosphere. Sat. aq NH_4_Cl solution (3 mL) was added, and the resulting aqueous phase was extracted twice with DCM (2 × 3 mL). The collected organic layers were dried over Na_2_SO_4_, filtered, and concentrated under vacuum to afford the title compound (0.33 g, 88%), which was used in the next step without any further purification. ^1^H NMR (400 MHz, DMSO-*d*_6_): δ 10.69 (1H, NH), 7.17 (d, *J* = 8.7 Hz, 1H), 6.88 (br s, 1H), 6.66 (dd, *J* = 8.7, 2.4 Hz, 1H), 4.49 (br s, 2H), 3.74 (s, 3H), 3.69 (t, *J* = 5.6 Hz, 2H), 2.74 (t, *J* = 5.6 Hz, 2H), 1.44 (s, 9H). UPLC-MS: *t*_R_ = 2.28 min; MS (ESI) *m*/*z*: calcd for C_17_H_21_N_2_O_3_ (M – H)^−^*m*/*z*: 301.3; found, 301.1 (M – H)^−^.

##### Step 2: *tert*-Butyl 8-Methoxy-5-methyl-1,3,4,5-tetrahydro-2*H*-pyrido[4,3-*b*]indole-2-carboxylate (**47**)

In a flame-dried Schlenk reactor, under the nitrogen atmosphere, sodium hydride (60% dispersion in mineral oil) (0.025 g, 0.621 mmol) was suspended in dry dimethylformamide (DMF) (3.0 mL). The mixture was cooled to 0 °C and intermediate **46** (0.15 g, 0.50 mmol) was added followed, after 30 min, by dropwise addition of iodomethane (0.038 mL, 0.61 mmol). The mixture turned yellow and was stirred at r.t. for 16 h, and then cooled water (5 mL) was added dropwise to the crude mixture. A white precipitate was formed, filtered, washed with water, and dried under high vacuum to afford the pure title compound (0.144 g, 91%). ^1^H NMR (400 MHz, DMSO-*d*_6_): δ 7.29 (d, *J* = 8.7 Hz, 1H), 6.93 (br s, 1H), 6.74 (d, *J* = 8.7 Hz, 1H), 4.50 (br s, 2H), 3.75 (s, 3H), 3.71 (t, *J* = 5.8 Hz, 2H), 3.58 (s, 3H), 2.77 (t, *J* = 5.8 Hz, 2H), 1.44 (s, 9H). UPLC-MS: *t*_R_ = 2.49 min; MS (ESI) *m*/*z*: calcd for C_18_H_25_N_2_O_3_ (M + H)^+^, 317.4; found, 317.0 (M + H)^+^.

##### Step 3: 8-Methoxy-5-methyl-2,3,4,5-tetrahydro-1*H*-pyrido[4,3-*b*]indole (**48**)

To a solution of **47** (0.12 g, 0.39 mmol) in dry DCM (3.0 mL), HCl (4.0 M) in dioxane (1.16 mL, 4.66 mmol) was added dropwise at r.t. During addition, the mixture turned dark. After being stirred at r.t. for 20 h, the solvents were removed under vacuum and the residue was treated with water (2 mL) and NaOH until pH 12 and extracted with DCM (3 × 3 mL). The organic extracts were dried over Na_2_SO_4_ and the solvent was evaporated *in vacuo*, affording the title compound (0.083 g, 81%). ^1^H NMR (400 MHz, DMSO-*d*_6_): δ 7.24 (d, *J* = 8.7 Hz, 1H), 6.83 (d, *J* = 2.4 Hz, 1H), 6.69 (dd, *J* = 8.9, 2.5 Hz, 1H), 3.80 (br s, 2H), 3.73 (s, 3H), 3.56 (s, 3H), 3.51 (s, 1H, NH), 3.28 (br s, 2H), 3.02 (t, *J* = 5.6 Hz, 2H). UPLC-MS: *t*_R_ = 1.28 min; MS (ESI) *m*/*z*: calcd for C_13_H_17_N_2_O (M + H)^+^, *m*/*z*: 217.3; found, 217.0 (M + H)^+^.

#### 7-Fluoro-10-methyl-1,2,3,4,5,6-hexahydroazepino[4,5-*b*]indole Hydrochloride (**61**)

Following Gp1a, the title compound was obtained from (2-fluoro-5-methyl-phenyl)hydrazine hydrochloride (**44a**) (0.58 g, 3.29 mmol) and *tert*-butyl 4-oxo-azepane-1-carboxylate (**60**) (0.7 g, 3.29 mmol) as a brown solid (0.2 g, 28%). UPLC-MS: *t*_R_ = 1.69 min; MS calcd for C_13_H_15_FN_2_*m*/*z*: 217.32; found, 218.1 [M + H]^+^.

### Preparation of Tetrahydro-γ-carboline **1–42** (Schemes 1–4)

#### General Procedure 1 (GP1)

The properly substituted 2,3,4,5-tetrahydropyrido[4,3-*b*]indole or hexahydroazepino[4,5-*b*]indole (1.0 equiv) and the desired, commercially available carboxylic acid (1.1 equiv), placed in a moisture-free round-bottom flask, were dissolved in anhydrous DMF (from 0.5 to 2.0 mL) under the argon atmosphere and cooled to 0 °C in an ice bath. The chosen coupling agent and a base, usually HATU (1.2 equiv) and DIPEA (2.0 equiv), were added to the reaction mixture, and the suspension was stirred at 0 °C for 30 min, then warmed to 25 °C, and stirred for 16 h. Work-up was then started: EtOAc was added to the reaction mixture, and the organic phase was washed with HCl 1.0 M (3 × 2 mL), followed by NaHCO_3_ SS (3 × 2 mL) and brine (3 × 2 mL). The organic phase was dried over Na_2_SO_4_ and evaporated. Crude product was purified by flash chromatography (eluting mixture composed by EtOAc in cyclohexane or DCM in MeOH) and eventually triturated with 10–20% DCM in *n*-pentane or 100% cyclohexane. The purified product was dried in Hi-Vac or lyophilized when necessary.

#### General Procedure 2 (GP2)

Commercially available carboxylic acid (1.0 equiv), substituted 2,3,4,5-tetrahydropyrido[4,3-*b*]indole (1.0 equiv), Et_3_N (2.0 equiv), and EDC·HCl (1.1 equiv) were dissolved in DCM (0.15–0.2 M solution) and stirred at r.t. for 16 h. The reaction was quenched with aq. HCl 2.0 M solution (10 mL), and the aqueous phase was extracted with EtOAc (2 × 5 mL). The combined extracts were washed with water (2 mL), brine (2 mL), dried over Na_2_SO_4_, filtered, and concentrated *in vacuo* to afford the crude product. The crude product was purified by flash chromatography (eluting mixture composed by EtOAc in cyclohexane or DCM in MeOH) and eventually triturated with 10–20% DCM in *n*-pentane or 100% cyclohexane. The purified product was dried in Hi-Vac or lyophilized when necessary.

#### (6-(Dimethylamino)-1*H*-indol-2-yl)-(1,3,4,5-tetrahydro-2*H*-pyrido[4,3-*b*]indol-2-yl)methanone (**1**)

Following GP1, the title compound was obtained from commercially available 2,3,4,5-tetrahydro-1*H*-pyrido[4,3-*b*]indole (**45p**) (0.05 g, 0.13 mmol) and 6-(dimethylamino)-1*H*-indole-2-carboxylic acid (**A**) (0.03 g, 0.13 mmol) after purification by preparative HPLC-MS [mobile phase: H_2_O (A) and CH_3_CN (B); linear gradient: 0–0.5 min 10% B; and 0.5–7.0 min 100% B] as a white solid (0.014 g, 34%). ^1^H NMR (600 MHz, DMSO-*d*_6_): δ 11.11 (s, 1H, NH), 10.97 (s, 1H, NH), 7.46 (d, *J* = 8.6 Hz, 1H), 7.44 (d, *J* = 8.4 Hz, 1H), 7.31 (d, *J* = 8.0 Hz, 1H), 7.04 (t, *J* = 7.6 Hz, 1H), 6.96 (t, *J* = 7.5, Hz, 1H), 6.85 (s, 1H), 6.74 (dd, *J* = 8.9, 2.3 Hz, 1H), 6.63 (d, *J* = 2.2 Hz, 1H), 4.93 (s, 2H), 4.10 (s, 2H), 2.97 (s, 2H), 2.92 (s, 6H). ^13^C NMR (150 MHz, DMSO-*d*_6_): δ 162.8 (Cq), 148.4 (Cq), 137.9 (Cq), 135.9 (Cq), 132.7 (Cq), 127.8 (Cq), 125.1 (Cq), 121.8 (CH), 120.7 (CH), 119.1 (Cq), 118.6 (CH), 117.2 (CH), 111.0 (CH), 110.0 (CH), 105.7 (Cq), 104.4 (CH). 93.8 (CH), 44.0 (CH_2_), 41.0 (CH_3_, 2C), 40.0 (CH_2_), 23.5 (CH_2_). UPLC-MS: *t*_R_ = 2.27 min; MS (ESI) *m*/*z*: calcd for C_22_H_23_N_4_O (M + H)^+^, 359.2; found, 359.3. HRMS (AP-ESI) *m*/*z*: calcd for C_22_H_23_N_4_O [M + H]^+^, 359.1872; found, 359.1862.

#### (1-Phenyl-1*H*-pyrazol-4-yl)-(1,3,4,5-tetrahydro-2*H*-pyrido[4,3-*b*]indol-2-yl)methanone (**2**)

Following GP1, the title compound **2** was obtained from commercially available 2,3,4,5-tetrahydro-1*H*-pyrido[4,3-*b*]indole (**45p**) (0.06 g, 0.32 mmol) and 1-phenylpyrazole-4-carboxylic acid (**B**) (0.03 g, 0.32 mmol), after purification by silica gel flash column chromatography with DCM/EtOAc (8:2) as the eluent, as a white solid (0.03 g, 26%). ^1^H NMR showed the presence of two conformers C*a*/*Cb* in a 56/44 ratio. ^1^H NMR (600 MHz, DMSO-*d*_6_): δ 10.97 (s, NH, 1H, C*a* + *Cb*), 8.90 (s, 1H, C*a* + *Cb*), 8.08 (s, 1H, C*a* + *Cb*), 7.94 (m, 2H, C*a* + *Cb*), 7.55–7.52 (m, 2H, C*a* + *Cb*), 7.46 (d, *J* = 7.8 Hz, 1H, C*a* + *Cb*), 7.37 (tt, *J* = 7.4, 1.1 Hz, 1H, C*a* + *Cb*), 7.31 (d, *J* = 8.3 Hz, 1H, C*a* + *Cb*), 7.04 (br s, 1H, C*a* + *Cb*), 6.96 (s, 1H, C*a* + *Cb*), 4.94 (m, 2H *Cb*), 4.77 (m, 2H, C*a*), 3.98 (m, 2H, C*a* + *Cb*), 3.00 (s, 2H, C*a*), 2.88 (s, 2H, *Cb*). ^13^C NMR (150 MHz, DMSO-*d*_6_): δ 163.0 (Cq, C*a* + *Cb*), 141.4 (CH, *Cb*), 140.9 (CH, C*a*), 139.2 (Cq, C*a* + *Cb*), 135.9 (Cq, C*a* + *Cb*), 132.7 (Cq, C*a* + *Cb*), 129.6 (CH, 2C, C*a* + *Cb*), 129.2 (Cq, *Cb*), 128.7 (Cq, C*a*), 126.9 (CH, C*a* + *Cb*), 125.2 (Cq, C*a*), 125.0 (Cq, *Cb*), 120.7 (CH, C*a* + *Cb*), 119.1 (CH, C*a* + *Cb*), 118.9 (CH, 2C, C*a* + *Cb*), 118.5 (CH, C*a* + *Cb*), 117.3 (CH, C*a* + *Cb*), 110.9 (CH, C*a* + *Cb*), 105.5 (Cq, C*a* + *Cb*), 44.8 (CH_2_, C*a*), 44.7 (CH_2_, *Cb*), 40.1 (CH_2_, C*a* + *Cb*), 24.0 (CH_2_, C*a*), 22.8 (CH_2_, C*b*). UPLC-MS: *t*_R_ = 2.12 min; MS (ESI) *m*/*z*: calcd for C_21_H_19_N_4_O (M + H)^+^, 343.1; found, 343.2. HRMS (AP-ESI) *m*/*z*: calcd for C_21_H_19_N_4_O [M + H]^+^, 343.1559; found, 343.1552.

#### (8-Methoxy-1,3,4,5-tetrahydropyrido[4,3-*b*]indol-2-yl)-[5-(trifluoromethyl)-1*H*-pyrazol-3-yl]methanone (**3**)

Following GP1, the title compound was obtained from 8-methoxy-2,3,4,5-tetrahydro-1*H*-pyrido[4,3-*b*]indole (**45q**) (0.04 g, 0.20 mmol) and 5-(trifluoromethyl)-1*H*-pyrazole-3-carboxylic acid (**C**) (0.036 g, 0.20 mmol), after purification by silica gel flash column chromatography with DCM/EtOAc (8:2) as the eluent, as a white solid (0.055 g, 76%). ^1^H NMR showed the presence of two conformers C*a*/*Cb* in a 64/36 ratio. ^1^H NMR (400 MHz, DMSO-*d*_6_): δ 14.38 (s, NH, 1H, C*a* + *Cb*), 10.79 (s, NH, 1H, C*a* + *Cb*), 7.26 (br s, 1H, *Cb*), 7.19 (m, 2H, C*a*, 1H, *Cb*), 7.00 (br s, 1H, C*a*), 6.95 (br s, 1H, *Cb*), 6.68 (d, *J* = 8.3 Hz, 1H, C*a* + *Cb*), 4.84 (m, 2H, C*a*), 4.78 (s, 2H, *Cb*), 4.00 (s, 2H, *Cb*), 3.92 (s, 2H, C*a*), 3.76 (s, 3H, C*a*), 3.72 (s, 3H, *Cb*), 2.96 (s, 2H, C*a*), 2.87 (s, 2H, *Cb*). ^13^C NMR (150 MHz, DMSO-*d*_6_): δ 159.4 (Cq, C*a*), 159.1 (Cq, *Cb*), 153.2 (Cq, C*a* + *Cb*), 140.9 (Cq, ^2^*J*_CF_ = 39.2 Hz, C*a* + *Cb*), 137.7 (Cq, C*a* + *Cb*), 133.2 (Cq, *Cb*), 132.8 (Cq, C*a*), 130.9 (Cq, C*a* + *Cb*), 125.5 (Cq, C*a*), 125.2 (Cq, *Cb*), 121.0 (Cq, ^1^*J*_CF_ = 268.0 Hz, Cq, CF3, C*a* + *Cb*), 111.6 (CH, C*a* + *Cb*), 110.5 (CH, C*a* + *Cb*), 104.9 (Cq, C*a* + *Cb*), 104.7 (CH, *Cb*), 104.0 (CH, C*a*) 99.9 (CH, *Cb*), 99.6 (CH, C*a*), 55.4 (CH_3_, *Cb*), 55.3 (CH_3_, C*a*), 44.6 (CH_2_, *Cb*), 44.4 (CH_2_, C*a*), 40.0 (CH_2_, C*a* + *Cb*), 23.9 (CH_2_, C*a*), 22.8 (CH_2_, *Cb*). ^19^F NMR (376 MHz, DMSO-*d*_6_): δ −59.7 (s) UPLC-MS: *t*_R_ = 2.00 min; MS (ESI) *m*/*z*: calcd for C_17_H_16_F_3_N_4_O_2_ (M + H)^+^, 365.1; found, 365.1. HRMS (AP-ESI) *m*/*z*: calcd for C_17_H_16_F_3_N_4_O_2_ [M + H]^+^, 365.1225; found, 365.1217.

#### (8-Methoxy-1,3,4,5-tetrahydropyrido[4,3-*b*]indol-2-yl)-(1*H*-pyrazol-3-yl)methanone (**4**)

Following GP1, the title compound was obtained from commercially available 8-methoxy-2,3,4,5-tetrahydro-1*H*-pyrido[4,3-*b*]indole (**45q**) (0.07 g, 0.35 mmol) and 1*H*-pyrazole-3-carboxylic acid (**D**) (0.039 g, 0.35 mmol), after purification by silica gel flash column chromatography with DCM/EtOAc (55:45) as the eluent, as a white solid (0.03 g, 30%). ^1^H NMR showed the presence of two conformers C*a*/*Cb* in a 64/36 ratio. ^1^H NMR (600 MHz, DMSO-*d*_6_): δ 13.26 (br s, NH, 1H, C*a* + *Cb*), 10.71 (s, NH, 1H, C*a* + *Cb*), 7.79 (s, 1H, C*a* + *Cb*), 7.19 (d, *J* = 8.6 Hz, 1H, C*a* + *Cb*), 6.99 (br s, 1H C*a*), 6.77 (br s, *Cb*), 6.68 (m, 1H, C*a* + *Cb*), 6.62 (s, 1H, C*a* + *Cb*), 5.02 (br s, 2H, *Cb*), 4.77 (br s, C*a*), 4.14 (br s, 2H, C*a*), 3.99 (br s, 2H, *Cb*), 3.77 (br s, 2H, C*a*), 3.72 (2H, *Cb*). ^13^C NMR (150 MHz, DMSO-*d*_6_): δ 163.5 (Cq, C*a* + *Cb*), 153.6 (Cq, C*a* + *Cb*), 133.9 (Cq, C*a*), 133.6 (Cq, *Cb*), 131.4 (Cq, C*a* + *Cb*), 129.7 (Cq, C*a* + *Cb*), 129.9 (CH, C*a* + *Cb*, extrapolated from HSQC), 126.1 (Cq, C*a*), 125.7 (Cq, *Cb*), 112.0 (CH, C*a* + *Cb*), 110.8 (CH, C*a* + *Cb*), 107.3 (CH, C*a* + *Cb*), 106.4 (Cq, *Cb*), 106.0 (Cq, C*a*), 100.1 (CH, C*a*), 99.8 (CH, *Cb*), 44.7 (CH_2_, C*a* + *Cb*), 40.5 (CH_2_, C*a* + *Cb*), 24.7 (CH_2_, C*a*), 23.4 (CH_2_, *Cb*). UPLC-MS: *t*_R_ = 1.52 min; MS (ESI) *m*/*z*: calcd for C_16_H_17_N_4_O_2_ (M + H)^+^, 297.1; found, 297.2. HRMS (AP-ESI) *m*/*z*: calcd for C_16_H_17_N_4_O_2_ [M + H]^+^, 297.1352; found, 297.1336.

#### (8-Methoxy-1,3,4,5-tetrahydro-2*H*-pyrido[4,3-*b*]indol-2-yl)-(5-methyl-1*H*-pyrazol-3-yl)methanone (**5**)

Following GP1, the title compound was obtained from commercially available 8-methoxy-2,3,4,5-tetrahydro-1*H*-pyrido[4,3-*b*]indole (**45q**) (0.04 g, 0.20 mmol) and 5-methyl-1*H*-pyrazole-3-carboxylic acid (**E**) (0.25 g, 0.20 mmol), after purification by silica gel flash column chromatography with DCM/MeOH (95:5) as the eluent, as an off-white solid (0.058 g, 95%). ^1^H NMR showed the presence of two conformers C*a*/*Cb* in a 71/29 ratio. ^1^H NMR (600 MHz, DMSO-*d*_6_): δ 12.85 (s, 1H, NH, C*a* + *Cb*), 10.70 (s, 1H, NH, C*a* + *Cb*), 7.18 (m, 1H, C*a* + *Cb*), 6.98 (s, 1H, C*a*), 6.76 (s, 1H, *Cb*), 6.68 (m, 1H, C*a* + *Cb*), 6.30 (m, 1H, C*a* + *Cb*), 5.06 (s, 2H, *Cb*), 4.75 (s, 2H, C*a*), 4.18–3.98 (m, 2H, C*a* + *Cb*), 3.77 (s, 3H, C*a*), 3.73 (s, 3H, *Cb*), 2.89–2.97 (m, 2H, C*a* + *Cb*), 2.28 (m, 3H, C*a* + *Cb*). ^13^C NMR (150 MHz, DMSO-*d*_6_): δ 163.3 (Cq, C*a* + *Cb*), 153.1 (Cq, C*a* + *Cb*), 147.2 (Cq, C*a* + *Cb*), 138.7 (Cq, C*a* + *Cb*), 133.4 (Cq, *Cb*), 133.1 (Cq, C*a*), 130.9 (Cq, C*a* + *Cb*), 125.6 (Cq, C*a*), 125.2 (Cq, *Cb*), 111.5 (CH, C*a* + *Cb*), 110.2 (CH, C*a* + *Cb*), 106.0 (Cq, C*a* + *Cb*), 105.9 (CH, C*a*), 105.5 (CH, *Cb*), 99.6 (CH, C*a*), 99.2 (CH, *Cb*), 55.3 (CH_3_, C*a* + *Cb*), 44.0 (CH_2_, C*a* + *Cb*), 40.1 (CH_2_, C*a* + *Cb*), 24.2 (CH_2_, C*a*), 22.96 (CH_2_, *Cb*), 10.16 (CH_3_, C*a* + *Cb*). UPLC-MS: *t*_R_ = 1.61 min; MS (ESI) *m*/*z*: calcd for C_17_H_19_N_4_O_2_ (M + H)^+^, 311.1; found, 311.1. HRMS (AP-ESI) *m*/*z*: calcd for C_17_H_19_N_4_O_2_ [M + H]^+^, 311.1508; found 311.1502.

#### (8-Methoxy-1,3,4,5-tetrahydro-2*H*-pyrido-(5-isopropyl-1*H*-pyrazol-3-yl)-[4,3-*b*]indol-2-yl)methanone (**6**)

Following GP1, the title compound was obtained from commercially available 8-methoxy-2,3,4,5-tetrahydro-1*H*-pyrido[4,3-*b*]indole (**45q**) (0.04 g, 0.20 mmol) and 5-isopropyl-1*H*-pyrazole-3-carboxylic acid (**F**) (0.031 g, 0.20 mmol), after purification by silica gel flash column chromatography with DCM/EtOAc (7:3) as the eluent, as a pink solid (0.052 g, 78%). ^1^H NMR showed the presence of two conformers C*a*/*Cb* in a 69/31 ratio. ^1^H NMR (600 MHz, DMSO-*d*_6_): δ 12.95 (s, 1H, NH, C*a* + *Cb*), 10.73 (s, 1H, NH, C*a* + *Cb*), 7.19–7.16 (m, 1H, C*a* + *Cb*), 6.98 (s, 1H, C*a*), 6.75 (s, 1H, *Cb*), 6.69–6.66 (m, 1H, C*a* + *Cb b*), 6.36 (m, 1H, C*a* + *Cb*), 5.11 (s, 2H, *Cb b*), 4.74 (2H, C*a*), 4.20 (dd, *J* = 5.5, 5.5 Hz, 2H, C*a*), 3.97 (dd, *J* = 5.5, 5.5 Hz 2H, *Cb*), 3.76 (s, 3H, C*a*), 3.72 (s, 3H, *Cb*), 2.99 (quint, *J* = 6.9 Hz, 1H, C*a* + *Cb*), 2.88 (dd, *J* = 5.3, 5.3 Hz, 2H, C*a*), 2.82 (dd, *J* = 5.0, 5.0 Hz, 2H, C*a*), 1.25 (s, 6H, C*a* + *Cb*). ^13^C NMR (150 MHz, DMSO-*d*_6_): δ 163.3 (Cq, C*a* + *Cb*), 153.1 (Cq, C*a* + *Cb*), 149.8 (Cq, C*a*), 149.7 (Cq, *Cb*), 146.9 (Cq, C*a* + *Cb b*), 133.5 (Cq, *Cb*), 133.2 (Cq, C*a*), 130.9 (Cq, *Cb*), 125.7 (Cq, *Cb*), 125.2 (Cq, C*a*), 111.6 (CH, *Cb*), 111.5 (CH, C*a*), 110.3 (CH, C*a* + *Cb*), 106.1 (Cq, *Cb*), 105.6 (Cq, C*a*), 103.8 (CH, *Cb*), 103.5 (CH, C*a*), 99.6 (CH, C*a*), 99.2 (CH, *Cb*), 55.3 (CH_3_, C*a* + *Cb*), 44.1 (CH_2_, C*a* + *Cb*), 40.0 (CH_2_, C*a* + *Cb*), 25.0 (CH, C*a*), 24.3 (CH, *Cb*), 23.0 (CH_2_, C*a*), 22.8 (CH_2_, *Cb*), 22.2 (CH_3_, 2C, C*a* + *Cb*). UPLC-MS: *t*_R_ = 1.85 min; MS (ESI) *m*/*z*: calcd for C_19_H_23_N_4_O_2_ (M + H)^+^, 339.1; found, 339.2. HRMS (AP-ESI) *m*/*z*: calcd for C_19_H_23_N_4_O_2_ [M + H]^+^, 339.1821; found, 339.1813.

#### (8-Methoxy-1,3,4,5-tetrahydro-2*H*-pyrido[4,3-*b*]indol-2-yl)-(5-phenyl-1*H*-pyrazol-3-yl)methanone (**7**)

Following GP2, the title compound was obtained from commercially available 8-methoxy-2,3,4,5-tetrahydro-1*H*-pyrido[4,3-*b*]indole (**45q**) (0.03 g, 0.15 mmol) and 5-phenyl-1*H*-pyrazole-3-carboxylic acid (**G**) (0.028 g, 0.15 mmol), after purification by silica gel flash column chromatography with DCM/EtOAc (50:50) as the eluent, as a white solid (0.016 g, 29%). ^1^H NMR showed the presence of two conformers C*a*/*Cb* in a 64/36 ratio. ^1^H NMR (600 MHz, DMSO-*d*_6_): δ 13.70 (s, 1H, NH, C*a* + *Cb*), 10.78 (s, 1H, NH, C*a* + *Cb*), 7.84 (m, 2H, C*a* + *Cb*), 7.46 (m, 2H, C*a*), 7.36 (m, 1H, C*a* + *Cb*), 7.20–7.17 (m, 1H, C*a* + *Cb*), 7.09 (m, 1H, C*a* + *Cb*), 7.00 (s, 1H, C*a*), 6.80 (s, 1H, *Cb*), 6.69–6.65 (m, 1H, C*a* + *Cb*), 5.06 (s, 2H, *Cb*), 4.78 (2H, C*a*), 4.16–4.02 (m, 2H, C*a* + *Cb*), 3.77 (s, 3H, C*a*), 3.71 (s, 3H, *Cb*), 2.94–2.96 (m, 2H, C*a* + *Cb*). ^13^C NMR (150 MHz, DMSO-*d*_6_): δ 162.9 (Cq, C*a* + *Cb*), 153.2 (Cq, C*a* + *Cb*), 142.5 (Cq, C*a* + *Cb*), 133.4 (Cq, *Cb*), 133.1 (Cq, C*a*), 130.9 (Cq, C*a* + *Cb*), 129.0 (CH, 3C, C*a*), 128.2 (CH, 3C, *Cb*), 125.6 (CH, 2C, *Cb*), 125.3 (CH, 2C, C*a*), 111.5 (CH, C*a* + *Cb*), 110.3 (CH, C*a* + *Cb b*), 105.4 (Cq, C*a* + *Cb*), 104.3 (CH, C*a* + *Cb*), 99.6 (CH, C*a*), 99.3 (CH, *Cb*), 55.3 (CH_3_, C*a* + *Cb*), 44.4 (CH_2_, C*a* + *Cb*), 40.1 (CH_2_, C*a* + *Cb*), 24.2 (CH_2_, C*a*), 23.0 (CH_2_, *Cb*). UPLC-MS: *t*_R_ = 2.00 min; MS (ESI) *m*/*z*: calcd for C_22_H_21_N_4_O_2_ (M + H)^+^, 373.2; found, 373.2. HRMS (AP-ESI) *m*/*z*: calcd for C_22_H_21_N_4_O_2_ [M + H]^+^ 373.1665; found 373.1651.

#### (8-Methoxy-1,3,4,5-tetrahydro-2*H*-pyrido[4,3-*b*]indol-2-yl)-(2-(trifluoromethyl)-1*H*-imidazol-4-yl)methanone (**8**)

Following GP2, the title compound was obtained from 8-methoxy-2,3,4,5-tetrahydro-1*H*-pyrido[4,3-*b*]indole (**45q**) (0.03 g, 0.15 mmol) and 2-(trifluoromethyl)-1*H*-imidazole-4-carboxylic acid (**H**) (0.027 g, 0.15 mmol), after purification by silica gel flash column chromatography with DCM/EtOAc (60:40) as the eluent, as a white solid (0.022 g, 40%). ^1^H NMR showed the presence of two conformers C*a*/*Cb* in a 69/31 ratio. ^1^H NMR (600 MHz, DMSO-*d*_6_): δ 10.75 (s, 1H, NH, C*a* + *Cb*), 7.90 (br s, 1H, C*a* + *Cb*), 7.18 (d, *J* = 8.7 Hz, 1H, C*a* + *Cb*), 6.98 (br s, 1H, C*a*), 6.80 (br s, 1H, *Cb*), 6.67 (dd, *J* = 8.7, 2.4 Hz, 1.45H, C*a* + *Cb*), 5.09 (s, 2H, *Cb*), 4.74 (s, 2H, C*a*), 4.25 (s, 2H, C*a*), 3.97 (br s, 2H, *Cb*), 3.75 (br s, 3H, C*a* + *Cb*), 2.91–2.84 (m, 2H, C*a* + *Cb*). ^13^C NMR (150 MHz, DMSO-*d*_6_): δ 161.9 (Cq, C*a* + *Cb*), 153.1 (Cq, C*a* + *Cb*), 137.1 (Cq, C*a* + *Cb*), 134.3 (Cq, C*a* + *Cb*), 133.4 (Cq, *Cb*), 133.1 (Cq, C*a*), 130.9 (Cq, C*a* + *Cb*), 125.6 (Cq, C*a* + *Cb*), 125.2 (CH, C*a* + *Cb*), 118.7 (Cq, ^1^*J*_CF_ = 268.9 Hz, C*a* + *Cb*), 111.6 (CH, C*a* + *Cb*), 110.3 (CH, C*a* + *Cb*), 105.9 (Cq, *Cb*), 105.4 (Cq, C*a*), 99.5 (CH, C*a* + *Cb*), 55.3 (CH_3_, C*a* + *Cb*), 44.0 (CH_2_, C*a* + *Cb*), 40.1 (CH_2_, C*a* + *Cb*), 24.2 (CH_2_, C*a*), 23.0 (CH_2_, *Cb*). ^19^F NMR (376 MHz, DMSO-*d*_6_): δ −60.8 (s). UPLC-MS: *t*_R_ = 1.69 min; MS (ESI) *m*/*z*: calcd for C_17_H_16_F_3_N_4_O_2_ (M + H)^+^, 365.1; found, 365.1. HRMS (AP-ESI) *m*/*z*: calcd for C_22_H_22_N_4_O [M + H]^+^, 365.1225; found, 365.1219.

#### (8-Methoxy-1,3,4,5-tetrahydro-2*H*-pyrido[4,3-*b*]indol-2-yl)-(5-bromofuran-2-yl)methanone (**9**)

Following GP2, the title compound was obtained from commercially available 8-methoxy-2,3,4,5-tetrahydro-1*H*-pyrido[4,3-*b*]indole (**45q**) (0.063 g, 0.26 mmol) and 5-bromofuran-2-carboxylic acid (**J**) (0.05 g, 0.26 mmol), after purification by silica gel flash column chromatography with cyclohexane/EtOAc (8:2) as the eluent, as an off-white solid (0.076 g, 77%). ^1^H NMR (600 MHz, CDCl_3_): δ 8.09 (s, NH), 7.19 (d, *J* = 8.7 Hz, 1H), 7.00 (d, *J* = 3.4 Hz, 1H), 6.9 (br s, 1H), 6.81 (d, *J* = 8.9 Hz, 1H), 6.44 (d, *J* = 3.5 Hz, 1H), 4.92 (br s, 2H), 4.08 (t, *J* = 5.9 Hz, 2H), 3.85 (s, 3H), 2.95 (br s, 2H). ^13^C NMR (151 MHz, CDCl_3_): δ 159.0 (Cq), 154.3 (Cq), 149.9 (Cq), 131.2 (Cq, 2C), 126.0 (Cq), 124.5 (Cq), 118.7 (CH), 113.5 (CH), 111.7 (CH, 2C), 106.8 (Cq), 100.2 (CH), 56.09 (CH_3_), 44.6 (CH_2_), 41.2 (CH_2_), 24.3 (CH_2_). UPLC-MS: *t*_R_ = 2.07 min; MS (ESI) *m*/*z*: calcd for C_17_H_16_BrN_2_O_3_ (M + H)^+^, 375.0; found, 375.1. HRMS (AP-ESI) *m*/*z*: calcd for C_17_H_16_BrN_2_O_3_ [M + H]^+^, 375.0344; found, 375.0335.

#### (8-Methoxy-1,3,4,5-tetrahydro-2*H*-pyrido[4,3-*b*]indol-2-yl)-(6-(dimethylamino)-1*H*-indol-2-yl)methanone (**10**)

Following GP2, the title compound was obtained from commercially available 8-methoxy-2,3,4,5-tetrahydro-1*H*-pyrido[4,3-*b*]indole (**45q**) (0.025 g, 0.12 mmol) and 6-(dimethylamino)-1*H*-indole-2-carboxylic acid (**A**) (0.025 g, 0.12 mmol), after purification by silica gel flash column chromatography with DCM/EtOAc (50:50) as the eluent, as a white solid (0.023 g, 50%). ^1^H NMR (400 MHz, DMSO-*d*_6_): δ 11.09 (s, NH, 1H), 10.76 (s, NH, 1H), 7.45 (d, *J* = 8.6 Hz, 1H), 7.18 (d, *J* = 8.6 Hz, 1H), 6.97 (s, 1H), 6.86 (s, 1H), 6.74 (dd, *J* = 8.9, 2.3 Hz, 1H), 6.68 (dd, *J* = 8.7, 2.4 Hz, 1H), 6.63 (s, 1H), 4.89 (br s, 2H), 4.05 (s, 2H), 3.74 (s, 3H), 2.93 (br s, 2H), 2.90 (s, 6H). ^13^C NMR (151 MHz, DMSO-*d*_6_): δ 162.9 (Cq), 153.2 (Cq), 148.5 (Cq), 137.9 (Cq), 133.3 (Cq), 130.93 (Cq), 127.8 (Cq), 125.5 (Cq), 121.8 (CH), 119.1 (Cq), 111.6 (CH), 110.4 (CH), 110.0 (CH), 105.6 (Cq), 104.4 (CH), 99.5 (CH), 93.8 (CH), 59.8 (CH_2_), 55.3 (CH_3_), 41.0 (2C, CH_3_), 40.1 (CH_2_), 23.7 (CH_2_). UPLC-MS: *t*_R_ = 2.17 min; MS (ESI) *m*/*z*: calcd for C_23_H_25_N_4_O_2_ (M + H)^+^, 389.2; found, 389.2. HRMS (AP-ESI) *m*/*z*: calcd for C_23_H_25_N_4_O_2_ [M + H]^+^, 389.1978; found, 389.1973.

#### (8-Methoxy-1,3,4,5-tetrahydro-2*H*-pyrido[4,3-*b*]indol-2-yl)-(1-methyl-5-(trifluoromethyl)-1*H*-pyrazol-3-yl)methanone (**11**)

Following GP1, the title compound was obtained from commercially available 8-methoxy-2,3,4,5-tetrahydro-1*H*-pyrido[4,3-*b*]indole (**45q**) (0.052 g, 0.26 mmol) and 2-(trifluoromethyl)-1*H*-imidazole-4-carboxylic acid (**K**) (0.05 g, 0.26 mmol), after purification by silica gel flash column chromatography with DCM/EtOAc (80:20) as the eluent, as a white solid (0.022 g, 22%). ^1^H NMR showed the presence of two conformers C*a*/*Cb* in a 67/33 ratio. ^1^H NMR (600 MHz, DMSO-*d*_6_): δ 10.77 (s, 1H, C*a* + *Cb*), 7.22 (s, 1H, C*a* + *Cb*), 7.19 (d, *J* = 8.6 Hz, 1H, C*a* + *Cb*), 7.17 (br s, 1H, *Cb*), 6.99 (d, *J* = 2.4 Hz, 1H, C*a*), 6.84 (d, *J* = 2.3 Hz, 1H, *Cb*), 6.69 (dd, *J* = 8.6, 2.5 Hz, 1H, C*a*), 6.67 (dd, *J* = 8.6, 2.5 Hz, 1H, C*b*), 4.95 (s, 2H, *Cb*), 4.78 (s, 2H, C*a*), 4.07 (t, *J* = 5.6 Hz, 2H, C*a*), 4.05 (s, 3H, C*a* + *Cb*), 3.99 (t, *J* = 5.6 Hz, 2H, *Cb*), 3.76 (s, 3H, C*a*), 3.76 (s, 3H, *Cb*), 2.90 (t, *J* = 5.8 Hz, 2H, C*a*), 2.90 (t, *J* = 5.8 Hz, 2H, *Cb*). ^13^C NMR (151 MHz, DMSO-*d*_6_): δ 161.4 (Cq, C*a* + *Cb*), 153.2 (Cq, C*a* + *Cb*), 145.8 (Cq, C*a* + *Cb*), 133.3 (Cq, *Cb*), 132.9 (Cq, C*a*), 131.3 (q, ^2^*J*_CF_ = 38.8 Hz, C*a* + *Cb*), 130.9 (Cq, C*a* + *Cb*), 125.6 (Cq, C*a*), 125.2 (Cq, *Cb*), 119.7 (Cq, ^1^*J*_CF_ = 268.4 Hz, C*a* + *Cb*), 111.6 (CH, C*a* + *Cb*), 110.4 (CH, C*a*), 110.3 (CH, *Cb*), 109.8 (CH, *Cb*), 109.7 (CH, C*a*), 105.6 (Cq, *Cb*), 105.1 (Cq, C*a*), 99.7 (CH, *Cb*), 99.6 (CH, C*a*), 55.3 (CH_3_, C*a* + *Cb*), 44.3 (CH_2_, C*a* + *Cb*), 40.1 (CH_2_, C*a* + *Cb*), 38.6 (CH_3_, C*a* + *Cb*), 24.1 (CH_2_, C*a*), 22.9 (CH_2_, *Cb*). ^19^F NMR (376 MHz, DMSO-*d*_6_): δ −58.2 (s). UPLC-MS: *t*_R_ = 2.07 min; MS (ESI) *m*/*z*: calcd for C_18_H_16_F_3_N_4_O_2_ (M – H)^−^, 377.4; found, 377.1. HRMS (AP-ESI) *m*/*z*: calcd for C_18_H_18_F_3_N_4_O_2_ [M + H]^+^, 379.1382; found, 379.1370.

#### (8-Methoxy-5-methyl-1,3,4,5-tetrahydro-2*H*-pyrido[4,3-*b*]indol-2-yl)-(5-(trifluoromethyl)-1*H*-pyrazol-3-yl)methanone (**12**)

Following GP1, the title compound was obtained from 8-methoxy-5-methyl-2,3,4,5-tetrahydro-1*H*-pyrido[4,3-*b*]indole hydrochloride (**48**) (0.068 g, 0.31 mmol) and 2-(trifluoromethyl)-1*H*-imidazole-4-carboxylic acid (**C**) (0.057 g, 0.31 mmol), after purification by silica gel flash column chromatography with DCM/EtOAc (80:20) as the eluent, as a white solid (0.28 g, 24%). ^1^H NMR showed the presence of two conformers C*a*/*Cb* in a 62/38 ratio. ^1^H NMR (600 MHz, DMSO-*d*_6_): δ 7.31–7.26 (m, 1H, C*a* + *Cb*), 7.18 (s, 1H, C*a*), 7.04 (br s, 1H, C*a*), 6.98 (br s, 1H, *Cb*), 6.76–6.74 (m, 1H, C*a* + *Cb*), 4.86 (s, 2H, *Cb*), 4.79 (s, 2H, C*a*), 4.03 (t, *J* = 5.3 Hz, 2H, *Cb b*), 3.95 (t, *J* = 6.0 Hz, 2H, C*a*), 3.77 (s, 3H, C*a*), 3.73 (s, 3H, *Cb*), 3.60 (s, 3H, C*a* + *Cb*), 2.99 (t, *J* = 5.8 Hz, 2H, C*a*), 2.90 (s, 2H, *Cb*). ^13^C NMR (151 MHz, DMSO-*d*_6_): δ 159.4 (Cq, C*a*), 159.3 (Cq, *Cb*), 158.5 (Cq, C*a* + *Cb*), 140.6 (Cq, C*a* + *Cb*), 138.3 (Cq, C*a*), 135.8 (Cq, *Cb*), 135.0 (Cq, *Cb*), 134.3 (Cq, C*a*), 131.9 (Cq, C*a* + *Cb*), 125.0 (Cq, C*a*), 124.6 (Cq *Cb*), 121.5 (Cq, ^1^*J*_CF_ = 268.0 Hz, C*a*), 121.3 (Cq, ^1^*J*_CF_ = 268.0 Hz, *Cb*), 110.5 (CH, *Cb*), 110.1 (CH, C*a*), 107.4 (CH, C*a* + *Cb*), 105.1 (CH, *Cb*), 104.7 (Cq, *Cb*), 104.5 (Cq, C*a*), 104.2 (CH, C*aa*), 100.0 (CH, *Cb*), 99.6 (CH, C*a*), 55.5 (CH_3_, C*a*), 55.4 (CH_3_, *Cb*), 44.5 (CH_2_, C*a* + *Cb*), 40.2 (CH_2_, C*a* + *Cb*), 29.6 (CH_3_, C*a* + *Cb*), 22.8 (CH_2_, C*a*), 21.7 (CH_2_, *Cb*). ^19^F NMR (376 MHz, DMSO-*d*_6_): δ −58.2 (s). UPLC-MS: *t*_R_ = 2.13 min; MS (ESI) *m*/*z*: calcd for C_18_H_16_F_3_N_4_O_2_ (M – H)^−^, 377.4; found, 377.1. HRMS (AP-ESI) *m*/*z*: calcd for C_18_H_18_F_3_N_4_O_2_ [M + H]^+^, 379.1382; found, 379.1371.

#### (1,3,4,5-Tetrahydro-2*H*-pyrido[4,3-*b*]indol-2-yl)-(5-(trifluoromethyl)-1*H*-pyrazol-3-yl)methanone (**13**)

Following GP1, the title compound was obtained from commercially available 2,3,4,5-tetrahydro-1*H*-pyrido[4,3-*b*]indole (**45p**) (0.02 g, 0.12 mmol) and 2-(trifluoromethyl)-1*H*-imidazole-4-carboxylic acid (**C**) (0.02 g, 0.12 mmol), after purification by silica gel flash column chromatography with cyclohexane/EtOAc (80:20) as the eluent, as a white solid (0.02 g, 45%) ^1^H NMR showed the presence of two conformers C*a*/*Cb* in a 59/41 ratio. ^1^H NMR (600 MHz, DMSO-*d*_6_): δ 14.39 (s, 1H, NH, C*a* + *Cb*), 10.99 (s, NH, 1H, C*a* + *Cb*), 7.48–7.42 (1H, C*a* + *Cb*), 7.32–7.27 (1H, C*a* + *Cb*), 7.19 (s, 1H, C*a*), 7.07–7.03 (m, 1H, C*a* + *Cb*), 7.00–6.94 (1H, C*a* + *Cb*), 4.90 (s, 2H, *Cb*), 4.80 (s, 2H, C*a*), 4.03 (br s, 2H, *Cb*), 3.94 (br s, 2H, C*a*), 2.98 (br s, 2H, *Cb*), 2.89 (br s, 2H, C*a*). ^13^C NMR (151 MHz, DMSO-*d*_6_): δ 159.5 (Cq, C*a*), 159.2 (Cq, *Cb*), 140.9 (Cq, C*a* + *Cb*), 137.9 (Cq, C*a* + *Cb*), 135.9 (Cq, C*a* + *Cb*), 132.5 (Cq, *Cb*), 132.3 (Cq, C*a*), 125.2 (Cq, C*a*), 124.8 (Cq, *Cb*), 121.4 (Cq, ^1^*J*_CF_ = 268.4 Hz, C*a* + *Cb*), 120.8 (CH, C*a* + *Cb*), 118.6 (CH, C*a*), 118.5 (CH, C*b*), 117.4 (CH, *Cb*), 117.3 (CH, C*a*), 111.0 (CH, C*a* + *Cb*), 105.1 (Cq, C*a* + *Cb*), 104.8 (CH, *Cb*), 104.2 (CH, C*a*), 44.5 (CH_2_, C*a*), 44.3 (CH_2_, *Cb*), 40.1 (CH_2_, *Cb*), 40.0 (CH_2_, C*a*), 23.8 (CH_2_, C*a*), 22.7 (CH_2_, *Cb*). ^19^F NMR (376 MHz, DMSO-*d*_6_): δ −59.1 (s). UPLC-MS: *t*_R_ = 2.27 min; MS (ESI) *m*/*z*: calcd for C_16_H_14_F_3_N_4_O (M + H)^+^, 335.1; found, 335.3. HRMS (AP-ESI) *m*/*z*: calcd for C_16_H_14_F_3_N_4_O [M + H]^+^, 335.1120; found, 335.1100.

#### (8-Methyl-1,3,4,5-tetrahydro-2*H*-pyrido[4,3-*b*]indol-2-yl)-(5-(trifluoromethyl)-1*H*-pyrazol-3-yl)methanone (**14**)

Following GP1, the title compound was obtained from commercially available 8-methyl-2,3,4,5-tetrahydro-1*H*-pyrido[4,3-*b*]indole (**45r**) (0.037 g, 0.20 mmol) and 5-(trifluoromethyl)-1*H*-pyrazole-3-carboxylic acid (**C**) (0.036 g, 0.20 mmol), after purification by silica gel flash column chromatography with DCM/EtOAc (8:2) as the eluent, as a white solid (0.03 g, 40%). ^1^H NMR showed the presence of two conformers C*a*/*Cb* in a 59/41 ratio. ^1^H NMR (600 MHz, DMSO-*d*_6_): δ 14.37 (s, 1H, NH, C*a* + *Cb*), 10.84 (s, 1H, NH, C*a* + *Cb*), 7.27 (s, 1H, *Cb*), 7.24 (1H, C*a*), 7.20–7.16 (2H, C*a* + *Cb*), 6.88–6.84 (1H, C*a* + *Cb*), 4.86 (s, 2H, *Cb*), 4.77 (s, 2H, C*a*), 4.02 (br s, 2H, *Cb*), 3.92 (br s, 2H, C*a*), 2.96 (br s, 2H, C*a*), 2.87 (br s, 2H, *Cb*), 2.37 (s, 3H, C*a*), 2.33 (s, 3H, *Cb*). ^13^C NMR (151 MHz, DMSO-*d*_6_): δ 159.5 (Cq, C*a*), 159.2 (Cq, *Cb*), 140.9 (Cq, C*a* + *Cb*), 137.9 (Cq, C*a* + *Cb*), 134.3 (Cq, C*a* + *Cb*), 132.5 (Cq, *Cb*), 132.3 (Cq, C*a*), 127.1 (Cq, C*a* + *Cb*), 125.4 (Cq, C*a*), 125.1 (Cq, *Cb*), 122.3 (CH, C*a* + *Cb*), 121.4 (Cq, ^1^*J*_CF_ = 262.2 Hz, C*a* + *Cb*), 117.0 (CH, C*a* + *Cb*), 110.7 (CH, *Cb*), 105.0 (CH, C*a* + *Cb*), 104.6 (Cq, *Cb*), 104.3 (Cq, C*a*), 104.1 (CH, C*a*), 44.5 (CH_2_, C*a*), 44.4 (CH_2_, *Cb*), 40.1 (CH_2_, *Cb*), 40.0 (CH_2_, C*a*), 23.8 (CH_2_, C*a*), 22.7 (CH_2_, *Cb*), 21.2 (CH_3_, C*a*), 21.1 (CH_3_, *Cb*). ^19^F NMR (376 MHz, DMSO-*d*_6_): δ −59.1 (s). UPLC-MS: *t*_R_ = 2.21 min; MS (ESI) *m*/*z*: calcd for C_17_H_16_F_3_N_4_O (M + H)^+^, 349.1; found, 349.1. HRMS (AP-ESI) *m*/*z*: calcd for C_17_H_16_F_3_N_4_O [M + H]^+^, 349.1276; found, 349.1262.

#### (9-Methyl-1,3,4,5-tetrahydro-2*H*-pyrido[4,3-*b*]indol-2-yl)-(5-(trifluoromethyl)-1*H*-pyrazol-3-yl)methanone (**15**)

Following GP2, a mixture of regioisomers **15** and **16** was obtained from a mixture of 7-methyl-2,3,4,5-tetrahydro-1*H*-pyrido[4,3-*b*]indole (**45d′**) and 9-methyl-2,3,4,5-tetrahydro-1*H*-pyrido[4,3-*b*]indole (**45d″**) (0.2 g, 1.07 mmol) and 5-(trifluoromethyl)-1*H*-pyrazole-3-carboxylic acid (**C**) (0.212 g, 1.18 mmol). The title compound was obtained, as a pure regioisomer, after purification by preparative HPLC-MS [mobile phase: H_2_O + 0.1% NH_3_ (A) and CH_3_CN + 0.1% NH_3_ (B); linear gradient: 0–0.5 min 30% B; 0.5–7.0 min 70% B], as a white solid (0.006 g, 7%). ^1^H NMR showed the presence of two conformers C*a*/*Cb* in a 62/38 ratio. ^1^H NMR (400 MHz, DMSO-*d*_6_): δ 14.36 (s, 1H, C*a* + *Cb*), 10.92 (s, 1H, C*a* + *Cb*), 7.15 (s, 1H, C*a* + *Cb*), 7.11 (s, 1H, *Cb*), 7.09 (s, 1H, C*a*), 6.90 (t, *J* = 7.5 Hz, 1H, C*a* + *Cb*), 6.72–6.67 (m, 1H, C*a* + *Cb*), 5.18 (s, 2H, *Cb*), 5.04 (s, 2H, C*a*), 3.94 (br s, 2H, C*a* + *Cb*), 2.96 (br s, 2H, C*a*), 2.87 (br s, 2H, *Cb*), 2.58 (s, 3H, C*a*), 2.46 (s, 3H, *Cb*). NOESY-2D: strong dipolar coupling between multiplet at 5.24–4.96 ppm and singlet at 5.58 ppm. ^13^C NMR (151 MHz, DMSO-*d*_6_): δ 159.8 (Cq, C*a*), 159.5 (Cq, *Cb*), 135.9 (Cq, C*a* + *Cb*), 131.6 (Cq, C*a* + *Cb*), 128.3 (Cq, C*a*), 128.2 (Cq, *Cb*), 124.7 (Cq, C*a* + *Cb*), 124.3 (Cq, C*a* + *Cb*, extrapolated from HMBC), 123.4 (Cq, ^1^*J*_CF_ = 285.6 Hz, C*a* + *Cb*), 120.8 (CH, C*a* + *Cb*), 119.6 (CH, C*a* + *Cb*), 108.8 (CH, C*a* + *Cb*), 105.3 (Cq, C*a* + *Cb*), 104.2 (CH, C*a* + *Cb*), 45.7 (CH_2_, *Cb*), 44.1 (CH_2_, C*a*), 40.4 (CH_2_, *Cb*), 40.1 (CH_2_, C*a*), 23.9 (CH_2_, C*a*), 22.7 (CH_2_, *Cb*), 19.6.2 (CH_3_, C*a*), 19.3 (CH_3_, *Cb*). ^19^F NMR (565 MHz, DMSO-*d*_6_): δ −58.0 (s). UPLC-MS: *t*_R_ = 2.13 min; MS (ESI) *m*/*z*: calcd for C_17_H_16_F_3_N_4_O (M + H)^+^, 349.1; found, 349.1. HRMS (AP-ESI) *m*/*z*: calcd for C_17_H_16_F_3_N_4_O [M + H]^+^, 349.1276; found, 349.1263.

#### (7-Methyl-1,3,4,5-tetrahydro-2*H*-pyrido[4,3-*b*]indol-2-yl)-(5-(trifluoromethyl)-1*H*-pyrazol-3-yl)methanone (**16**)

Following GP2, a mixture of regioisomer **15** and **16** was obtained from a mixture of 7-methyl-2,3,4,5-tetrahydro-1*H*-pyrido[4,3-*b*]indole (**45d′**) and 9-methyl-2,3,4,5-tetrahydro-1*H*-pyrido[4,3-*b*]indole (**45d″**) (0.20 g, 1.07 mmol) and 5-(trifluoromethyl)-1*H*-pyrazole-3-carboxylic acid (**C**) (0.212 g, 1.18 mmol). The title compound was obtained, as a pure regioisomer, after purification by preparative HPLC-MS [mobile phase: H_2_O + 0.1% NH_3_ (A) and CH_3_CN + 0.1% NH_3_ (B); linear gradient: 0–0.5 min 30% B; 0.5–7.0 min 70% B], as a white solid (0.006 g, 7%). ^1^H NMR showed the presence of two conformers C*a*/*Cb* in a 62/38 ratio. ^1^H NMR (400 MHz, DMSO-*d*_6_): δ 14.35 (s, 1H, NH, C*a* + *Cb*), 10.83 (s, NH, 1H, C*a* + *Cb*), 7.33 (d, *J* = 8.3 Hz, 1H, C*a*), 7.29 (d, *J* = 8.4 Hz, 0.6 Hz, 1H, *Cb*), 7.25 (s, 1H, *Cb*), 7.17 (s, 1H, C*a*), 7.10 (s, 1H, C*a*), 7.08 (s, 1H, *Cb*), 6.82 (d, *J* = 8.1 Hz, 1H, C*a*), 6.78 (d, *J* = 8.1 Hz, 1H, *Cb*), 4.87 (s, 2H, *Cb*), 4.77 (s, 2H, C*a*), 4.01 (br s, 2H, *Cb*), 3.93 (br s, 2H, C*a*), 2.95 (br s, 2H, C*a*), 2.86 (br s, 2H, *Cb*), 2.38 (s, 3H, C*a*), 2.36 (s, 3H, *Cb*). ^13^C NMR (151 MHz, DMSO-*d*_6_) for major C*a*: δ 159.6 (Cq), 136.4 (Cq), 131.7 (Cq), 131.5 (Cq), 129.7 (Cq), 123.1 (Cq), 121.6 (Cq, CF_3_, extrapolated from HMBC), 120.3 (CH), 117.0 (CH), 110.9 (CH), 105.0 (Cq), 104.2 (CH), 44.2 (CH_2_), 40.1 (CH_2_), 23.8 (CH_2_), 21.4 (CH_3_). ^19^F NMR (565 MHz, DMSO-*d*_6_): δ −58.0 (s). UPLC-MS: *t*_R_ = 2.17 min; MS (ESI) *m*/*z*: calcd for C_17_H_16_F_3_N_4_O (M + H)^+^, 349.1; found, 349.1. HRMS (AP-ESI) *m*/*z*: calcd for C_17_H_16_F_3_N_4_O [M + H]^+^, 349.1276; found, 349.1263.

#### (6-Methyl-1,3,4,5-tetrahydro-2*H*-pyrido[4,3-*b*]indol-2-yl)-(5-(trifluoromethyl)-1*H*-pyrazol-3-yl)methanone (**17**)

Following GP2, the title compound was obtained from 6-methyl-2,3,4,5-tetrahydro-1*H*-pyrido[4,3-*b*]indole hydrochloride (**45c**) (0.06 g, 0.27 mmol) and 5-(trifluoromethyl)-1*H*-pyrazole-3-carboxylic acid (**C**) (0.053 g, 0.30 mmol), after purification by silica gel flash column chromatography with DCM/EtOAc (85:15) as the eluent, as a white solid (0.04 g, 42%). ^1^H NMR showed the presence of two conformers C*a*/*Cb* in a 61/39 ratio. ^1^H NMR (600 MHz, DMSO-*d*_6_): δ 14.39 (s, 1H, NH, C*a* + *Cb*), 10.91 (s, 1H, NH, C*a* + *Cb*), 7.29–7.26 (m, 1H, C*a* + *Cb*), 7.25–2.23 (m, 1H, *Cb*), 7.20 (s, 1H, C*a*), 6.9 (t, *J* = 7.3 Hz, 1H, C*a*), 6.86–6.84 (m, 1H, C*a*, 2H, *Cb*), 4.88 (s, 2H, *Cb*), 4.79 (s, 2H, C*a*), 4.03 (t, *J* = 5.0 Hz, 2H, *Cb*), 3.93 (t, *J* = 5.2 Hz, 2H, C*a*), 3.00 (br s, 2H, C*a*), 2.90 (br s, 2H, *Cb*), 2.43 (s, 3H, C*a*), 2.42 (s, 1.95H, *Cb*). ^13^C NMR (151 MHz, DMSO-*d*_6_): δ 159.5 (Cq, C*a*), 159.0 (Cq, *Cb*), 140.6 (Cq, *Cb*), 137.8 (Cq, C*a*), 135.4 (Cq, C*a* + *Cb*), 132.3 (Cq, *Cb*), 132.1 (Cq, C*a*), 124.8 (Cq, C*a*), 124.5 (Cq, *Cb*), 121.4 (Cq, ^1^*J*_CF_ = 267.8 Hz, C*a* + *Cb*), 121.4 (CH, C*a* + *Cb*), 120.5 (Cq, C*a* + *Cb*), 118.9 (CH, C*a*), 108.8 (CH, *Cb*), 115.0 (CH, *Cb*), 114.9 (CH, C*a*), 105.5 (Cq, *Cb*), 105.2 (Cq, C*a*), 105.0 (CH, *Cb*), 105.1 (CH, C*a*), 44.6 (CH_2_, C*a*), 44.2 (CH_2_, *Cb*), 40.2 (CH_2_, *Cb*), 40.0 (CH_2_, C*a*), 23.9 (CH_2_, C*a*), 22.8 (CH_2_, *Cb*), 16.8 (CH_3_, C*a* + *Cb*). ^19^F NMR (565 MHz, DMSO-*d*_6_): δ −59.1 (s). UPLC-MS: *t*_R_ = 2.19 min; MS (ESI) *m*/*z*: calcd for C_17_H_16_F_3_N_4_O (M + H)^+^, 349.2; found, 349.2. HRMS (AP-ESI) *m*/*z*: calcd for C_17_H_16_F_3_N_4_O [M + H]^+^, 349.1276; found, 349.1257.

#### (8-Isopropyl-1,3,4,5-tetrahydro-2*H*-pyrido[4,3-*b*]indol-2-yl)-(5-(trifluoromethyl)-1*H*-pyrazol-3-yl)methanone (**18**)

Following GP2, the title compound was obtained from 8-isopropyl-2,3,4,5-tetrahydro-1*H*-pyrido[4,3-*b*]indole hydrochloride (**45e**) (0.06 g, 0.24 mmol) and 5-(trifluoromethyl)-1*H*-pyrazole-3-carboxylic acid (**C**) (0.047 g, 0.26 mmol), after purification by silica gel flash column chromatography with DCM/EtOAc (80:20) as the eluent, as a white solid (0.056 g, 62%). ^1^H NMR showed the presence of two conformers C*a*/*Cb* in a 59/41 ratio. ^1^H NMR (600 MHz, DMSO-*d*_6_): δ 14.38 (s, NH, 1H, C*a* + *Cb*), 10.79 (s, NH, 1H, C*a* + *Cb*), 7.30–7.17 (m, 3H, C*a* + *Cb*), 6.95 (d, *J* = 8.1 Hz, 1H, C*a* + *Cb*), 4.86 (s, 2H, *Cb*), 4.80 (s, 2H, C*a*), 4.01 (br s, 2H, *Cb*), 3.93 (br s, 2H, C*a*), 2.96 (m, 3H, C*a*), 2.88 (m, 3H, *Cb*), 1.24 (m, 6H, C*a* + *Cb*). ^13^C NMR (151 MHz, DMSO-*d*_6_): δ 159.5 (Cq, C*a*), 159.3 (Cq, *Cb*), 140.5 (Cq, C*a* + *Cb*), 138.7 (Cq, C*a* + *Cb*), 138.2 (Cq, C*a* + *Cb*), 134.5 (Cq, C*a* + *Cb*), 132.5 (Cq, *Cb*), 132.2 (Cq, C*a*), 125.3 (Cq, C*a*), 124.9 (Cq, *Cb*), 121.4 (Cq, ^1^*J*_CF_ = 268.1 Hz, C*a* + *Cb*), 119.8 (CH, C*a*), 119.6 (CH, *Cb*), 114.3 (CH, *Cb*), 114.1 (CH, C*a*), 110.7 (CH, C*a* + *Cb*), 104.8 (Cq, C*a* + *Cb*), 104.6 (CH, C*a*), 104.1 (CH, *Cb*), 44.5 (CH_2_, C*a*), 44.4 (CH_2_, *Cb*), 40.2 (CH_2_, *Cb*), 40.0 (CH_2_, C*a* + *Cb*), 33.6 (CH, C*a* + *Cb*), 24.6 (CH_3_, C*a* + *Cb*), 23.8 (CH_2_, C*a*), 22.7 (CH_2_, *Cb*). ^19^F NMR (565 MHz, DMSO-*d*_6_): δ −60.0 (s). UPLC-MS: *t*_R_ = 2.43 min; MS (ESI) *m*/*z*: calcd for C_19_H_20_F_3_N_4_O (M + H)^+^, 377.2; found, 377.2. HRMS (AP-ESI) *m*/*z*: calcd for C_19_H_20_F_3_N_4_O [M + H]^+^, 377.1589; found, 377.1574.

#### (8-Fluoro-1,3,4,5-tetrahydro-2*H*-pyrido[4,3-*b*]indol-2-yl)(5-(trifluoromethyl)-1*H*-pyrazol-3-yl)methanone (**19**)

Following GP2, the title compound was obtained from commercially available 8-fluoro-2,3,4,5-tetrahydro-1*H*-pyrido[4,3-*b*]indole (**45s**) (0.05 g, 0.26 mmol) and 5-(trifluoromethyl)-1*H*-pyrazole-3-carboxylic acid (**C**) (0.047 g, 0.26 mmol), after purification by silica gel flash column chromatography with DCM/EtOAc (80:20) as the eluent, as a white solid (0.012 g, 13%). ^1^H NMR showed the presence of two conformers C*a*/*Cb* in a 61/39 ratio. ^1^H NMR (600 MHz, DMSO-*d*_6_): δ 14.38 (s, NH, 1H, C*a* + *Cb*), 11.10 (s, NH, 1H, C*a* + *Cb*), 7.30–7.26 (m, 2H, C*a* + *Cb*), 7.19 (s, 1H, C*a*), 6.89–6.84 (m, 1H, C*a* + *Cb*), 4.86 (s, 2H, *Cb*), 4.77 (s, 2H, C*a*), 4.02 (br s, 2H, *Cb*), 3.91 (br s, 2H, C*a*), 2.99 (br s, 2H, C*a*), 2.89 (br s, 2H, *Cb*). ^13^C NMR (151 MHz, DMSO-*d*_6_): δ 159.4 (Cq, C*a*), 159.0 (Cq, *Cb*), 156.8 (Cq, d, ^1^*J*_CF_ = 230.9 Hz, C*a* + *Cb*), 141.0 (Cq, q, ^2^*J*_CF_ = 36.6 Hz, C*a* + *Cb*), 137.7 (Cq, C*a*), 137.6 (Cq, *Cb*), 134.8 (Cq, *Cb*), 134.5 (Cq, C*a*), 132.6 (Cq, C*a* + *Cb*), 125.4 (Cq, d, ^3^*J*_CF_ = 7.9 Hz, C*a* + *Cb*), 121.5 (Cq, q, ^1^*J*_CF_ = 268.1 Hz, C*a* + *Cb*), 111.8 (CH, d, ^3^*J*_CF_ = 9.5 Hz, C*a* + *Cb*), 108.6 (CH, d, ^2^*J*_CF_ = 25.9 Hz, C*a* + *Cb*), 105.4 (Cq, C*a* + *Cb*), 105.2 (CH, ^2^*J*_CF_ = 23.7 Hz, C*a* + *Cb*), 104.0 (CH, C*a*), 102.6 (CH, C*a*), 44.5 (CH_2_, C*a*), 44.2 (CH_2_, *Cb*), 40.1 (CH_2_, *Cb*), 40.0 (CH_2_, C*a*), 23.8 (CH_2_, C*a*), 22.8 (CH_2_, *Cb*). ^19^F NMR (565 MHz, DMSO-*d*_6_): δ −58.1 (s, C*a* + *Cb*), −123.0 (s, C*a*), −123.3 (s, *Cb*). UPLC-MS: *t*_R_ = 2.08 min; MS (ESI) *m*/*z*: calcd for C_16_H_13_F_4_N_4_O (M + H)^+^, 353.2; found, 353.2. HRMS (AP-ESI) *m*/*z*: calcd for C_16_H_13_F_4_N_4_O [M + H]^+^, 353.1025; found, 353.1011.

#### (6-Fluoro-1,3,4,5-tetrahydro-2*H*-pyrido[4,3-*b*]indol-2-yl)-(5-(trifluoromethyl)-1*H*-pyrazol-3-yl)methanone (**20**)

Following GP1, the title compound was obtained from 6-fluoro-2,3,4,5-tetrahydro-1*H*-pyrido[4,3-*b*]indole (**45f**) (0.038 g, 0.20 mmol) and 5-(trifluoromethyl)-1*H*-pyrazole-3-carboxylic acid (**C**) (0.036 g, 0.20 mmol), after purification by silica gel flash column chromatography with DCM/EtOAc (8:2) as the eluent, as a white solid (0.052 g, 92%). ^1^H NMR showed the presence of two conformers C*a*/*Cb* in a 62/38 ratio. ^1^H NMR (600 MHz, DMSO-*d*_6_): δ 14.37 (s, NH, 1H, C*a* + *Cb*), 11.44 (s, NH, 1H, C*a* + *Cb*), 7.31–7.25 (m, 2H, C*a* + *Cb*), 7.19 (s, 1H, C*a*), 6.95–6.87 (m, 1H, C*a* + *Cb*), 4.90 (s, 2H, *Cb*), 4.82 (s, 2H, C*a*), 4.04 (br s, 2H, *Cb*), 3.95 (br s, 2H, C*a*), 3.00 (br s, 2H, C*a*), 2.92 (br s, 2H, *Cb*). ^13^C NMR (151 MHz, DMSO-*d*_6_): δ 159.6 (Cq, C*a*), 159.3 (Cq, *Cb*), 148.8 (Cq, d, ^1^*J*_CF_ = 242.1 Hz, C*a* + *Cb*), 140.6 (Cq, C*a* + *Cb*), 138.2 (Cq, C*a* + *Cb*), 133.9 (Cq, *Cb*), 133.7 (Cq, C*a*), 129.1 (Cq, C*a*), 128.8 (Cq, *Cb*), 123.5 (Cq, d, ^3^*J*_CF_ = 12.8 Hz, C*a* + *Cb*), 121.4 (Cq, ^1^*J*_CF_ = 267.8 Hz, C*a* + *Cb*), 119.0 (CH, C*a* + *Cb*), 113.6 (CH, C*a* + *Cb*), 106.2 (Cq, *Cb*), 106.0 (Cq, C*a*), 105.8 (CH, d, ^2^*J*_CF_ = 16.8 Hz, C*a* + *Cb*), 105.0 (CH, *Cb*), 104.2 (CH, C*a*), 44.4 (CH_2_, C*a*), 44.2 (CH_2_, *Cb*), 40.1 (CH_2_, C*a* + *Cb*), 23.8 (CH_2_, C*a*), 22.7 (CH_2_, *Cb*). ^19^F NMR (565 MHz, DMSO-*d*_6_): δ −60.1 (s), −133.7 (s). UPLC-MS: *t*_R_ = 2.12 min; MS (ESI) *m*/*z*: calcd for C_16_H_13_F_4_N_4_O (M + H)^+^, 353.1; found, 353.1. HRMS (AP-ESI) *m*/*z*: calcd for C_16_H_13_F_4_N_4_O [M + H]^+^, 353.1025; found, 353.1007.

#### (8-(Trifluoromethyl)-1,3,4,5-tetrahydro-2*H*-pyrido[4,3-*b*]indol-2-yl)-(5-(trifluoromethyl)-1*H*-pyrazol-3-yl)methanone (**21**)

Following GP2, the title compound was obtained from 8-(trifluoromethyl)-2,3,4,5-tetrahydro-1*H*-pyrido [4,3-*b*]indole (**45g**) (0.1 g, 0.42 mmol) and 5-(trifluoromethyl)-1*H*-pyrazole-3-carboxylic acid (**C**) (0.075 g, 0.42 mmol), after purification by silica gel flash column chromatography with DCM/EtOAc (80:20) as the eluent, as a white solid (0.038 g, 22%). ^1^H NMR showed the presence of two conformers C*a*/*Cb* in a 56/44 ratio. ^1^H NMR (600 MHz, DMSO-*d*_6_): δ 14.31 (s, NH, 1H, C*a* + *Cb*), 11.51 (s, NH, 1.0H, C*a*), 11.48 (s, NH, 1H, *Cb*), 7.93 (s, 1H, C*a* + *Cb*), 7.51–7.46 (m, 1H, C*a* + *Cb*), 7.33–7.36 (s, 1H, C*a*; 2H, *Cb*), 7.21 (s, 1H, C*a*), 4.97 (s, 2H, *Cb*), 4.87 (s, 2H, C*a*), 4.04 (br s, 2H, *Cb*), 3.95 (br s, 2H, C*a*), 3.03 (br s, 2H, C*a*), 2.93 (br s, 1.6H, *Cb*). ^13^C NMR (151 MHz, DMSO-*d*_6_): δ 159.6 (Cq, C*a*), 159.2 (Cq, *Cb*), 140.8 (Cq, C*a*), 137.9 (Cq, *Cb*), 137.5 (Cq, C*a* + *Cb*), 135.2 (Cq, *Cb*), 134.9 (Cq, C*a*) 125.7 (Cq, q, ^1^*J*_CF_ = 271.6 Hz, C*a* + *Cb*), 124.6 (Cq, C*a*), 124.3 (Cq, *Cb*), 121.4 (Cq, q, ^1^*J*_CF_ = 268.2 Hz, C*a* + *Cb*), 119.5 (Cq, q, ^2^*J*_CF_ = 31.2 Hz, C*a*), 119.4 (Cq, q, ^2^*J*_CF_ = 31.7 Hz, *Cb*), 117.2 (CH, q, ^3^*J*_CF_ = 3.3 Hz, C*a* + *Cb*), 115.5 (CH, *Cb*), 115.2 (CH, C*a*), 111.6 (CH, C*a* + *Cb*), 106.4 (Cq, *Cb*), 106.2 (Cq, C*a*), 105.3 (CH, C*a*), 104.3 (CH, *Cb*), 44.4 (CH_2_, C*a*), 44.1 (CH_2_, *Cb*), 40.1 (CH_2_, C*a* + *Cb*), 23.8 (CH_2_, C*a*), 22.7 (CH_2_, *Cb*). ^19^F NMR (565 MHz, DMSO-*d*_6_): δ −57.1 (s, *Cb*), −57.3 (s, C*a*) −59.0 (s, C*a*), −59.1 (s, *Cb*). UPLC-MS: *t*_R_ = 2.26 min; MS (ESI) *m*/*z*: calcd for C_17_H_13_F_6_N_4_O (M + H)^+^, 403.1; found, 403.3. HRMS (AP-ESI) *m*/*z*: calcd for C_17_H_13_F_6_N_4_O [M + H]^+^, 403.0994; found, 403.0986.

#### (8-(Trifluoromethoxy)-1,3,4,5-tetrahydro-2*H*-pyrido[4,3-*b*]indol-2-yl)-(5-(trifluoromethyl)-1*H*-pyrazol-3-yl)methanone (**22**)

Following GP1, the title compound was obtained from 8-(trifluoromethoxy)-2,3,4,5-tetrahydro-1*H*-pyrido[4,3-*b*]indole (**45h**) (0.051 g, 0.20 mmol) and 5-(trifluoromethyl)-1*H*-pyrazole-3-carboxylic acid (**C**) (0.036 g, 0.20 mmol), after purification by silica gel flash column chromatography with DCM/EtOAc (7:3) as the eluent, as a white solid (0.072 g, 88%). ^1^H NMR showed the presence of two conformers C*a*/*Cb* in a 59/41 ratio. ^1^H NMR (600 MHz, DMSO-*d*_6_): δ 14.41 (s, NH, 1H, C*a* + *Cb*), 11.31 (s, NH, 1.0H, C*a*), 11.29 (s, NH, 1H, *Cb*), 7.52 (s, 1H, C*a* + *Cb*), 7.40–7.36 (m, 1H, C*a* + *Cb*), 7.30 (s, 1H, *Cb*), 7.20 (s, 1H, C*a*), 7.03–6.99 (m, 1H, C*a* + *Cb*), 4.91 (s, 2H, *Cb*), 4.81 (s, 2H, C*a*), 4.03 (br s, 2H, *Cb*), 3.94 (br s, 2H, C*a*), 3.01 (br s, 2H, C*a*), 2.91 (br s, 2H, *Cb*). ^13^C NMR (151 MHz, DMSO-*d*_6_): δ 159.5 (Cq, C*a*), 159.2 (Cq, *Cb*), 141.6 (Cq, C*a* + *Cb*), 140.8 (Cq, C*a* + *Cb*), 137.7 (Cq, C*a* + *Cb*), 135.2 (Cq, *Cb*), 135.0 (Cq, C*a*), 134.4 (Cq, C*a* + *Cb*), 125.3 (Cq, C*a*), 125.0 (Cq, *Cb*), 121.4 (Cq, q, ^1^*J*_CF_ = 268.5 Hz, C*a* + *Cb*), 120.5 (Cq, q, ^1^*J*_CF_ = 254.2 Hz, C*a* + *Cb*), 114.2 (CH, C*a* + *Cb*), 111.8 (CH, C*a* + *Cb*), 110.3 (CH, *Cb*), 110.0 (CH, C*a*), 105.9 (Cq, C*a*), 105.7 (Cq, C*a*), 105.2 (CH, C*a*), 104.2 (CH, *Cb*), 44.4 (CH_2_, C*a*), 44.1 (CH_2_, *Cb*), 40.1 (CH_2_, C*a* + *Cb*), 23.9 (CH_2_, C*a*), 22.8 (CH_2_, *Cb*). ^19^F NMR (565 MHz, DMSO-*d*_6_): δ −55.9 (s), −59.1 (s). UPLC-MS: *t*_R_ = 2.33 min; MS (ESI) *m*/*z*: calcd for C_17_H_13_F_6_N_4_O_2_ (M + H)^+^, 419.1; found, 419.2. HRMS (AP-ESI) *m*/*z*: calcd for C_17_H_13_F_6_N_4_O_2_ [M + H]^+^, 419.0943; found, 419.0932.

#### 2-(5-(Trifluoromethyl)-1*H*-pyrazole-3-carbonyl)-2,3,4,5-tetrahydro-1*H*-pyrido[4,3-*b*]indole-8-carbonitrile (**23**)

Following GP1, the title compound was obtained from 2,3,4,5-tetrahydro-1*H*-pyrido[4,3-*b*]indole-8-carbonitrile (**45j**) (0.039 g, 0.20 mmol) and 5-(trifluoromethyl)-1*H*-pyrazole-3-carboxylic acid (**C**) (0.036 g, 0.20 mmol), after purification by preparative HPLC-MS [mobile phase: H_2_O (A) and CH_3_CN (B); linear gradient: 0–0.5 min 10%B; 0.5–7.0 min 100%B], as a white solid (0.012 g, 20%). ^1^H NMR showed the presence of two conformers C*a*/*Cb* in a 56/44 ratio. ^1^H NMR (600 MHz, DMSO-*d*_6_): δ 14.40 (s, NH, 1H, C*a* + *Cb*), 11.65 (s, NH, 1.0H, C*a*), 11.62 (s, NH, 1H, *Cb*), 8.09 (br s, 1H, C*a* + *Cb*), 7.49–7.45 (m, 1H, C*a* + *Cb*), 7.42–7.38 (m, 1H, C*a* + *Cb*), 7.29 (s, 1H, *Cb*), 7.20 (s, 1H, C*a*), 4.94 (s, 2H, *Cb*), 4.84 (s, 2H, C*a*), 4.03 (br s, 2H, *Cb*), 3.95 (br s, 2H, C*a*), 3.02 (br s, 2H, C*a*), 2.92 (br s, 2H, *Cb*). ^13^C NMR (151 MHz, DMSO-*d*_6_): δ 159.6 (Cq, C*a*), 159.3 (Cq, *Cb*), 140.8 (Cq, C*a* + *Cb*), 137.8 (Cq, C*a* + *Cb*), 135.6 (Cq, *Cb*), 135.4 (Cq, C*a*), 125.1 (Cq, C*a*), 124.8 (Cq, *Cb*), 123.8 (CH, C*a* + *Cb*), 123.4 (Cq, C*a* + *Cb*), 123.2 (CH, C*a* + *Cb*), 121.4 (Cq, q, ^1^*J*_CF_ = 268.7 Hz, C*a* + *Cb*), 120.9 (Cq, C*a* + *Cb*), 112.2 (CH, C*a* + *Cb*), 106.4 (Cq, *Cb*), 106.2 (Cq, C*a*), 105.3 (CH, *Cb*), 104.3 (CH, C*a*), 100.7 (Cq, C*a*), 100.5 (Cq, *Cb*), 44.3 (CH_2_, C*a*), 43.9 (CH_2_, *Cb*), 40.1 (CH_2_, C*a* + *Cb*), 23.7 (CH_2_, C*a*), 22.7 (CH_2_, *Cb*). ^19^F NMR (565 MHz, DMSO-*d*_6_): δ −59.1 (s). UPLC-MS: *t*_R_ = 1.92 min; MS (ESI) *m*/*z*: calcd for C_17_H_13_F_3_N_5_O (M + H)^+^, 360.1; found, 360.2. HRMS (AP-ESI) *m*/*z*: calcd for C_17_H_13_F_3_N_5_O [M + H]^+^, 360.1072; found, 360.1063.

#### (8-(Methylsulfonyl)-1,3,4,5-tetrahydro-2*H*-pyrido[4,3-*b*]indol-2-yl)-(5-(trifluoromethyl)-1*H*-pyrazol-3-yl)methanone (**24**)

Following GP2, the title compound was obtained from 8-methylsulfonyl-2,3,4,5-tetrahydro-1*H*-pyrido[4,3-*b*]indole (**45k**) (0.068 g, 0.24 mmol) and 5-(trifluoromethyl)-1*H*-pyrazole-3-carboxylic acid (**C**) (0.043 g, 0.24 mmol), after purification by silica gel flash column chromatography with cyclohexane/EtOAc (50:50) as the eluent, as a white solid in (0.031 g, 31%). ^1^H NMR showed the presence of two conformers C*a*/*Cb* in a 56/44 ratio. ^1^H NMR (600 MHz, DMSO-*d*_6_): δ 14.41 (s, NH, 1H, C*a* + *Cb*), 11.64 (s, NH, 1.0H, C*a*), 11.62 (s, 1H, *Cb*), 8.13 (br s, 1H, C*a* + *Cb*), 7.58 (t, *J* = 8.8 Hz, 1H, C*a* + *Cb*), 7.52 (t, *J* = 9.3 Hz, 1H, C*a* + *Cb*), 7.37 (s, 1H, *Cb*), 7.21 (s, 1H, C*a*), 4.99 (s, 2H, *Cb*), 4.88 (s, 2H, C*a*), 4.04 (br s, 2H, *Cb*), 3.97 (br s, 2H, C*a*), 3.17 (s, 3H, C*a*), 3.12 (2.4, 3H, *Cb*), 3.04 (br s, 2H, C*a*), 2.94 (br s, 2H, *Cb*). ^13^C NMR (151 MHz, DMSO-*d*_6_): δ 159.6 (Cq, C*a*), 159.2 (Cq, *Cb*), 140.8 (Cq, C*a* + *Cb*), 138.1 (Cq, C*a* + *Cb*), 137.6 (Cq, C*a* + *Cb*), 135.7 (Cq, *Cb*), 135.6 (Cq, C*a*), 131.1 (Cq, C*a*), 131.0 (Cq, *Cb*), 124.6 (Cq, C*a*), 124.3 (Cq, *Cb*), 121.4 (Cq, q, ^1^*J*_CF_ = 267.7 Hz, C*a* + *Cb*), 119.2 (CH, *Cb*), 119.1 (CH, C*a*), 118.0 (CH, *Cb*), 117.8 (CH, C*a*), 111.45 (CH, C*a* + *Cb*), 106.9 (Cq, *Cb*), 106.7 (Cq, C*a*), 105.3 (CH, *Cb*), 104.3 (CH, C*a*), 44.6 (CH_3_, *Cb*), 44.5 (CH_3_, C*a*), 44.3 (CH_2_, C*a*), 44.1 (CH_2_, *Cb*), 40.1 (CH_2_, C*a* + *Cb*), 23.8 (CH_2_, C*a*), 22.7 (CH_2_, *Cb*). ^19^F NMR (565 MHz, DMSO-*d*_6_): δ −60.0 (s). UPLC-MS: *t*_R_ = 1.71 min; MS (ESI) *m*/*z*: calcd for C_17_H_16_F_3_N_4_O_3_S (M + H)^+^, 413.1; found, 413.2. HRMS (AP-ESI) *m*/*z*: calcd for C_17_H_16_F_3_N_4_O_3_S [M + H]^+^, 413.0895; found, 413.0896.

#### (6-Fluoro-8-methoxy-1,3,4,5-tetrahydro-2*H*-pyrido[4,3-*b*]indol-2-yl)-(5-(trifluoromethyl)-1*H*-pyrazol-3-yl)methanone (**25**)

Following GP1, the title compound was obtained from 6-fluoro-8-methoxy-2,3,4,5-tetrahydro-1*H*-pyrido [4,3-*b*]indole hydrochloride (**45b**) (0.15 g, 0.58 mmol) and 5-(trifluoromethyl)-1*H*-pyrazole-3-carboxylic acid (**C**) (0.115 g, 0.64 mmol), after purification by preparative HPLC-MS [mobile phase: H_2_O (A) and CH_3_CN (B); linear gradient: 0–0.5 min 10% B; 0.5–7.0 min 100% B], as a white solid (0.056 g, 25%). ^1^H NMR showed the presence of two conformers C*a*/*Cb* in a 62/38 ratio. ^1^H NMR (600 MHz, DMSO-*d*_6_): δ 14.40 (s, NH, 1H, C*a* + *Cb*), 11.27 (s, NH, 1H, C*a* + *Cb*), 7.27 (br s, 1H, *Cb*), 7.20 (br s, 1H, C*a*), 6.88 (br s, 1H, C*a*), 6.82 (br s, 1H, *Cb*), 6.58 (d, *J* = 12.3, 1H, C*a* + *Cb*), 4.85 (s, 2H, *Cb*), 4.77 (s, 2H, C*a*), 4.01 (br s, 2H, *Cb*), 3.92 (t, *J* = 5.6 Hz, 2H, C*a*), 3.77 (s, 3H, C*a*), 3.74 (1.8, 3H, *Cb*), 2.97 (t, *J* = 5.5 Hz, 2H, C*a*), 2.87 (t, *J* = 5.5 Hz, 2H, *Cb*). ^13^C NMR (151 MHz, DMSO-*d*_6_): δ 159.6 (Cq, C*a*), 159.3 (Cq, *Cb*), 153.3 (Cq, d, ^3^*J*_CF_ = 9.0 Hz, C*a* + *Cb*), 149.2 (Cq, d, *J* = 242.0 Hz, C*a* + *Cb*), 140.7 (Cq, C*a* + *Cb*), 137.9 (Cq, C*a* + *Cb*), 134.6 (Cq, *Cb*), 134.2 (Cq, C*a*), 128.5 (Cq, d, ^3^*J*_CF_ = 7.7 Hz, C*a*), 128.1 (Cq, d, ^3^*J*_CF_ = 7.2 Hz, *Cb*), 121.3 (Cq, q, ^1^*J*_CF_ = 268.0 Hz, C*a* + *Cb*), 118.5 (Cq, d, ^2^*J*_CF_ = 13.2 Hz, C*a* + *Cb*), 106.1 (Cq, *Cb*), 105.9 (Cq, C*a*), 105.1 (CH, *Cb*), 104.3 (CH, C*a*), 96.6 (CH, d, ^2^*J*_CF_ = 19.6 Hz, C*a* + *Cb*), 96.1 (CH, *Cb*), 95.8 (CH, C*a*), 55.8 (CH_3_, *Cb*), 55.7 (CH_3_, C*a*), 44.4 (CH_2_, C*a*), 44.3 (CH_2_, *Cb*), 40.2 (CH_2_, *Cb*), (40.1, CH_2_, *Ca*), 23.9 (CH_2_, C*a*), 22.8 (CH_2_, *Cb*). ^19^F NMR (565 MHz, DMSO-*d*_6_): δ −60.1 (s), −132.0 (s). UPLC-MS: *t*_R_ = 1.86 min; MS (ESI) *m*/*z*: calcd for C_17_H_15_F_4_N_4_O_2_ (M + H)^+^, 383.1; found, 383.2. HRMS (AP-ESI) *m*/*z*: calcd for C_17_H_15_F_4_N_4_O_2_ [M + H]^+^, 383.1131; found, 383.1129.

#### (6-Fluoro-8-methyl-1,3,4,5-tetrahydro-2*H*-pyrido[4,3-*b*]indol-2-yl)-(5-(trifluoromethyl)-1*H*-pyrazol-3-yl)methanone (**26**)

Following GP2, the title compound was obtained from 6-fluoro-8-methyl-2,3,4,5-tetrahydro-1*H*-pyrido [4,3-*b*]indole hydrochloride (**45i**) (0.147 g, 0.61 mmol) and 5-(trifluoromethyl)-1*H*-pyrazole-3-carboxylic acid (**C**) (0.11 g, 0.61 mmol), after purification by preparative HPLC-MS [mobile phase: H_2_O (A) and CH_3_CN (B); linear gradient: 0–0.5 min 50% B; 0.5–7.0 min 100% B], as a white solid (0.015 g, 6%). ^1^H NMR showed the presence of two conformers C*a*/*Cb* in a 56/44 ratio. ^1^H NMR (400 MHz, DMSO-*d*_6_): δ 14.39 (s, NH, 1H, C*a* + *Cb*), 11.29 (s, NH, 1H, C*a* + *Cb*), 7.27 (br s, 1H, *Cb*), 7.19 (br s, 1H, C*a*), 7.07 (m, 1H, C*a* + *Cb*), 6.74 (d, *J* = 12.3 Hz, 1H, C*a* + *Cb*), 4.85 (s, 2H, *Cb*), 4.77 (br s, 2H, C*a*), 4.02 (br s, 2H, *Cb*), 3.92 (br s, 2H, C*a*), 2.96 (br s, 2H, C*a*), 2.88 (br s, 2H, *Cb*), 2.35 (s, 3H, C*a* + *Cb*). ^13^C NMR (151 MHz, DMSO-*d*_6_) for major C*a*: δ 159.4 (Cq), 148.4 (Cq, d, ^1^*J*_CF_ = 242.7 Hz), 140.9 (Cq, d, ^2^*J*_CF_ = 37.8 Hz), 137.6 (Cq), 133.7 (Cq), 129.1 (Cq), 128.4 (Cq), 121.6 (Cq, d, ^3^*J*_CF_ = 12.1 Hz), 121.5 (Cq, q, ^1^*J*_CF_ = 268.4 Hz, C*a* + *Cb*), 113.2 (CH), 107.2 (CH, d, ^2^*J*_CF_ = 16.2 Hz), 105.4 (Cq), 104.1 (CH), 44.4 (CH_2_), 40.4 (CH_2_), 23.8 (CH_2_), 21.0 (CH_3_). ^19^F NMR (565 MHz, DMSO-*d*_6_): δ −60.1 (s), −134.2 (s). UPLC-MS: *t*_R_ = 2.24 min; MS (ESI) *m*/*z*: calcd for C_17_H_15_F_4_N_4_O (M + H)^+^, 367.1; found, 367.2. HRMS (AP-ESI) *m*/*z*: calcd for C_17_H_15_F_4_N_4_O [M + H]^+^, 367.1182; found, 367.1170.

#### (6,8-Difluoro-1,3,4,5-tetrahydro-2*H*-pyrido[4,3-*b*]indol-2-yl)-(5-(trifluoromethyl)-1*H*-pyrazol-3-yl)methanone (**27**)

Following GP2, the title compound was obtained from 6,8-difluoro-2,3,4,5-tetrahydro-1*H*-pyrido[4,3-*b*]indole hydrochloride (**45l**) (0.1 g, 0.41 mmol) and 5-(trifluoromethyl)-1*H*-pyrazole-3-carboxylic acid (**C**) (0.074 g, 0.41 mmol), after purification by preparative HPLC-MS [mobile phase: H_2_O (A) and CH_3_CN (B); linear gradient: 0–0.5 min 10% B; 0.5–7.0 min 100% B], as a white solid (0.049 g, 32%). ^1^H NMR showed the presence of two conformers C*a*/*Cb* in a 57/43 ratio. ^1^H NMR (600 MHz, DMSO-*d*_6_): δ 14.27 (s, NH, 1H, C*a* + *Cb*), 11.59 (s, NH, 1H, C*a* + *Cb*), 7.27–7.20 (m, 1H, C*a*; 2H, *Cb*), 7.19 (br s, 1H, C*a*), 6.93–6.87 (m, 1H, C*a* + *Cb*), 4.87 (s, 2H, *Cb*), 4.77 (br s, 2H, C*a*), 4.02 (br s, 2H, *Cb*), 3.94 (br s, 2H, C*a*), 3.00 (br s, 2H, C*a*), 2.88 (br s, 1.5H, *Cb*). ^13^C NMR (151 MHz, DMSO-*d*_6_): δ 159.6 (Cq, C*a*), 159.2 (Cq, *Cb*), 155.7 (Cq, dd, ^1,2^*J*_CF_ = 233.7, 9.8 Hz, C*a* + *Cb*), 147.7 (Cq, d, ^1,2^*J*_CF_ = 245.1, 9.6 Hz, C*a* + *Cb*), 140.5 (Cq, C*a* + *Cb*), 138.0 (Cq, C*a* + *Cb*), 135.9 (Cq, *Cb*), 135.7 (Cq, C*a*), 127.8 (Cq, C*a*), 127.5 (Cq, *Cb*), 121.4 (Cq, q, ^1^*J*_CF_ = 268.8 Hz, C*a* + *Cb*), 120.2 (Cq, d, ^2^*J*_CF_ = 12.8 Hz, C*a* + *Cb*), 106.8 (Cq, *Cb*), 106.5 (Cq, C*a*), 105.2 (CH, *Cb*), 104.3 (CH, C*a*), 99.1 (CH, t, ^2^*J*_CF_ = 26.3 Hz, C*a* + *Cb*), 95.9 (CH, dd, ^2^*J*_CF_ = 21.0 Hz, *Cb*), 95.9 (CH, dd, ^2^*J*_CF_ = 21.0 Hz, C*a*), 44.3 (CH_2_, C*a*), 44.0 (CH_2_, *Cb*), 40.0 (CH_2_, C*a* + *Cb*), 23.9 (CH_2_, C*a*), 22.8 (CH_2_, *Cb*). ^19^F NMR (565 MHz, DMSO-*d*_6_): δ −59.0 (s, C*a* + *Cb*), −121.4 (s, C*a*), −121.7 (s, *Cb*) −129.3 (s, C*a* + *Cb*). UPLC-MS: *t*_R_ = 2.23 min; MS (ESI) *m*/*z*: calcd for C_16_H_12_F_5_N_4_O (M + H)^+^, 371.1; found, 371.2. HRMS (AP-ESI) *m*/*z*: calcd for C_16_H_12_F_5_N_4_O [M + H]^+^, 371.0931; found, 371.0931.

#### (6-Fluoro-8-trifluoromethyl-1,3,4,5-tetrahydro-2*H*-pyrido[4,3-*b*]indol-2-yl)-(5-(trifluoromethyl)-1*H*-pyrazol-3-yl)methanone (**28**)

Following GP2, the title compound was obtained from 6-fluoro-8-(trifluoromethyl)-2,3,4,5-tetrahydro-1*H*-pyrido[4,3-*b*]indole hydrochloride (**45m**) (0.15 g, 0.51 mmol) and 5-(trifluoromethyl)-1*H*-pyrazole-3-carboxylic acid (**C**) (0.101 g, 0.56 mmol), after purification by silica gel flash column chromatography with cyclohexane/EtOAc (0 to 50%) as the eluent, as a white solid (0.012 g, 6%). ^1^H NMR showed the presence of two conformers C*a*/*Cb* in a 57/43 ratio. ^1^H NMR (600 MHz, DMSO-*d*_6_): δ 14.27 (s, NH, 1H, C*a* + *Cb*), 12.03 (s, NH, 1H, C*a* + *Cb*), 7.85 (br s, 1H, C*a* + *Cb*), 7.27–7.22 (m, 2H, C*a* + *Cb*), 4.96 (s, 2H, *Cb*), 4.87 (br s, 2H, C*a*), 4.03 (br s, 2H, *Cb*), 3.94 (br s, 2H, C*a*), 3.04 (br s, 2H, C*a*), 2.93 (br s, 2H, *Cb*). ^13^C NMR (151 MHz, DMSO-*d*_6_) for the major C*a*: δ 159.5 (Cq), 148.2 (Cq, d, ^1^*J*_CF_ = 244.0 Hz), 141.0 (Cq, d, ^2^*J*_CF_ = 37.1 Hz), 137.5 (Cq), 136.3 (Cq), 128.4 (Cq, d, ^3^*J*_CF_ = 7.5 Hz), 124.8 (Cq, q, ^1^*J*_CF_ = 271.6 Hz), 125.0 (Cq, d, ^3^*J*_CF_ = 12.6), 121.4 (Cq, q, ^1^*J*_CF_ = 268.0 Hz), 112.2 (CH), 107.5 (Cq), 105.2 (Cq), 104.0 (CH), 102.8 (d, ^2^*J*_CF_ = 20.2 Hz), 44.3 (CH_2_), 40.1 (CH_2_), 23.8 (CH_2_). ^19^F NMR (565 MHz, DMSO-*d*_6_): δ −56.4, −58.0, −129.9. UPLC-MS: *t*_R_ = 2.06 min; MS (ESI) *m*/*z*: calcd for C_17_H_12_F_7_N_4_O (M + H)^+^, 421.1; found, 421.5. HRMS (AP-ESI) *m*/*z*: calcd for C_17_H_12_F_7_N_4_O [M + H]^+^, 421.0899; found, 421.0901.

#### (6,9-Difluoro-1,3,4,5-tetrahydro-2*H*-pyrido[4,3-*b*]indol-2-yl)-(5-(trifluoromethyl)-1*H*-pyrazol-3-yl)methanone (**29**)

Following GP1, the title compound was obtained from 6,9-difluoro-2,3,4,5-tetrahydro-1*H*-pyrido[4,3-*b*]indole hydrochloride (**45n**) (0.1 g, 0.41 mmol) and 5-(trifluoromethyl)-1*H*-pyrazole-3-carboxylic acid (**C**) (0.081 g, 0.45 mmol), after purification by silica gel flash column chromatography with cyclohexane/EtOAc (0 to 30%) as the eluent, as a white solid (0.02 g, 13%). ^1^H NMR showed the presence of two conformers C*a*/*Cb* in a 67/33 ratio. ^1^H NMR (400 MHz, DMSO-*d*_6_): δ 14.42 (s, NH, 1H, C*a* + *Cb*), 11.80 (s, NH, 1H, C*a* + *Cb*), 7.21 (br s, 1H, C*a* + *Cb*), 6.87–6.82 (br s, 1H, C*a* + *Cb*), 6.72–6.67 (m, 1H, C*a* + *Cb*), 4.96 (s, 2H, *Cb*), 4.88 (br s, 2H, C*a*), 4.02 (br s, 2H, *Cb*), 3.94 (br s, 2H, C*a*), 3.00 (br s, 2H, C*a*), 2.90 (br s, 2H, *Cb*). ^13^C NMR (151 MHz, DMSO-*d*_6_) for the major C*a*: δ 159.5 (Cq), 151.3 (Cq, d, ^1^*J*_CF_ = 236.7 Hz), 145.3 (Cq, d, ^1^*J*_CF_ = 238.2 Hz), 140.8 (Cq), 137.8 (Cq), 134.1 (Cq), 125.5 (Cq), 120.6 (Cq), 116.7 (Cq), 105.6 (CH, dd, ^2,3^*J*_CF_ = 19.8, 8.8 Hz), 104.6 (Cq), 104.0 (CH), 103.3 (CH, dd, ^2,3^*J*_CF_ = 21.7, 7.3 Hz), 44.1 (CH_2_), 40.1 (CH_2_), 23.7 (CH_2_). ^19^F NMR (565 MHz, DMSO-*d*_6_): δ – 68.7, −128.6, −136.5. UPLC-MS: *t*_R_ = 2.32 min; MS (ESI) *m*/*z*: calcd for C_16_H_12_F_5_N_4_O (M + H)^+^, 371.1; found, 371.3. HRMS (AP-ESI) *m*/*z*: calcd for C_16_H_12_F_5_N_4_O [M + H]^+^, 371.0931; found, 371.0921.

#### (6-Fluoro-9-methyl-1,3,4,5-tetrahydro-2*H*-pyrido[4,3-*b*]indol-2-yl)-(5-(trifluoromethyl)-1*H*-pyrazol-3-yl)methanone (**30**)

Following GP2, the title compound was obtained from 6-fluoro-9-methyl-2,3,4,5-tetrahydro-1*H*-pyrido [4,3-*b*]indole hydrochloride (**45a**) (0.1 g, 0.42 mmol) and 5-(trifluoromethyl)-1*H*-pyrazole-3-carboxylic acid (**C**) (0.076 g, 0.42 mmol), after purification by silica gel flash column chromatography with DCM/EtOAc (80:20) as the eluent, as a white solid (0.031 g, 20%). ^1^H NMR showed the presence of two conformers C*a*/*Cb* in a 62/38 ratio. ^1^H NMR (600 MHz, DMSO-*d*_6_): δ 14.43 (s, NH, 1H, C*a* + *Cb*), 11.40 (s, NH, 1H, C*a* + *Cb*), 7.20 (br s, 1H, C*a* + *Cb*), 6.75–6.61 (m, 2H, C*a* + *Cb*), 5.12 (s, 2H, *Cb*), 5.04 (br s, 2H, C*a*), 4.00 (br s, 2H, *Cb*), 3.90 (br s, 2H, C*a*), 2.97 (br s, 2H, C*a*), 2.88 (br s, 2H, *Cb*), 2.54 (s, 3H, C*a*), 2.40 (s, 3H, *Cb*). ^13^C NMR (151 MHz, DMSO-*d*_6_): δ 159.6 (Cq, C*a*), 159.3 (Cq, *Cb*), 147.6 (Cq, d, ^1^*J*_CF_ = 239.8 Hz, C*a* + *Cb*), 140.8 (Cq, C*a* + *Cb*), 137.8 (Cq, C*a* + *Cb*), 133.2 (Cq, *Cb*), 133.1 (Cq, C*a*), 128.1 (Cq, C*a*), 127.8 (Cq, *Cb*), 124.5 (Cq, C*a*), 124.3 (Cq, *Cb*), 123.4 (Cq, d, ^3^*J*_CF_ = 13.8 Hz, C*a* + *Cb*), 121.4 (Cq, q, ^1^*J*_CF_ = 268.4 Hz, C*a* + *Cb*), 119.4 (CH, d, ^3^*J*_CF_ = 6.3 Hz, C*a* + *Cb*), 106.8 (Cq, *Cb*), 106.4 (Cq, C*a*), 105.4 (CH, d, ^2^*J*_CF_ = 16.4 Hz, C*a* + *Cb*), 104.8 (CH, *Cb*), 104.3 (CH, C*a*), 45.4 (CH_2_, *Cb*), 44.0 (CH_2_, C*a*), 41.2 (CH_2_, *Cb*), 40.1 (CH_2_, C*a*), 23.9 (CH_2_, C*a*), 22.8 (CH_2_, *Cb*), 18.9 (CH_3_, C*a*), 18.6 (CH_3_, *Cb*). ^19^F NMR (565 MHz, DMSO-*d*_6_): δ −59.1, −136.9. UPLC-MS: *t*_R_ = 2.22 min; MS (ESI) *m*/*z*: calcd for C_17_H_15_F_4_N_4_O (M + H)^+^, 367.2; found, 367.3. HRMS (AP-ESI) *m*/*z*: calcd for C_17_H_15_F_4_N_4_O [M + H]^+^, 367.1182; found, 367.1168.

#### (9-Fluoro-6-methyl-1,3,4,5-tetrahydro-2*H*-pyrido[4,3-*b*]indol-2-yl)(5-(trifluoromethyl)-1*H*-pyrazol-3-yl)methanone (**31**)

Following GP2, the title compound was obtained from 9-fluoro-6-methyl-2,3,4,5-tetrahydro-1*H*-pyrido [4,3-*b*]indole hydrochloride (**45o**) (0.09 g, 0.37 mmol) and 5-(trifluoromethyl)-1*H*-pyrazole-3-carboxylic acid (**C**) (0.067 g, 0.37 mmol), after purification by silica gel flash column chromatography with cyclohexane/EtOAc (0 to 80%) as the eluent, as a white solid (0.041 g, 13%). ^1^H NMR showed the presence of two conformers C*a*/*Cb* in a 64/36 ratio. ^1^H NMR (600 MHz, DMSO-*d*_6_): δ 14.42 (s, NH, 1H, C*a* + *Cb*), 11.21 (s, NH, 1H, C*a* + *Cb*), 7.21 (br s, 1H, C*a* + *Cb*), 7.17 (br s, 1H, *Cb*), 6.80–6.76 (m, 1H, C*a* + *Cb*), 6.65 (t, *J* = 8.5 Hz, 1H, C*a*), 6.60 (t, *J* = 8.8 Hz, 1H, *Cb*), 4.96 (s, 2H, *Cb*), 4.89 (br s, 2H, C*a*), 4.02 (br s, 2H, *Cb*), 3.93 (br s, 2H, C*a*), 3.00 (br s, 2H, C*a*), 2.91 (br s, 2H, *Cb*), 2.39 (s, 3H, C*a* + *Cb*). ^13^C NMR (151 MHz, DMSO-*d*_6_): δ 159.6 (Cq, C*a* + *Cb*), 154.0 (Cq, d, ^1^*J*_CF_ = 239.1 Hz, C*a*), 153.8 (Cq, d, ^1^*J*_CF_ = 239.8 Hz, *Cb*), 140.8 (Cq, *Cb*), 137.8 (Cq, *Cb*), 137.7 (Cq, d, ^3^*J*_CF_ = 11.3 Hz, C*a* + *Cb*), 132.7 (Cq, *Cb*), 132.5 (Cq, C*a*), 121.4 (Cq, q, ^1^*J*_CF_ = 268.2 Hz, C*a* + *Cb*), 121.3 (CH, d, ^3^*J*_CF_ = 7.3 Hz, C*a* + *Cb*), 116.6 (Cq, d, ^4^*J*_CF_ = 2.9 Hz, C*a* + *Cb*), 113.2 (Cq, d, ^2^*J*_CF_ = 21.4 Hz, C*a*), 112.9 (Cq, d, ^2^*J*_CF_ = 21.3 Hz, *Cb*), 104.6 (CH, *Cb*), 104.2 (CH, C*a*), 103.5 (CH, d, ^2^*J*_CF_ = 18.2 Hz, C*a* + *Cb*), 103.2 (Cq, C*a* + *Cb*), 44.9 (CH_2_, *Cb*), 44.2 (CH_2_, C*a*), 40.7 (CH_2_, *Cb*), 40.1 (CH_2_, C*a*), 23.7 (CH_2_, C*a*), 22.6 (CH_2_, *Cb*), 16.2 (CH_3_, C*a* + *Cb*). ^19^F NMR (565 MHz, DMSO-*d*_6_): δ −60.1, −129.5. UPLC-MS: *t*_R_ = 2.21 min; MS (ESI) *m*/*z*: calcd for C_17_H_15_F_4_N_4_O (M + H)^+^, 367.1; found, 367.2. HRMS (AP-ESI) *m*/*z*: calcd for C_17_H_15_F_4_N_4_O [M + H]^+^, 367.1182; found, 367.1171.

#### (±)-(6-Fluoro-1,9-dimethyl-1,3,4,5-tetrahydro-2*H*-pyrido[4,3-*b*]indol-2-yl)-(5-(trifluoromethyl)-1*H*-pyrazol-3-yl)methanone (**32**)

Following GP1, the title compound was obtained from racemic 6-fluoro-1,9-dimethyl-2,3,4,5-tetrahydro-1*H*-pyrido[4,3-*b*]indole (**52**) (0.09 g, 0.35 mmol) and 5-(trifluoromethyl)-1*H*-pyrazole-3-carboxylic acid (**C**) (0.062 g, 035 mmol), after purification by silica gel flash column chromatography with cyclohexane/EtOAc (0 to 50%) as the eluent, as a white solid (0.06 g, 45%). ^1^H NMR showed the presence of two conformers C*a*/*Cb* in a 77/33 ratio. ^1^H NMR (600 MHz, DMSO-*d*_6_): δ 14.33 (br s, NH, 1H, C*a* + *Cb*), 11.42 (br s, 1H, C*a*), 11.40 (br s, NH, 1H, *Cb*), 7.18 (br s, 1H, C*a*), 7.07 (br s, 1H, *Cb*), 6.76 (dd, *J* = 11.1, 7.9 Hz, 1H, C*a*), 6.73 (dd, *J* = 11.1, 8.2 Hz, 1H, *Cb*), 6.69 (dd, *J* = 8.0, 4.8 Hz, 1H, C*a*), 6.63 (dd, *J* = 7.6, 4.7 Hz, 1H, *Cb*), 5.99 (q, *J* = 6.6 Hz, 1H, C*a*), 5.57 (br s, 1H, *Cb*), 4.69 (dd, *J* = 13.2, 5.6 Hz, 1H, *Cb*), 4.15 (dd, *J* = 13.7, 5.4 Hz, 1H, C*a*), 3.71 (ddd, *J* = 14.1, 11.9, 4.4 Hz, 1H, C*a*), 3.47 (td, *J* = 12.4, 5.3 Hz, 1H, *Cb*), 3.12 (ddd, *J* = 17.1, 11.9, 6.0 Hz, 1H, C*a*), 2.84–2.93 (m, 1H, *Cb*), 2.82 (dd, *J* = 16.5, 4.2 Hz, 1H, C*a*), 2.57 (s, 3H, C*a*), 2.35 (s, 3H, *Cb*), 1.65 (d, *J* = 6.5 Hz, 3H, *Cb*), 1.56 (d, *J* = 6.5 Hz, 3H, C*a*). ^13^C NMR (151 MHz, DMSO-*d*_6_): δ 159.3 (Cq, *Cb*), 158.8 (Cq, C*a*), 147.6 (Cq, d, ^1^*J*_CF_ = 239.4 Hz, C*a* + *Cb*), 140.8 (Cq, *Cb*), 138.0 (Cq, *Cb*), 132.7 (Cq, *Cb*), 132.5 (Cq, C*a*), 127.4 (d, ^4^*J*_CF_ = 5.5 Hz, C*a*), 126.9 (d, ^4^*J*_CF_ = 5.2 Hz, *Cb*), 124.0 (Cq, C*a* + *Cb*), 123.8 (Cq, d, ^4^*J*_CF_ = 2.3 Hz, C*a* + *Cb*), 123.5 (Cq, q, ^2^*J*_CF_ = 14.4 Hz, C*a* + *Cb*), 121.4 (Cq, q, ^1^*J*_CF_ = 267.7 Hz, C*a* + *Cb*), 120.0 (CH, d, ^3^*J*_CF_ = 7.4 Hz, *Cb*), 119.9 (CH, d, ^3^*J*_CF_ = 6.1 Hz, C*a*), 112.2 (Cq, *Cb*), 111.8 (Cq, C*a*), 105.4 (CH, d, ^2^*J*_CF_ = 6.1 Hz, C*a* + *Cb*), 104.1 (CH, C*a*), 103.4 (CH, *Cb*), 50.0 (CH, *Cb*), 45.3 (CH, C*a*), 40.1 (CH_2_, C*a*), 34.1 (CH_2_, *Cb*), 23.6 (CH_2_, C*a*), 23.1 (CH_3_, *Cb*), 22.5 (CH_2_, *Cb*), 21.6 (CH_3_, C*a*), 19.3 (CH_3_, C*a*), 18.80 (CH_3_, *Cb*). ^19^F NMR (376 MHz, DMSO-*d*_6_): δ −59.0, −137.0. UPLC-MS: *t*_R_ = 2.58 min; MS (ESI) *m*/*z*: calcd for C_18_H_17_F_4_N_4_ (M + H)^+^, 381.1; found, 381.3. HRMS (AP-ESI) *m*/*z*: calcd for C_18_H_17_F_4_N_4_ [M + H]^+^, 381.1338; found, 381.1333.

#### (±)-(6-Fluoro-3,9-dimethyl-1,3,4,5-tetrahydro-2*H*-pyrido[4,3-*b*]indol-2-yl)-(5-(trifluoromethyl)-1*H*-pyrazol-3-yl)methanone (**33**)

Following GP1, the title compound was obtained from racemic 6-fluoro-3,9-dimethyl-2,3,4,5-tetrahydro-1*H*-pyrido[4,3-*b*]indole (**53**) (0.07 g, 0.27 mmol) and 5-(trifluoromethyl)-1*H*-pyrazole-3-carboxylic acid (**C**) (0.048 g, 0.27 mmol), after purification by silica gel flash column chromatography with cyclohexane/EtOAc (0 to 50%) as the eluent, as a white solid (0.049 g, 47%). ^1^H NMR (400 MHz, DMSO-*d*_6_): δ 14.37 (br s, NH), 11.36 (br s, NH), 7.18 (br s, 1H), 6.73 (dd, *J* = 11.2, 8.0 Hz, 1H), 6.66 (m, 1H), 4.48–5.57 (m, 3H), 3.13 (m, 1H), 2.65 (m, 1H), 2.56 (s, 3H), 2.34 (m, 1H), 1.26 (d, *J* = 6.8 Hz, 3H). ^13^C NMR (151 MHz, DMSO-*d*_6_): δ 159.5 (Cq), 147.5 (Cq, d, ^1^*J*_CF_ = 239.2 Hz), 140.5 (Cq), 138.6 (Cq), 127.8 (Cq), 124.4 (Cq), 123.5 (Cq, d, ^1^*J*_CF_ = 13.7 Hz), 121.4 (Cq, q, ^1^*J*_CF_ = 268.4 Hz), 119.3 (CH, d, *J* = 6.1 Hz), 118.7 (Cq), 105.2 (CH, d, ^2^*J*_CF_ = 15.9 Hz), 104.8 (Cq), 103.8 (CH), 48.1 (CH), 37.2 (CH_2_), 28.9 (CH_2_), 18.7 (CH_3_), 18.1 (CH_3_). ^19^F NMR (565 MHz, DMSO-*d*_6_): δ −60.0, −138.0. UPLC-MS: *t*_R_ = 2.58 min; MS (ESI) *m*/*z*: calcd for C_18_H_17_F_4_N_4_O (M + H)^+^, 381.1; found, 381.3. HRMS (AP-ESI) *m*/*z*: calcd for C_18_H_17_F_4_N_4_O_4_ [M + H]^+^, 381.1338; found 381.1333.

#### (6-Fluoro-3,3,9-trimethyl-1,3,4,5-tetrahydro-2*H*-pyrido[4,3-*b*]indol-2-yl)-(5-(trifluoromethyl)-1*H*-pyrazol-3-yl)methanone (**34**)

Following GP1, the title compound was obtained from 6-fluoro-3,3,9-trimethyl-2,3,4,5-tetrahydro-1*H*-pyrido[4,3-*b*]indole (**57**) (0.045 g, 0.19 mmol) and 5-(trifluoromethyl)-1*H*-pyrazole-3-carboxylic acid (**C**) (0.08 g, 0.44 mmol), after purification by silica gel flash column chromatography with cyclohexane/EtOAc (0 to 50%) as the eluent, as a white solid (0.016 g, 20%). ^1^H NMR (400 MHz, DMSO-*d*_6_): δ 14.29 (br s, 1H), 11.38 (br s, 1H), 7.14 (s, 1H), 6.70 (dd, *J* = 11.3, 7.8 Hz, 1H), 6.59 (ddd, *J* = 7.8, 4.7, 0.6 Hz, 1H), 5.0 (s, 2H), 2.96 (s, 2H), 2.30 (s, 3H), 1.59 (s, 6H). ^13^C NMR (151 MHz, DMSO-*d*_6_): δ 162.0 (Cq), 147.6 (Cq, d, ^1^*J*_CF_ = 239.9 Hz), 140.1 (Cq), 133.6 (Cq), 127.5 (Cq, d, ^3^*J*_CF_ = 5.5 Hz), 124.1 (Cq, ^4^*J*_CF_ = 2.8 Hz), 123.1 (Cq, d, ^2^*J*_CF_ = 13.7 Hz), 120.5 (Cq), 119.4 (CH, d, ^3^*J*_CF_ = 6.3 Hz), 106.6 (Cq), 105.2 (CH, d, ^2^*J*_CF_ = 15.4 Hz), 104.6 (CH), 55.8 (Cq), 45.0 (CH_2_), 35.3 (CH_2_), 26.9 (CH_3_), 18.6 (CH_3_). ^19^F NMR (565 MHz, DMSO-*d*_6_): δ −58.3, −135.8. UPLC-MS: *t*_R_ = 2.45 min; MS (ESI) *m*/*z*: calcd for C_19_H_19_F_4_N_4_O (M + H)^+^, 394.4; found, 394.5. HRMS (AP-ESI) *m*/*z*: calcd for C_19_H_19_F_4_N_4_O [M + H]^+^, 395.1495; found, 395.1481.

#### (±)-(4-Fluoro-1-methyl-5,6,7,8,9,10-hexahydro-7,10-epiminocyclohepta[*b*]indol-11-yl)-(5-(trifluoromethyl)-1*H*-pyrazol-3-yl)methanone (**35**)

Following GP1, the title compound was obtained from racemic 4-fluoro-1-methyl-5,6,7,8,9,10-hexahydro-7,10-epiminocyclohepta[*b*]indole hydrochloride (**59**) (0.2 g, 0.75 mmol) and 5-(trifluoromethyl)-1*H*-pyrazole-3-carboxylic acid (**C**) (0.135 g, 0.75 mmol), after purification by silica gel flash column chromatography with cyclohexane/EtOAc (0 to 80%) as the eluent, as a white solid (0.056 g, 5%). ^1^H NMR showed the presence of two conformers C*a*/*Cb* in a 56/44 ratio. ^1^H NMR (600 MHz, DMSO-*d*_6_): δ 14.39 (s, 1.6H, C*a* + *Cb*), 11.43 (br s, NH, C*a*), 11.40 (s, 1H, *Cb*), 7.30 (s, 1H, *Cb*), 7.04 (s, 1H, C*a*), 6.73–6.64 (m, 2H, C*a* + *Cb*), 5.92 (d, *J* = 5.5 Hz, 1H, C*a*), 5.76 (br s, 1H, *Cb*), 4.99 (dd, *J* = 7.5, 5.5 Hz, 1H, C*a*), 4.95 (s, 1H, *Cb*), 3.42 (dd, *J* = 16.3, 4.4 Hz, 1H, C*a*), 3.30 (m, 1H, *Cb*), 2.74 (d, *J* = 16.5 Hz, 1H, *Cb*), 2.70 (d, *J* = 16.4 Hz, 1H, C*a*), 2.56 (br s, 3H, *Cb*), 2.43 (br s, 3H, C*a*), 2.39–2.36 (m, 1H, C*a*), 2.32–2.27 (m, 1H, *Cb*), 2.06–2.08 (m, 1H, C*a* + *Cb*), 1.78–1.72 (m, 1H, C*a* + *Cb*). ^13^C NMR (151 MHz, DMSO-*d*_6_): δ 156.4 (Cq, C*a*), 155.8 (Cq, *Cb*), 147.7 (Cq, d, ^1^*J*_CF_ = 239.7 Hz, C*a* + *Cb*), 141.1 (Cq, *Cb*), 138.2 (Cq, C*a*), 132.3 (Cq, *Cb*), 131.8 (Cq, C*a*), 126.8 (Cq, d, ^3^*J*_CF_ = 5.5 Hz, C*a*), 126.2 (Cq, d, ^3^*J*_CF_ = 5.4 Hz, *Cb*), 123.9 (Cq, C*a*), 123.5 (Cq, C*a* + *Cb*), 123.0 (Cq, d, ^2^*J*_CF_ = 10.9 Hz, C*a*), 122.9 (Cq, d, ^2^*J*_CF_ = 10.9 Hz, C*a*), 121.4 (Cq, q, ^1^*J*_CF_ = 269.5 Hz, C*a* + *Cb*), 119.7 (Cq, d, ^4^*J*_CF_ = 5.7 Hz, C*a*), 119.5 (CH, d, ^2^*J*_CF_ = 28.5 Hz, C*a* + *Cb*), 115.5 (Cq, *Cb*), 115.1 (Cq, C*a*), 105.00 (CH, d, ^2^*J*_CF_ = 15.7 Hz, C*a*), 104.9 (CH, d, ^2^*J*_CF_ = 15.6 Hz, *Cb*), 104.4 (CH, *Cb*), 104.1 (CH, C*a*), 55.3 (CH, *Cb*), 53.5 (CH, *Cb*), 51.5 (CH, C*a*), 51.1 (CH, C*a*), 36.6 (CH_2_, C*a*), 34.8 (CH_2_, *Cb*), 33.7 (CH_2_, *Cb*), 31.6 (CH_2_, C*a*), 29.6 (CH_2_, C*a*), 27.3 (CH_2_, *Cb*), 19.3 (CH_3_, *Cb*), 18.9 (CH_3_, *Cb*). ^19^F NMR (376 MHz, DMSO-*d*_6_): δ −59.1, −136.7. UPLC-MS: *t*_R_ = 2.29 min; MS (ESI) *m*/*z*: calcd for C_19_H_17_F_4_N_4_O (M + H)^+^, 393.1; found, 393.2. HRMS (AP-ESI) *m*/*z*: calcd for C_19_H_17_F_4_N_4_O [M + H]^+^, 393.1338; found, 393.1340.

#### (*R*)- or (*S*)-(4-Fluoro-1-methyl-5,6,7,8,9,10-hexahydro-7,10-epiminocyclohepta[*b*]indol-11-yl)(5-(trifluoromethyl)-1*H*-pyrazol-3-yl)methanone (**36**)

The title compound was obtained after semipreparative chiral separation of racemic (4-fluoro-1-methyl-5,6,7,8,9,10-hexahydro-7,10-epiminocyclohepta[*b*]indol-11-yl)-(5-(trifluoromethyl)-1*H*-pyrazol-3-yl)methanone (**35**) on a Daicel ChiralPak AD column (250 × 10 mm ID, particle size 10 μm) using heptane–EtOH (75:25) as the mobile phase, as a white solid (0.023 g, 28%). NMR spectra, UPLC-MS data, and HRMS data are consistent with the corresponding racemate **35**. Enantiomeric excess was determined to be >99% after chiral HPLC analysis. [α]_589_^25^ +78.2 (*c* 0.5, CH_3_OH).

#### (*S*)- or (*R*)-(4-Fluoro-1-methyl-5,6,7,8,9,10-hexahydro-7,10-epiminocyclohepta[*b*]indol-11-yl)(5-(trifluoromethyl)-1*H*-pyrazol-3-yl)methanone (**37**)

The title compound was obtained after semipreparative chiral separation of racemic (4-fluoro-1-methyl-5,6,7,8,9,10-hexahydro-7,10-epiminocyclohepta[*b*]indol-11-yl)-(5-(trifluoromethyl)-1*H*-pyrazol-3-yl)methanone (**35**) on a Daicel ChiralPak AD column (250 × 10 mm ID, particle size 10 μm) using heptane–EtOH (75:25) as the mobile phase, as a white solid (0.024 g, 28%). NMR spectra, UPLC-MS and HRMS data are consistent with the corresponding racemate **35**. Enantiomeric excess was determined to be >99% after chiral HPLC analysis. [α]_589_^25^ −75.0 (*c* 0.25, CH_3_OH).

#### (7-Fluoro-10-methyl-1,4,5,6-tetrahydroazepino[4,5-*b*]indol-3(2*H*)-yl)-(5-(trifluoromethyl)-1*H*-pyrazol-3-yl)methanone (**38**)

Following the GP1, the title compound was obtained from 7-fluoro-10-methyl-1,2,3,4,5,6-hexahydroazepino[4,5-*b*]indole hydrochloride (**61**) (0.2 g, 0.75 mmol) and 5-(trifluoromethyl)-1*H*-pyrazole-3-carboxylic acid (**C**) (0.135 g, 0.75 mmol), after purification by silica gel flash column chromatography with cyclohexane/EtOAc (0 to 50%) as the eluent, as a white solid (0.019 g, 7%). ^1^H NMR showed the presence of two conformers C*a*/*Cb* in a 50/50 ratio. ^1^H NMR (600 MHz, DMSO-*d*_6_): δ 14.34 (s, NH, 1H, C*a* + *Cb*), 11.24 (br s, NH, 1H, C*a*), 11.19 (br s, 1H, *Cb*), 7.16 (s, 1H, C*a*), 7.14 (s, 1H, *Cb*), 6.67 (ddd, *J* = 11.0, 7.8, 1.2 Hz, 2H, C*a* + *Cb*), 6.60–6.57 (m, 2H, C*a* + *Cb*), 3.96 (m, 1H, C*a* + *Cb*), 3.93–3.90 (m, 3H, C*a* + *Cb*), 3.29–3.25 (m, 2H, C*a* + *Cb*), 3.14–3.10 (m, 2H, C*a* + *Cb*), 2.56 (s, 3H, C*a*), 2.54 (s, 3H, *Cb*). ^13^C NMR (151 MHz, DMSO-*d*_6_): δ 159.5 (Cq, C*a*), 159.3 (Cq, *Cb*), 147.6 (Cq, d, ^1^*J*_CF_ = 239.3 Hz, C*a* + *Cb*), 137.8 (Cq, C*a* + *Cb*), 136.1 (Cq, C*a* + *Cb*), 135.2 (Cq, C*a* + *Cb*), 130.0 (Cq, *J* = 22.2 Hz, C*a*), 129.9 (Cq, *Cb*), 124.9 (Cq, d, *J* = 7.8 Hz, C*a* + *Cb*), 122.43 (Cq, d, *J* = 13.8) 122–42 (Cq, d, *J* = 13.8), 121.4 (Cq, ^1^*J*_CF_ = 268.0 Hz, C*a* + *Cb*), 119.9 (CH, d, *J* = 6.0 Hz, C*a*), 119.7 (CH, *J* = 6.0 Hz, *Cb*), 111.2 (Cq, C*a*), 111.2 (Cq, *Cb*), 107.4 (Cq, d, C*a* + *Cb*), 104.7 (CH, d, *J* = 15.3 Hz, *Cb*), 104.6 (CH, d, *J* = 15.3 Hz, C*a*), 104.2 (CH, C*a* + *Cb*), 49.2 (CH_2_, C*a*), 48.2 (CH_2_, *Cb*), 45.3 (CH_2_, C*a*), 44.8 (CH_2_, *Cb*), 27.8 (CH_2_, C*a*), 27.0 (CH_2_, *Cb*), 26.7 (CH_2_, C*a*), 25.7 (CH_2_, *Cb*), 20.1 (CH_3_, C*a*), 19.9 (CH_3_, *Cb*). ^19^F NMR (376 MHz, DMSO-*d*_6_): δ −59.1 (C*a* + *Cb*), −137.1 (C*a*), −137.2 (*Cb*). UPLC-MS: *t*_R_ = 2.00 min; MS calcd for C_18_H_16_F_4_N_4_O *m*/*z*: 380.33; found, 381.5 [M + H]^+^. HRMS (AP-ESI) *m*/*z*: calcd for C_18_H_16_F_4_N_4_O [M + H]^+^, 381.1338; found, 381.1333.

#### (*R*)-(6-Fluoro-3,9-dimethyl-1,3,4,5-tetrahydro-2*H*-pyrido[4,3-*b*]indol-2-yl)-(5-(trifluoromethyl)-1*H*-pyrazol-3-yl)methanone (**39**)

In a similar fashion to racemic (6-fluoro-3,9-dimethyl-1,3,4,5-tetrahydro-2*H*-pyrido[4,3-*b*]indol-2-yl)-(5-(trifluoromethyl)-1*H*-pyrazol-3-yl)methanone (**33**), the title compound was obtained from commercially available, enantiomerically pure (*R*)-*tert*-butyl 2-methyl-4-oxopiperidine-1-carboxylate (**54**) as a white solid (0.065 g, 51%). NMR spectra, UPLC-MS data, and HRMS data are consistent with the corresponding racemate **33**. Enantiomeric excess was determined to be 97% after chiral HPLC analysis. [α]_589_^25^ +68.8 (*c* 1.0, CH_3_OH).

#### (*S*)-(6-Fluoro-3,9-dimethyl-1,3,4,5-tetrahydro-2*H*-pyrido[4,3-*b*]indol-2-yl)-(5-(trifluoromethyl)-1*H*-pyrazol-3-yl)methanone (**40**)

In a similar fashion to racemic (6-fluoro-3,9-dimethyl-1,3,4,5-tetrahydro-2*H*-pyrido[4,3-*b*]indol-2-yl)-(5-(trifluoromethyl)-1*H*-pyrazol-3-yl)methanone (**33**), the title compound was obtained from commercially available, enantiomerically pure (*S*)-*tert*-butyl 2-methyl-4-oxopiperidine-1-carboxylate (**55**) as a white solid (0.060 g, 50%). NMR spectra, UPLC-MS data, and HRMS data are consistent with the corresponding racemate **33**. Enantiomeric excess was determined to be 78% (least value due to peak-tailing) after chiral HPLC analysis. [α]_589_^25^ −56.8 (*c* 1.0, CH_3_OH).

#### (*R*)- or (*S*)-(6-Fluoro-1,9-dimethyl-1,3,4,5-tetrahydro-2*H*-pyrido[4,3-b]indol-2-yl)-(5-(trifluoromethyl)-1*H*-pyrazol-3-yl)methanone (**41**)

The title compound was obtained after semipreparative chiral separation of racemic (6-fluoro-1,9-dimethyl-1,3,4,5-tetrahydro-2*H*-pyrido[4,3-*b*]indol-2-yl)-(5-(trifluoromethyl)-1*H*-pyrazol-3-yl)methanone (**32**) on a Daicel ChiralPak AD column (250 × 10 mm ID, particle size 10 μm) using heptane/2-propanol (95:5) as the mobile phase, as a white solid (0.005 g, 19%). NMR spectra, UPLC-MS data, and HRMS data are consistent with the corresponding racemate **32**. Enantiomeric excess was determined to be 97% after chiral HPLC analysis. [α]_589_^25^ +57.7 (*c* 1.0, CH_3_OH).

#### (*S*)- or (*R*)-(6-Fluoro-1,9-dimethyl-1,3,4,5-tetrahydro-2*H*-pyrido[4,3-*b*]indol-2-yl)-(5-(trifluoromethyl)-1*H*-pyrazol-3-yl)methanone (**42**)

The title compound was obtained after semipreparative chiral separation of racemic (6-fluoro-1,9-dimethyl-1,3,4,5-tetrahydro-2*H*-pyrido[4,3-*b*]indol-2-yl)-(5-(trifluoromethyl)-1*H*-pyrazol-3-yl)methanone (**32**) on a Daicel ChiralPak AD column (250 × 10 mm ID, particle size 10 μm) using heptane/2-propanol (95:5) as the mobile phase, as a white solid (0.003 g, 11%). NMR spectra, UPLC-MS data, and HRMS data are consistent with the corresponding racemate. Enantiomeric excess was determined to be 63% (least value due to peak-tailing) after chiral HPLC analysis.

### Biology

#### Cell Models and Cell Culture procedures

FRT cells stably expressing mutant F508del-CFTR or G551D-CFTR and the halide-sensitive yellow fluorescent protein (HS-YFP) YFP-H148Q/I152L and CFBE41o-cells stably expressing F508del-CFTR and HS-YFP were generated as previously described.^21,57^ FRT cells were cultured using the Coon’s modification of Ham’s F12 medium, while CFBE41o-cells were cultured in the modified Eagle’s medium. Media were supplemented with 10% fetal calf serum, 2 mM l-glutamine, 100 U/mL of penicillin, and 100 mg/mL of streptomycin. For functional assays of CFTR activity based on the HS-YFP assays, CFBE41o- or FRT cells were plated (50,000 cells/well) on clear-bottom 96-well black microplates (Corning Life Sciences, Acton, MA). The following day, cells were assayed. In the case of FRT cells expressing F508del-CFTR, plates were kept at 32 °C (or treated with correctors at 37 °C where indicated) for an additional 24 h to rescue the mutant trafficking defect, before assays.

Primary bronchial epithelial cells were cultured as previously described.^58^ In brief, epithelial cells were cultured in a serum-free medium (LHC9 mixed with RPMI 1640, 1:1) supplemented with hormones and supplements to support cell number amplification. Then, the cells were seeded at high density on porous membranes (500,000 cells for 1 cm^2^ Snapwell inserts, for Ussing chamber studies; 200,000 cells for 0.33 cm^2^ Mini-Transwell inserts, for TEER/PD measurements). After 24 h, the serum-free medium was replaced with Dulbecco’s modified Eagle’s medium (DMEM)/Ham’s F12 containing 2% fetal bovine serum (FBS) plus hormones and supplements. Differentiation of cells to form a tight epithelium was monitored by measuring transepithelial electrical resistance and potential difference with an epithelial voltohmmeter (EVOM1, World Precision Instruments). After 8–10 days, the apical medium was removed, and the cells received nutrients only from the basolateral side (air–liquid interface, ALI) to promote further differentiation of the epithelium. Cells were maintained under ALI for 2–3 weeks before experiments.

#### HS-YFP-Based Assay for CFTR Activity

Prior assay, cells were washed with phosphate-buffered saline (PBS) containing (in mM) 137 NaCl, 2.7 KCl, 8.1 Na_2_HPO_4_, 1.5 KH_2_PO_4_, 1 CaCl_2_, and 0.5 MgCl_2_. Cells were then incubated for 25 min with 60 μL of PBS plus forskolin (20 μM) and test compounds (at the desired concentration) to stimulate mutant CFTR. Cells were then transferred to microplate readers (FluoStar Optima; BMG Labtech, Offenburg, Germany) for CFTR activity determination. The plate readers were equipped with high-quality excitation (HQ500/20X: 500 ± 10 nm) and emission (HQ535/30M: 535 ± 15 nm) filters for YFP (Chroma Technology). The assay consisted of a continuous 14 s fluorescence reading, 2 s before and 12 s after injection of 165 μL of an iodide-containing solution (PBS with Cl^–^ replaced by I^–^; final I^–^ concentration 100 mM). Data were normalized to the initial background-subtracted fluorescence. To determine the I^–^ influx rate, the final 10 s of the data for each well was fitted with a linear function to extrapolate the initial slope (d*F*/d*t*).

Each experimental condition was tested in three independent experiments, each one performed with three biological replicates (*n* = 9).

#### TEER/PD Measurements

Differentiated bronchial epithelia were treated with compounds included in the appropriate culture medium at the indicated concentrations for 24 h at 37 °C and 5% CO_2_, before measuring the TEER and/or PD by means of an epithelial voltohmmeter (EVOM1, World Precision Instruments).

The electrical measurements were done in Coon’s modified Ham’s F-12 medium, where NaHCO_3_ was replaced with 20 mM Na–HEPES (pH 7.3). TEER and PD were measured in each well under basal conditions, after ENaC inhibition with apical amiloride (10 μM), after CFTR stimulation with forskolin (10 μM) plus test compounds (at the desired concentration) on both sides, and after CFTR inhibition with apical PPQ102 (30 μM). After each treatment, we waited 10 min before recording the electrical parameters. The TEER and PD values for each well were converted into short-circuit current equivalent by Ohm’s law.

Each experimental condition was tested in three independent experiments, each one performed with three biological replicates (*n* = 9).

#### Short-Circuit Current Recordings

Differentiated bronchial epithelia on Snapwell inserts were mounted in a Ussing chamber with internal fluid circulation. Apical and basolateral hemichambers were filled with 5 mL of a solution containing (in mM) 126 NaCl, 0.38 KH_2_PO_4_, 2.13 K_2_HPO_4_, 1 MgSO_4_, 1 CaCl_2_, 24 NaHCO_3_, and 10 glucose, and both sides were continuously bubbled with a 5% CO_2_–95% air mixture, with the temperature of the solution maintained at 37 °C. The transepithelial voltage was short-circuited with a voltage clamp (DVC-1000, World Precision Instruments) connected to the apical and basolateral chambers via Ag/AgCl electrodes and agar bridges (1 M KCl in 1% agar). The offset between voltage electrodes and the fluid electrical resistance were set to zero before each set of experiments. The short-circuit current was recorded with a PowerLab 4/25 (ADInstruments) analog-to-digital converter connected to a personal computer.

Each experimental condition was tested in three independent experiments, each one performed with three biological replicates (*n* = 9).

#### Statistical Analysis

Each experimental condition was tested in three independent experiments, each one performed with three biological replicates (*n* = 9). The Kolmogorov–Smirnov test was used to evaluate the assumption of normality. The statistical significance of the effect of single treatments on CFTR activity or expression was tested by parametric one-way ANOVA, followed by the Dunnett multiple comparisons test (all groups against the control group) as a post-hoc test. In the case of a combination of treatments, statistical significance was verified by ANOVA, followed by the Tukey test (for multiple comparisons) as the post-hoc test. Normally distributed data are expressed as mean ± SD, and significances are two-sided. Differences were considered statistically significant when *P* was less than 0.05.

### *In Vitro* ADMET

#### Aqueous Kinetic Solubility Assay

The aqueous kinetic solubility was determined from a 10 mM DMSO stock solution of test compound in PBS at pH 7.4. The study was performed by incubation of an aliquot of 10 mM DMSO stock solution in PBS (pH 7.4) at a target concentration of 250 μM (2.5% DMSO). The incubation was carried out under shaking at 25 °C for 24 h, followed by centrifugation at 21,100*g* for 30 min. The supernatant was further diluted (4:1) with CH_3_CN and the dissolved test compound was quantified by UV at 215 nm on a Waters ACQUITY UPLC/MS system consisting of an SQD mass spectrometer equipped with an electrospray ionization interface and a photodiode array detector. Electrospray ionization in the positive mode was used in the mass scan range of 100–500 Da. The PDA range was 210–400 nm. The analyses were run on an ACQUITY UPLC BEH C_18_ column (50 × 2.1 mm ID, particle size 1.7 μm) with a VanGuard BEH C_18_ precolumn (5 × 2.1 mm ID, particle size 1.7 μm) using 10 mM NH_4_OAc in H_2_O at pH 5 adjusted with AcOH (A) and 10 mM NH_4_OAc in CH_3_CN–H_2_O (95:5) at pH 5 (B) as the mobile phase. The aqueous kinetic solubility (in μM) was calculated by dividing the peak areas of the dissolved test compound and the test compound in the reference (250 μM of test compound in CH_3_CN) and multiply by the target concentration and dilution factor.

#### Liver Microsomal Stability Assay

Phase I: 10 mM DMSO stock solution of the test compound was preincubated at 37 °C for 15 min with rat, dog, or human liver microsomes in 0.1 M Tris–HCl buffer (pH 7.5) with 10% DMSO. The final concentration was 4.6 μM. After preincubation, the cofactors (NADPH, G6P, G6PDH, and MgCl_2_ predissolved in 0.1 M Tris–HCl) were added to the incubation mixture and the incubation was continued at 37 °C for 1 h.

Phase II: 10 mM DMSO stock solution of the test compound was preincubated at 37 °C for 15 min with human liver microsomes added alamethicin in 0.1 M Tris–HCl buffer (pH 7.5) with 10% DMSO. The final concentration was 4.6 μM. After preincubation, the cofactors (UDPGA, d-saccharic acid lactone, and MgCl_2_ predissolved in 0.1 M Tris–HCl) were added to the incubation mixture and the incubation was continued at 37 °C for 1 h.

For both phase I and II studies: At each time point (0, 5, 15, 30, and 60 min), 30 μL of the incubation mixture was diluted with 200 μL of cold CH_3_CN spiked with 200 nM of an appropriate internal standard, followed by centrifugation at 3270*g* for 15 min. The supernatant was further diluted with H_2_O (1:1) for analysis. A reference incubation mixture (microsomes without cofactors) was prepared for each test compound and analyzed at *t* = 0 and 60 min in order to verify the compound’s stability in the matrix. The two time points were diluted as for the time points of the incubation mixture above. The supernatants were analyzed by LC/MS–MS on a Waters ACQUITY UPLC/MS TQD system consisting of a TQD (triple quadrupole detector) mass spectrometer equipped with an electrospray ionization interface and a photodiode array eλ detector. Electrospray ionization was applied in positive mode. Compound-dependent parameters as MRM transitions and collision energy were developed for each compound. The analyses were run on an ACQUITY UPLC BEH C_18_ (50 × 2.1 mm ID, particle size 1.7 μm) with a VanGuard BEH C_18_ precolumn (5 × 2.1 mm ID, particle size 1.7 μm) at 40 °C, using H_2_O + 0.1% HCOOH (A) and CH_3_CN + 0.1% HCOOH (B) as the mobile phase. The percentage of test compound remaining at each time point relative to *t* = 0 was calculated by the response factor on the basis of the internal standard peak area. The percentage of test compound versus time was plotted and fitted by GraphPad Prism (GraphPad Software, Version 5 for Windows, CA, USA, www.graphpad.com) to estimate the compound’s half-life (*t*_1/2_), which was reported as mean value along with the standard deviation (*n* = 3).

#### HepG2 Cell toxicity Assay

##### Cell Culture Conditions

To increase the detection of drug-induced mitochondrial effects in a preclinical cell-based assay, HepG2 hepatocellular carcinoma cells (ATCC HB-8065) were forced to rely on mitochondrial oxidative phosphorylation rather than glycolysis by substituting galactose (10 mM) for glucose (25 mM) in the growth media (DMEM, Life Technologies).

##### Media Composition

High-glucose media: high-glucose DMEM (Invitrogen 11995-065) containing 25 mM glucose, 1.0 mM sodium pyruvate, supplemented with 5 mM *N*-(2-hydroxyethyl)piperazine-*N*′-(2-ethanesulfonic acid) (HEPES), and 10% FBS. Galactose media: DMEM deprived of glucose (Invitrogen 11966-025), supplemented with 10 mM galactose, 2.0 mM glutamine (6 mM final), 5.0 mM HEPES, 10% FBS, and 1.0 mM sodium pyruvate.

##### Cell Viability Assessment

For the cytotoxicity assay, cells were plated at 20,000 cells/well in 100 μL of cell culture media in 96-well plates and allowed to grow overnight. The cells were treated for 24 h with 2.0 or 20 μM of each compound. All compounds were dissolved in DMSO with a stock concentration of 4 mM. The first dilution step of compounds was prepared in DMSO (200× stock solutions), while the second dilution step was carried out in a complete cell culture medium (5% DMSO). Of this dilution, 10 μL were added to the wells of the 96-well plate, with a final DMSO concentration of 0.5%. Rotenone, a well-known mitochondrial inhibitor, was used as a reference compound. After treatment, cellular viability was assessed by using two different assays, run on independent plates: the CellTiter-Glo (CTG) Luminescent Cell Viability Assay (Promega), which determines the number of viable cells based on the quantitation of the ATP present, and the thiazolyl blue tetrazolium blue (MTT) dye (Aldrich), which is converted to water-insoluble MTT formazan crystals by mitochondrial dehydrogenases of living cells. Each experimental condition (i.e., control, reference, and compounds’ doses) has been tested in three technical replicates.

### *In Vivo* Pharmacology

#### Animals

Male Sprague–Dawley rats, 2 month old and weighing 175–200 g (Charles River, Calco, Italy), were used. Animals were group-housed in ventilated cages and had free access to food and water. They were maintained under a 12 h light/dark cycle (lights on at 8:00 am) at a controlled temperature (21 ± 1 °C) and relative humidity (55 ± 10%). All experiments were carried out in accordance with the guidelines established by the European Communities Council Directive (Directive 2010/63/EU of September 22, 2010) and approved by the National Council on Animal Care of the Italian Ministry of Health. All efforts were made to minimize animal suffering and to use the minimal number of animals required to produce reliable results.

#### Pharmacokinetic Methods

Compound **39** was administered intravenously (i.v.) and orally (p.o.) to cannulated Sprague–Dawley rats at doses of 3 and 10 mg/kg, respectively. PEG400/Tween 80/saline solution was used as a vehicle at 10/10/80% in volume, respectively. Three animals per dose were treated. Blood samples at 0, 15, 30, 60, 90, 120, 240, and 360 min after administration were collected for p.o. arm. Blood samples at 0, 5, 15, 30, 60, 90, 120, and 240 min after administration were collected for i.v. arm. Plasma was separated from blood by centrifugation for 15 min at 3500 rpm at 4 °C, collected in an Eppendorf tube, and frozen (−80 °C). Control animals treated with vehicle only were also included in the experimental protocol.

#### Sample Preparation for Lung Exposure Analysis

Three animals per dose and timing were treated. Compound **39** was dissolved in PEG400/Tween80/saline solution at 10/10/80% in volume and administered orally at a dose of 10 mg/kg. After 120 and 240 min, rats were sacrificed and lungs were immediately dissected, frozen on dry ice, and stored at −80 °C until analysis. Lung samples were homogenized in RIPA buffer (150 mM NaCl, 1.0% Triton X-100, 0.5% sodium deoxycholate, 0.1% sodium dodecyl sulfate, 50 mM Tris, and pH 8.0) and then split into two aliquots kept at −80 °C until analysis. An aliquot was used for compound lung-level evaluations. The second aliquot was kept for protein content evaluation using the bicinchoninic acid assay (Thermo Scientific, Rockford, IL, USA).

#### Bioanalytical Analyses

Plasma samples were centrifuged at 21,100*g* for 15 min at 4 °C, while homogenized lung samples were vigorously whirled. An aliquot of each sample was extracted (1:3) with cold CH_3_CN containing 200 nM of an appropriate internal standard being a close analogue of the parent compound. A calibration curve was prepared in both blank mouse plasma and naïve lung homogenate over a 1 nM to 10 μM range. Three quality controls were prepared by spiking the parent compound in both blank mouse plasma and naïve lung homogenate to 20, 200, and 2000 nM as final concentrations. The calibrators and quality controls were extracted (1:3) with the same extraction solution as the plasma and lung samples. The plasma and lung samples, the calibrators, and quality controls were centrifuged at 3270*g* for 15 min at 4 °C. The supernatants were further diluted (1:1) with H_2_O and analyzed by LC/MS–MS on a Waters ACQUITY UPLC/MS TQD system consisting of a TQD mass spectrometer equipped with an electrospray ionization interface and a photodiode array eλ detector. Electrospray ionization was applied in the positive mode. Compound-dependent parameters such as MRM transitions and collision energy were developed for the parent compound and the internal standard. The mobile phase was H_2_O + 0.1% HCOOH (A) and CH_3_CN + 0.1% HCOOH (B) at a flow rate of 0.5 mL/min. For plasma samples, the analyses were run on an ACQUITY UPLC BEH C_18_ (50 × 2.1 mm ID, particle size 1.7 μm) with a VanGuard BEH C_18_ precolumn (5 × 2.1 mm ID, particle size 1.7 μm) at 40 °C. A linear gradient was applied starting at 30% B with an initial hold for 0.2 min and then 30–100% B in 2 min. For lung samples, the analyses were run on an ACQUITY UPLC BEH C_18_ (100 × 2.1 mm ID, particle size 1.7 μm) with a VanGuard BEH C_18_ precolumn (5 × 2.1 mm ID, particle size 1.7 μm) at 40 °C. A linear gradient was applied starting at 30% B with an initial hold for 0.2 min and then 30–100% B in 6 min. All samples (plasma and lung samples, calibrators, and quality controls) were quantified by MRM peak area response factor in order to determine the levels of the parent compound in plasma and lung. The concentrations versus time data were plotted and the profiles were fitted using PK Solutions Excel Application (Summit Research Service, USA) in order to determine the pharmacokinetic parameters.

## Supporting Information Available

UPLC/MS traces and ^1^H, ^13^C, and ^19^F NMR spectral data for final compounds; chiral HPLC data for enantioenriched final compounds; UPLC/MS traces of primary hits from screening; and experimental procedures and results from additional *in vivo* assays (PDF)
Molecular formula strings for all final compounds (CSV)

## Abbreviations

CF: cystic fibrosis
CFTR: cystic fibrosis transmembrane conductance regulator
F508del: deletion of phenylalanine 508
FRT: Fischer rat thyroid
HS-YFP: halide-sensitive yellow fluorescent protein
HBE: primary human bronchial epithelial cells
TEER/PD: transepithelial electrical resistance/potential difference
CPT-cAMP: 8-(4-chlorophenylthio)adenosine 3′,5′-cyclic monophosphate
PPQ-102: 7,9-dimethyl-11-phenyl-6-(5-methylfuran-2-yl)-5,6-dihydro-pyrimido-[4′,5′-3,4]pyrrolo[1,2-*a*]quinoxaline-8,10-(7*H*,9*H*)-dione
ENaC: epithelial sodium channel (or amiloride-sensitive sodium channel)
NADPH: nicotinamide adenine dinucleotide phosphate (reduced)
UDPGA: uridine-diphosphate-glucuronic acid trisodium salt
HATU: 1-[bis(dimethylamino)methylene]-1*H*-1,2,3-triazolo[4,5-*b*]pyridinium 3-oxide hexafluorophosphate
DIPEA: *N*,*N*-diisopropylethylamine
EDC·HCl: *N*-(3-dimethylaminopropyl)-*N*′-ethylcarbodiimide hydrochloride

## Footnotes

a Pure regioisomer **52** was obtained as a racemate, starting from enantiopure (*R*)-**50**, because of the epimerization process during Fischer indole synthesis.

b Pure regioisomer **52** was obtained as a racemate starting from enantiopure (*S*)-**51** because of the epimerization process during Fischer indole synthesis.
