# Supplementary material for: Identification, Structure–Activity Relationship, and Biological Characterization of 2,3,4,5-Tetrahydro-1H-pyrido[4,3-b]indoles as a Novel Class of CFTR Potentiators
Source: J Med Chem. 2020 Sep 18;63(19):11169–94. doi: 10.1021/acs.jmedchem.0c01050 (PMC8011931; doi:10.1021/acs.jmedchem.0c01050)
Supplement: Supplementary file 1 — jm0c01050_si_001.pdf [file jm0c01050_si_001.pdf]

SUPPORTING INFORMATION

Identification, Structure-Activity Relationship and Biological Characterization of 2,3,4,5-Tetrahydro-1H-pyrido[4,3-b]indoles as a Novel Class of CFTR Potentiators

Nicoletta Brindani,[1] Ambra Gianotti,[2] Simone Giovani,[1] Francesca Giacomina,[1] Paolo Di Fruscia,[1] Federico Sorana,[1] Sine Mandrup Bertozzi,[3] Giuliana Ottonello,[3] Luca Goldoni,[3] Ilaria Penna,[1] Debora Russo,[1] Maria Summa,[3] Rosalia Bertorelli,[3] Loretta Ferrera,[2] Emanuela Pesce,[2] Elvira Sondo,[2] Luis J. V. Galietta,[4,5] Tiziano Bandiera,[1] Nicoletta Pedemonte,[2,] Fabio Bertozzi[1,]**

[1]D3-PharmaChemistry, Istituto Italiano di Tecnologia (IIT), 16163 - Genova, Italy; [2]UOC Genetica Medica, IRCCS Istituto Giannina Gaslini, 16147 - Genova, Italy; [3]Analytical Chemistry and Translational Pharmacology, Istituto Italiano di Tecnologia (IIT), 16163 - Genova, Italy; [4]Telethon Institute of Genetics and Medicine (TIGEM), 80078 - Pozzuoli, Italy; [5]Department of Translational Medical Sciences (DISMET), University of Naples Federico II, 80138 - Naples, Italy.

Table of contents

- **Experimental Part** S3
- *1. Representative ^{1}H, ^{13}C, ^{19}F NMR spectra of final compounds **1-42*** S3
  - 1.1. Quantitative ^{1}H NMR S3
- *2. LC/MS analyses of Hits 1-6 and final compounds **1-42*** S62
- *3. Chiral HPLC analyses of final compounds **32**, **33**, **35-37**, **39-42*** S87
- *4. In-vivo characterization of compound **39*** S92
- *5. References* S94

Experimental part

1. Representative ^{1}H, ^{13}C, ^{19}F NMR Spectra of final compounds 1-42

NMR experiments were run at 300 K on a Bruker Avance III 400 system (400.13 MHz for ^{1}H, and 100.62 MHz for ^{13}C), equipped with a BBI probe and Z-gradients, and Bruker FT NMR Avance III 600 MHz spectrometer equipped with a 5 mm CryoProbeTM QCI $^{1}H/^{19}F–^{13}C/^{15}N$–D quadruple resonance, a shielded z-gradient coil and the automatic sample changer SampleJet™ NMR system (600 MHz for ^{1}H, 151 MHz for ^{13}C and 565 MHz for ^{19}F). Chemical shifts for ^{1}H and ^{13}C spectra were reported in parts per million (ppm), calibrating the residual non-deuterated solvent peak for the ^{1}H and ^{13}C, respectively to 7.26 ppm and 77.16 ppm for $CDCl_3$ and 2.50 ppm and 39.52 ppm for DMSO-d_6, whereas spectra in D_2O were referred to TSP (Trimethylsilylpropanoic acid) peak set at 0.00 ppm.

1.1 Quantitative ^{1}H NMR

Purity of the final compounds was determined by UPLC/MS and quantitative ^{1}H NMR (qNMR). qNMR experiments were acquired with 64 transients, after an automatic 90° degree pulse length optimization,[1] by using 65536 digit points, 30 s of interpulses delay, and the receiver gain fixed (64), the spectral width was 22.55 ppm with the offset positioned at 6.17 ppm. An apodization esponential function equivalent to 0.3Hz was applied to FIDs before Fourier transform. Spectra were phased, and baseline corrected, automatically. For purity evaluation by NMR assay (qNMR), the signal of final compound (10 mM solution in DMSO-d_6), was compared to the peak of an equimolar external standard solution of maleic acid (TraceCERT, 99.99%, Sigma-Aldrich, Milan, Italy), after the normalization for the number of protons generating such signals, by using the PULCON method.[2]

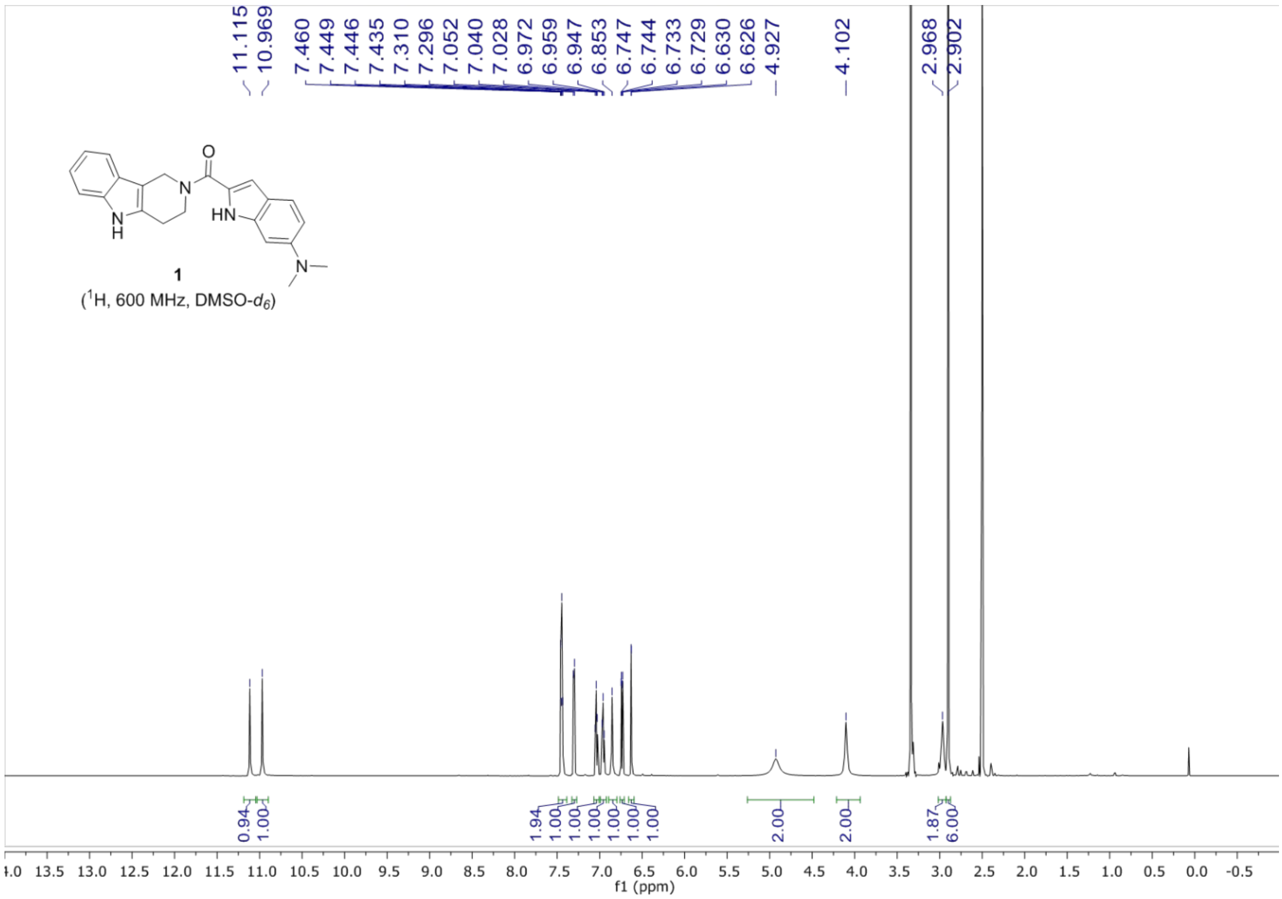
11.115
10.969
7.460
7.449
7.446
7.435
7.310
7.296
7.052
7.040
7.028
6.972
6.959
6.947
6.853
6.747
6.744
6.733
6.729
6.630
6.626
4.927
4.102
2.968
2.902
1
(^{1}H, 600 MHz, DMSO-d_6)
0.94
1.00
1.94
1.00
1.00
1.00
1.00
1.00
1.00
2.00
2.00
1.87
6.00
f1 (ppm)

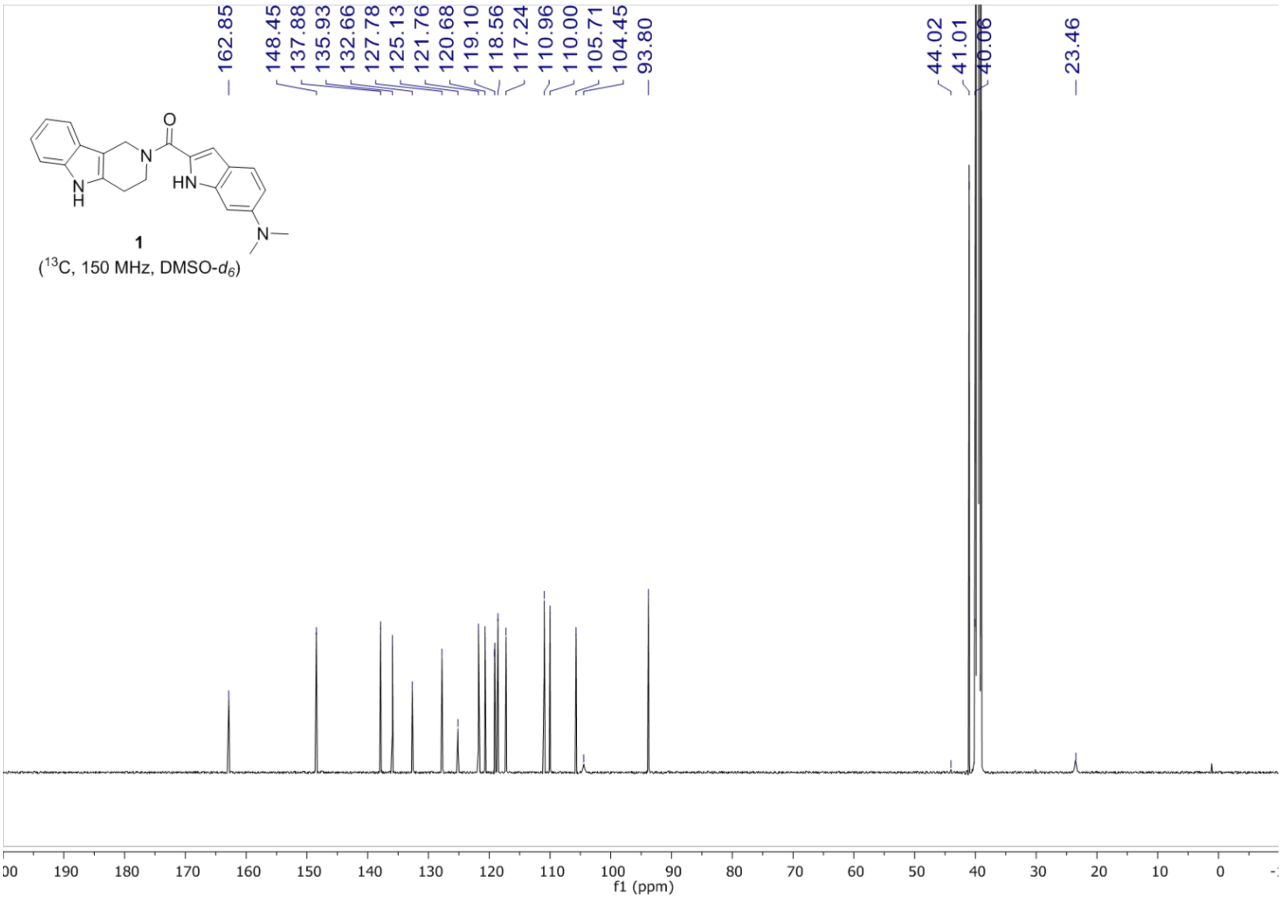
162.85
148.45
137.88
135.93
132.66
127.78
125.13
121.76
120.68
119.10
118.56
117.24
110.96
110.00
105.71
104.45
93.80
44.02
41.01
40.06
23.46
1
(^{13}C, 150 MHz, DMSO-d_6)
f1 (ppm)

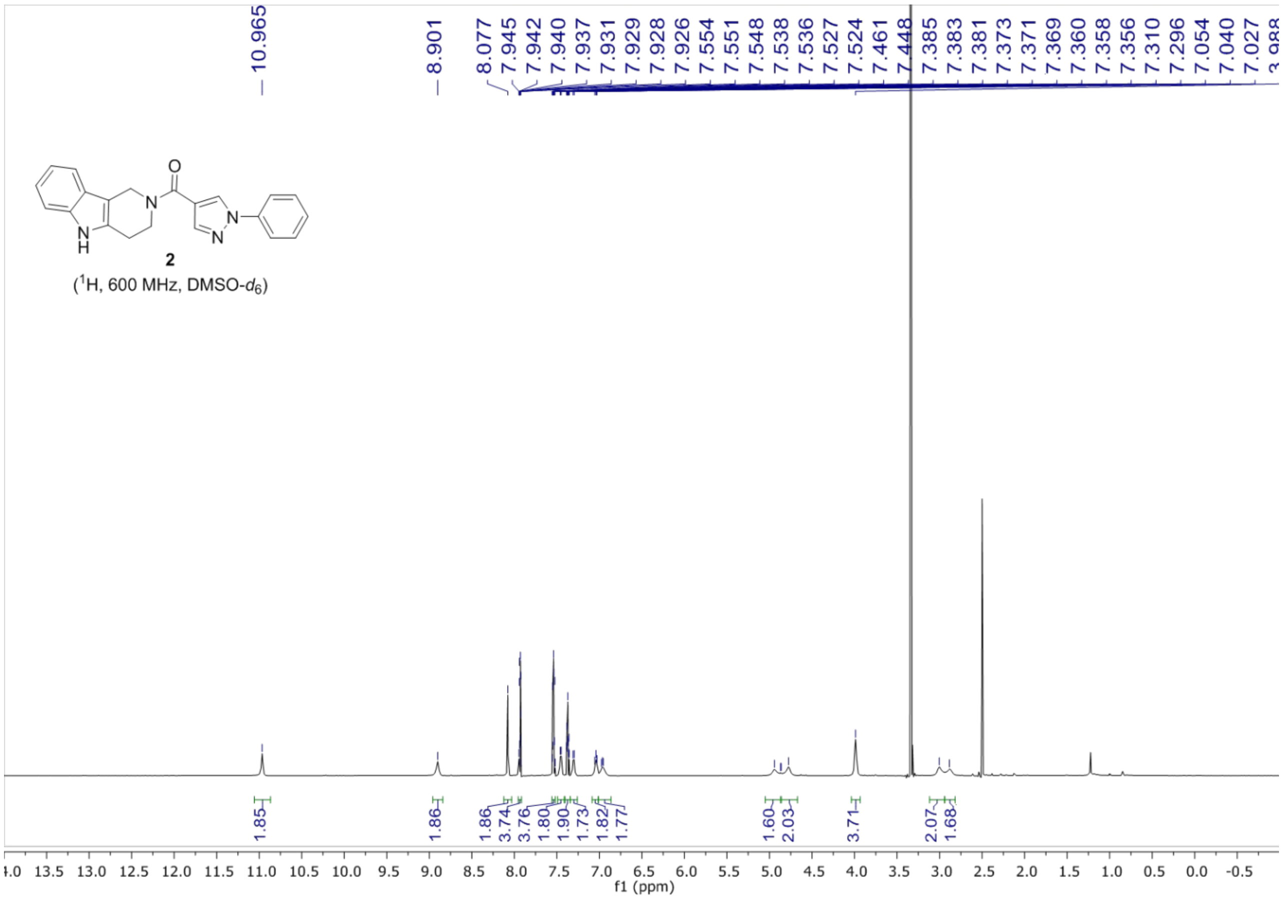
10.965
8.901
8.077
7.945
7.942
7.940
7.937
7.931
7.929
7.928
7.926
7.554
7.551
7.548
7.538
7.536
7.527
7.524
7.461
7.448
7.385
7.383
7.381
7.373
7.371
7.369
7.360
7.358
7.356
7.310
7.296
7.054
7.040
7.027
[illegible]
2
(^{1}H, 600 MHz, DMSO-d_6)
1.85
1.86
1.86
3.74
3.76
1.80
1.90
1.73
1.82
1.77
1.60
2.03
3.71
2.07
1.68
f1 (ppm)

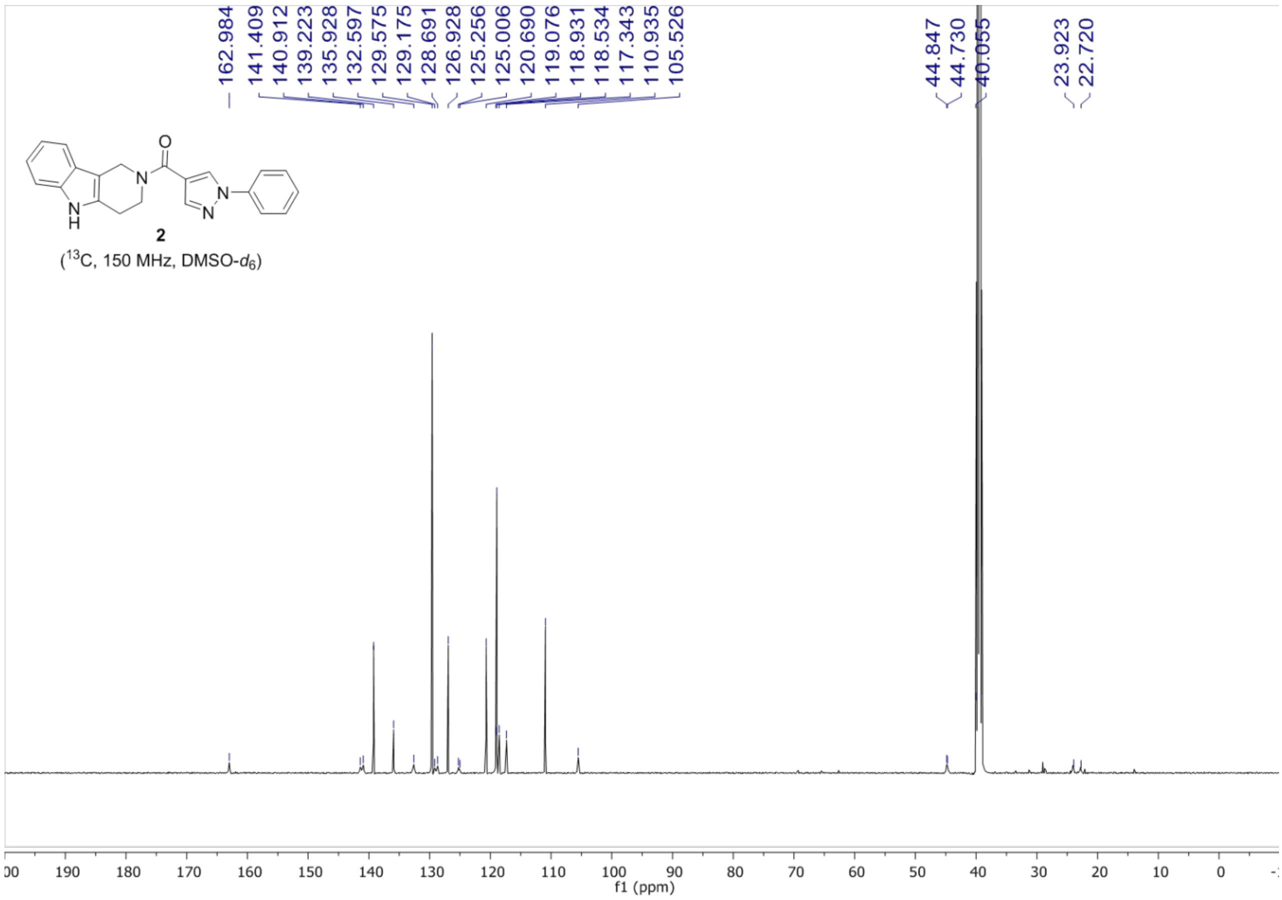
162.984
141.409
140.912
139.223
135.928
132.597
129.575
129.175
128.691
126.928
125.256
125.006
120.690
119.076
118.931
118.534
117.343
110.935
105.526
44.847
44.730
40.055
23.923
22.720
2
(^{13}C, 150 MHz, DMSO-d_6)
f1 (ppm)

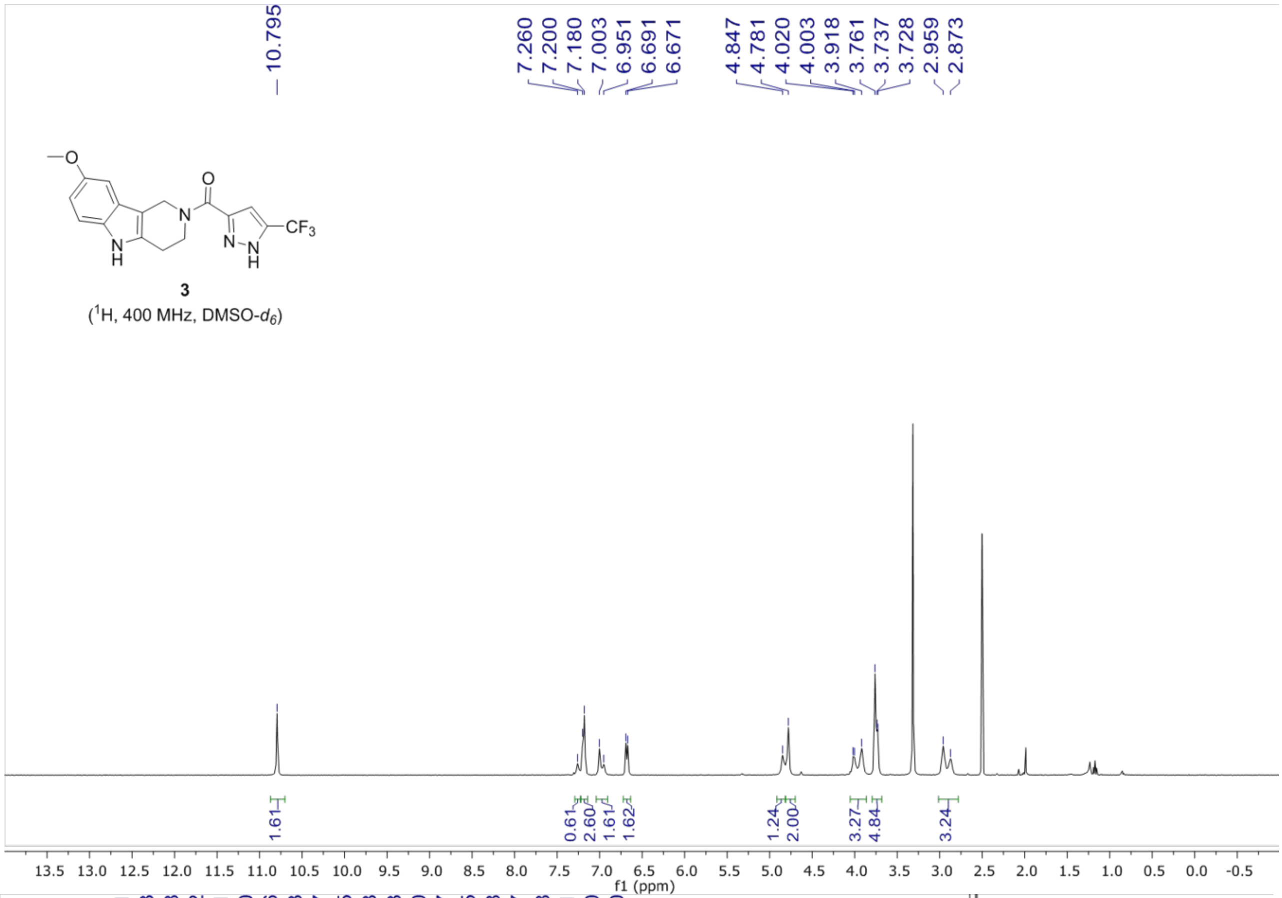
10.795
7.260
7.200
7.180
7.003
6.951
6.691
6.671
4.847
4.781
4.020
4.003
3.918
3.761
3.737
3.728
2.959
2.873
O
N
CF3
N
H
N
N
H
3
(1H, 400 MHz, DMSO-d6)
1.61
0.61
2.60
1.61
1.62
1.24
2.00
3.27
4.84
3.24
13.5 13.0 12.5 12.0 11.5 11.0 10.5 10.0 9.5 9.0 8.5 8.0 7.5 7.0 6.5 6.0 5.5 5.0 4.5 4.0 3.5 3.0 2.5 2.0 1.5 1.0 0.5 0.0 -0.5
f1 (ppm)

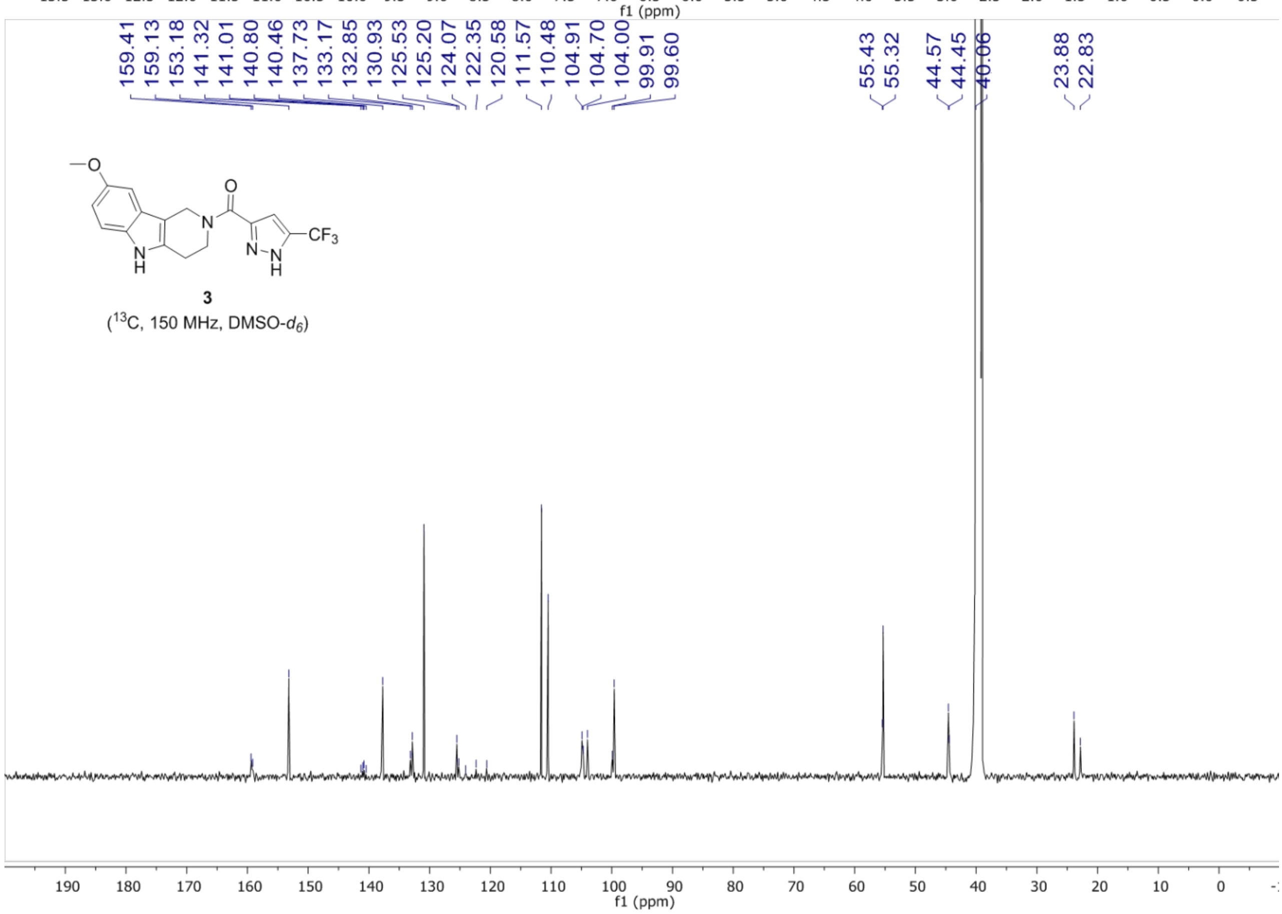
159.41
159.13
153.18
141.32
141.01
140.80
140.46
137.73
133.17
132.85
130.93
125.53
125.20
124.07
122.35
120.58
111.57
110.48
104.91
104.70
104.00
99.91
99.60
55.43
55.32
44.57
44.45
40.06
23.88
22.83
O
N
CF3
N
H
N
N
H
3
(13C, 150 MHz, DMSO-d6)
190 180 170 160 150 140 130 120 110 100 90 80 70 60 50 40 30 20 10 0
f1 (ppm)

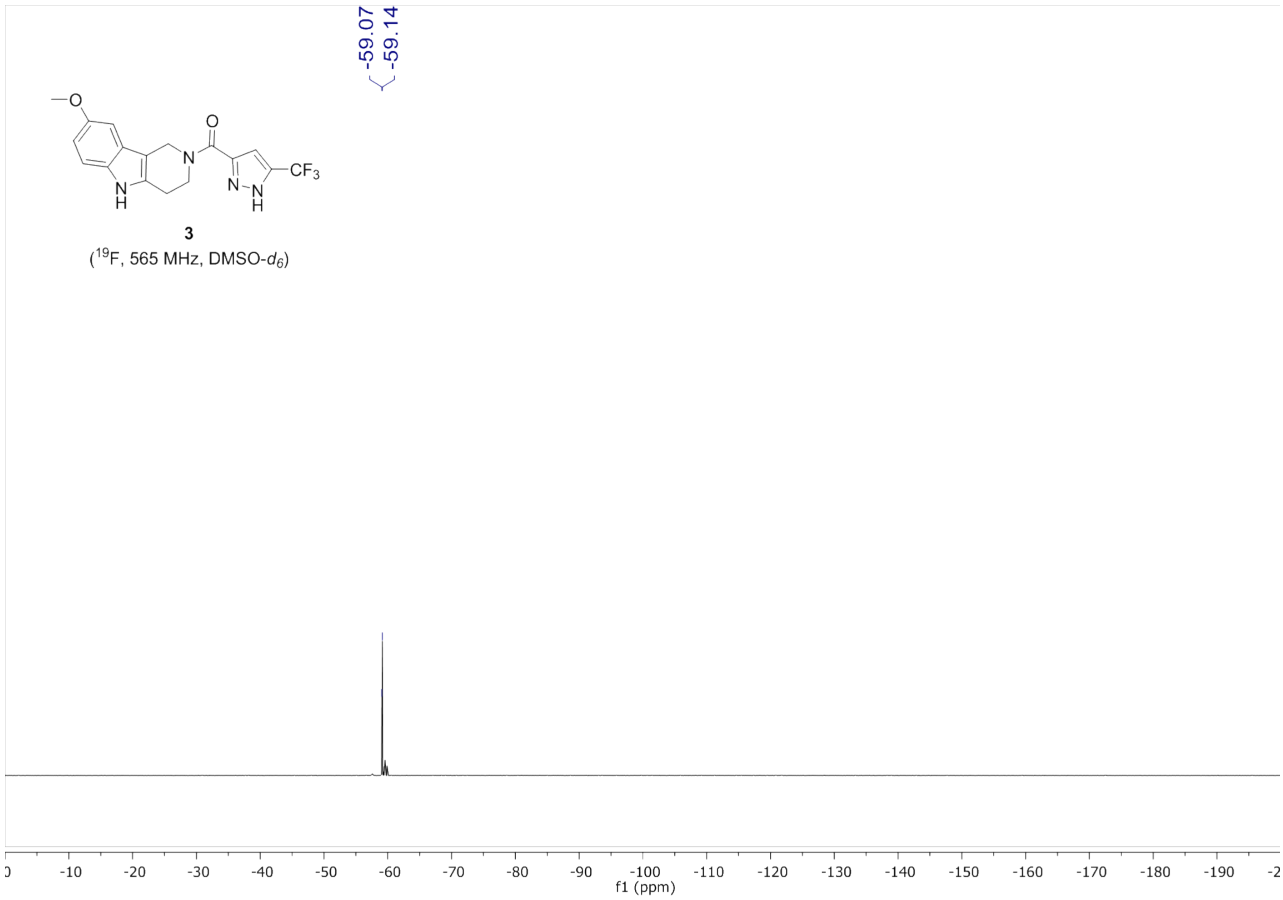
-59.07
-59.14
O
N
CF3
N
N
H
H
3
(19F, 565 MHz, DMSO-d6)
f1 (ppm)

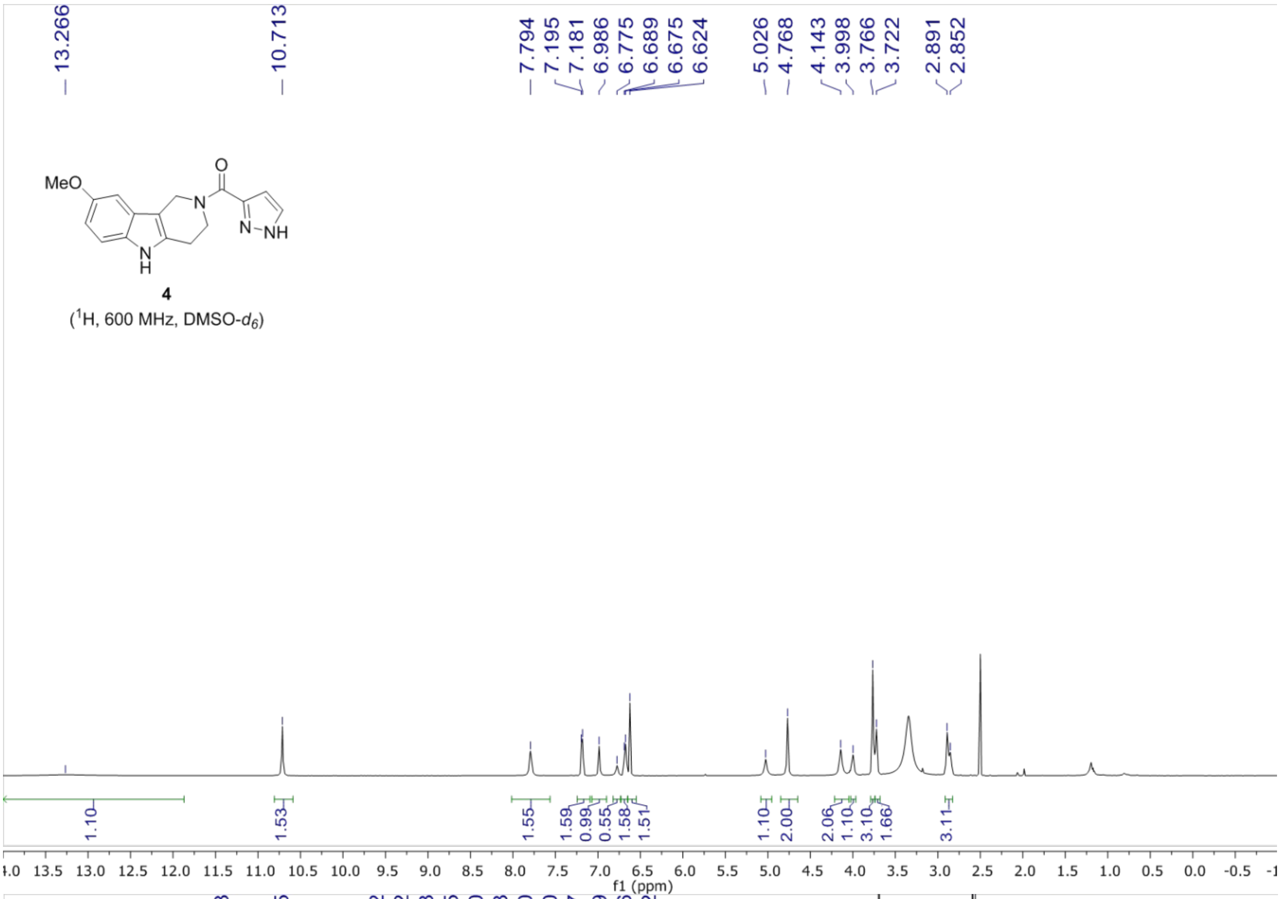
13.266
10.713
7.794 7.195 7.181 6.986 6.775 6.689 6.675 6.624
5.026 4.768 4.143 3.998 3.766 3.722 2.891 2.852
MeO
O
N
N
NH
H
4
(^{1}H, 600 MHz, DMSO-d_6)
1.10 1.53 1.55 1.59 0.99 0.55 1.58 1.51 1.10 2.00 2.06 1.10 3.10 1.66 3.11
f1 (ppm)

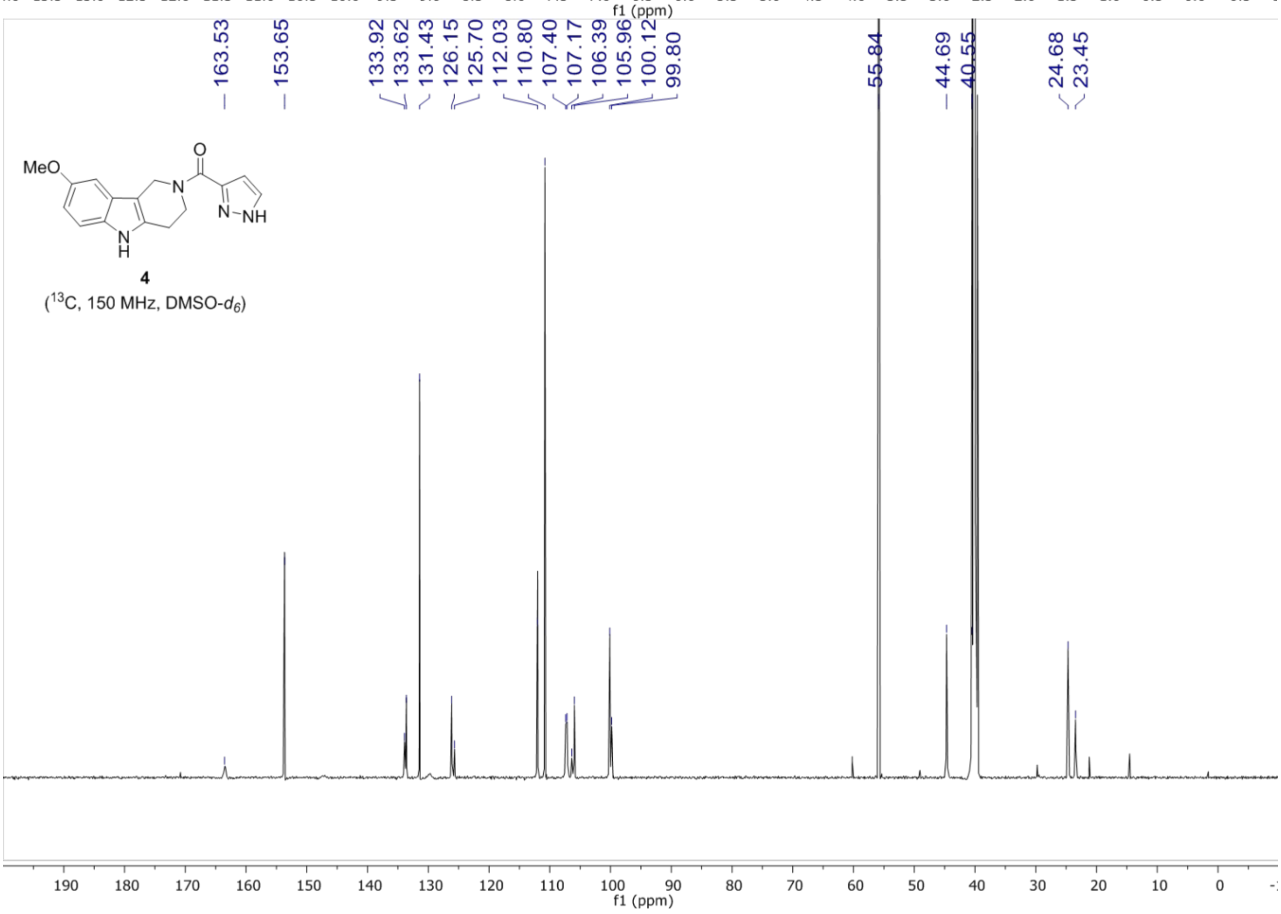
163.53
153.65
133.92 133.62 131.43 126.15 125.70 112.03 110.80 107.40 107.17 106.39 105.96 100.12 99.80
55.84
44.69 40.55
24.68 23.45
MeO
O
N
N
NH
H
4
(^{13}C, 150 MHz, DMSO-d_6)
f1 (ppm)

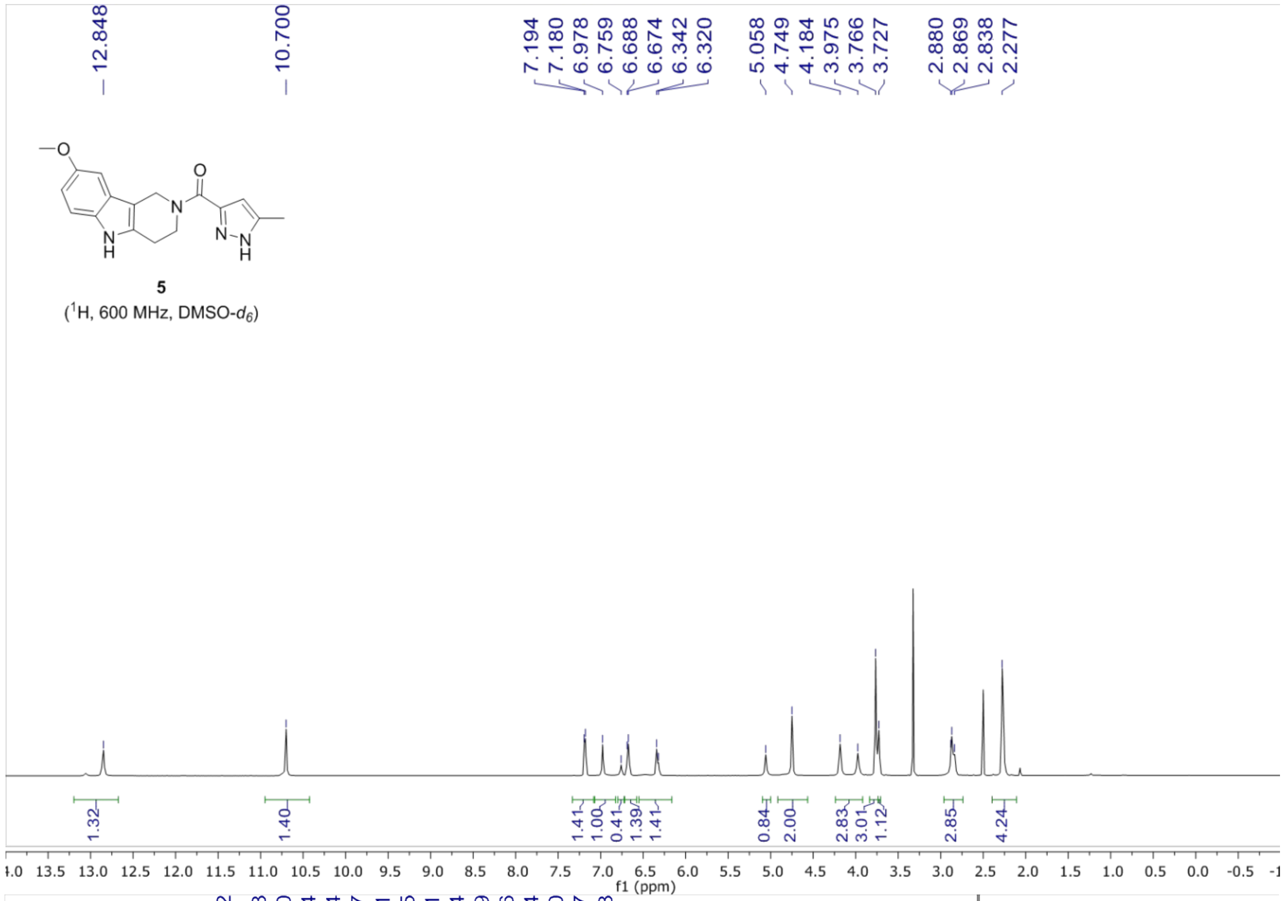

5

(^{1}H, 600 MHz, DMSO-d_6)

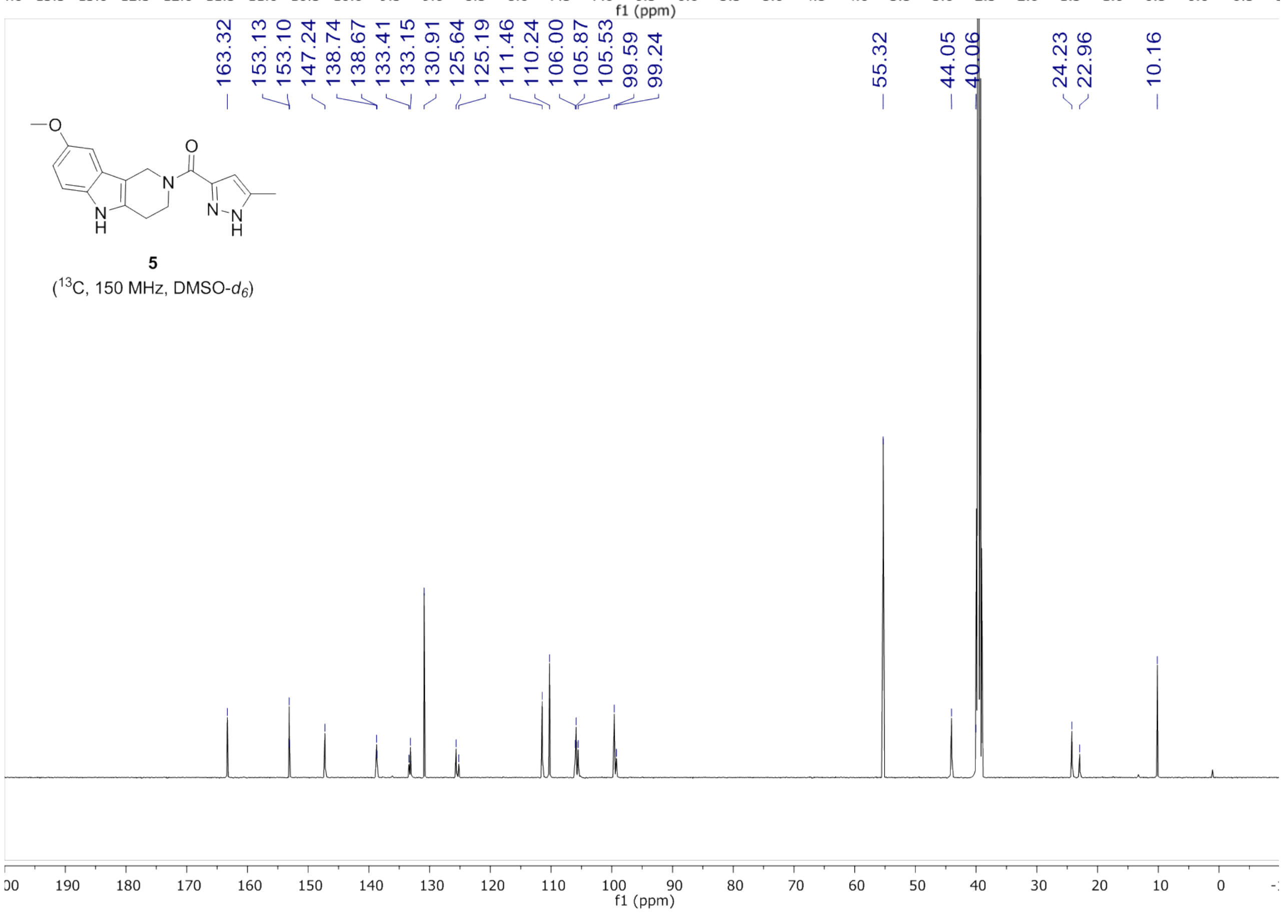

5

(^{13}C, 150 MHz, DMSO-d_6)

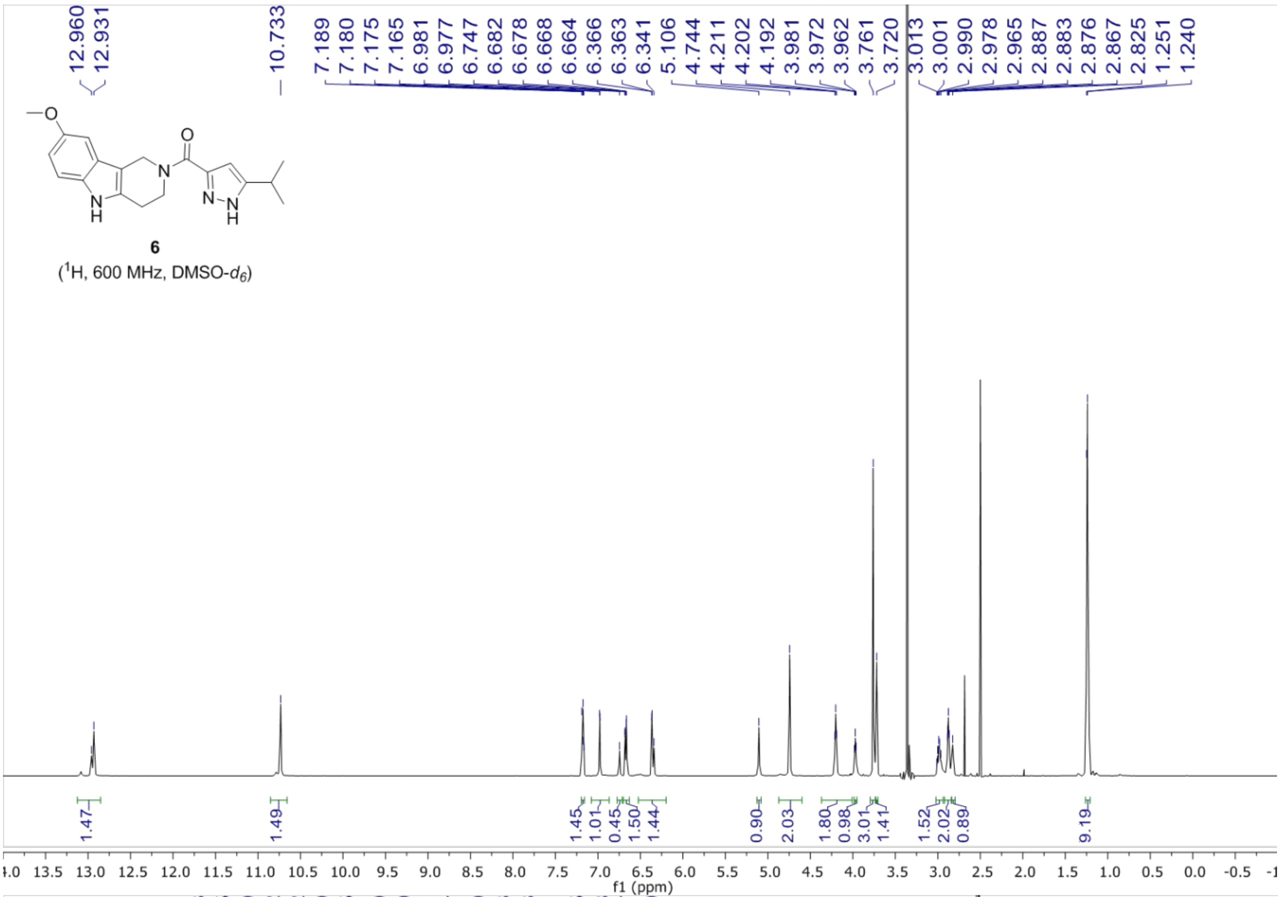
12.960
12.931
10.733
7.189
7.180
7.175
7.165
6.981
6.977
6.747
6.682
6.678
6.668
6.664
6.366
6.363
6.341
5.106
4.744
4.211
4.202
4.192
3.981
3.972
3.962
3.761
3.720
3.013
3.001
2.990
2.978
2.965
2.887
2.883
2.876
2.867
2.825
1.251
1.240
6
(1H, 600 MHz, DMSO-d6)
1.47
1.49
1.45
1.01
0.45
1.50
1.44
0.90
2.03
1.80
0.98
3.01
1.41
1.52
2.02
0.89
9.19
f1 (ppm)

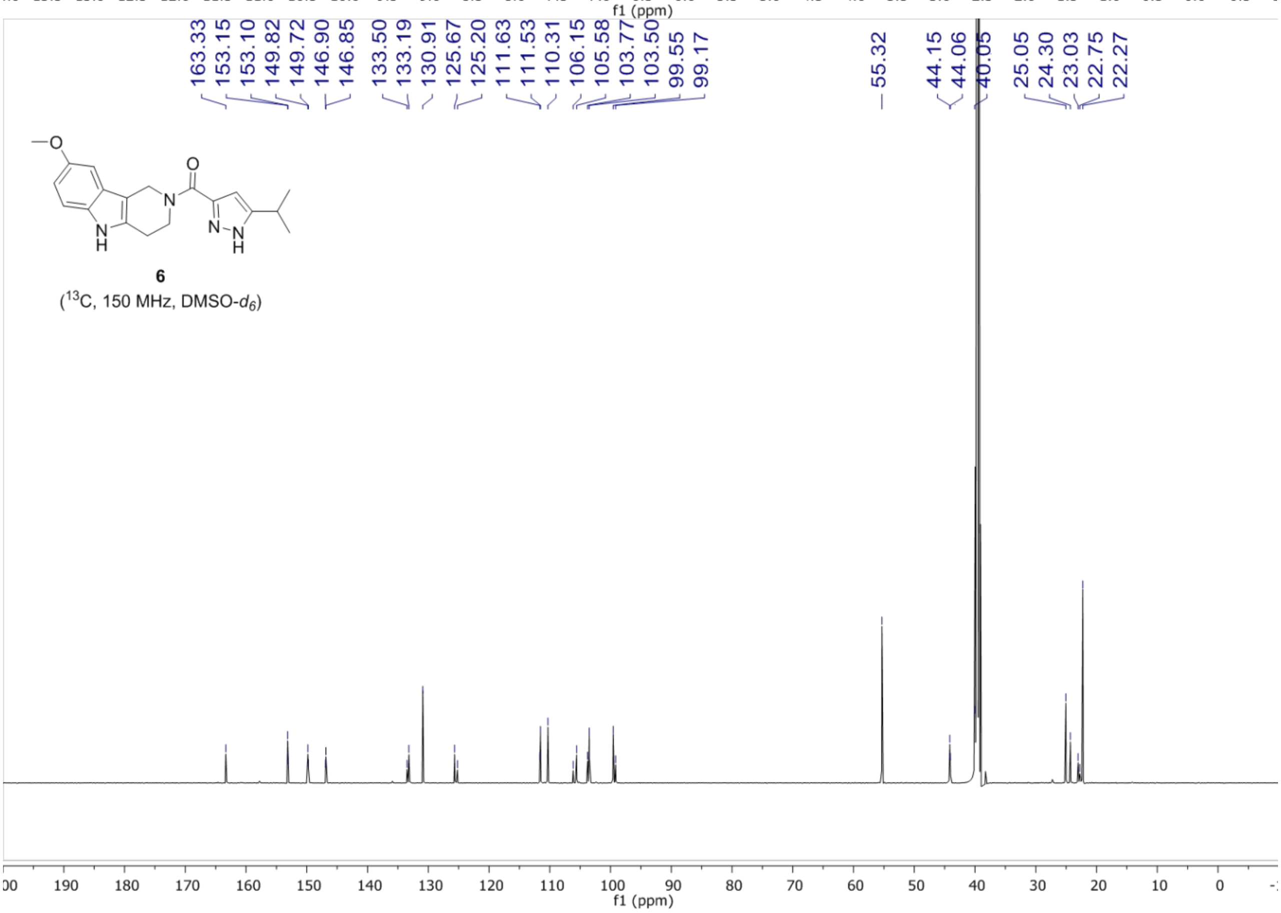
163.33
153.15
153.10
149.82
149.72
146.90
146.85
133.50
133.19
130.91
125.67
125.20
111.63
111.53
110.31
106.15
105.58
103.77
103.50
99.55
99.17
55.32
44.15
44.06
40.05
25.05
24.30
23.03
22.75
22.27
6
(13C, 150 MHz, DMSO-d6)
f1 (ppm)

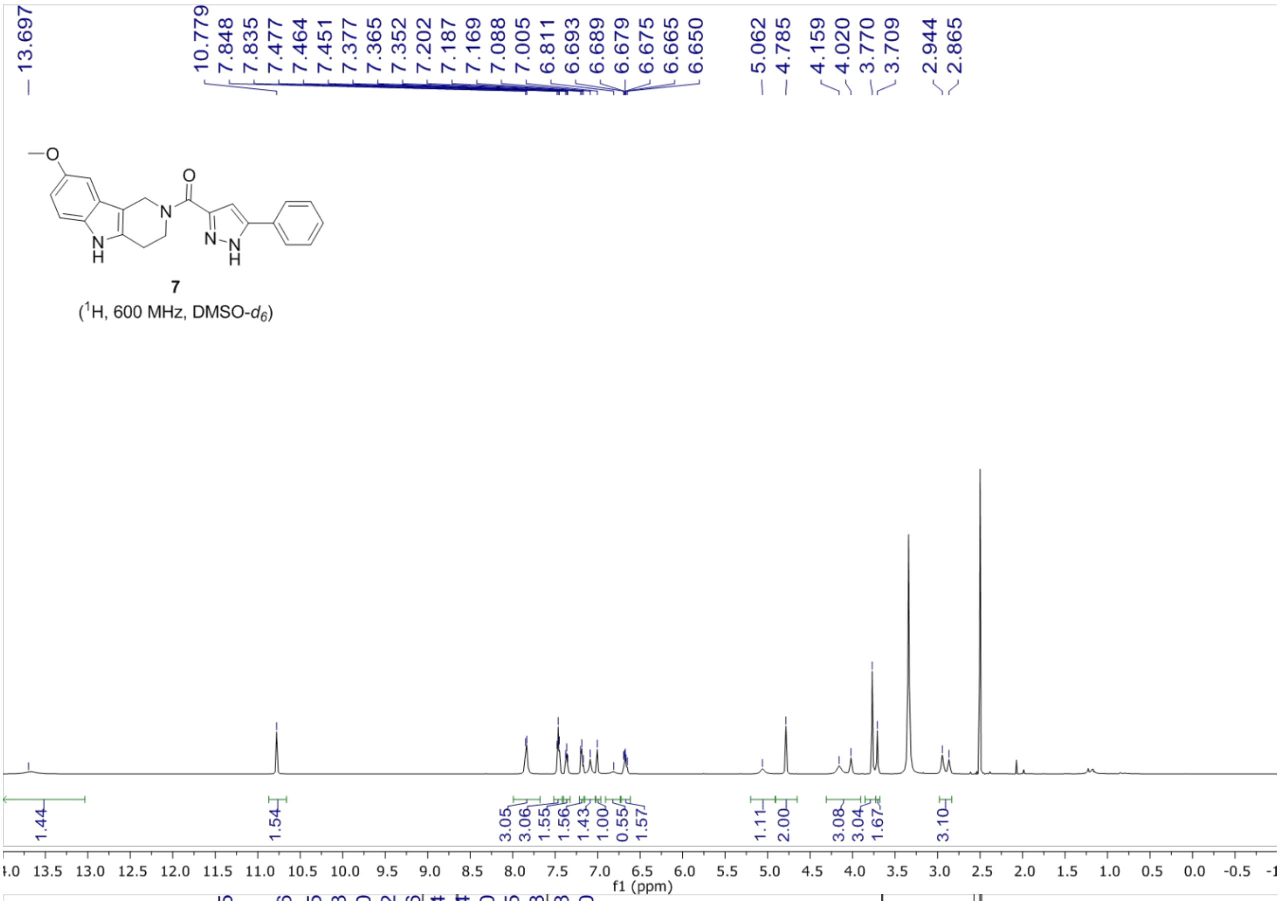

7

(^{1}H, 600 MHz, DMSO-d_6)

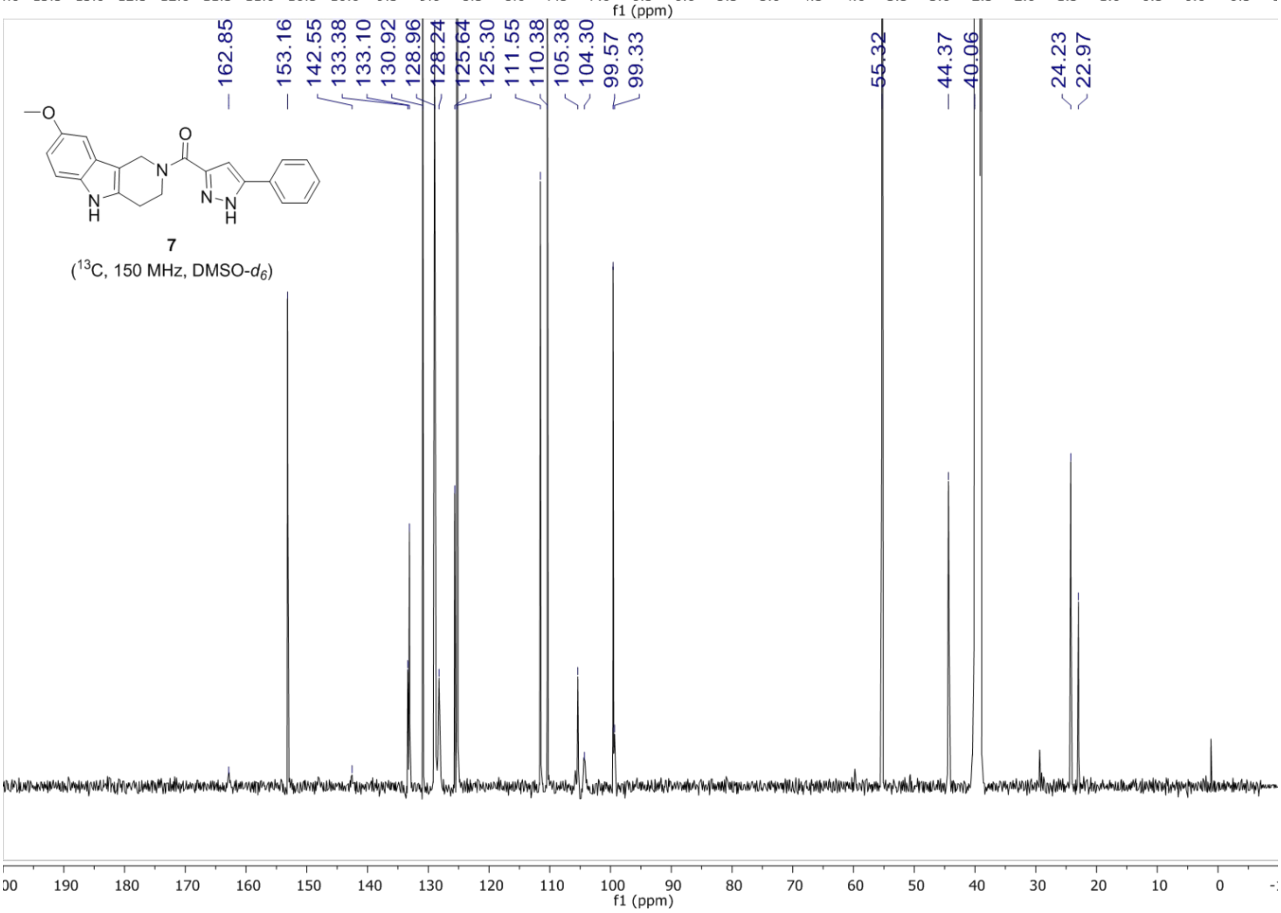

7

(^{13}C, 150 MHz, DMSO-d_6)

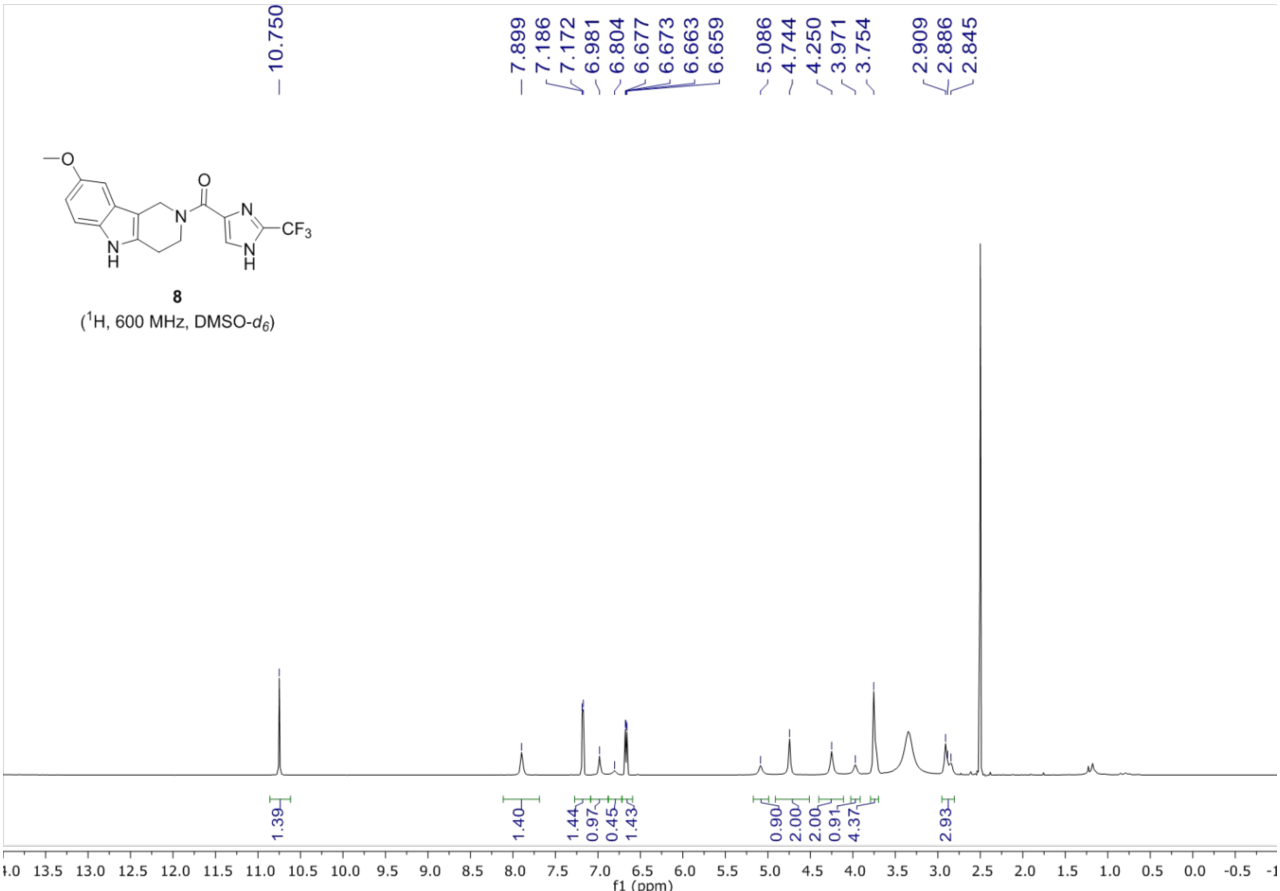
8
(1H, 600 MHz, DMSO-d6)
10.750
7.899
7.186
7.172
6.981
6.804
6.677
6.673
6.663
6.659
5.086
4.744
4.250
3.971
3.754
2.909
2.886
2.845
1.39
1.40
1.44
0.97
0.45
1.43
0.90
2.00
2.00
0.91
4.37
2.93
f1 (ppm)

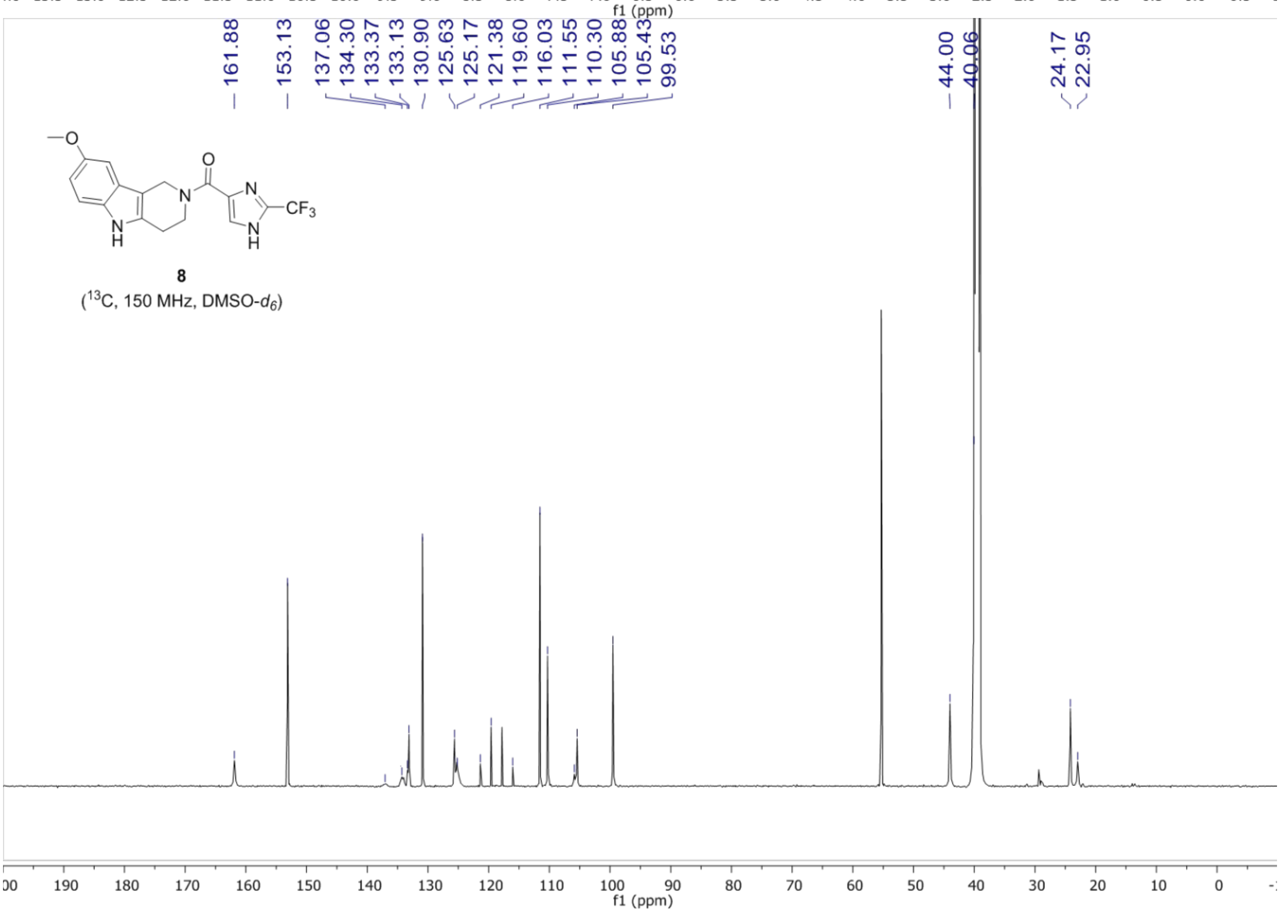
8
(13C, 150 MHz, DMSO-d6)
161.88
153.13
137.06
134.30
133.37
133.13
130.90
125.63
125.17
121.38
119.60
116.03
111.55
110.30
105.88
105.43
99.53
44.00
40.06
24.17
22.95
f1 (ppm)

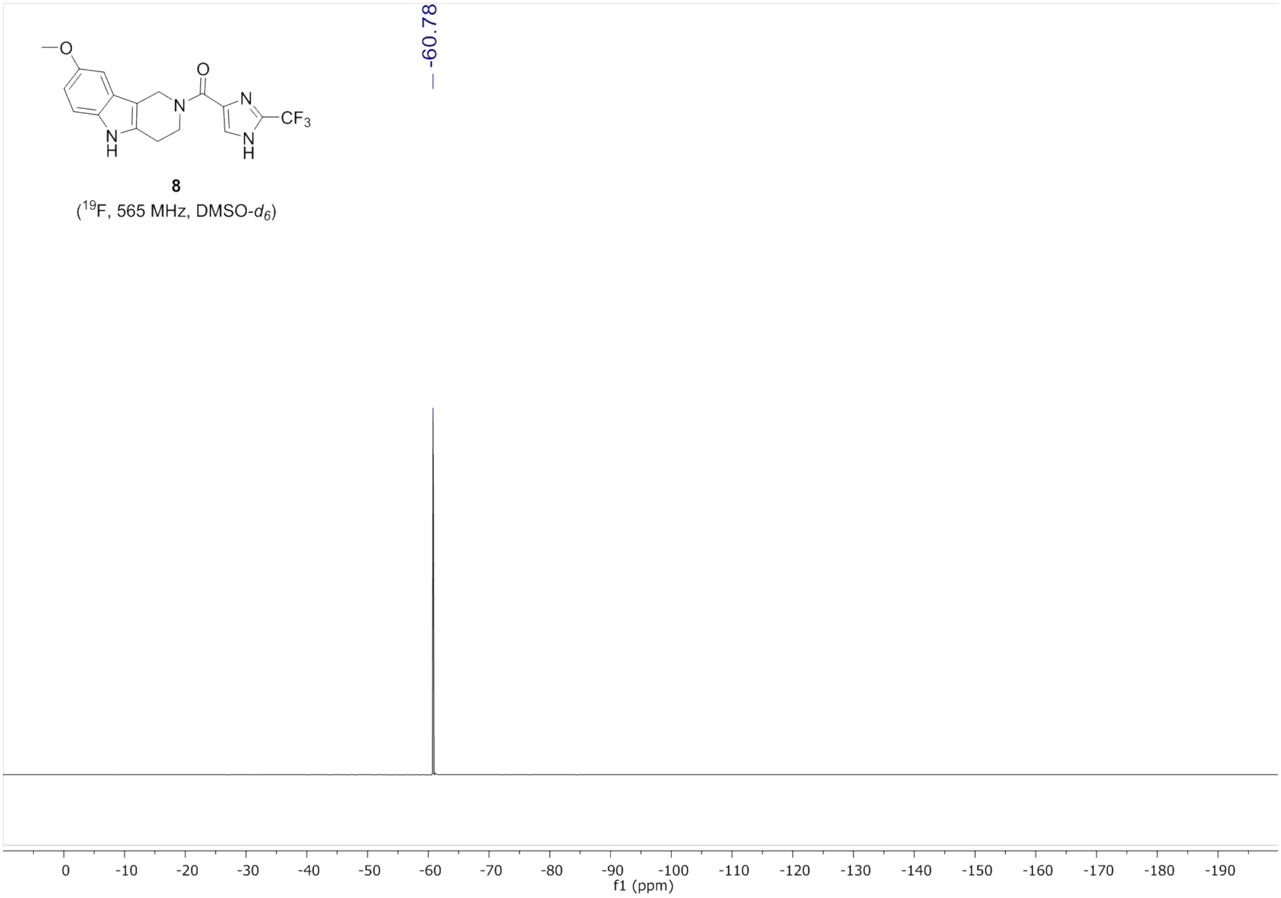

-60.78
O
O
N
N
CF3
N
H
N
H
8
(19F, 565 MHz, DMSO-d6)
0 -10 -20 -30 -40 -50 -60 -70 -80 -90 -100 -110 -120 -130 -140 -150 -160 -170 -180 -190
f1 (ppm)

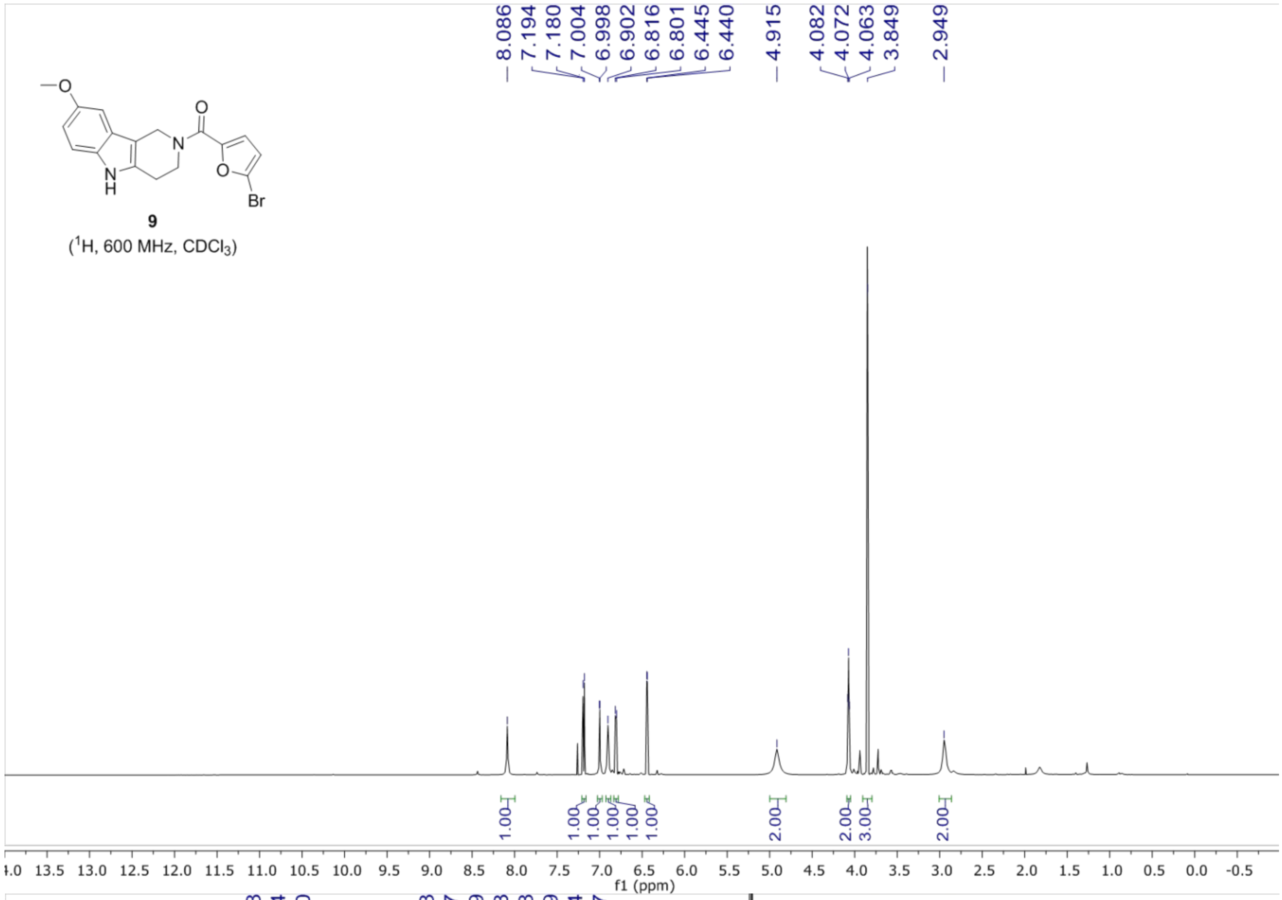
9
(^{1}H, 600 MHz, $CDCl_3$)
8.086 7.194 7.180 7.004 6.998 6.902 6.816 6.801 6.445 6.440 4.915 4.082 4.072 4.063 3.849 2.949
1.00 1.00 1.00 1.00 1.00 1.00 2.00 2.00 3.00 2.00
f1 (ppm)

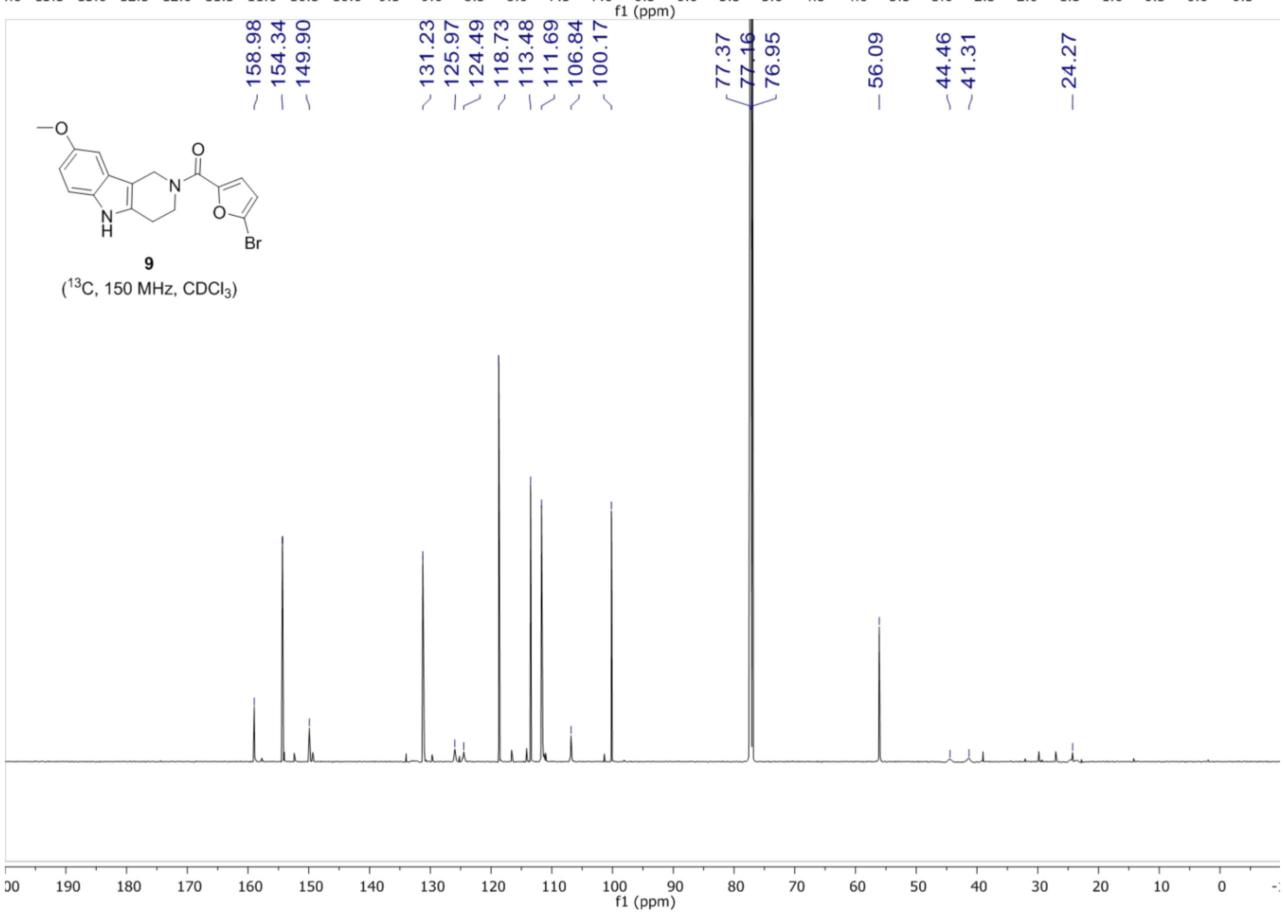
9
(^{13}C, 150 MHz, $CDCl_3$)
158.98 154.34 149.90 131.23 125.97 124.49 118.73 113.48 111.69 106.84 100.17 77.37 77.16 76.95 56.09 44.46 41.31 24.27
f1 (ppm)

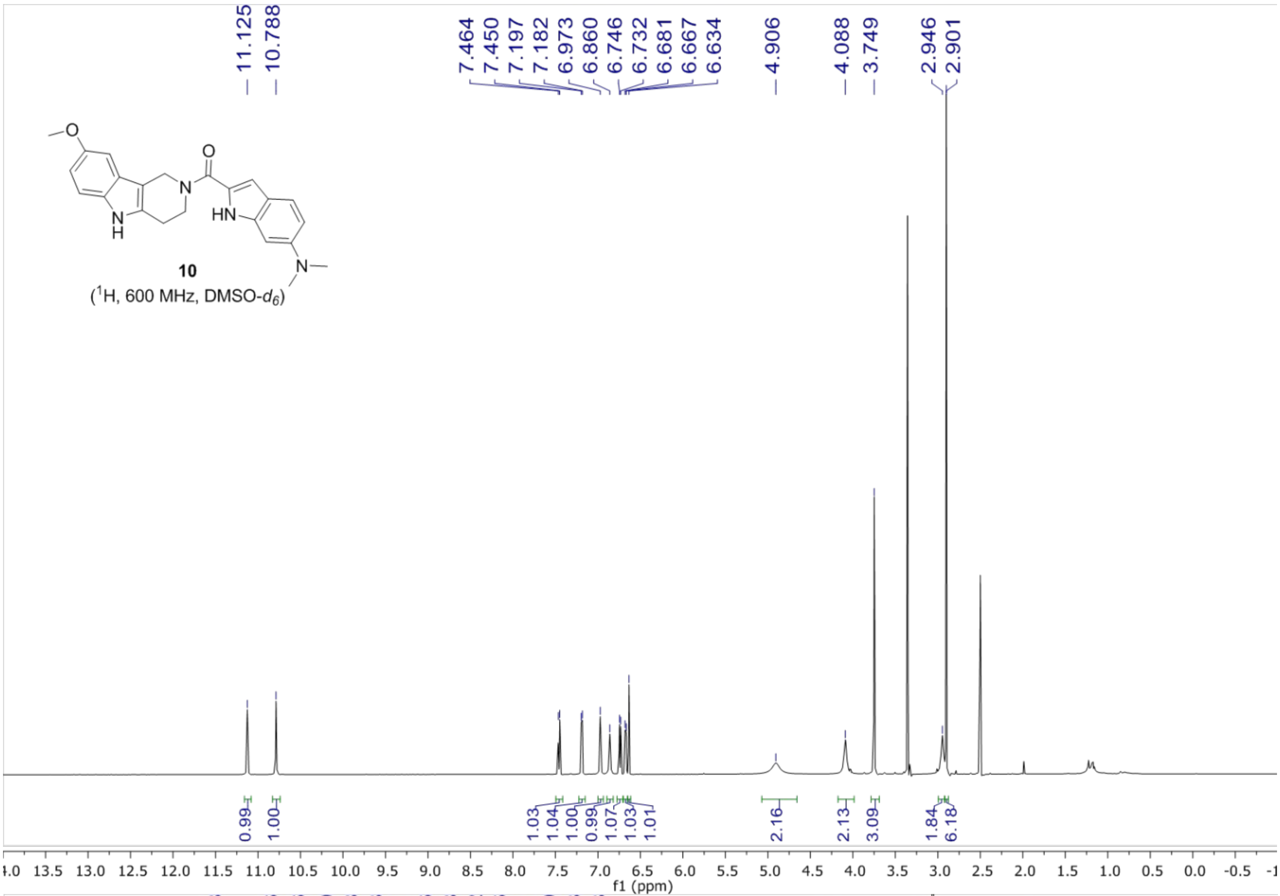
11.125
10.788
7.464
7.450
7.197
7.182
6.973
6.860
6.746
6.732
6.681
6.667
6.634
4.906
4.088
3.749
2.946
2.901
O
N
HN
N
H
N
10
(^{1}H, 600 MHz, DMSO-d_6)
0.99
1.00
1.03
1.04
1.00
0.99
1.07
1.03
1.01
2.16
2.13
3.09
1.84
6.18
f1 (ppm)

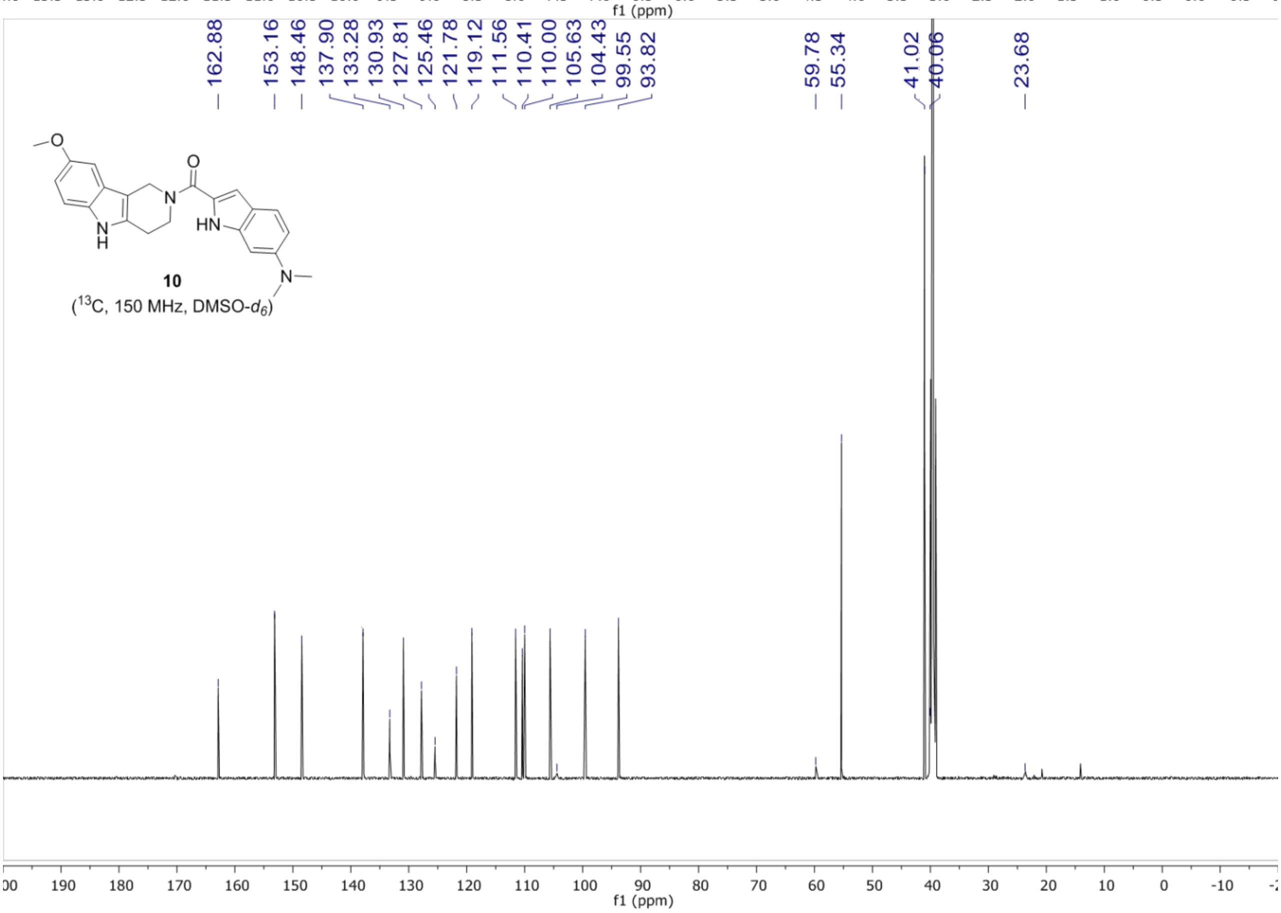
162.88
153.16
148.46
137.90
133.28
130.93
127.81
125.46
121.78
119.12
111.56
110.41
110.00
105.63
104.43
99.55
93.82
59.78
55.34
41.02
40.06
23.68
O
N
HN
N
H
N
10
(^{13}C, 150 MHz, DMSO-d_6)
f1 (ppm)

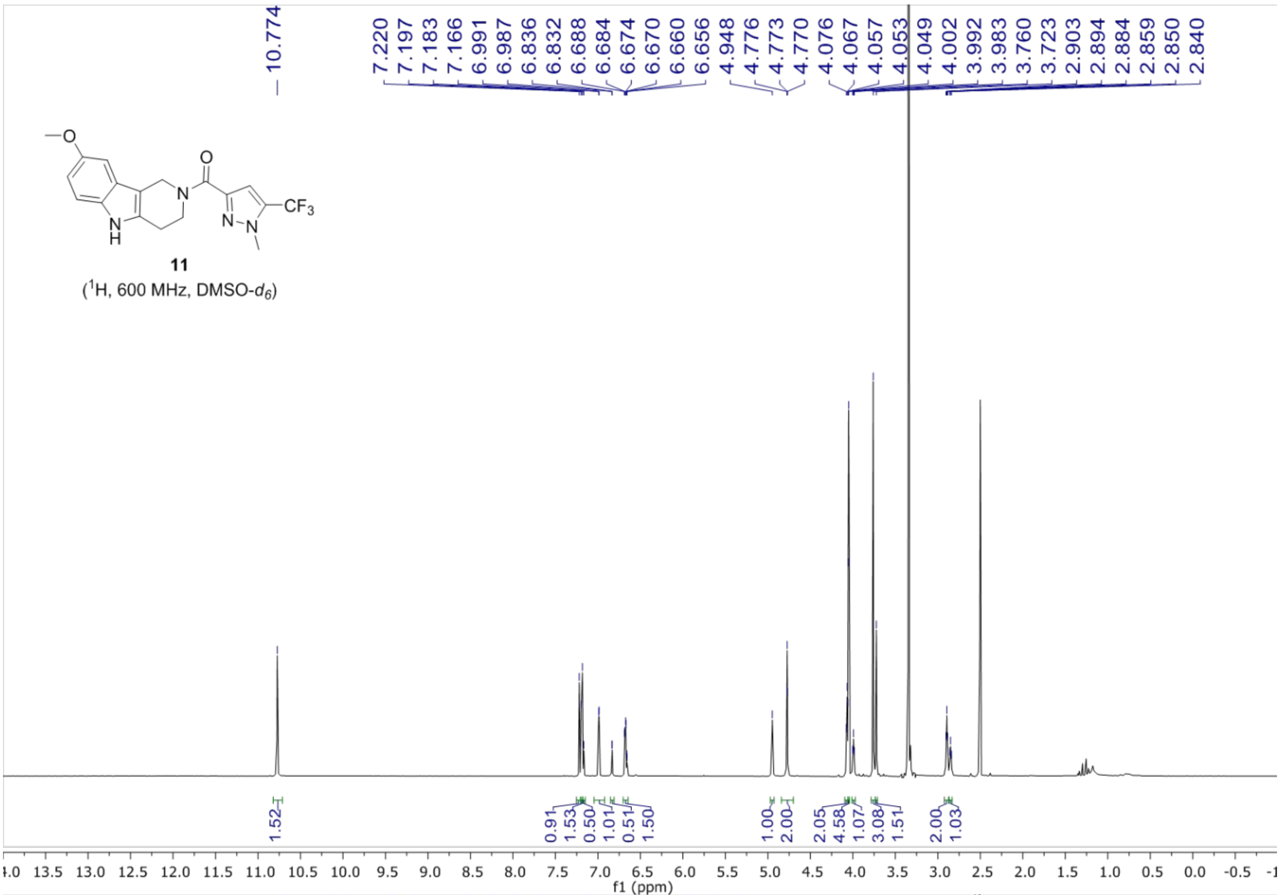

10.774
7.220
7.197
7.183
7.166
6.991
6.987
6.836
6.832
6.688
6.684
6.674
6.670
6.660
6.656
4.948
4.776
4.773
4.770
4.076
4.067
4.057
4.053
4.049
4.002
3.992
3.983
3.760
3.723
2.903
2.894
2.884
2.859
2.850
2.840
O
CF3
N
N
N
H
11
(1H, 600 MHz, DMSO-d6)
1.52
0.91
1.53
0.50
1.01
0.51
1.50
1.00
2.00
2.05
4.58
1.07
3.08
1.51
2.00
1.03
f1 (ppm)

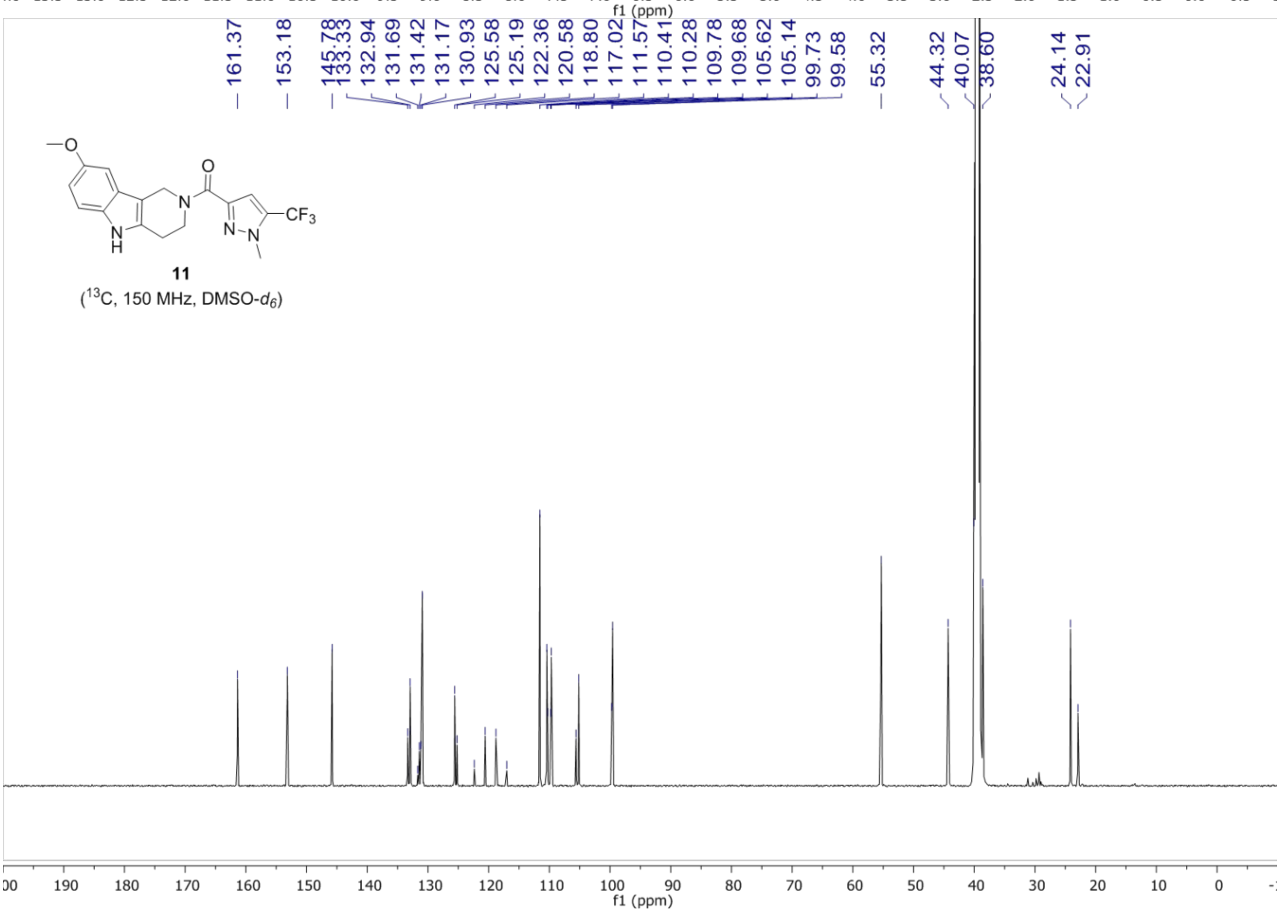

161.37
153.18
145.78
133.33
132.94
131.69
131.42
131.17
130.93
125.58
125.19
122.36
120.58
118.80
117.02
111.57
110.41
110.28
109.78
109.68
105.62
105.14
99.73
99.58
55.32
44.32
40.07
38.60
24.14
22.91
O
CF3
N
N
N
H
11
(13C, 150 MHz, DMSO-d6)
f1 (ppm)

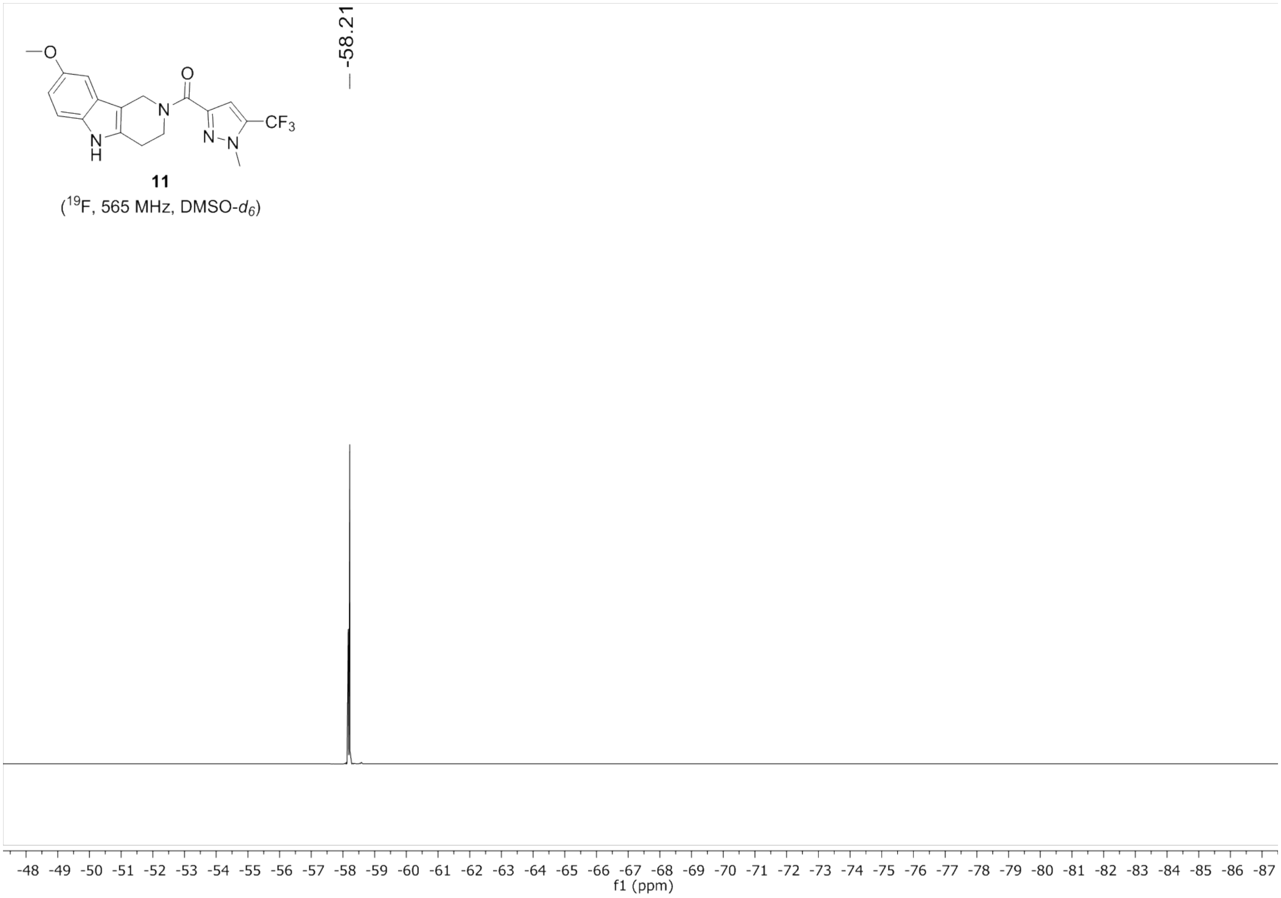
CF3
11
(19F, 565 MHz, DMSO-d6)
-58.21
-48 -49 -50 -51 -52 -53 -54 -55 -56 -57 -58 -59 -60 -61 -62 -63 -64 -65 -66 -67 -68 -69 -70 -71 -72 -73 -74 -75 -76 -77 -78 -79 -80 -81 -82 -83 -84 -85 -86 -87
f1 (ppm)

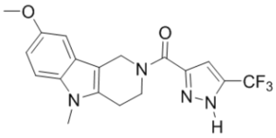

12

(^{1}H, 600 MHz, DMSO-d_6)

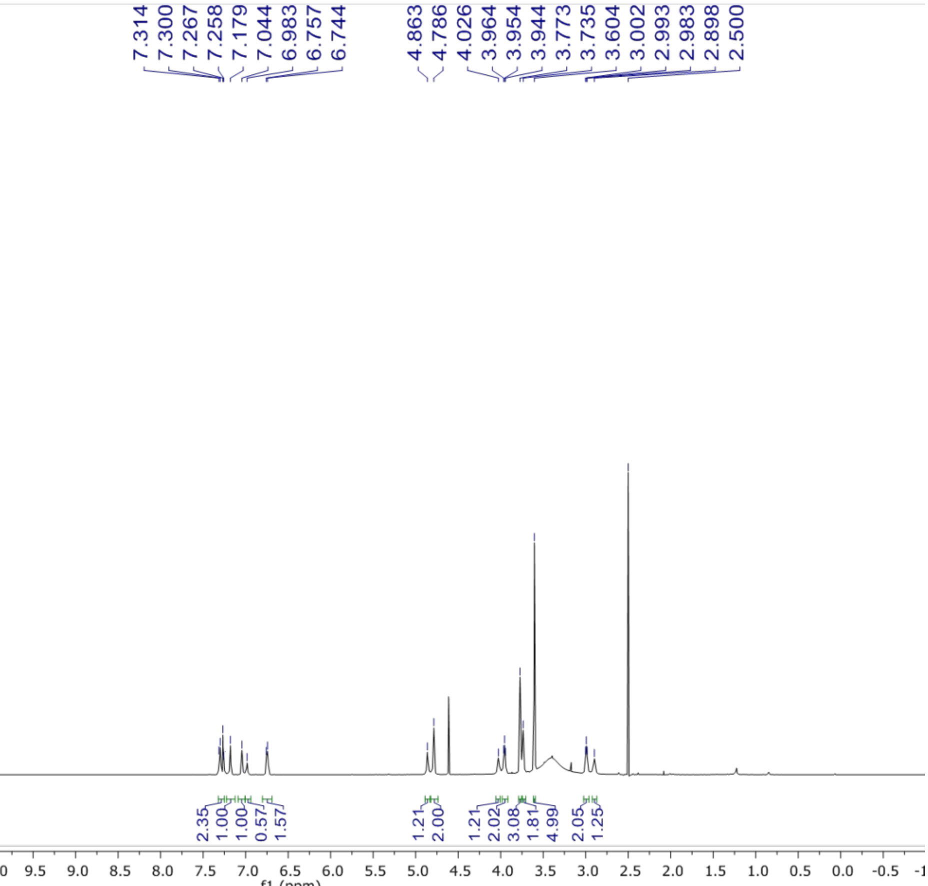

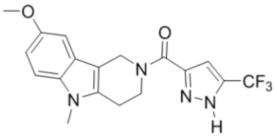

12

(^{13}C, 150 MHz, DMSO-d_6)

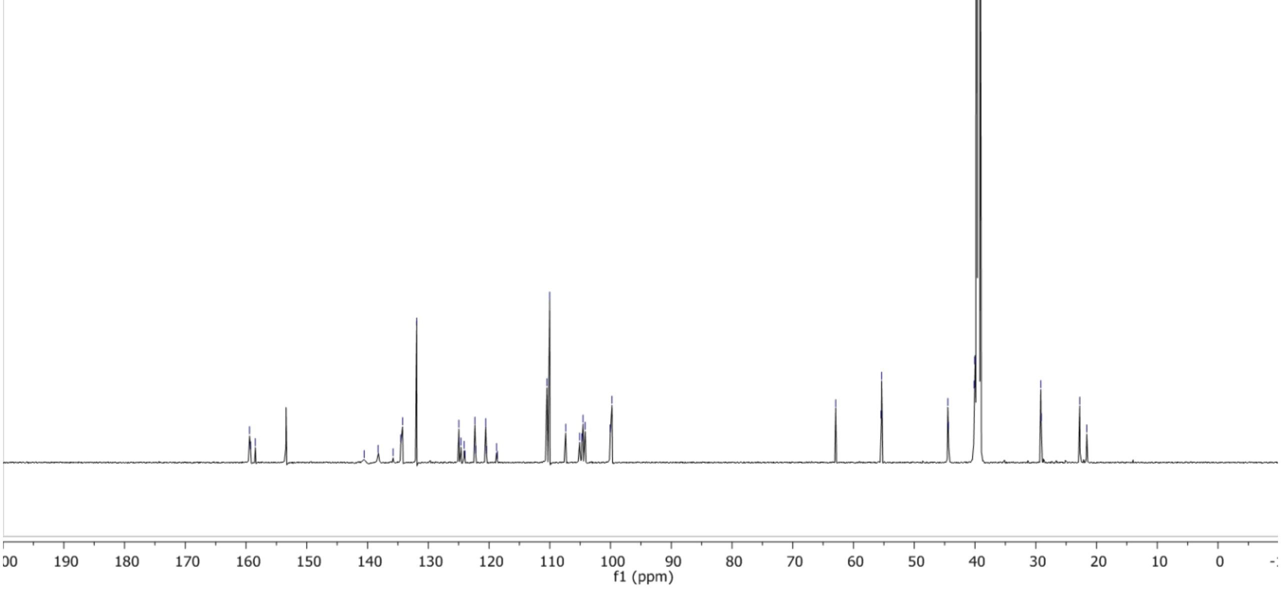

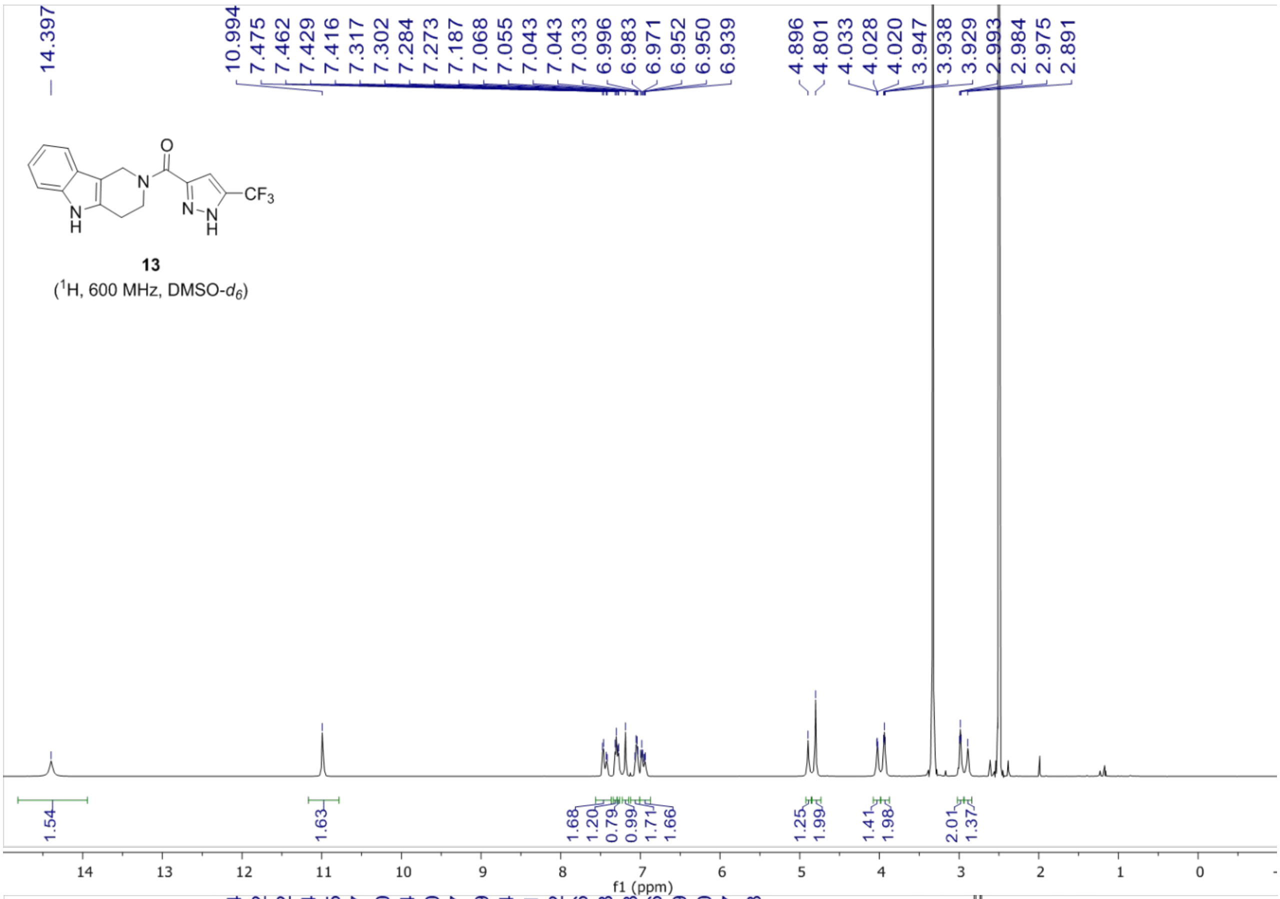
14.397
10.994 7.475 7.462 7.429 7.416 7.317 7.302 7.284 7.273 7.187 7.068 7.055 7.043 7.043 7.033 6.996 6.983 6.971 6.952 6.950 6.939
4.896 4.801 4.033 4.028 4.020 3.947 3.938 3.929 2.993 2.984 2.975 2.891
O
N
CF3
N
H
N
H
13
(1H, 600 MHz, DMSO-d6)
1.54
1.63
1.68 1.20 0.79 0.99 1.71 1.66
1.25 1.99
1.41 1.98
2.01 1.37
14 13 12 11 10 9 8 7 6 5 4 3 2 1 0
f1 (ppm)

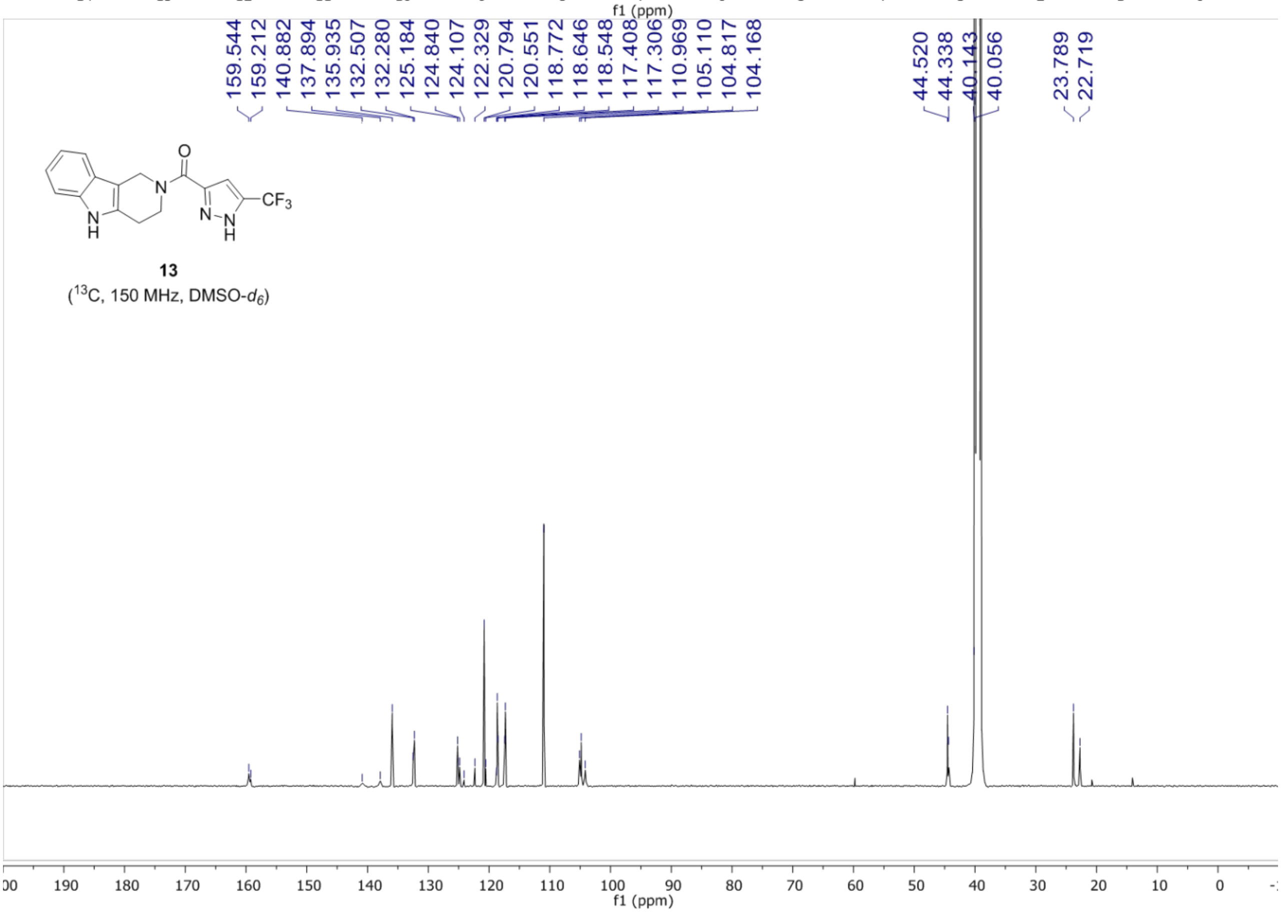
159.544 159.212 140.882 137.894 135.935 132.507 132.280 125.184 124.840 124.107 122.329 120.794 120.551 118.772 118.646 118.548 117.408 117.306 110.969 105.110 104.817 104.168
44.520 44.338 40.143 40.056
23.789 22.719
O
N
CF3
N
H
N
H
13
(13C, 150 MHz, DMSO-d6)
190 180 170 160 150 140 130 120 110 100 90 80 70 60 50 40 30 20 10 0
f1 (ppm)

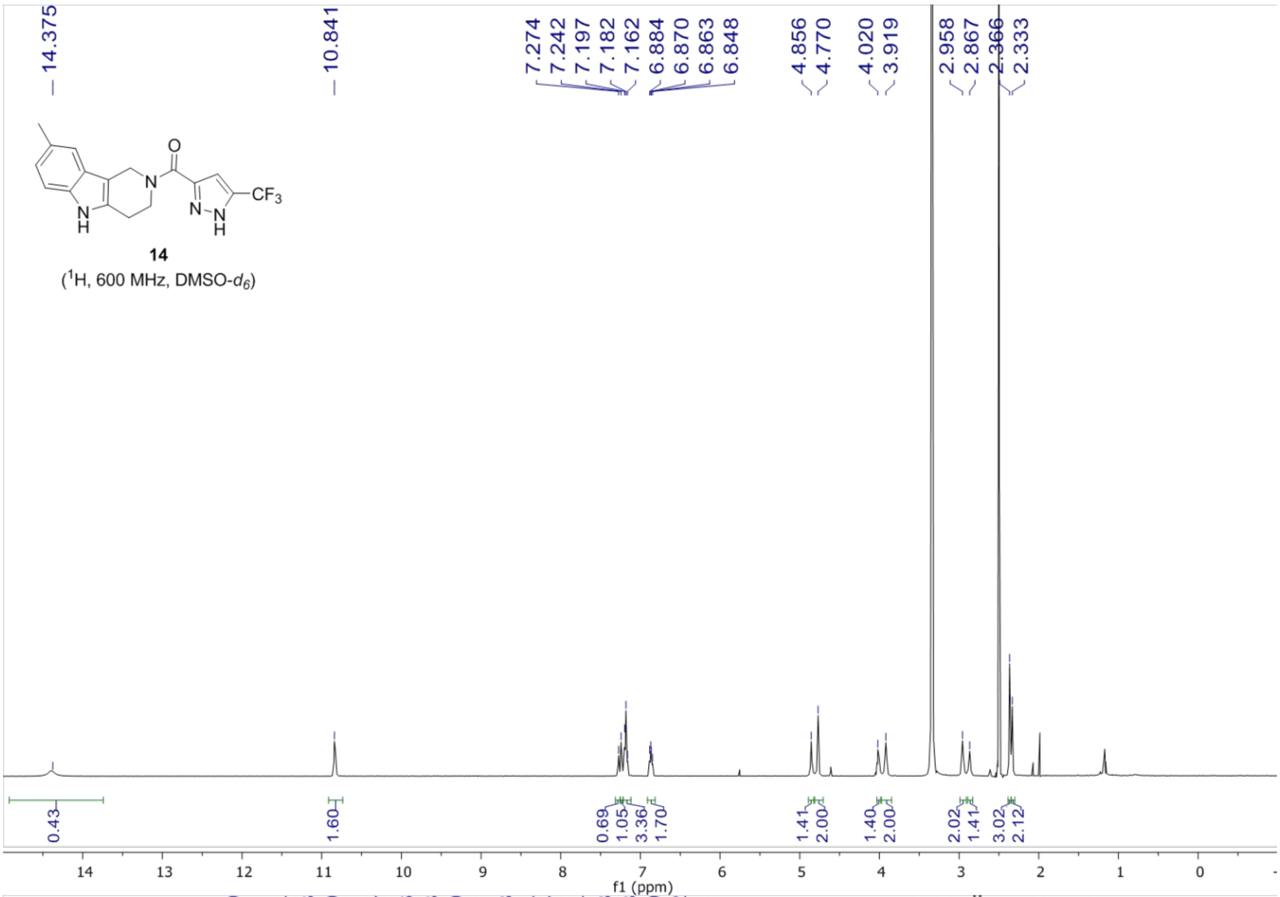
14.375
10.841
7.274 7.242 7.197 7.182 7.162 6.884 6.870 6.863 6.848
4.856 4.770
4.020 3.919
2.958 2.867 2.366 2.333
CF3
N
O
N
H
N
N
H
14
(1H, 600 MHz, DMSO-d6)
0.43
1.60
0.69 1.05 3.36 1.70
1.41 2.00
1.40 2.00
2.02 1.41
3.02 2.12
14 13 12 11 10 9 8 7 6 5 4 3 2 1 0
f1 (ppm)

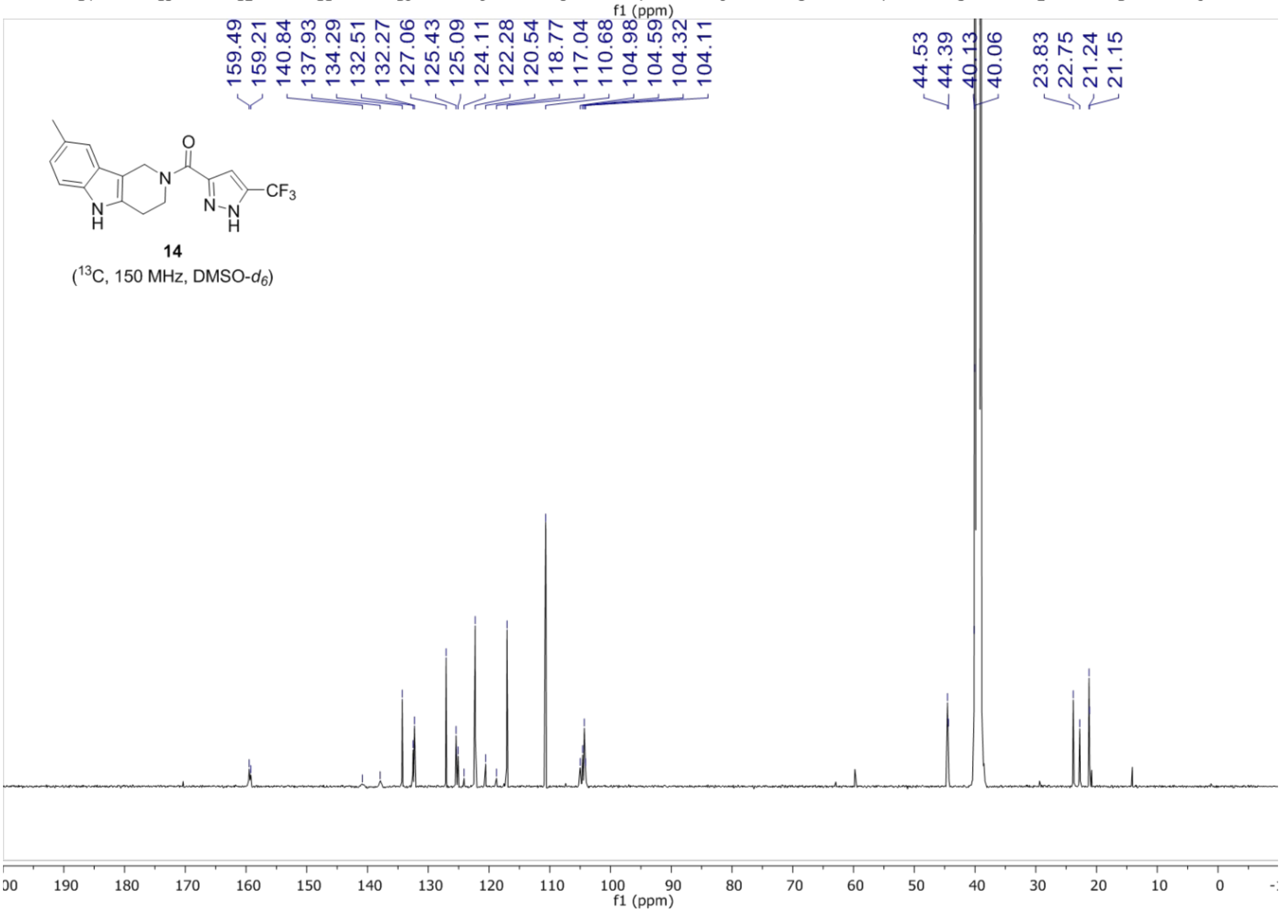
159.49 159.21 140.84 137.93 134.29 132.51 132.27 127.06 125.43 125.09 124.11 122.28 120.54 118.77 117.04 110.68 104.98 104.59 104.32 104.11
44.53 44.39 40.13 40.06
23.83 22.75 21.24 21.15
CF3
N
O
N
H
N
N
H
14
(13C, 150 MHz, DMSO-d6)
190 180 170 160 150 140 130 120 110 100 90 80 70 60 50 40 30 20 10 0
f1 (ppm)

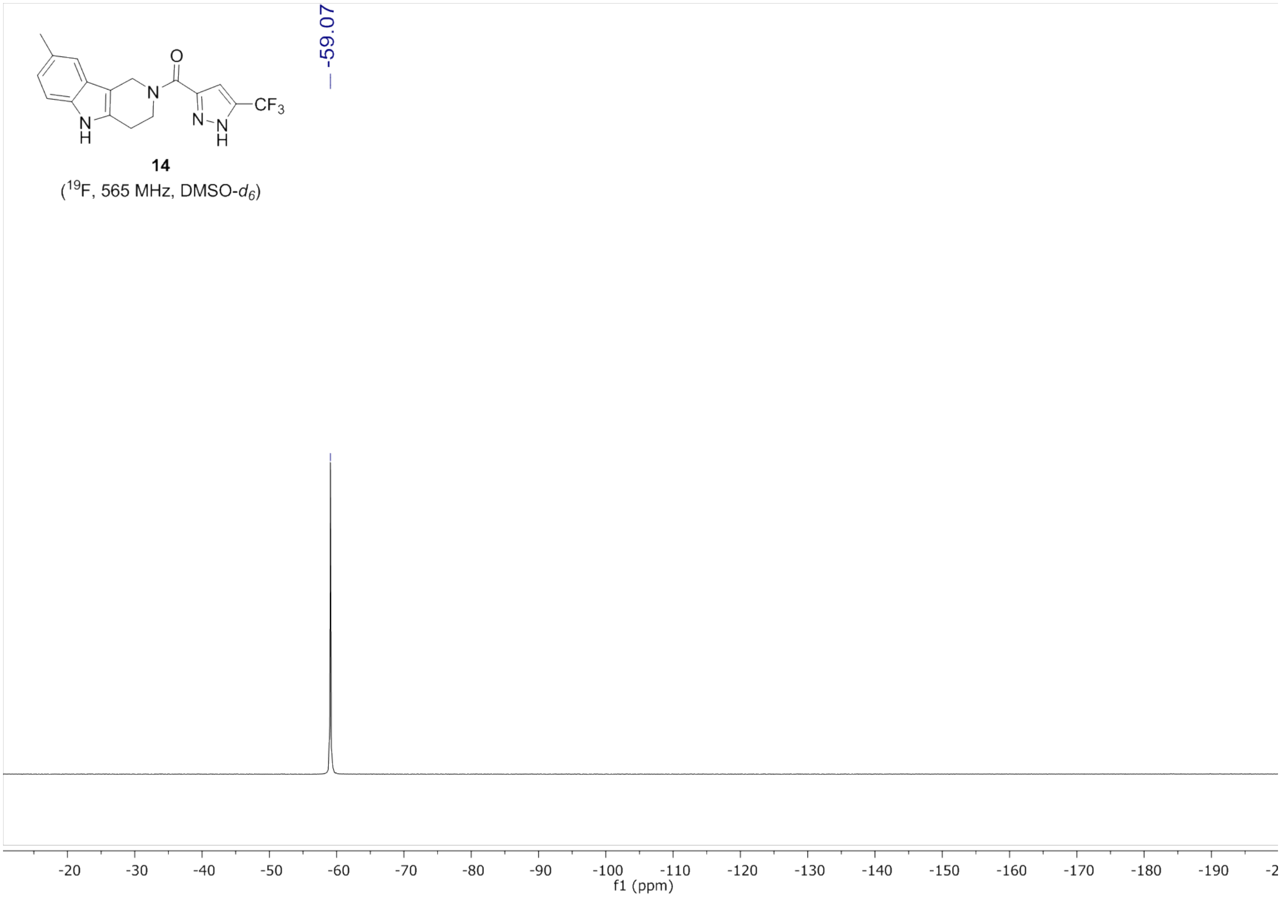

14

(^{19}F, 565 MHz, DMSO-d_6)

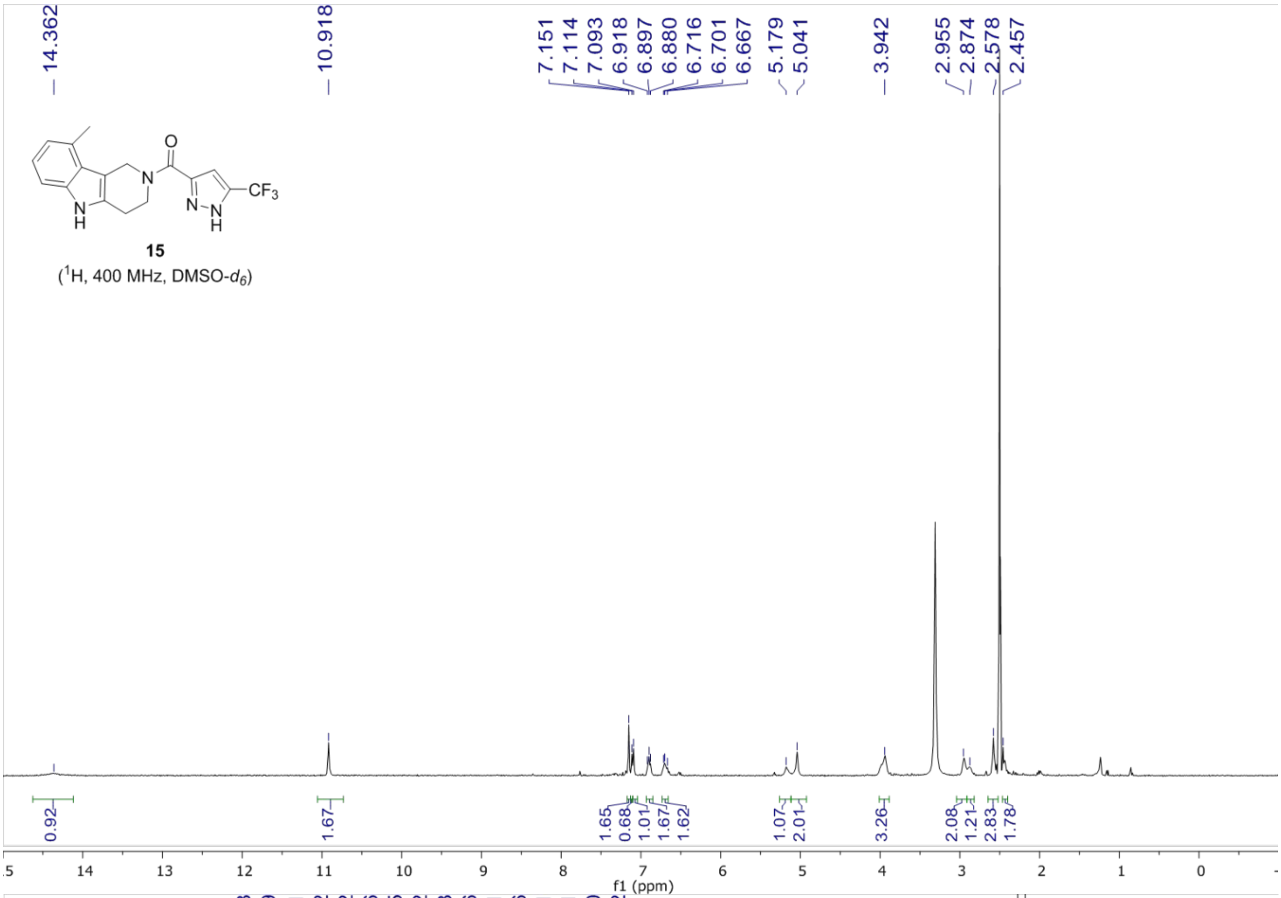
14.362
10.918
7.151
7.114
7.093
6.918
6.897
6.880
6.716
6.701
6.667
5.179
5.041
3.942
2.955
2.874
2.578
2.457
O
N
CF3
NH
N
H
15
(1H, 400 MHz, DMSO-d6)
0.92
1.67
1.65
0.68
1.01
1.67
1.62
1.07
2.01
3.26
2.08
1.21
2.83
1.78
f1 (ppm)

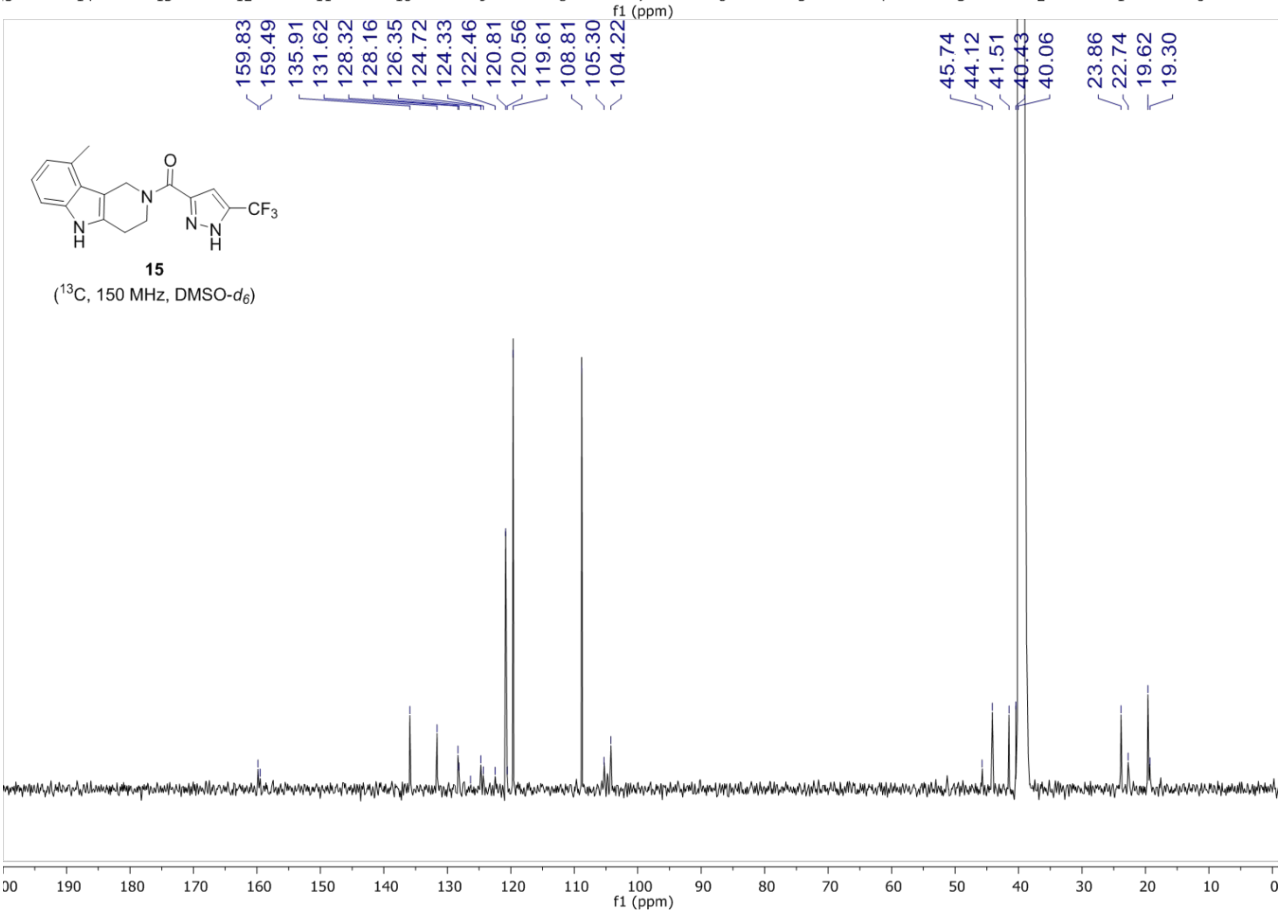
159.83
159.49
135.91
131.62
128.32
128.16
126.35
124.72
124.33
122.46
120.81
120.56
119.61
108.81
105.30
104.22
45.74
44.12
41.51
40.43
40.06
23.86
22.74
19.62
19.30
O
N
CF3
NH
N
H
15
(13C, 150 MHz, DMSO-d6)
f1 (ppm)

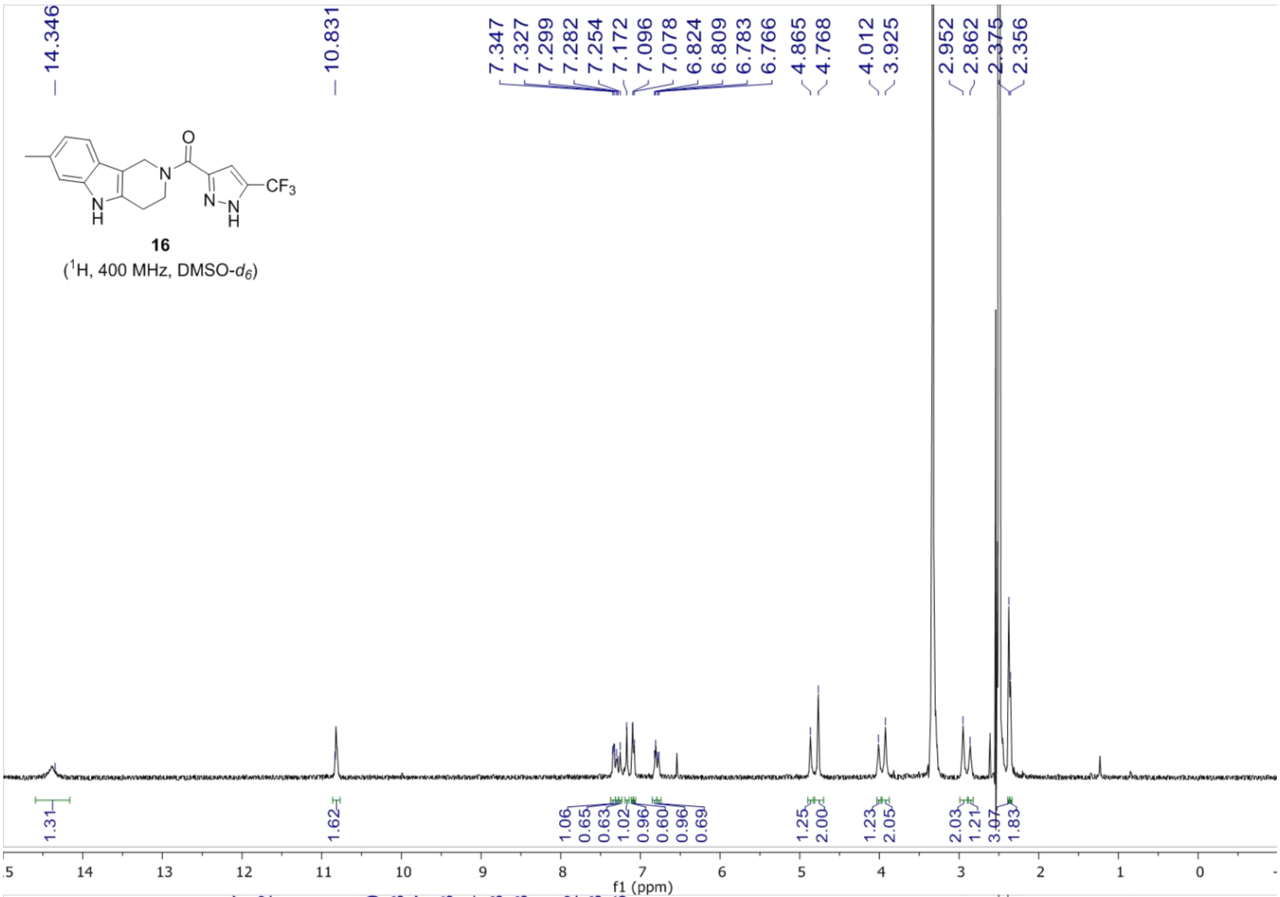
14.346
10.831
7.347
7.327
7.299
7.282
7.254
7.172
7.096
7.078
6.824
6.809
6.783
6.766
4.865
4.768
4.012
3.925
2.952
2.862
2.375
2.356
O
CF3
N
N
N
H
H
16
(1H, 400 MHz, DMSO-d6)
1.31
1.62
1.06
0.65
0.63
1.02
0.96
0.60
0.96
0.69
1.25
2.00
1.23
2.05
2.03
1.21
3.07
1.83
f1 (ppm)

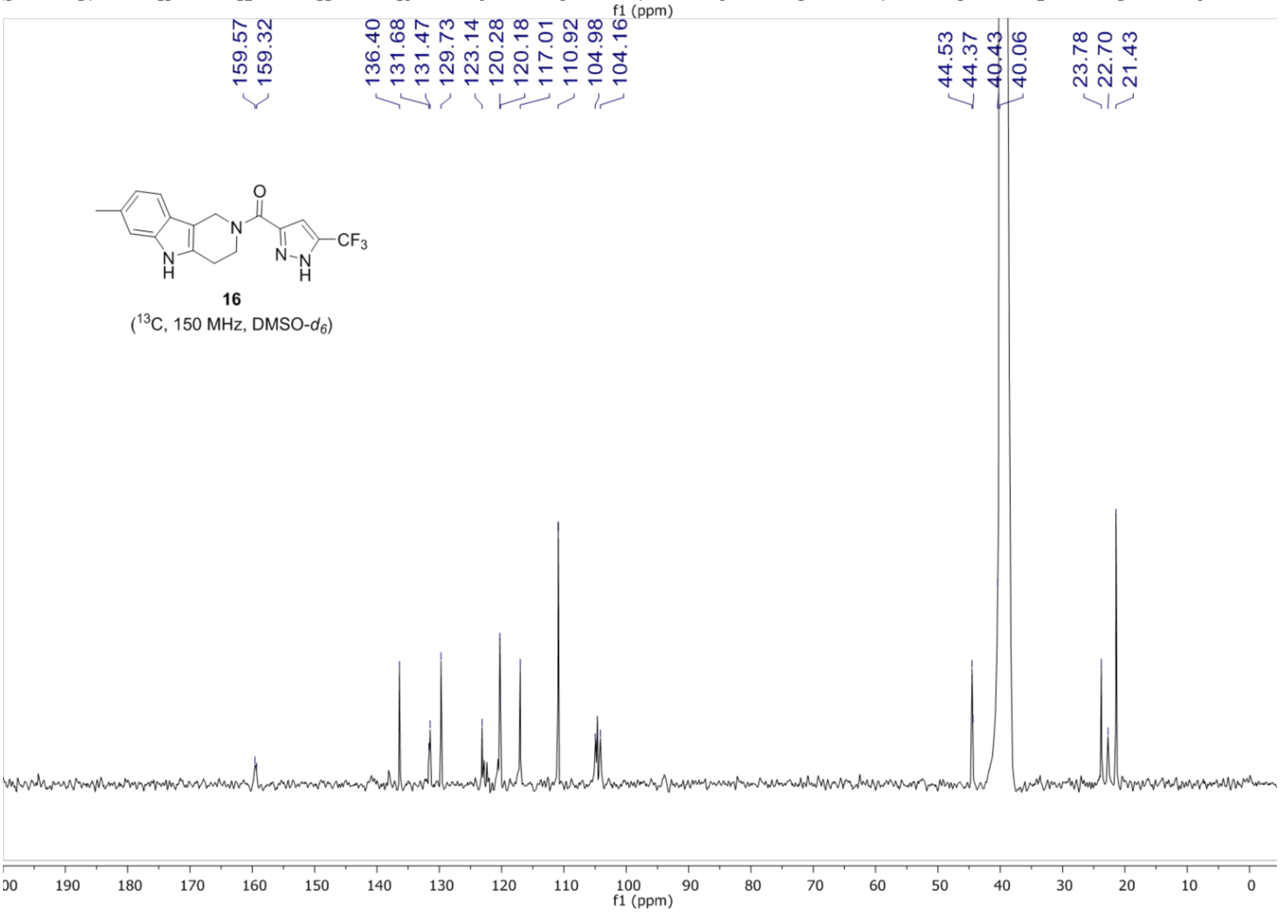
159.57
159.32
136.40
131.68
131.47
129.73
123.14
120.28
120.18
117.01
110.92
104.98
104.16
44.53
44.37
40.43
40.06
23.78
22.70
21.43
O
CF3
N
N
N
H
H
16
(13C, 150 MHz, DMSO-d6)
f1 (ppm)

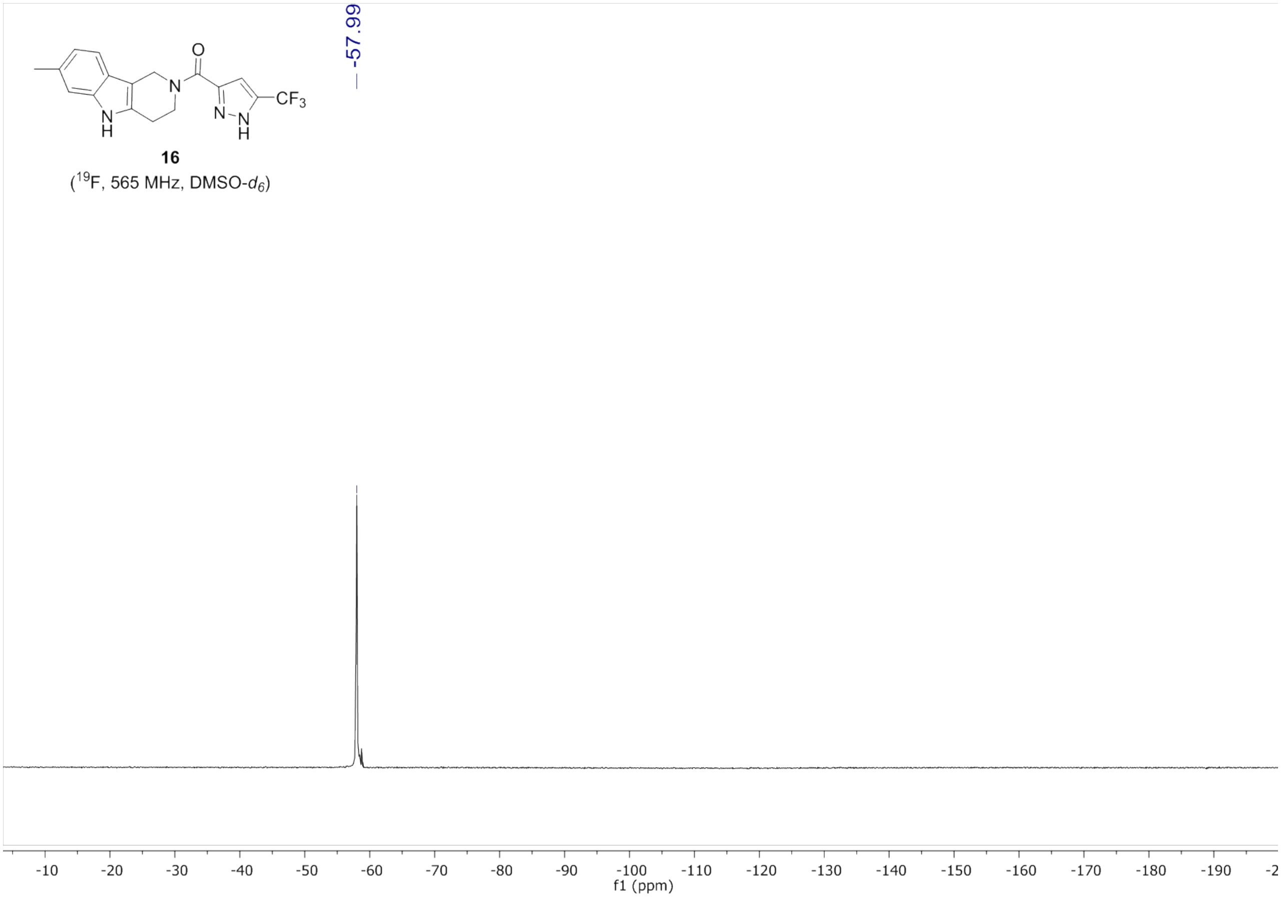

CF3
N
H
N
O
N
H
16
(19F, 565 MHz, DMSO-d6)
-57.99
-10
-20
-30
-40
-50
-60
-70
-80
-90
-100
-110
-120
-130
-140
-150
-160
-170
-180
-190
f1 (ppm)

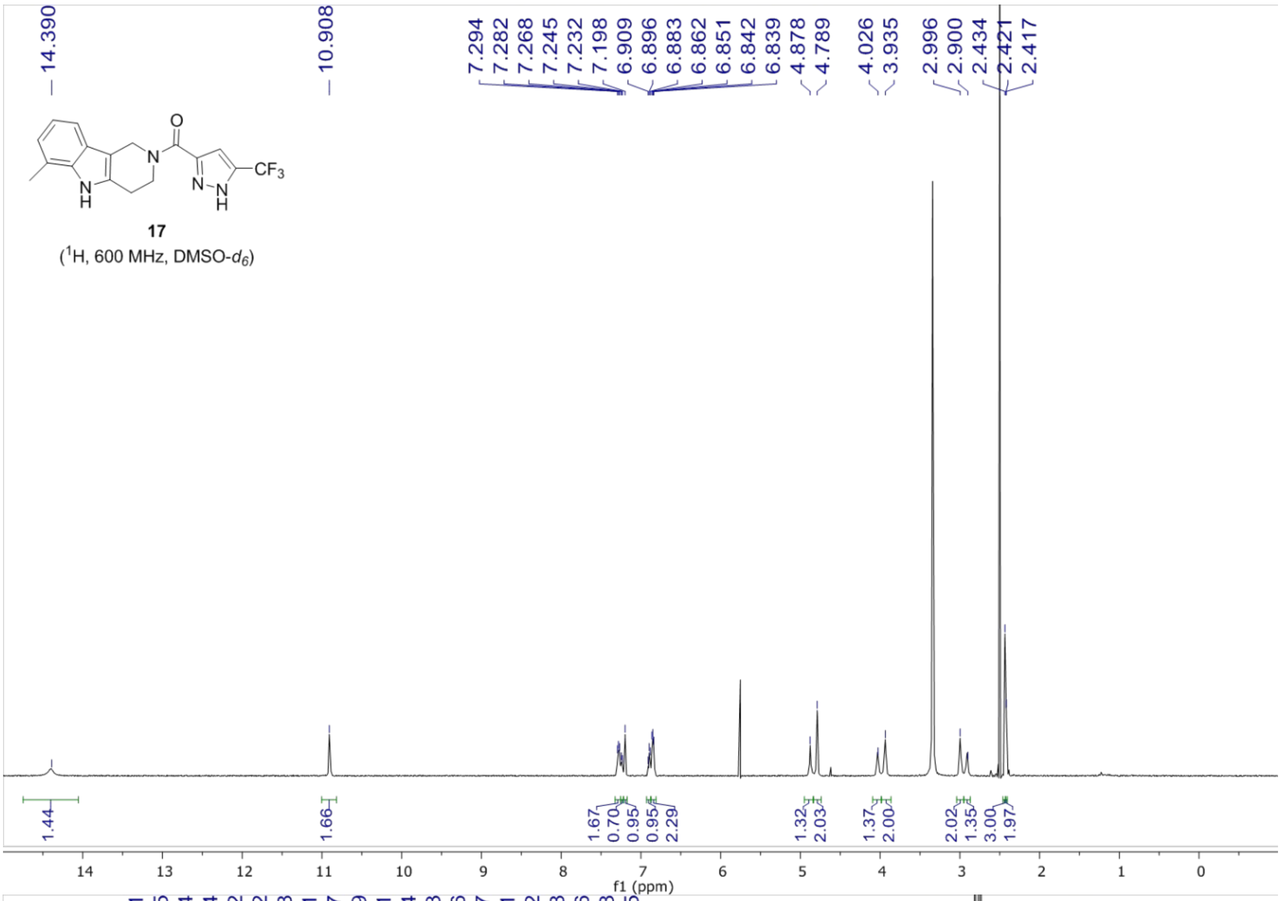
14.390
10.908
7.294 7.282 7.268 7.245 7.232 7.198 6.909 6.896 6.883 6.862 6.851 6.842 6.839 4.878 4.789
4.026 3.935
2.996 2.900 2.434 2.421 2.417
O
N
CF3
N
H
N-N
H
17
(1H, 600 MHz, DMSO-d6)
1.44
1.66
1.67 0.70 0.95 0.95 2.29
1.32 2.03
1.37 2.00
2.02 1.35 3.00 1.97
f1 (ppm)

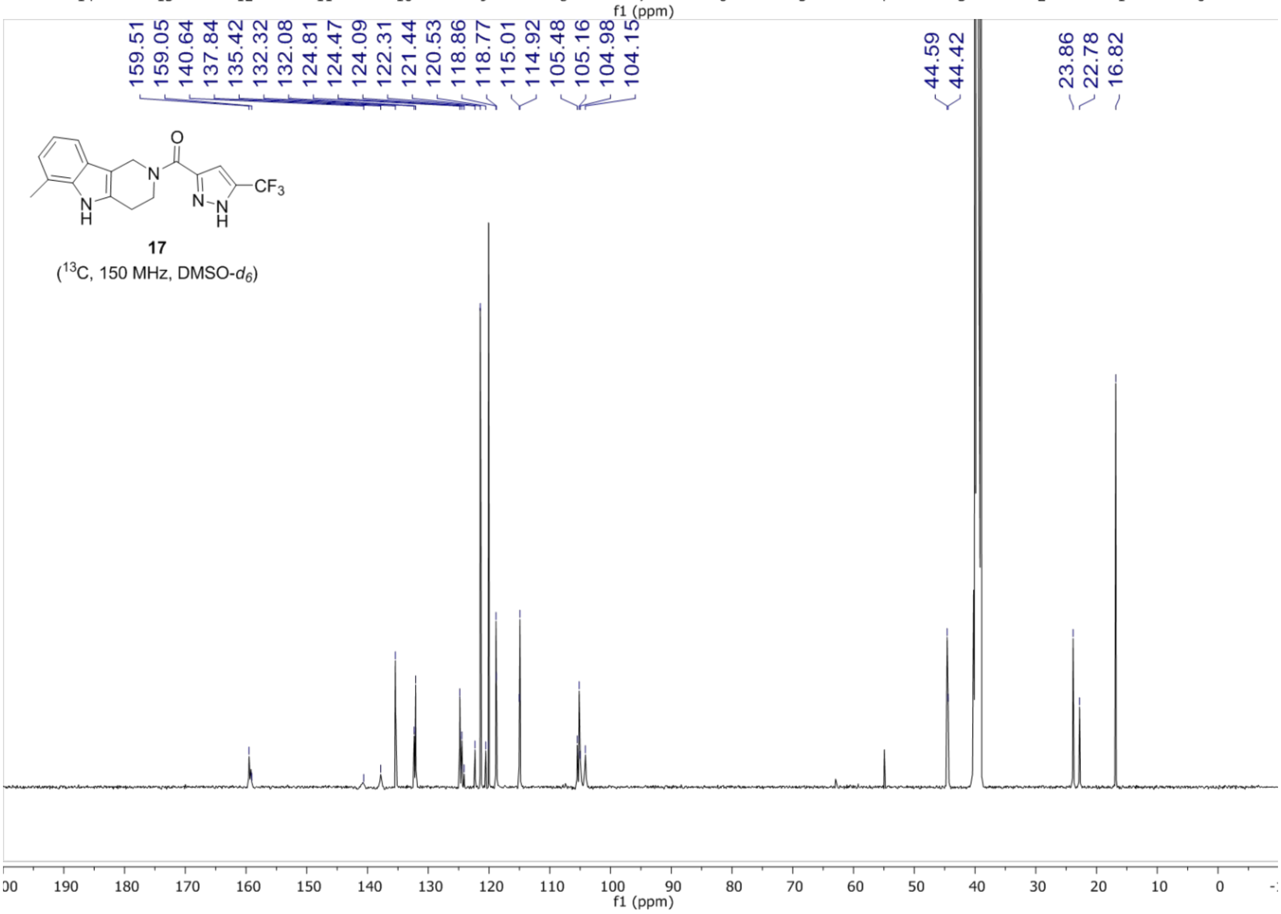
159.51 159.05 140.64 137.84 135.42 132.32 132.08 124.81 124.47 124.09 122.31 121.44 120.53 118.86 118.77 115.01 114.92 105.48 105.16 104.98 104.15
44.59 44.42
23.86 22.78 16.82
17
(13C, 150 MHz, DMSO-d6)
f1 (ppm)

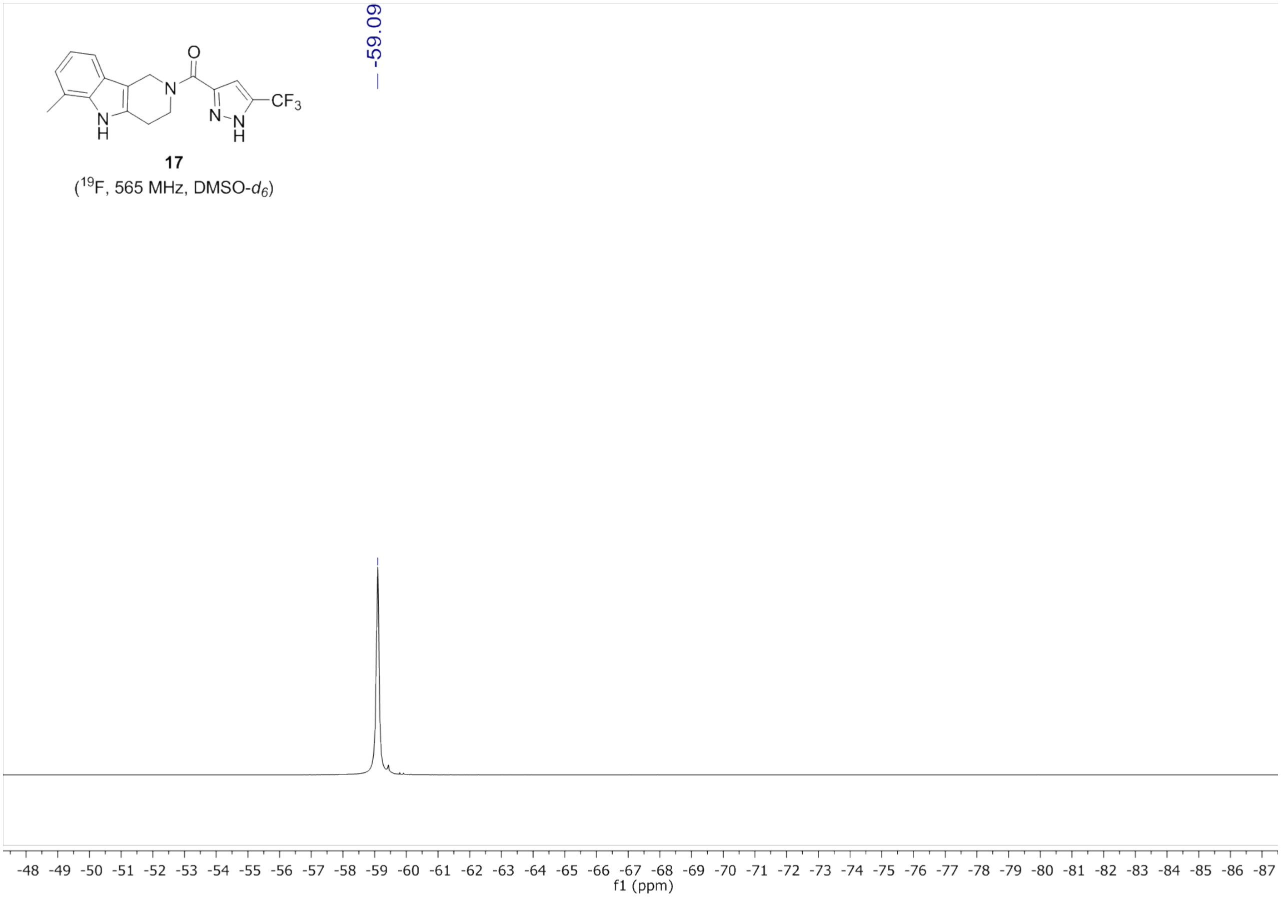

17

(^{19}F, 565 MHz, DMSO-d_6)

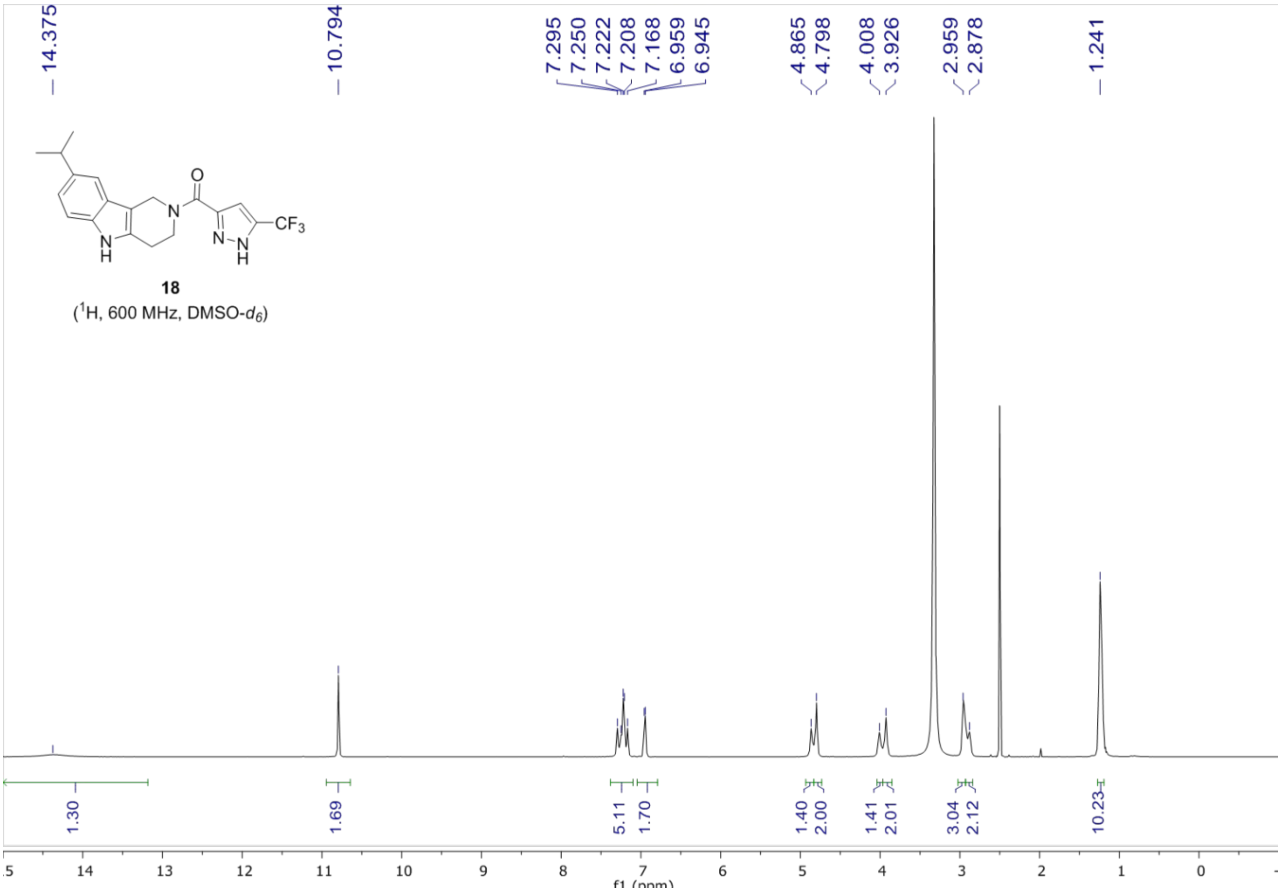
14.375
10.794
7.295
7.250
7.222
7.208
7.168
6.959
6.945
4.865
4.798
4.008
3.926
2.959
2.878
1.241
CF3
N
O
NH
N
H
18
(1H, 600 MHz, DMSO-d6)
1.30
1.69
5.11
1.70
1.40
2.00
1.41
2.01
3.04
2.12
10.23
f1 (ppm)

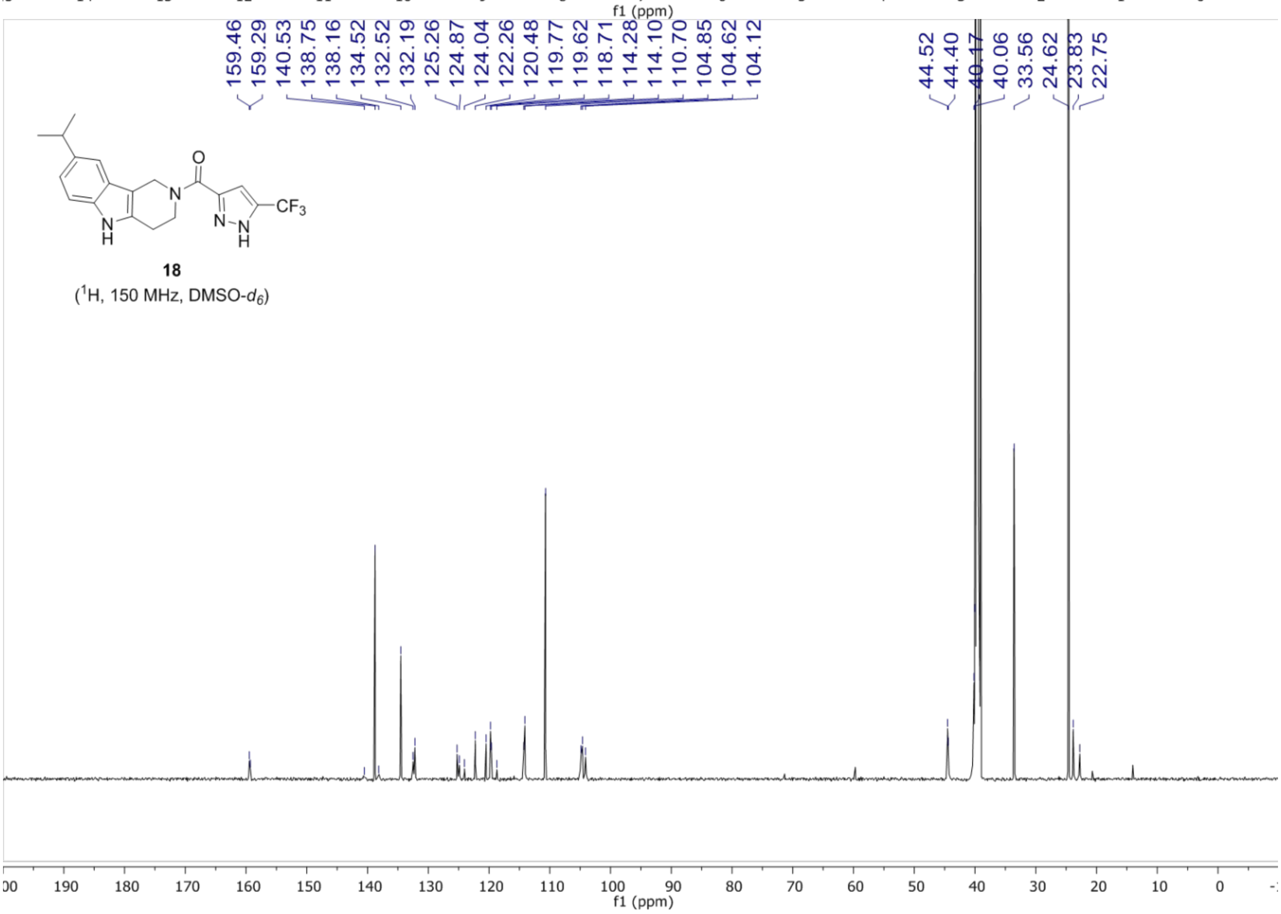
159.46
159.29
140.53
138.75
138.16
134.52
132.52
132.19
125.26
124.87
124.04
122.26
120.48
119.77
119.62
118.71
114.28
114.10
110.70
104.85
104.62
104.12
44.52
44.40
40.17
40.06
33.56
24.62
23.83
22.75
CF3
N
O
NH
N
H
18
(1H, 150 MHz, DMSO-d6)
f1 (ppm)

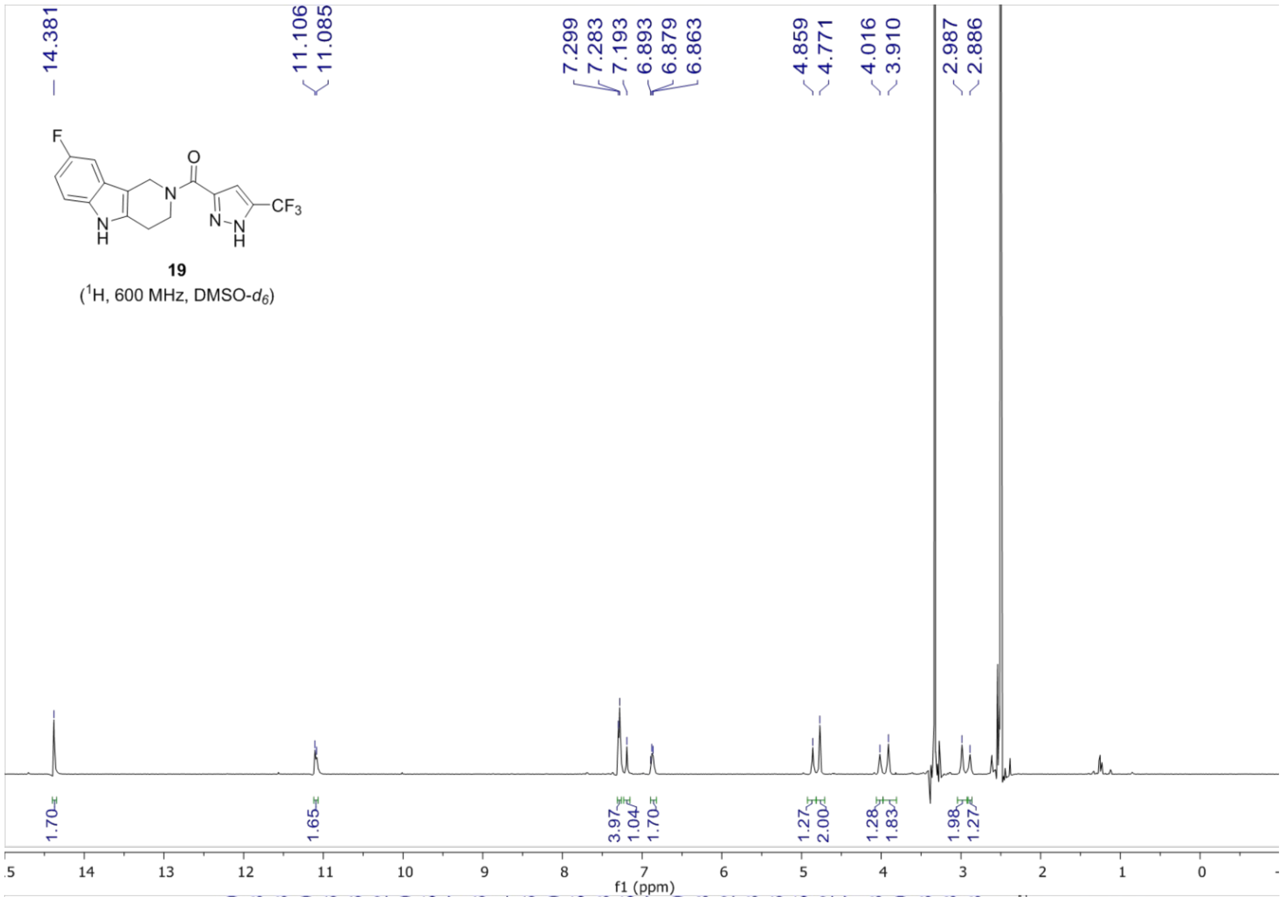
14.381
11.106
11.085
7.299
7.283
7.193
6.893
6.879
6.863
4.859
4.771
4.016
3.910
2.987
2.886
F
O
N
CF3
N
N
H
H
19
(1H, 600 MHz, DMSO-d6)
1.70
1.65
3.97
1.04
1.70
1.27
2.00
1.28
1.83
1.98
1.27
f1 (ppm)

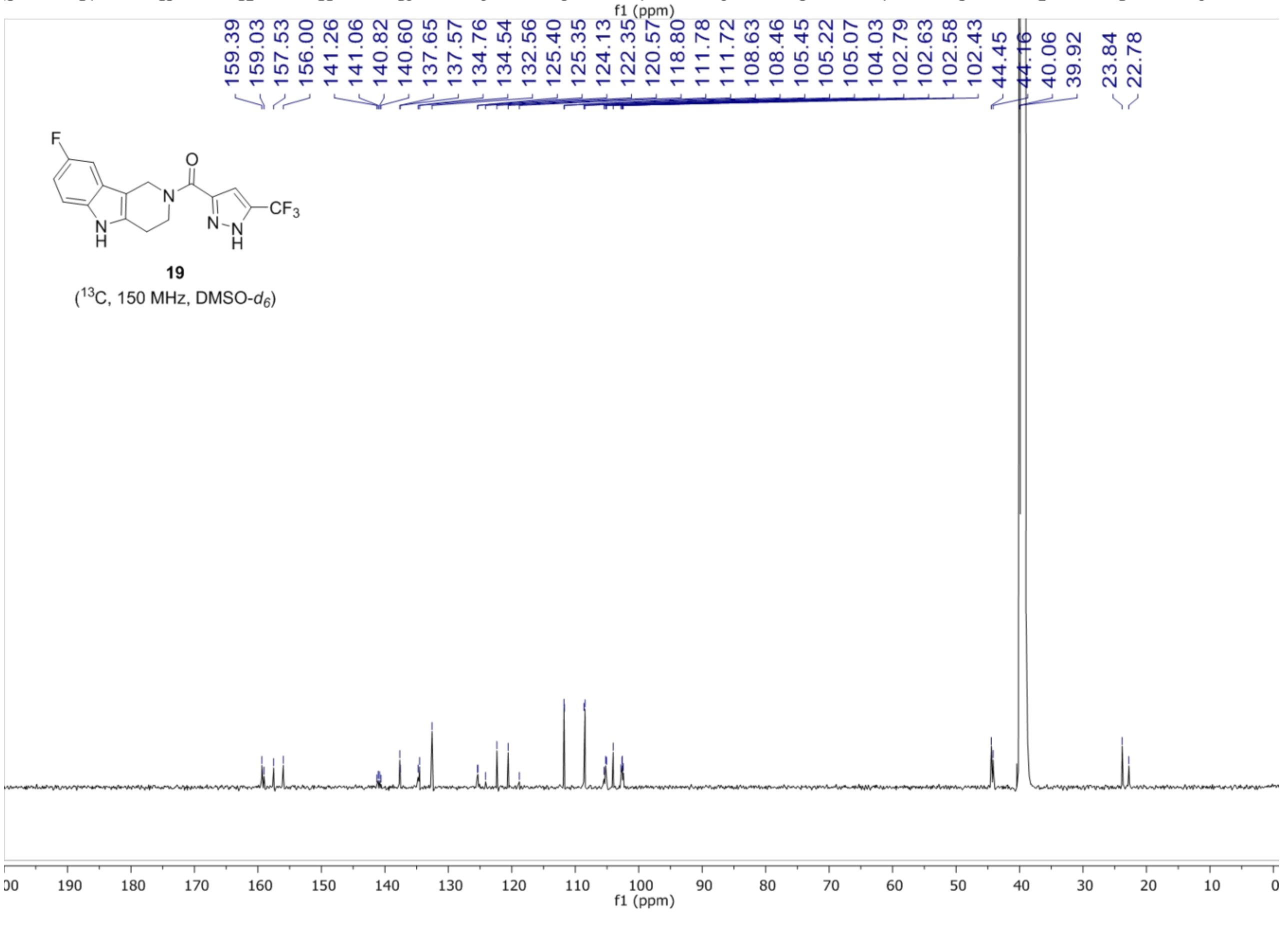
159.39
159.03
157.53
156.00
141.26
141.06
140.82
140.60
137.65
137.57
134.76
134.54
132.56
125.40
125.35
124.13
122.35
120.57
118.80
111.78
111.72
108.63
108.46
105.45
105.22
105.07
104.03
102.79
102.63
102.58
102.43
44.45
44.16
40.06
39.92
23.84
22.78
F
O
N
CF3
N
N
H
H
19
(13C, 150 MHz, DMSO-d6)
f1 (ppm)

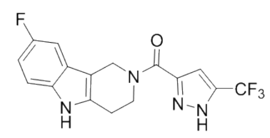

19

(^{19}F, 565 MHz, DMSO-d_6)

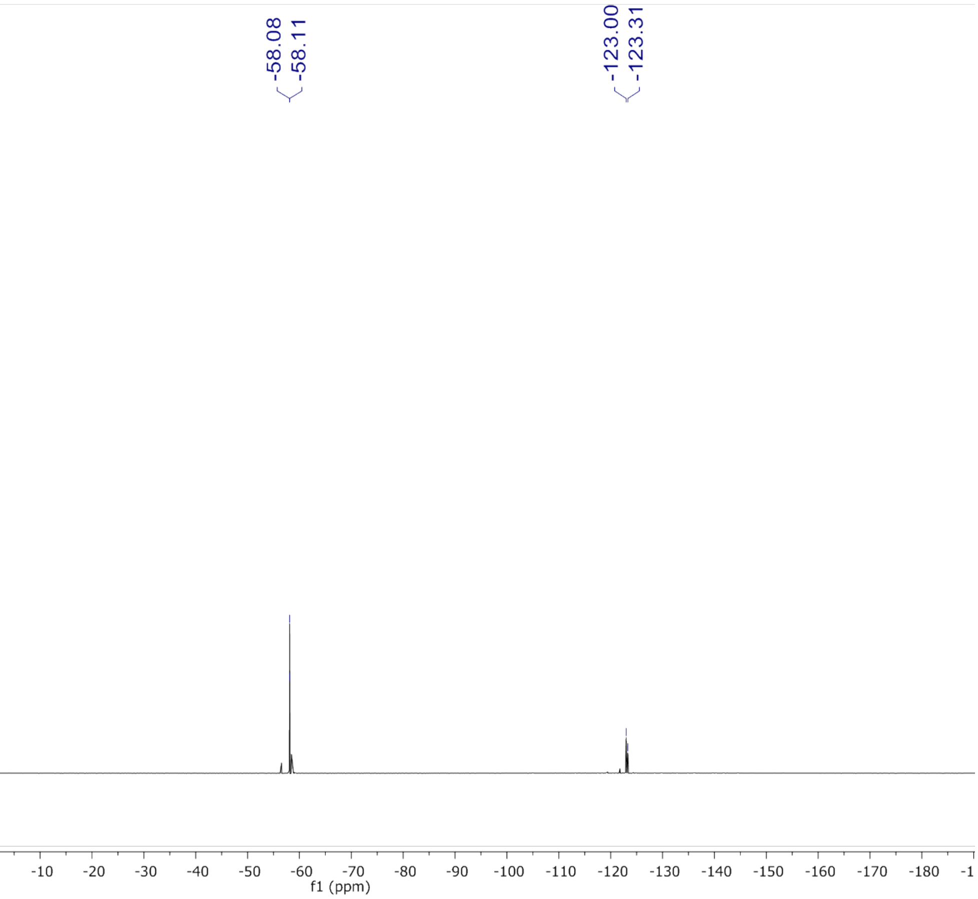

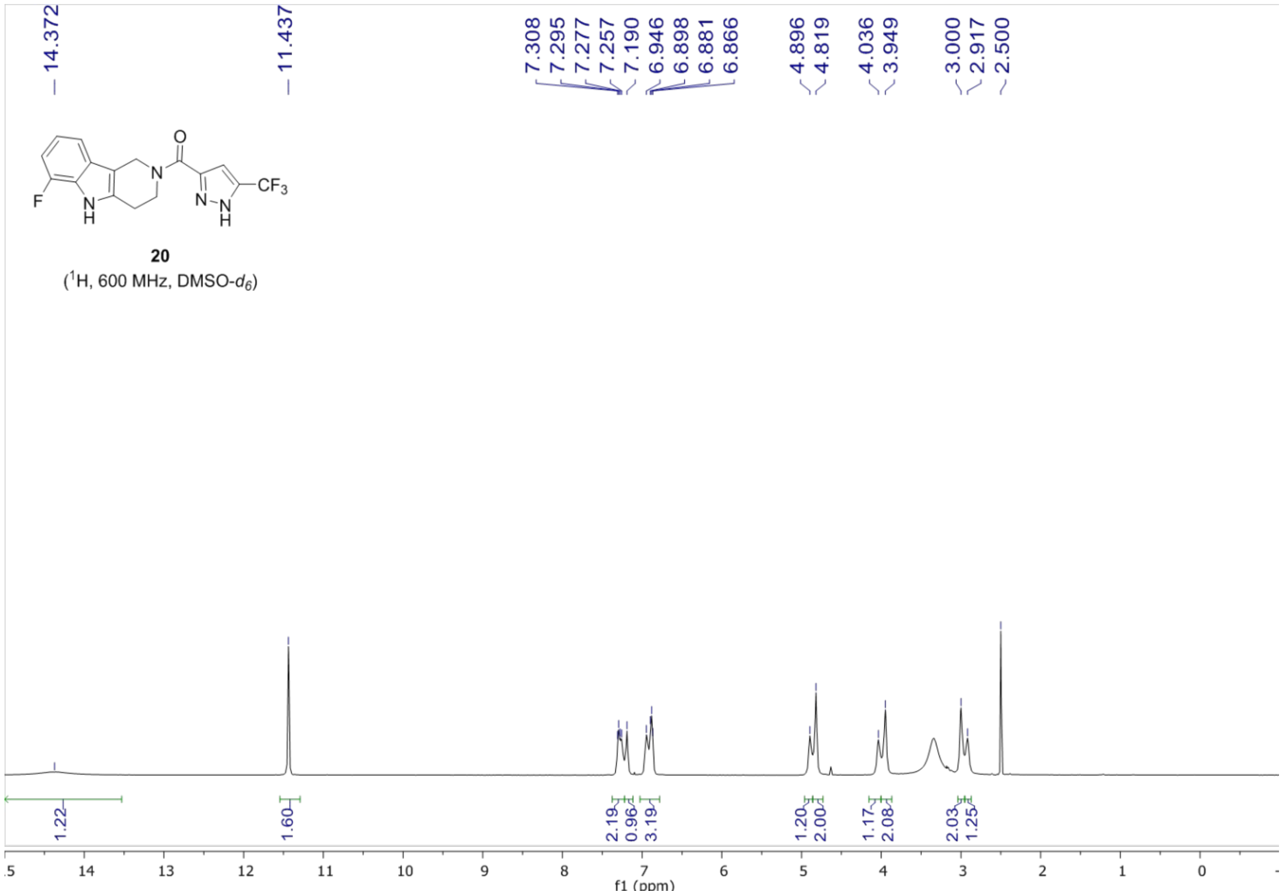
14.372
11.437
7.308
7.295
7.277
7.257
7.190
6.946
6.898
6.881
6.866
4.896
4.819
4.036
3.949
3.000
2.917
2.500
F
N
H
N
O
N
N
H
CF3
20
(1H, 600 MHz, DMSO-d6)
1.22
1.60
2.19
0.96
3.19
1.20
2.00
1.17
2.08
2.03
1.25
f1 (ppm)

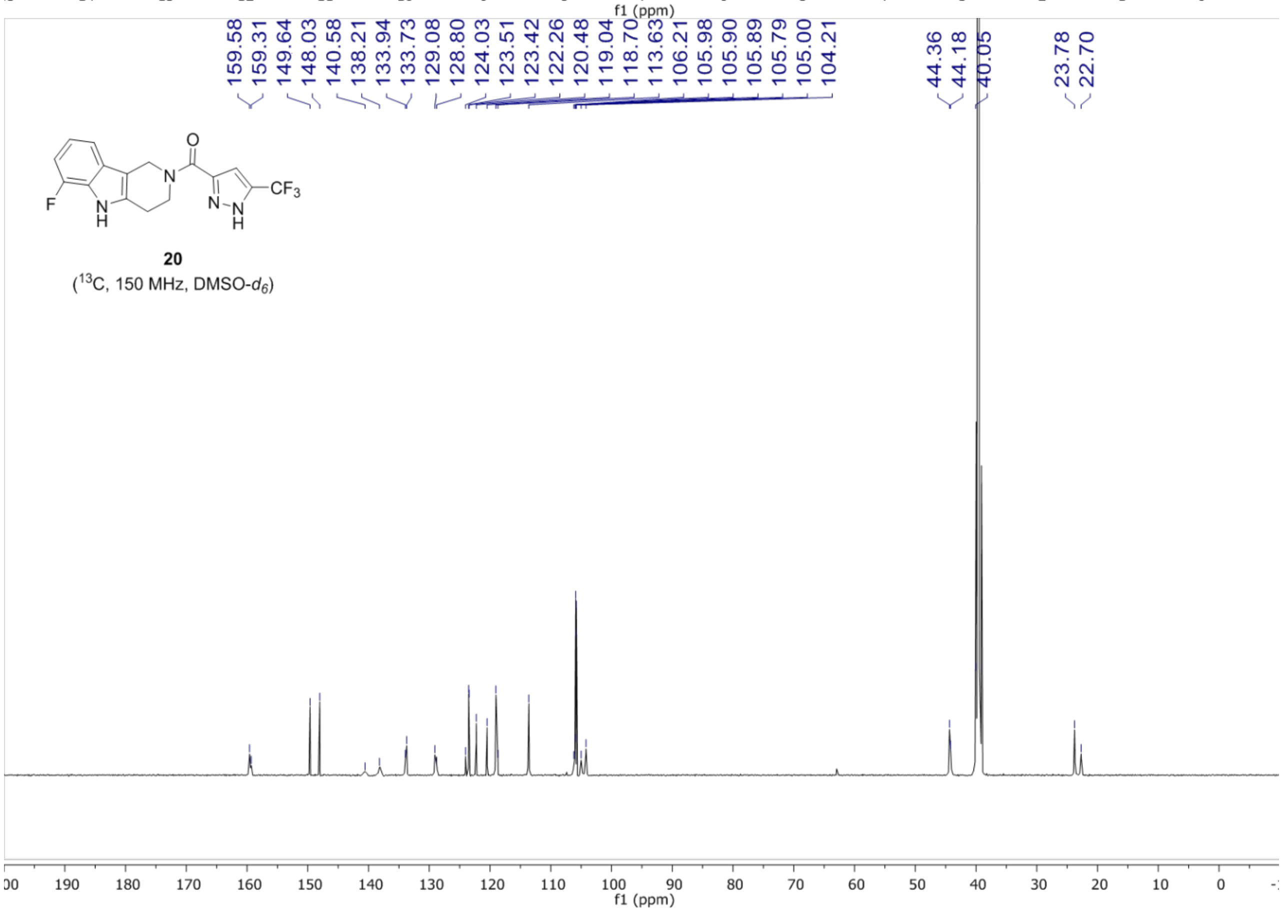
159.58
159.31
149.64
148.03
140.58
138.21
133.94
133.73
129.08
128.80
124.03
123.51
123.42
122.26
120.48
119.04
118.70
113.63
106.21
105.98
105.90
105.89
105.79
105.00
104.21
44.36
44.18
40.05
23.78
22.70
20
(13C, 150 MHz, DMSO-d6)
f1 (ppm)

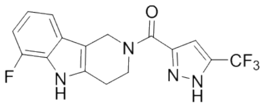

20
(^{19}F, 565 MHz, DMSO-d_6)

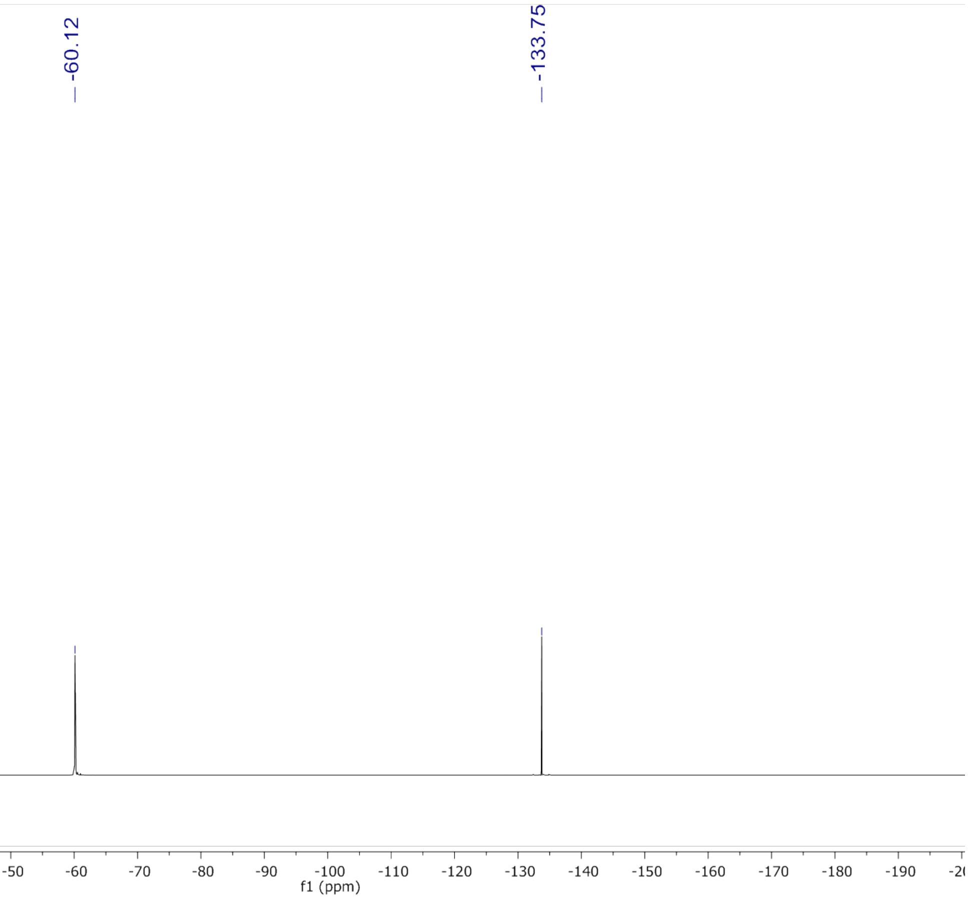

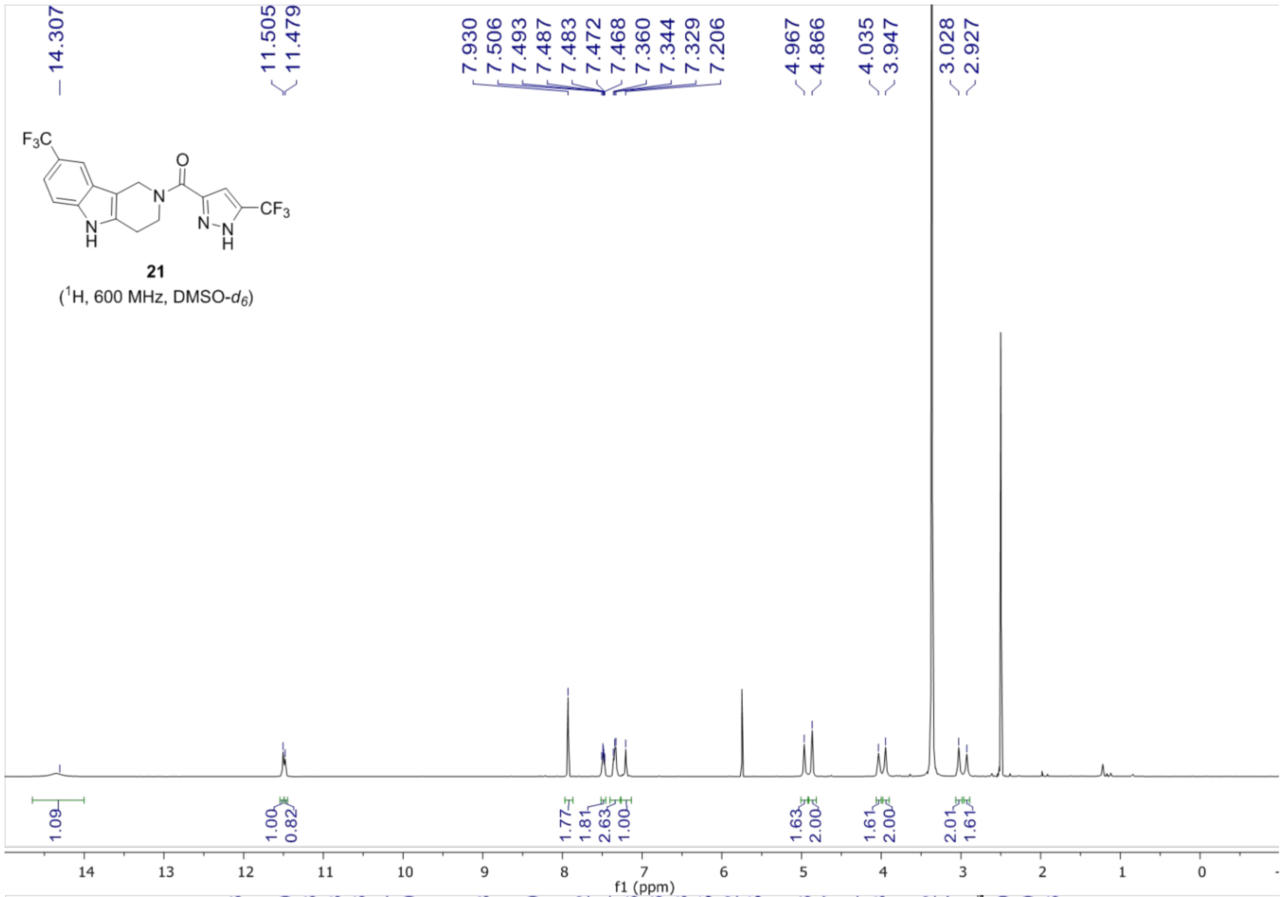
14.307
11.505
11.479
7.930
7.506
7.493
7.487
7.483
7.472
7.468
7.360
7.344
7.329
7.206
4.967
4.866
4.035
3.947
3.028
2.927
21
(^{1}H, 600 MHz, DMSO-d_6)
1.09
1.00
0.82
1.77
1.81
2.63
1.00
1.63
2.00
1.61
2.00
2.01
1.61
f1 (ppm)

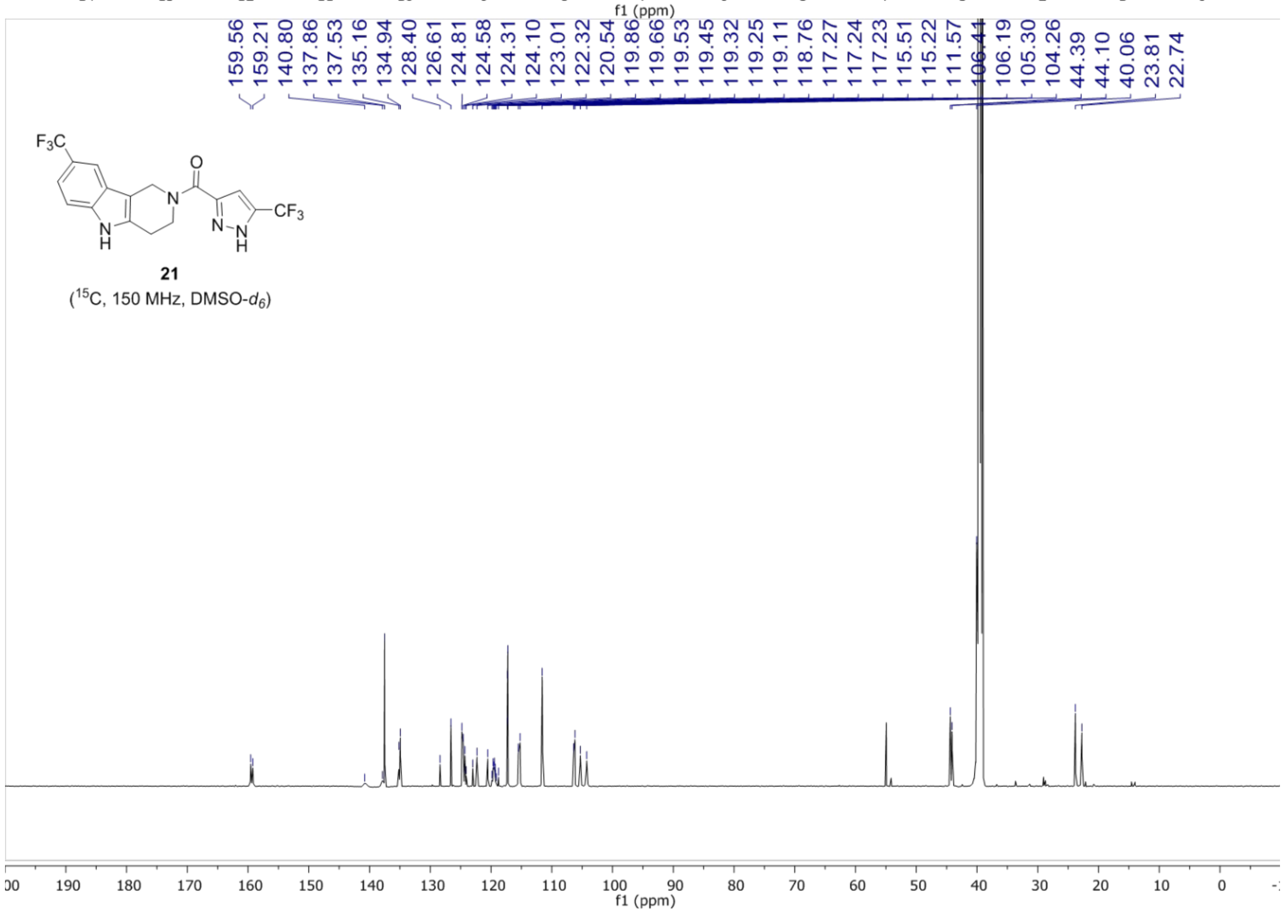
159.56
159.21
140.80
137.86
137.53
135.16
134.94
128.40
126.61
124.81
124.58
124.31
124.10
123.01
122.32
120.54
119.86
119.66
119.53
119.45
119.32
119.25
119.11
118.76
117.27
117.24
117.23
115.51
115.22
111.57
106.44
106.19
105.30
104.26
44.39
44.10
40.06
23.81
22.74
21
(^{15}C, 150 MHz, DMSO-d_6)
f1 (ppm)

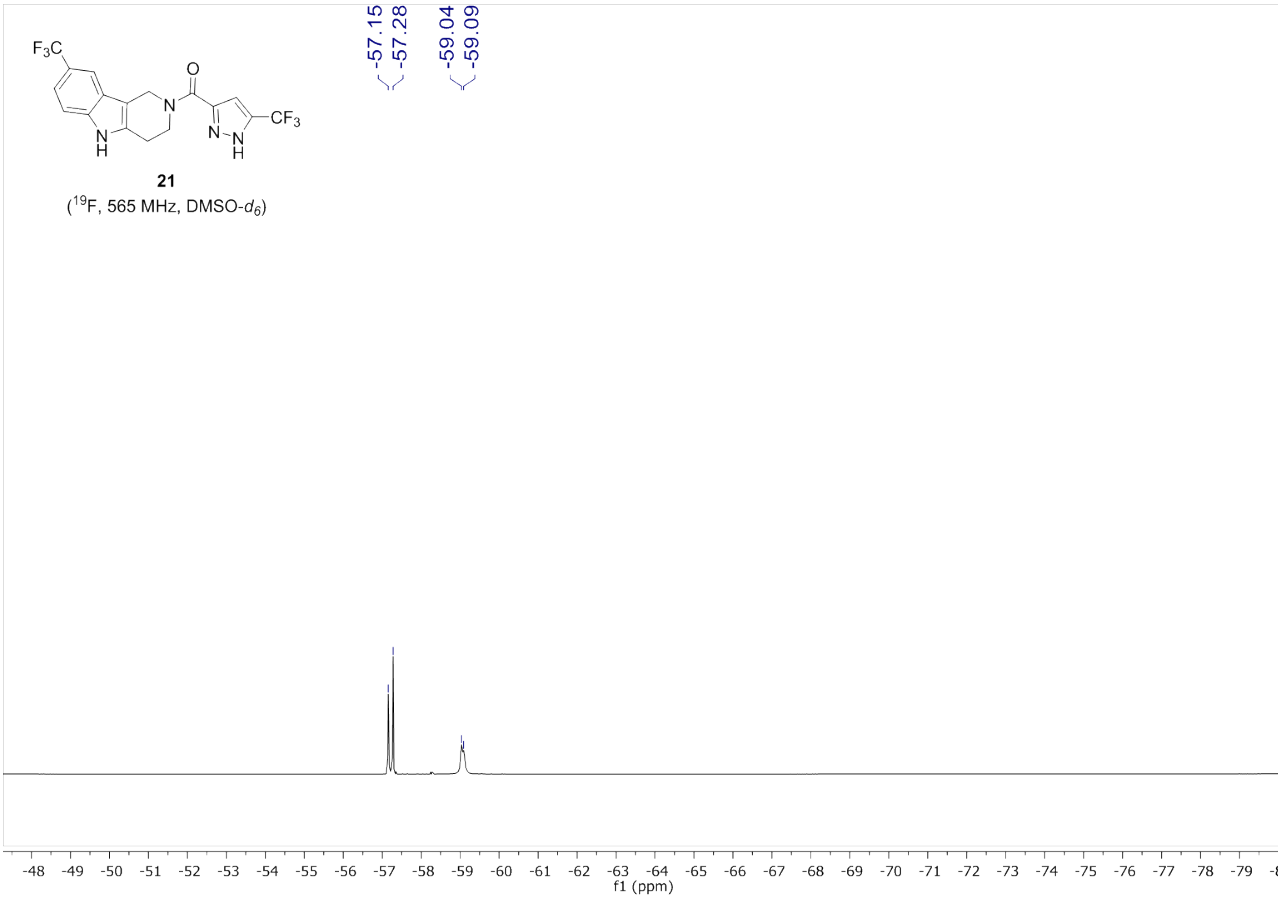

21

(^{19}F, 565 MHz, DMSO-d_6)

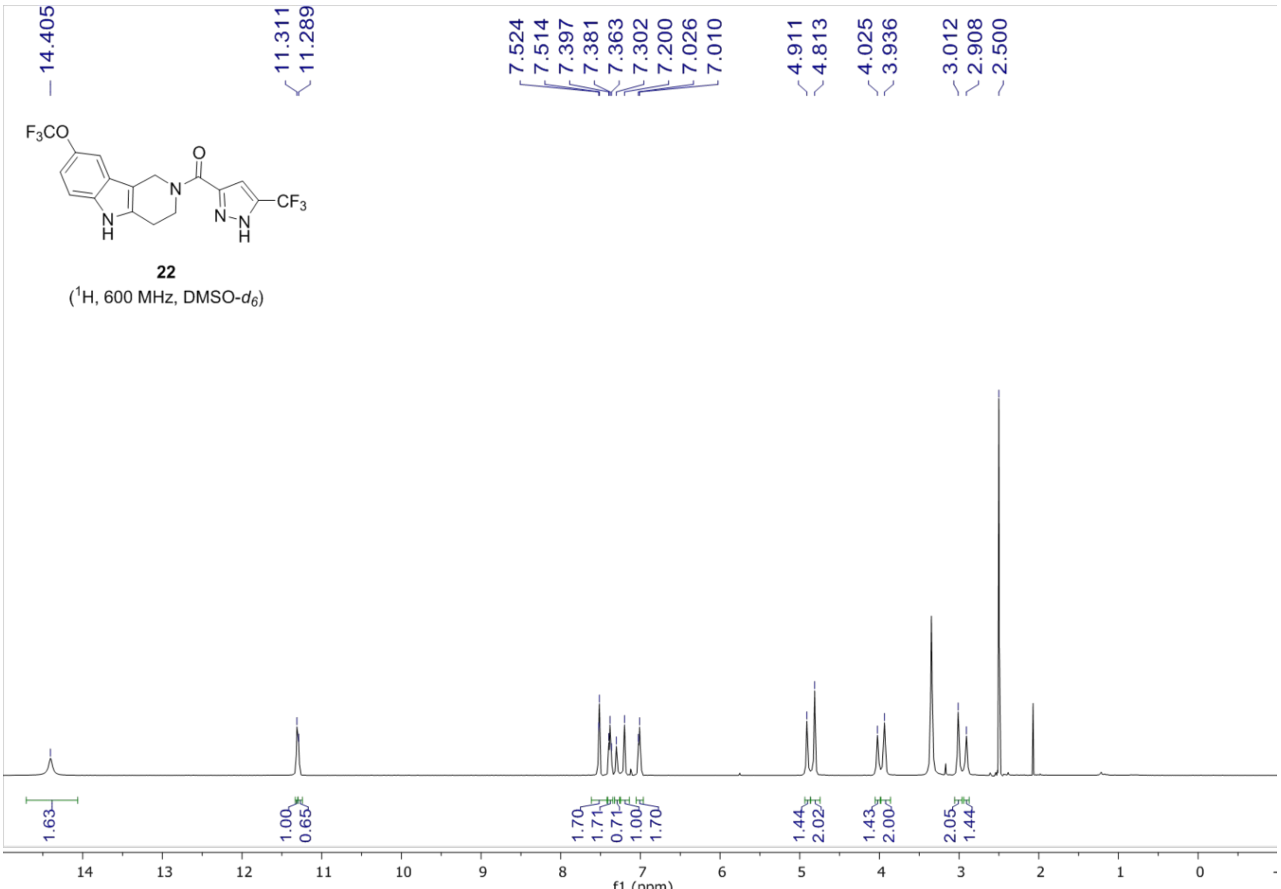
14.405
11.311
11.289
7.524
7.514
7.397
7.381
7.363
7.302
7.200
7.026
7.010
4.911
4.813
4.025
3.936
3.012
2.908
2.500
F3CO
O
N
CF3
N
H
N
N
H
22
(1H, 600 MHz, DMSO-d6)
1.63
1.00
0.65
1.70
1.71
0.71
1.00
1.70
1.44
2.02
1.43
2.00
2.05
1.44
14 13 12 11 10 9 8 7 6 5 4 3 2 1 0
f1 (ppm)

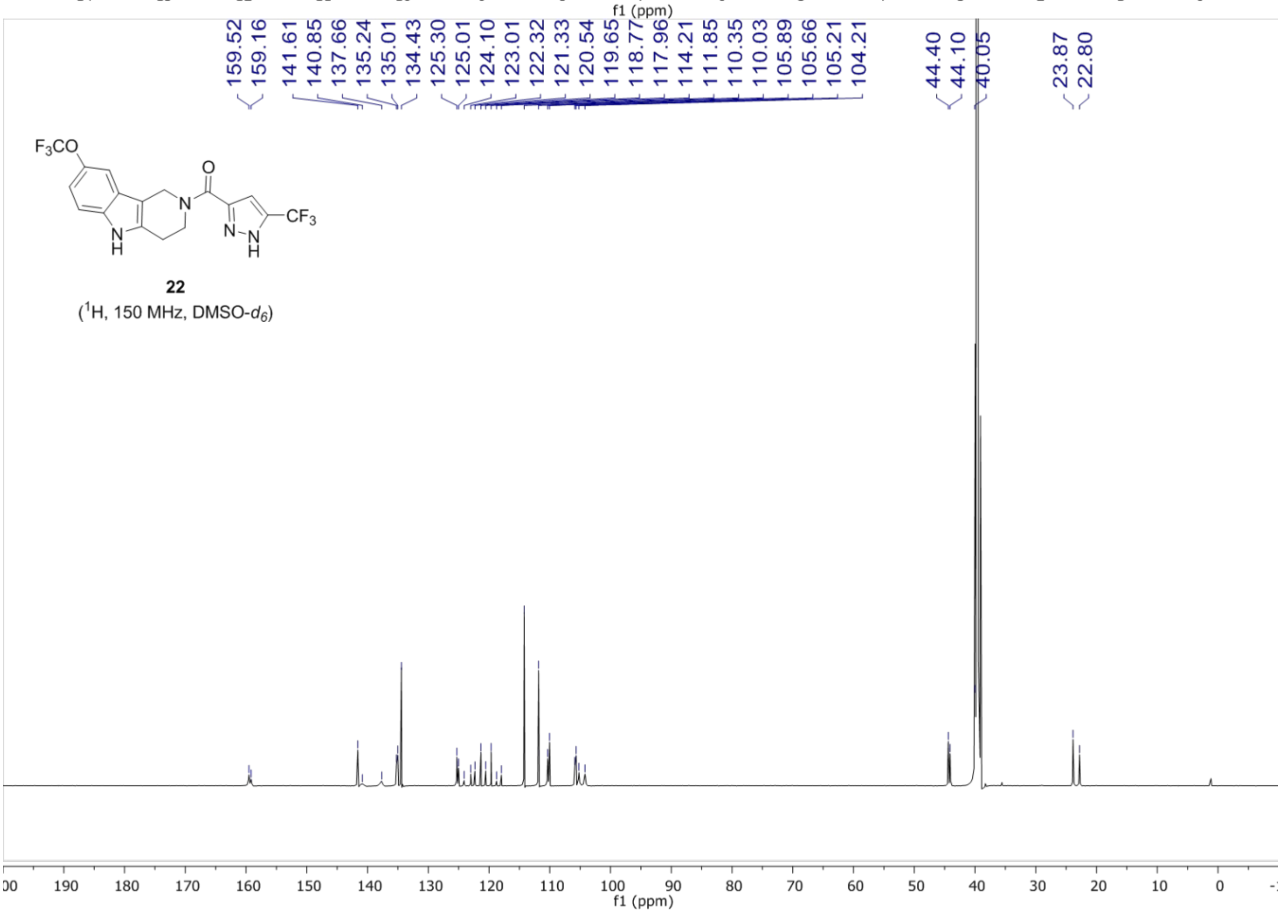
159.52
159.16
141.61
140.85
137.66
135.24
135.01
134.43
125.30
125.01
124.10
123.01
122.32
121.33
120.54
119.65
118.77
117.96
114.21
111.85
110.35
110.03
105.89
105.66
105.21
104.21
44.40
44.10
40.05
23.87
22.80
F3CO
O
N
CF3
N
H
N
N
H
22
(1H, 150 MHz, DMSO-d6)
190 180 170 160 150 140 130 120 110 100 90 80 70 60 50 40 30 20 10 0
f1 (ppm)

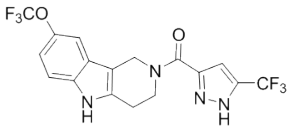

22
(^{19}F, 565 MHz, DMSO-d_6)

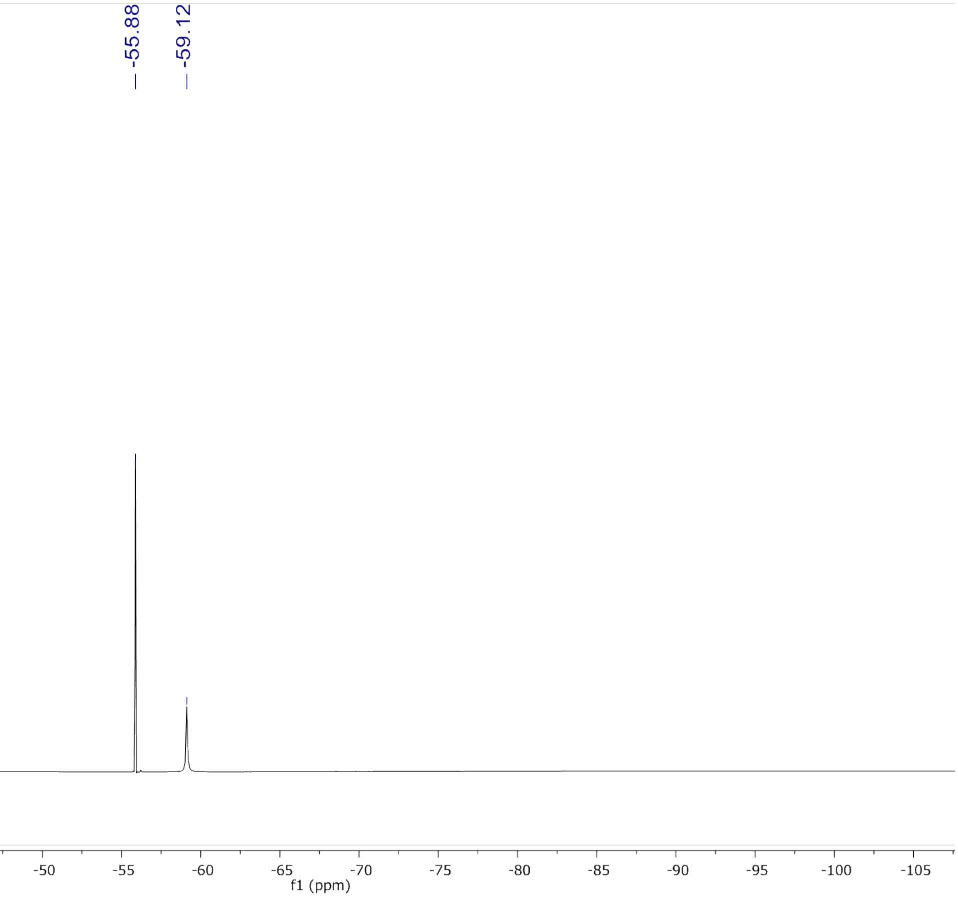

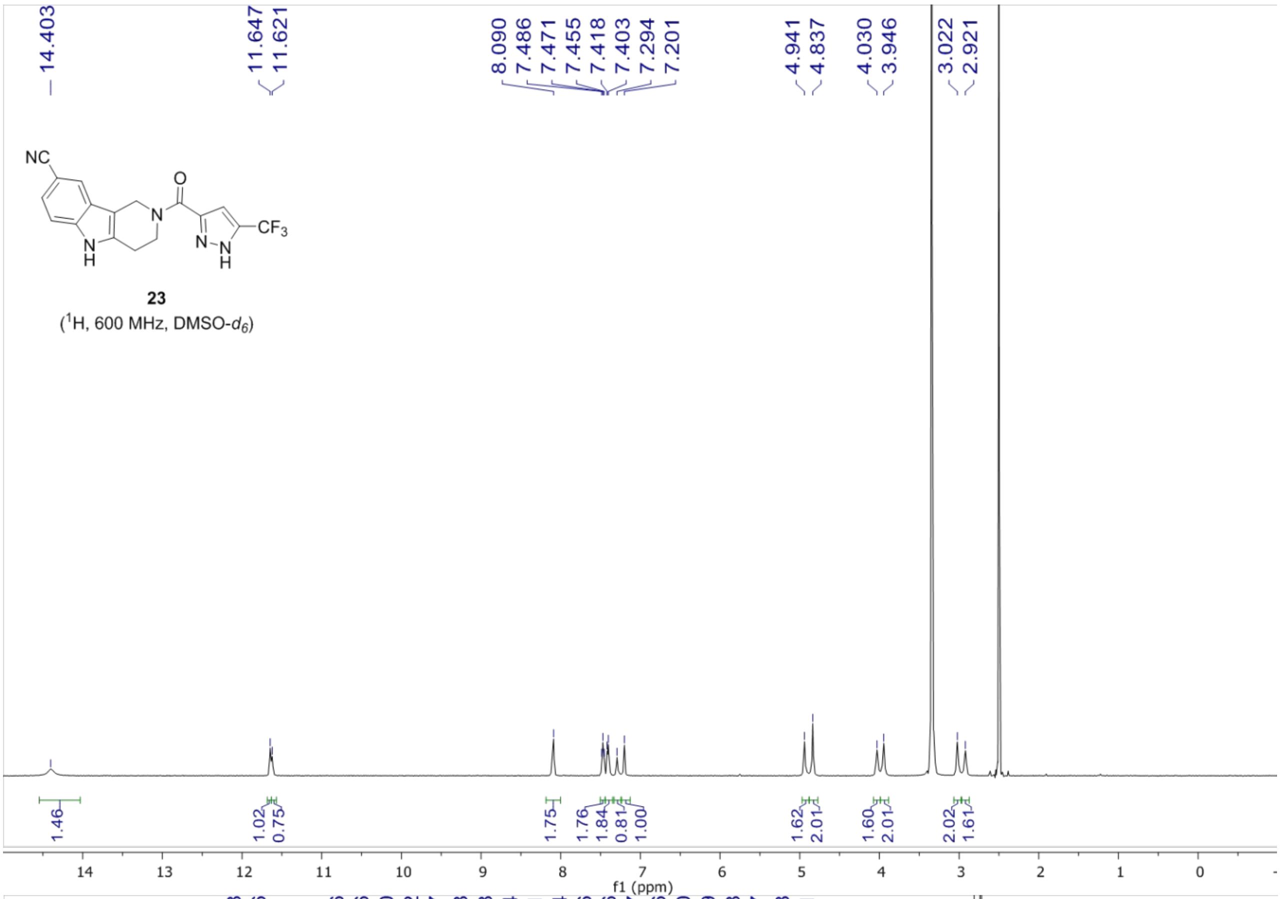
14.403
11.647
11.621
8.090
7.486
7.471
7.455
7.418
7.403
7.294
7.201
4.941
4.837
4.030
3.946
3.022
2.921
NC
O
N
CF3
N
N
H
H
23
(1H, 600 MHz, DMSO-d6)
1.46
1.02
0.75
1.75
1.76
1.84
0.81
1.00
1.62
2.01
1.60
2.01
2.02
1.61
f1 (ppm)

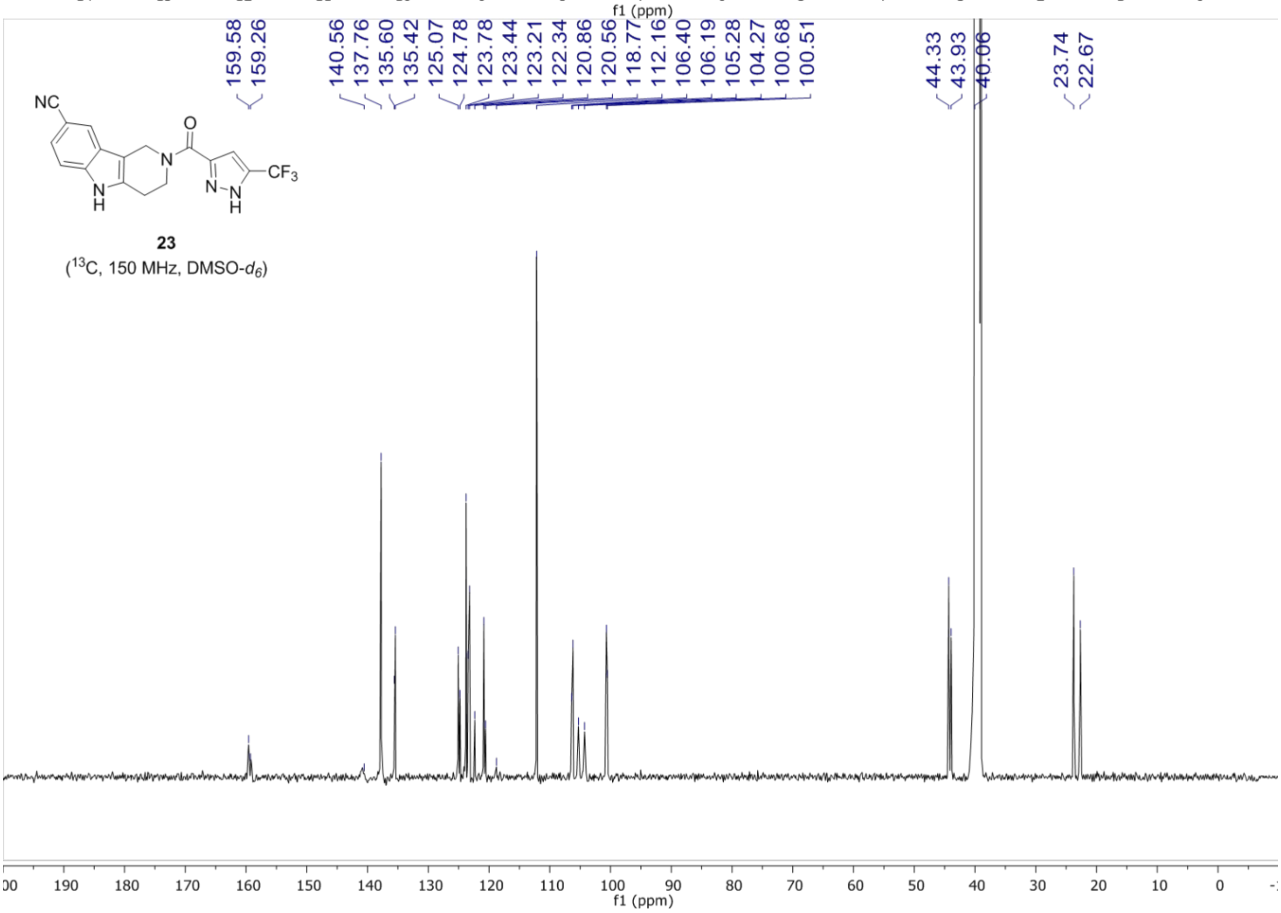
159.58
159.26
140.56
137.76
135.60
135.42
125.07
124.78
123.78
123.44
123.21
122.34
120.86
120.56
118.77
112.16
106.40
106.19
105.28
104.27
100.68
100.51
44.33
43.93
40.06
23.74
22.67
NC
O
N
CF3
N
N
H
H
23
(13C, 150 MHz, DMSO-d6)
f1 (ppm)

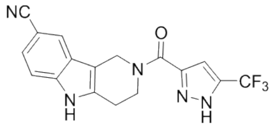

23
(^{19}F, 565 MHz, DMSO-d_6)

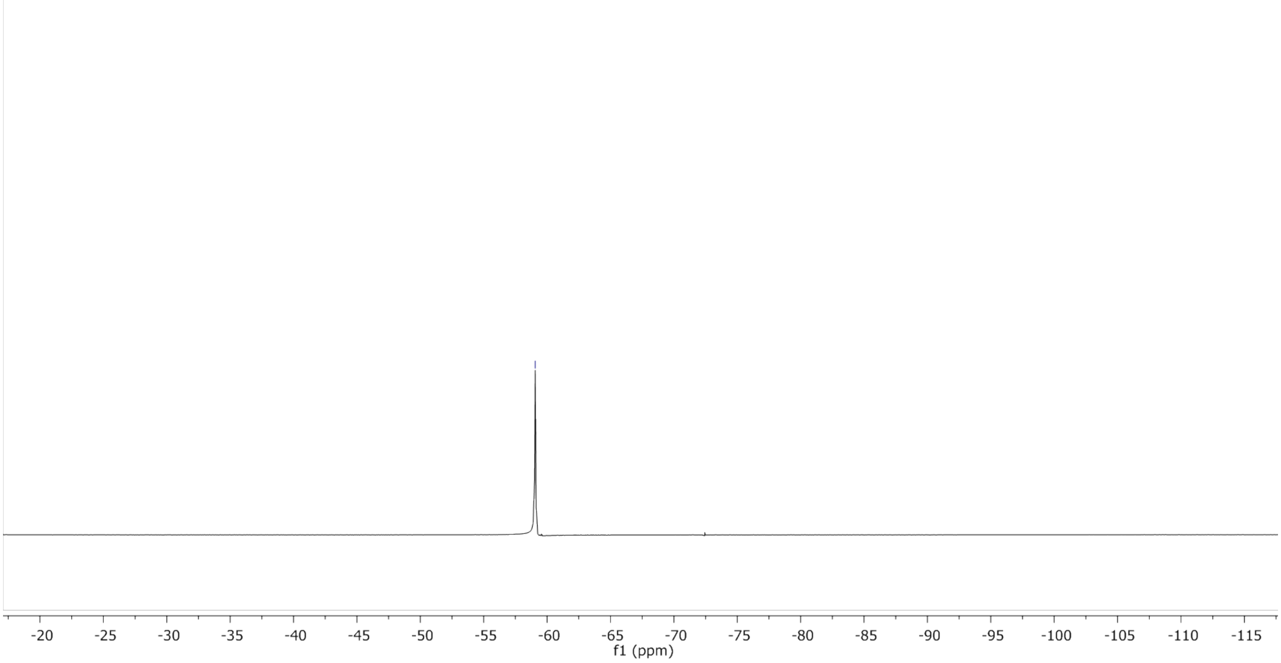

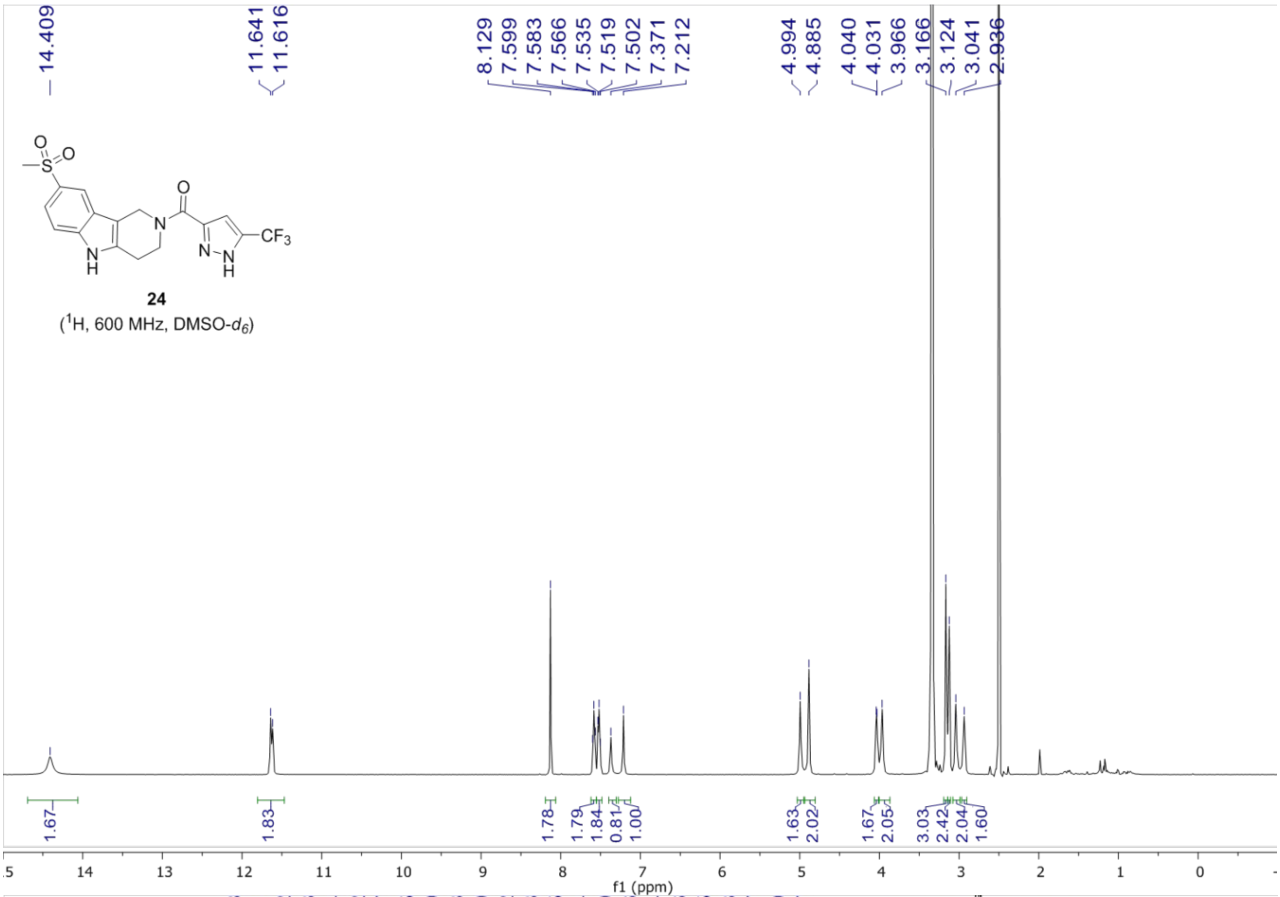
14.409
11.641
11.616
8.129
7.599
7.583
7.566
7.535
7.519
7.502
7.371
7.212
4.994
4.885
4.040
4.031
3.966
3.166
3.124
3.041
2.936
CF3
24
(1H, 600 MHz, DMSO-d6)
1.67
1.83
1.78
1.79
1.84
0.81
1.00
1.63
2.02
1.67
2.05
3.03
2.42
2.04
1.60
f1 (ppm)

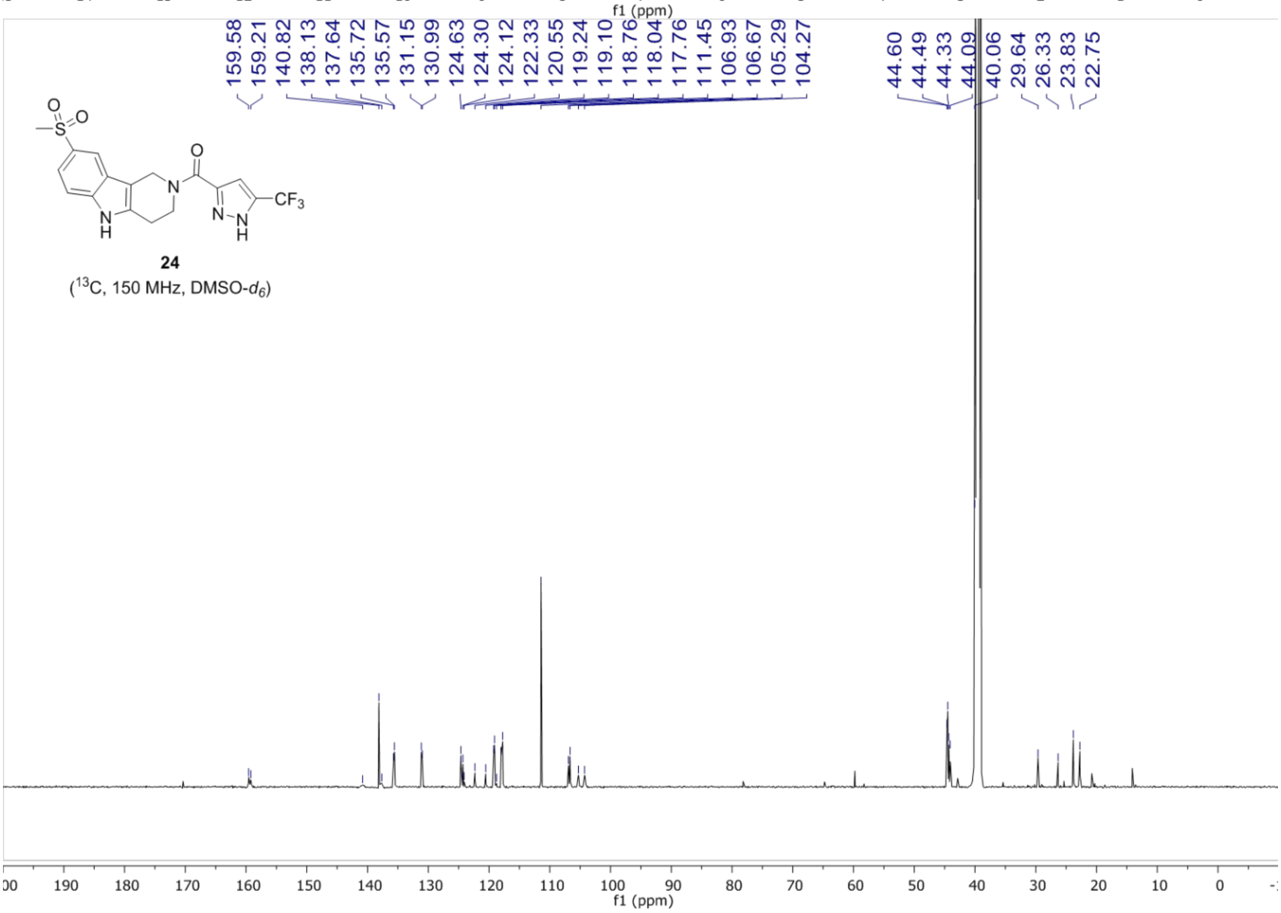
159.58
159.21
140.82
138.13
137.64
135.72
135.57
131.15
130.99
124.63
124.30
124.12
122.33
120.55
119.24
119.10
118.76
118.04
117.76
111.45
106.93
106.67
105.29
104.27
44.60
44.49
44.33
44.09
40.06
29.64
26.33
23.83
22.75
CF3
24
(13C, 150 MHz, DMSO-d6)
f1 (ppm)

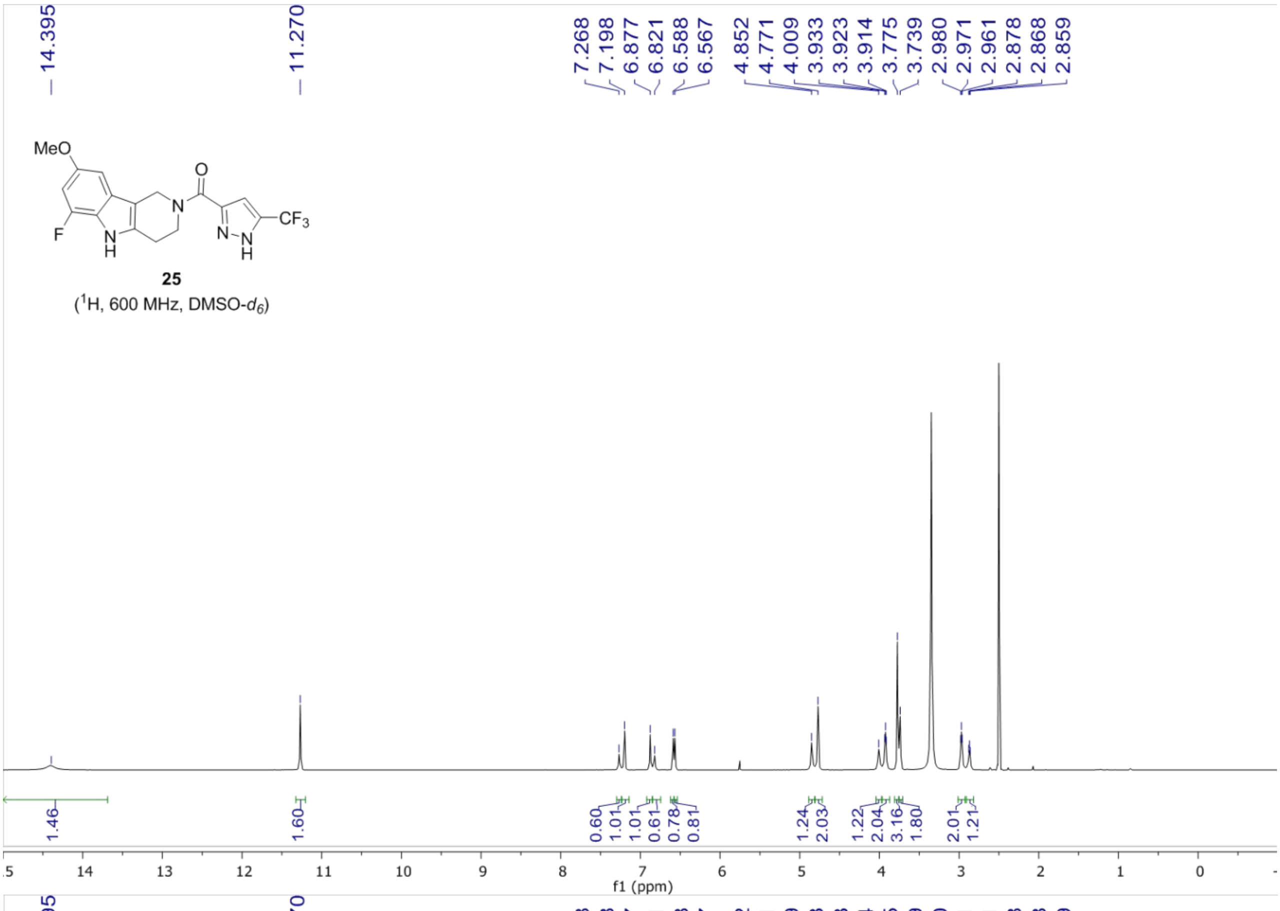
14.395
11.270
7.268
7.198
6.877
6.821
6.588
6.567
4.852
4.771
4.009
3.933
3.923
3.914
3.775
3.739
2.980
2.971
2.961
2.878
2.868
2.859
MeO
O
N
CF3
F
N
H
N
N
H
25
(1H, 600 MHz, DMSO-d6)
1.46
1.60
0.60
1.01
1.01
0.61
0.78
0.81
1.24
2.03
1.22
2.04
3.16
1.80
2.01
1.21
f1 (ppm)

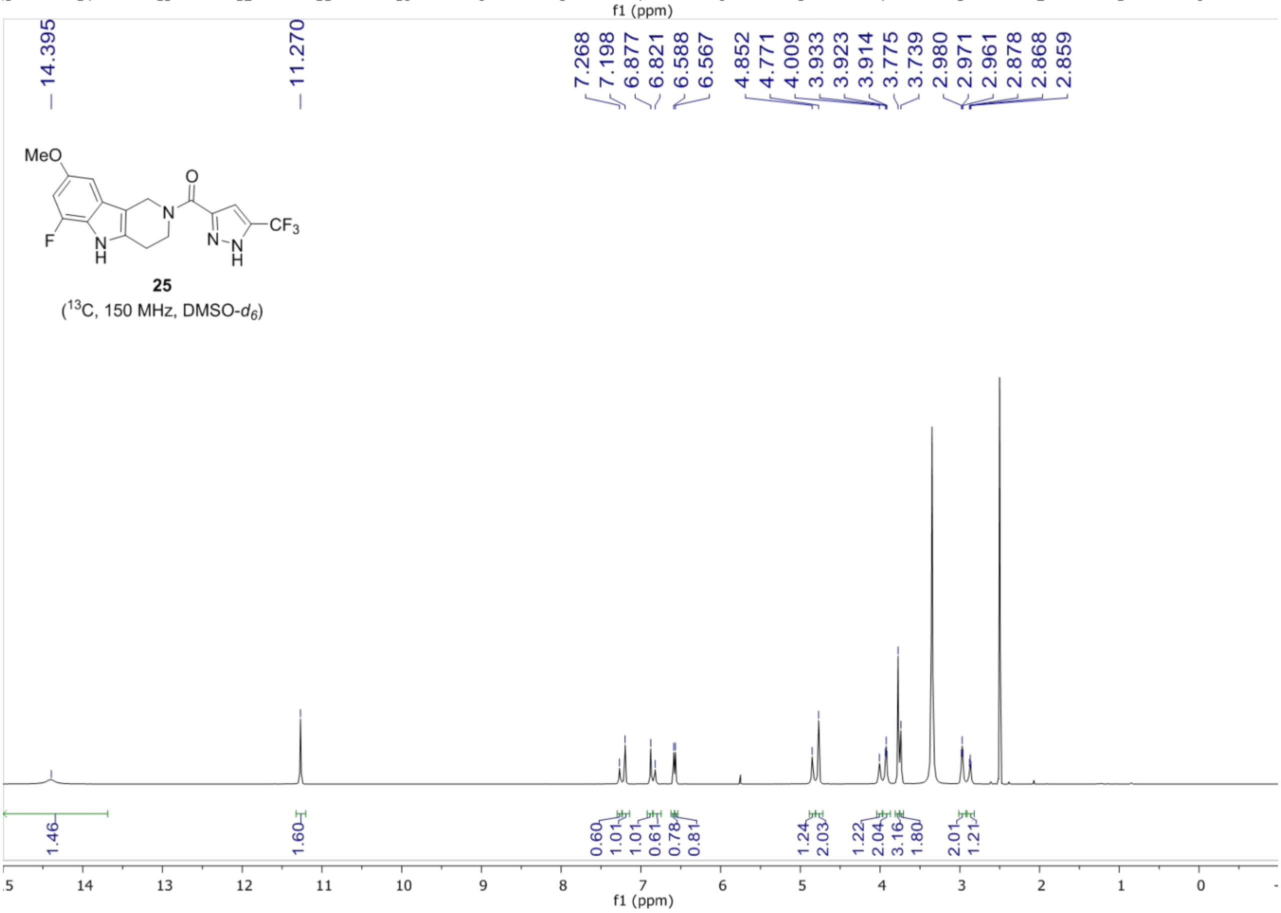
14.395
11.270
7.268
7.198
6.877
6.821
6.588
6.567
4.852
4.771
4.009
3.933
3.923
3.914
3.775
3.739
2.980
2.971
2.961
2.878
2.868
2.859
MeO
O
N
CF3
F
N
H
N
N
H
25
(13C, 150 MHz, DMSO-d6)
1.46
1.60
0.60
1.01
1.01
0.61
0.78
0.81
1.24
2.03
1.22
2.04
3.16
1.80
2.01
1.21
f1 (ppm)

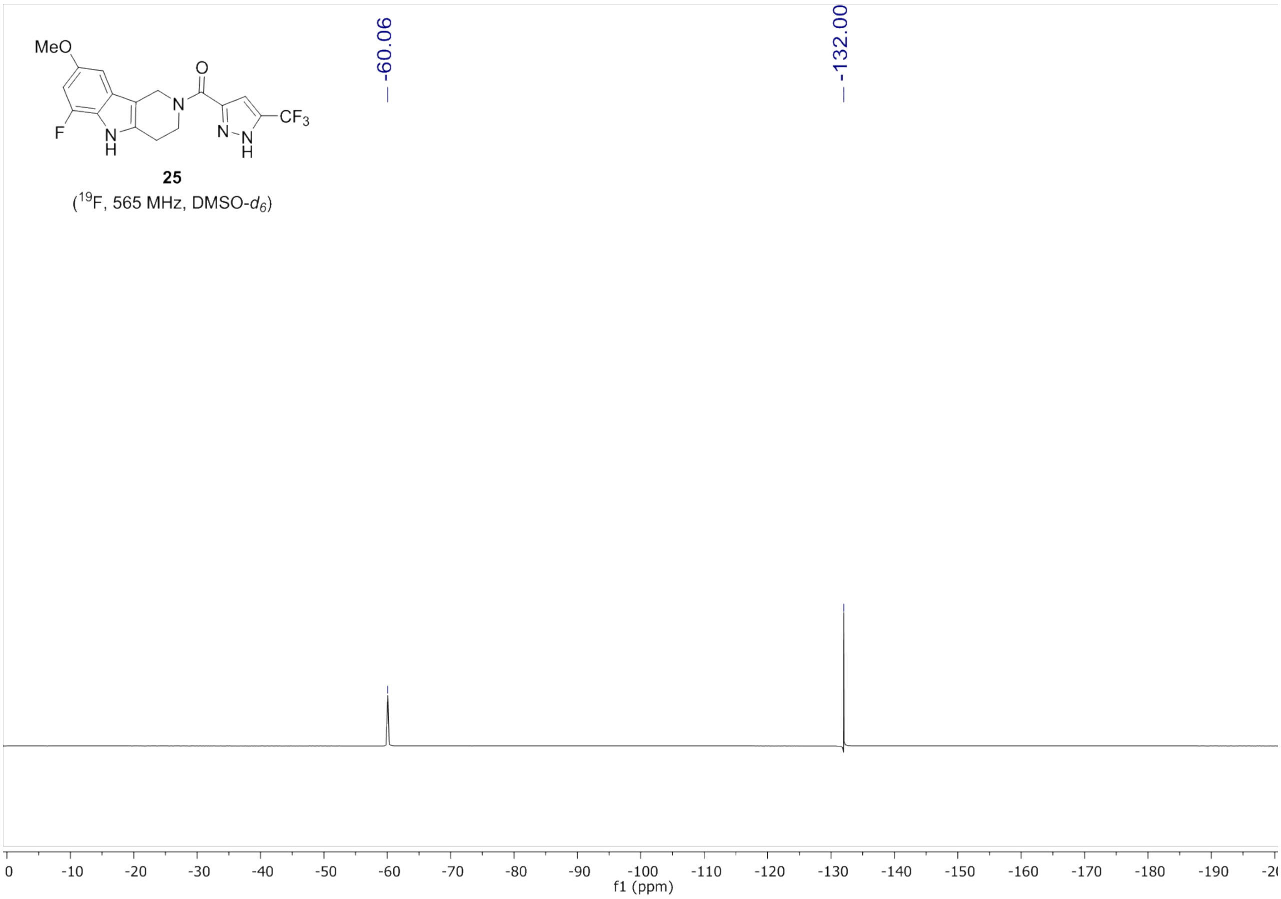
MeO
O
N
F
N
H
CF3
N
N
H
25
(19F, 565 MHz, DMSO-d6)
-60.06
-132.00
0
-10
-20
-30
-40
-50
-60
-70
-80
-90
-100
-110
-120
-130
-140
-150
-160
-170
-180
-190
f1 (ppm)

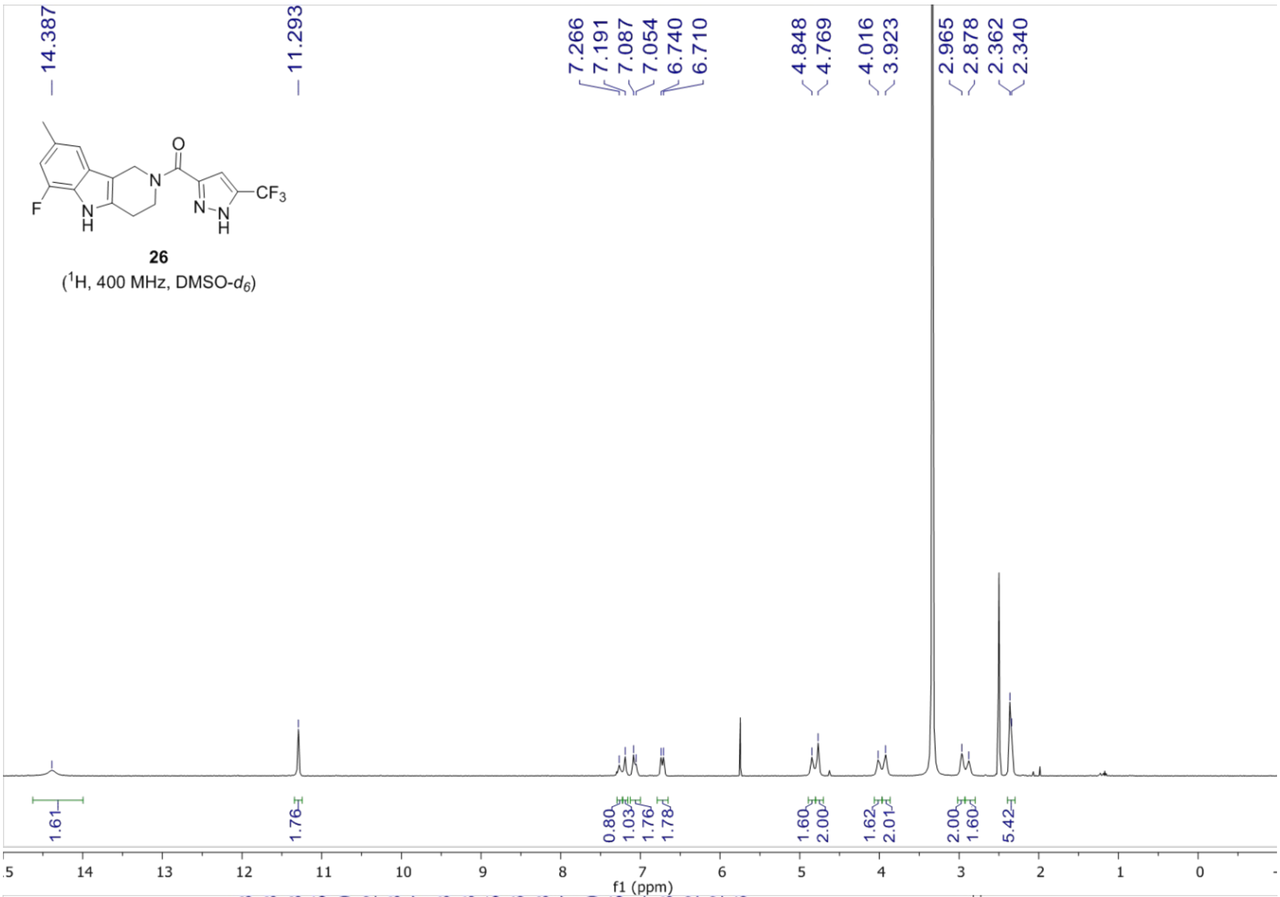
14.387
11.293
7.266
7.191
7.087
7.054
6.740
6.710
4.848
4.769
4.016
3.923
2.965
2.878
2.362
2.340
O
N
CF3
F
NH
N
N
H
26
(1H, 400 MHz, DMSO-d6)
1.61
1.76
0.80
1.03
1.76
1.78
1.60
2.00
1.62
2.01
2.00
1.60
5.42
f1 (ppm)

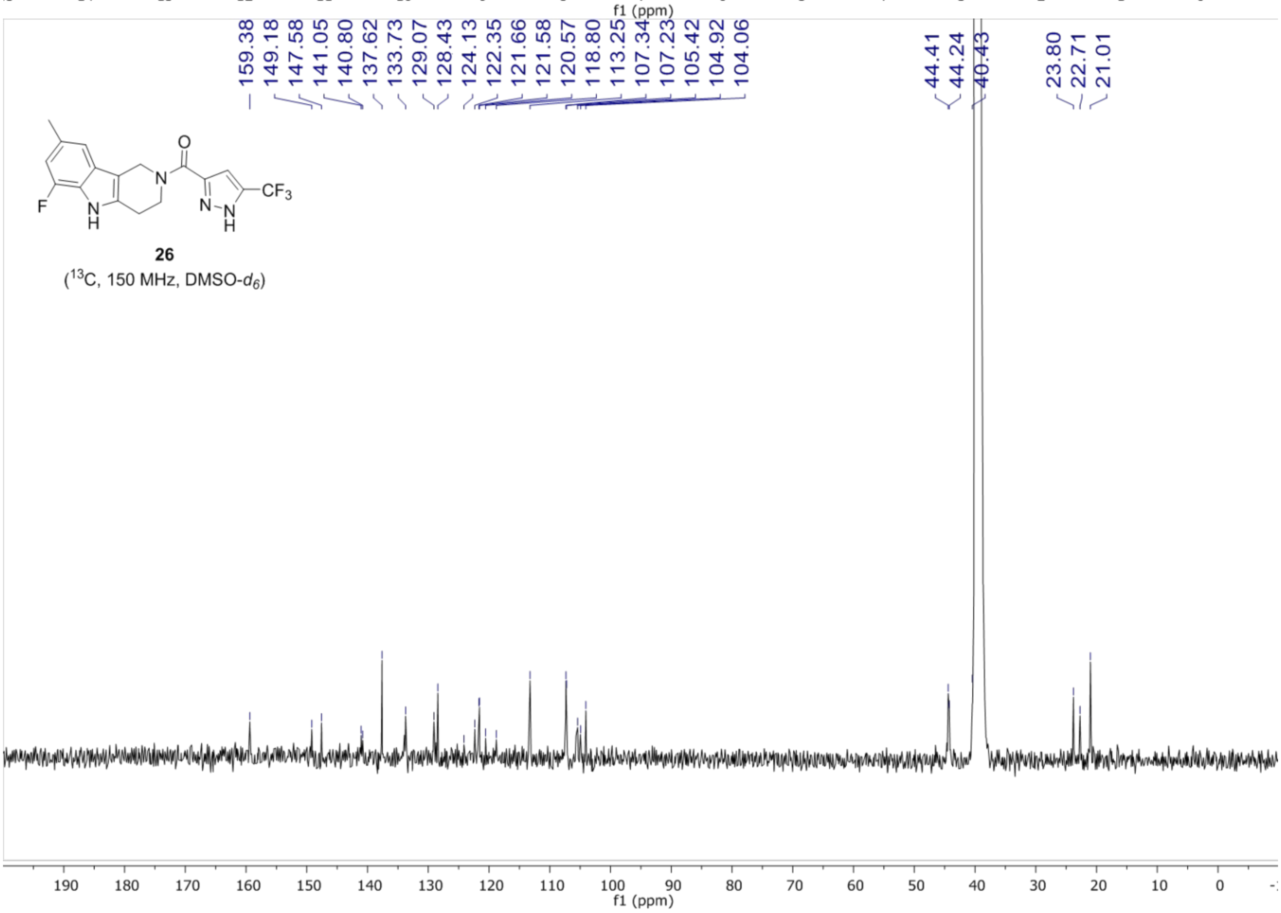
159.38
149.18
147.58
141.05
140.80
137.62
133.73
129.07
128.43
124.13
122.35
121.66
121.58
120.57
118.80
113.25
107.34
107.23
105.42
104.92
104.06
44.41
44.24
40.43
23.80
22.71
21.01
26
(13C, 150 MHz, DMSO-d6)
f1 (ppm)

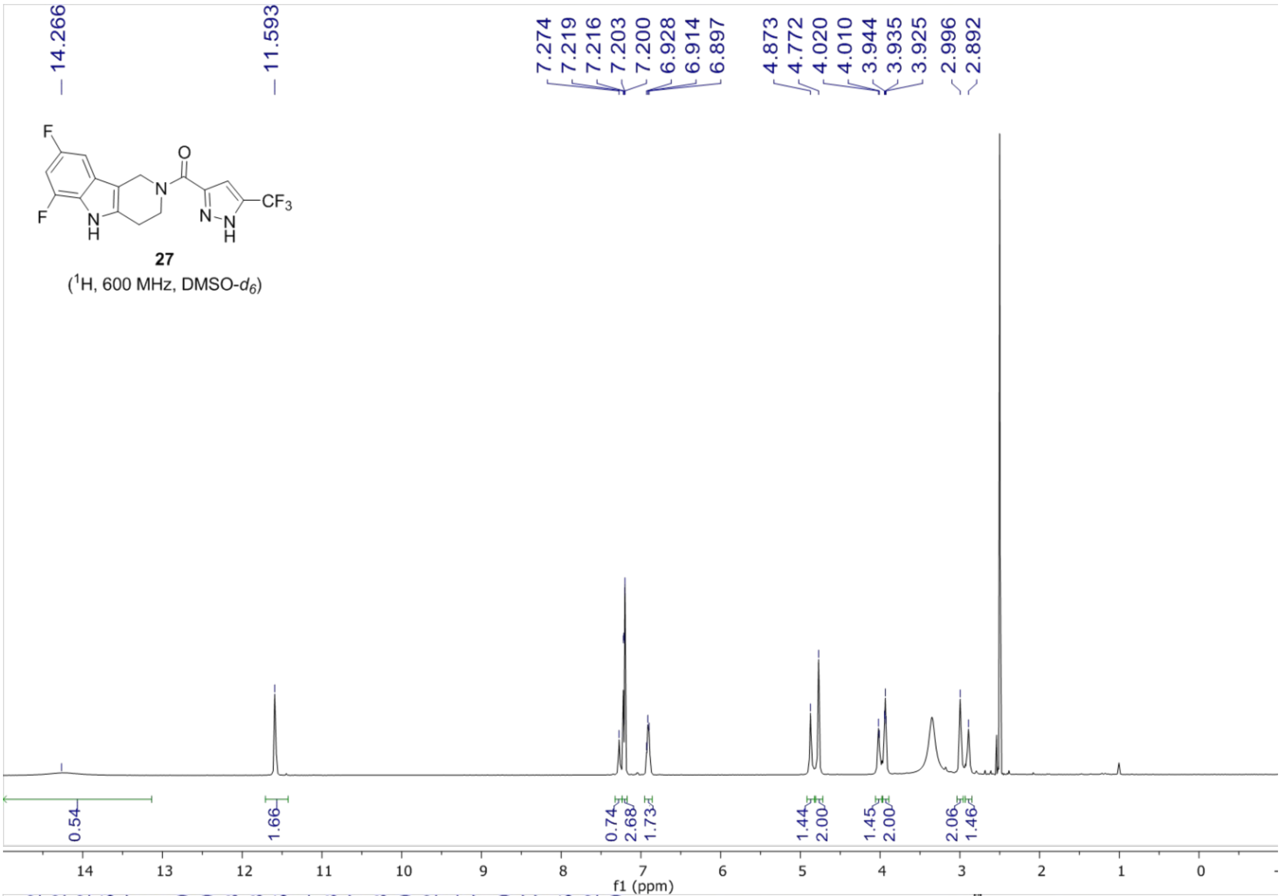
14.266
11.593
7.274 7.219 7.216 7.203 7.200 6.928 6.914 6.897
4.873 4.772 4.020 4.010 3.944 3.935 3.925 2.996 2.892
F
F
O
N
N
H
N
N
H
CF3
27
(1H, 600 MHz, DMSO-d6)
0.54
1.66
0.74 2.68 1.73
1.44 2.00
1.45 2.00
2.06 1.46
f1 (ppm)

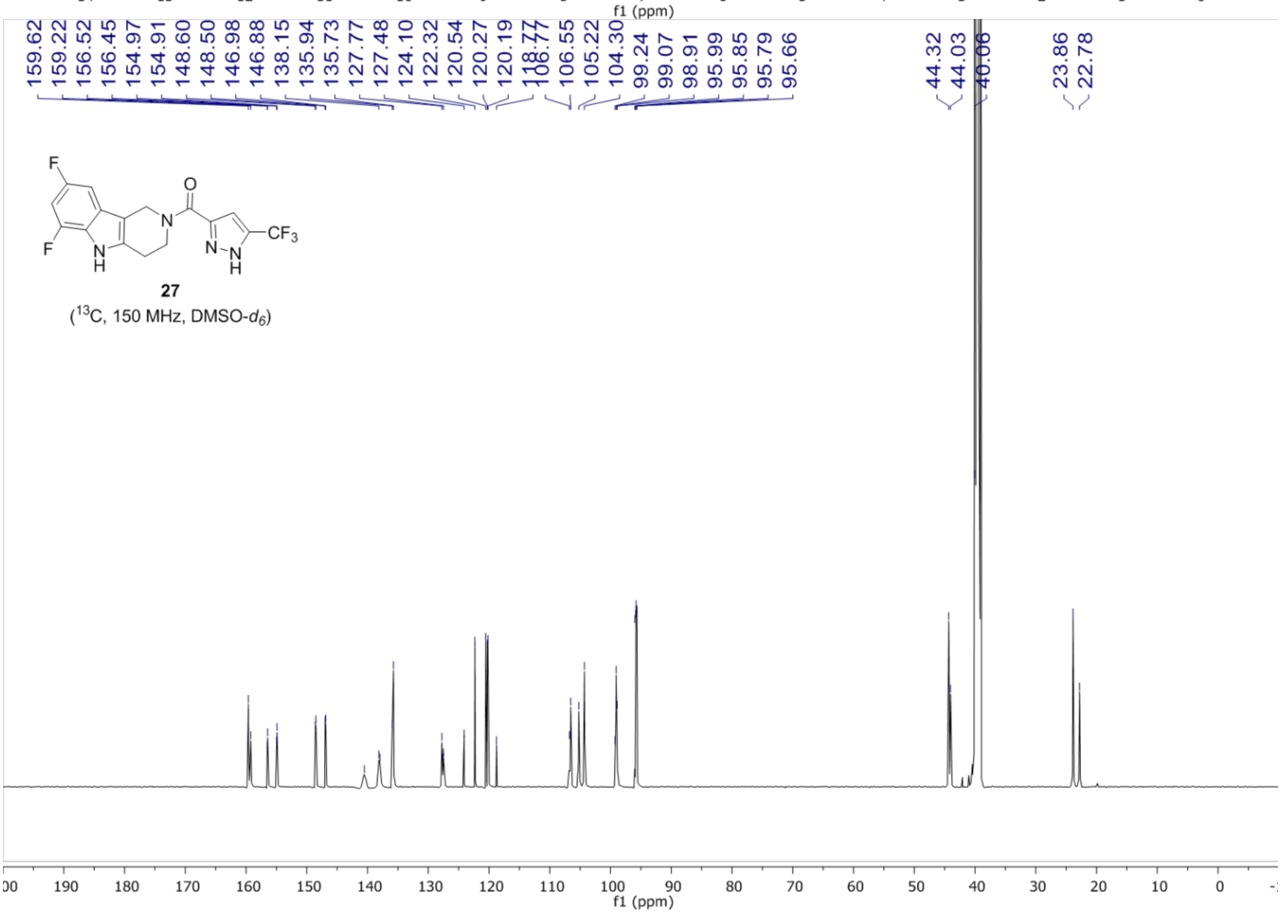
159.62 159.22 156.52 156.45 154.97 154.91 148.60 148.50 146.98 146.88 138.15 135.94 135.73 127.77 127.48 124.10 122.32 120.54 120.27 120.19
106.55 105.22 104.30 99.24 99.07 98.91 95.99 95.85 95.79 95.66
44.32 44.03 40.06
23.86 22.78
F
F
O
N
N
H
N
N
H
CF3
27
(13C, 150 MHz, DMSO-d6)
f1 (ppm)

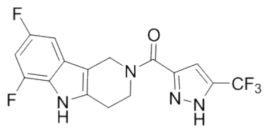

27

(^{19}F, 565 MHz, DMSO-d_6)

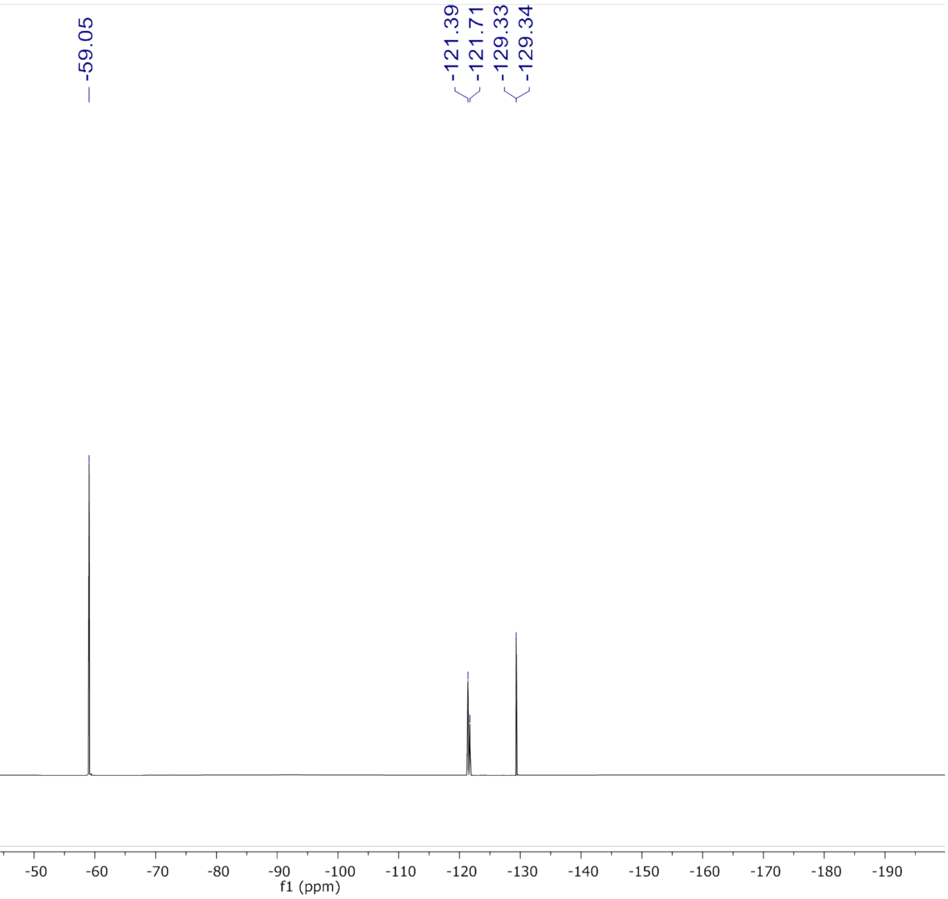

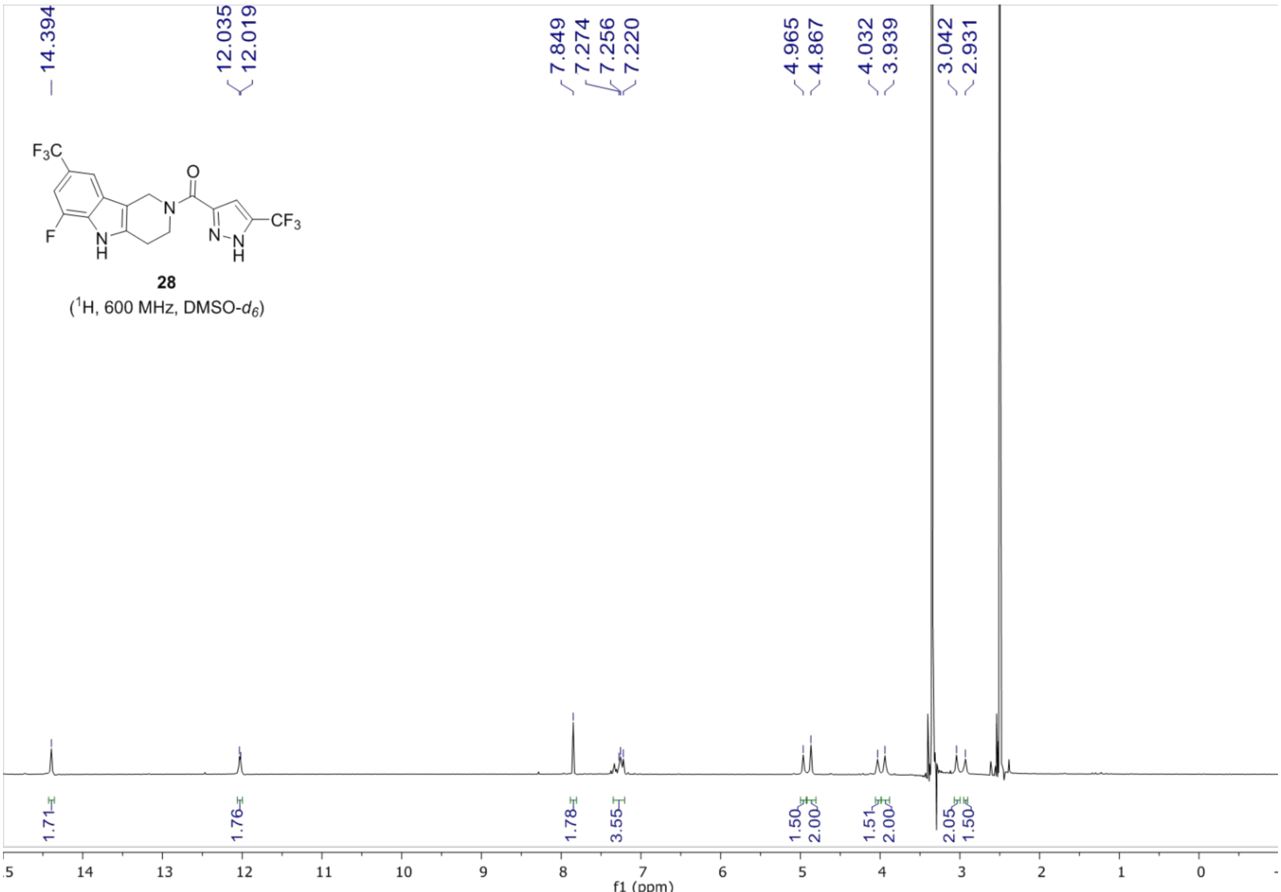

28
(^{1}H, 600 MHz, DMSO-d_6)

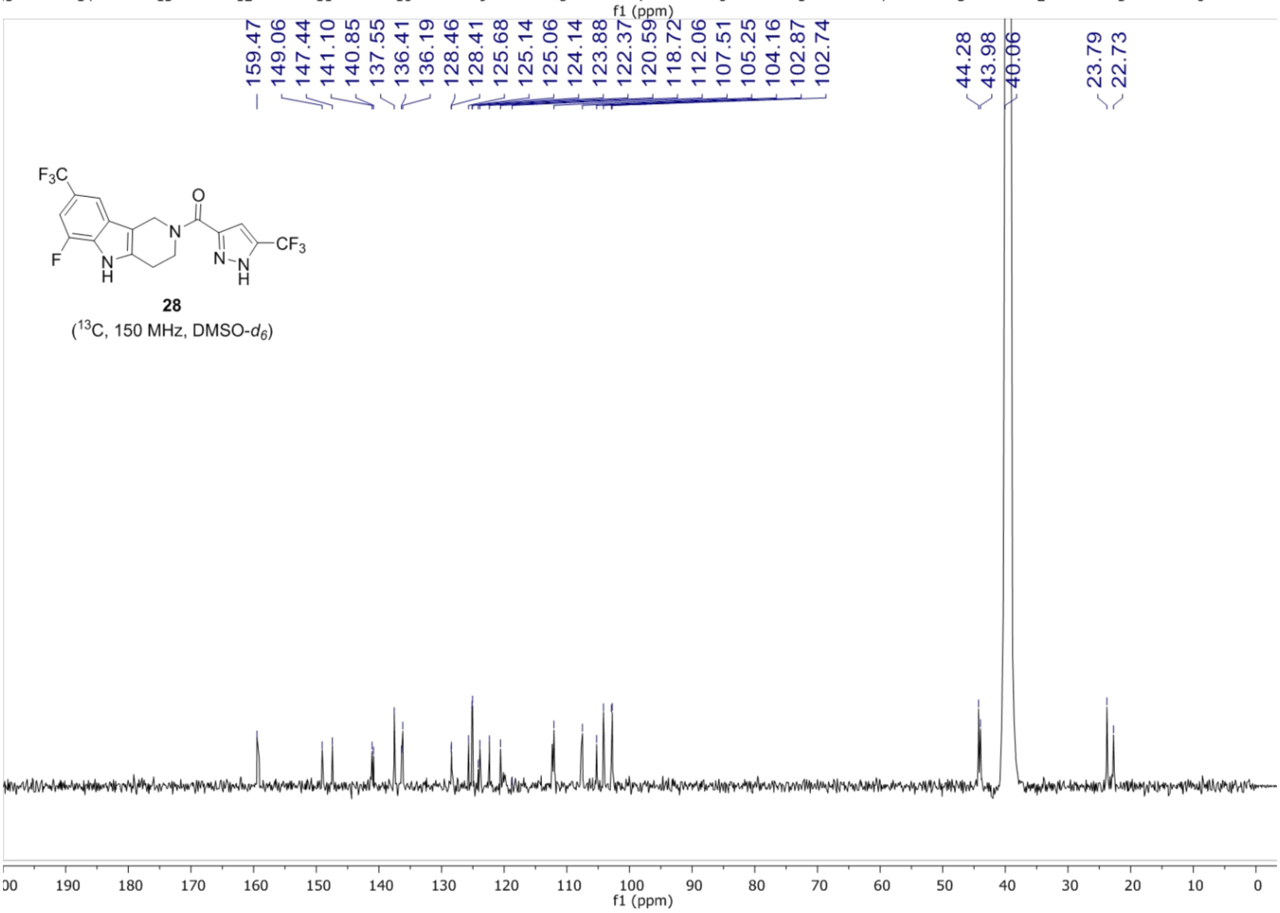

28
(^{13}C, 150 MHz, DMSO-d_6)

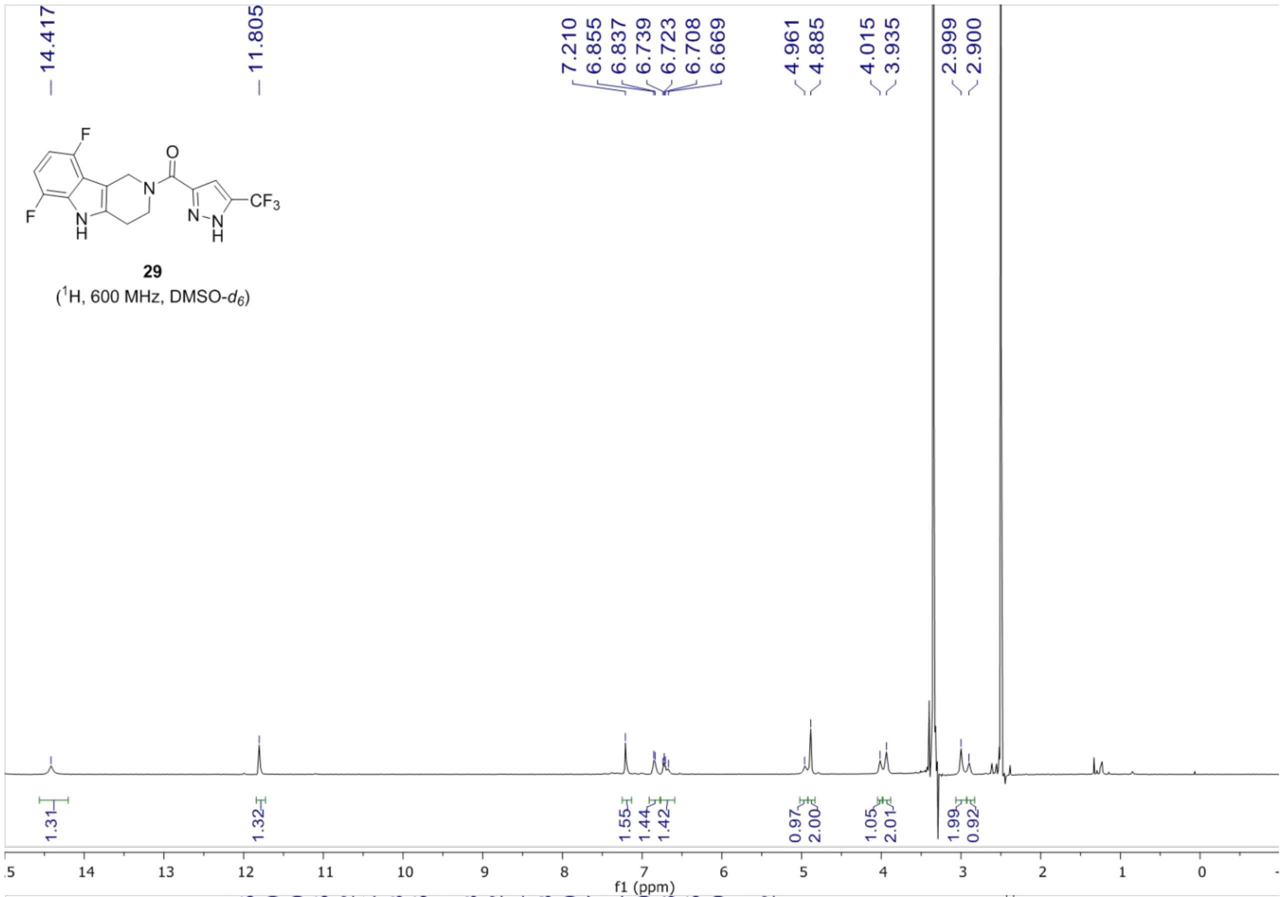
14.417
11.805
7.210
6.855
6.837
6.739
6.723
6.708
6.669
4.961
4.885
4.015
3.935
2.999
2.900
O
F
N
CF3
N
N
H
H
29
(1H, 600 MHz, DMSO-d6)
1.31
1.32
1.55
1.44
1.42
0.97
2.00
1.05
2.01
1.99
0.92
f1 (ppm)

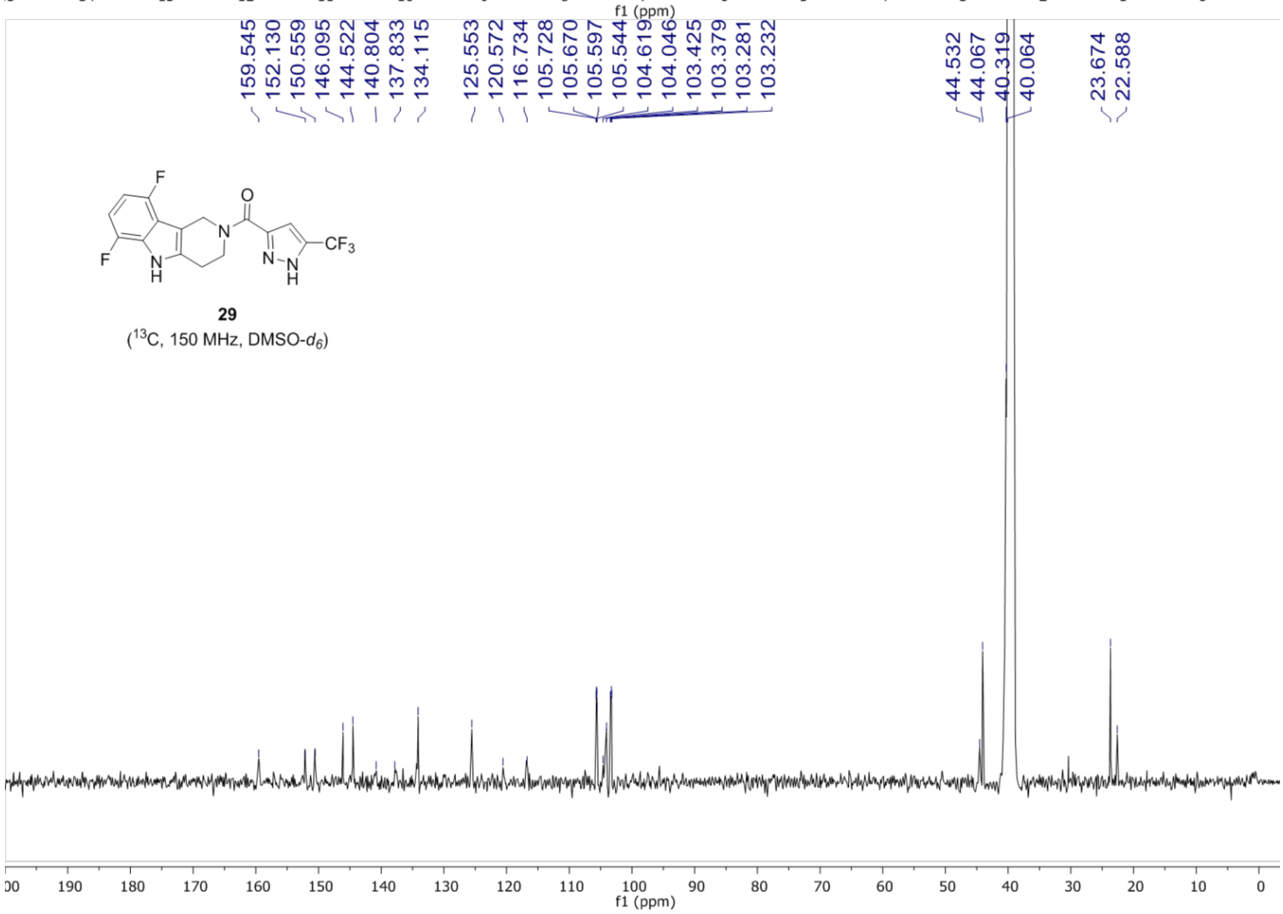
159.545
152.130
150.559
146.095
144.522
140.804
137.833
134.115
125.553
120.572
116.734
105.728
105.670
105.597
105.544
104.619
104.046
103.425
103.379
103.281
103.232
44.532
44.067
40.319
40.064
23.674
22.588
O
F
N
CF3
N
N
H
H
29
(13C, 150 MHz, DMSO-d6)
f1 (ppm)

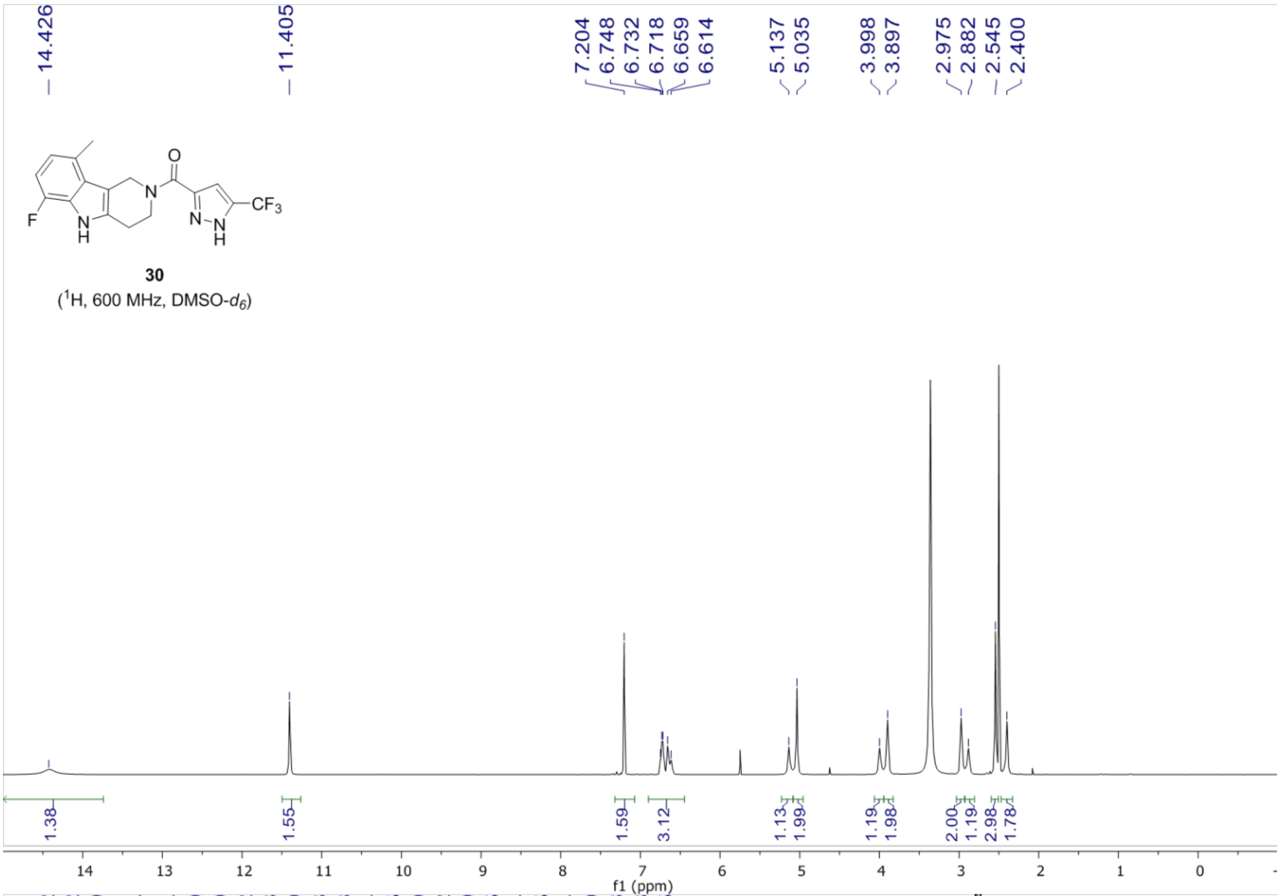
14.426
11.405
7.204
6.748
6.732
6.718
6.659
6.614
5.137
5.035
3.998
3.897
2.975
2.882
2.545
2.400
F
H
N
O
N
N
H
CF3
30
(1H, 600 MHz, DMSO-d6)
1.38
1.55
1.59
3.12
1.13
1.99
1.19
1.98
2.00
1.19
2.98
1.78
f1 (ppm)

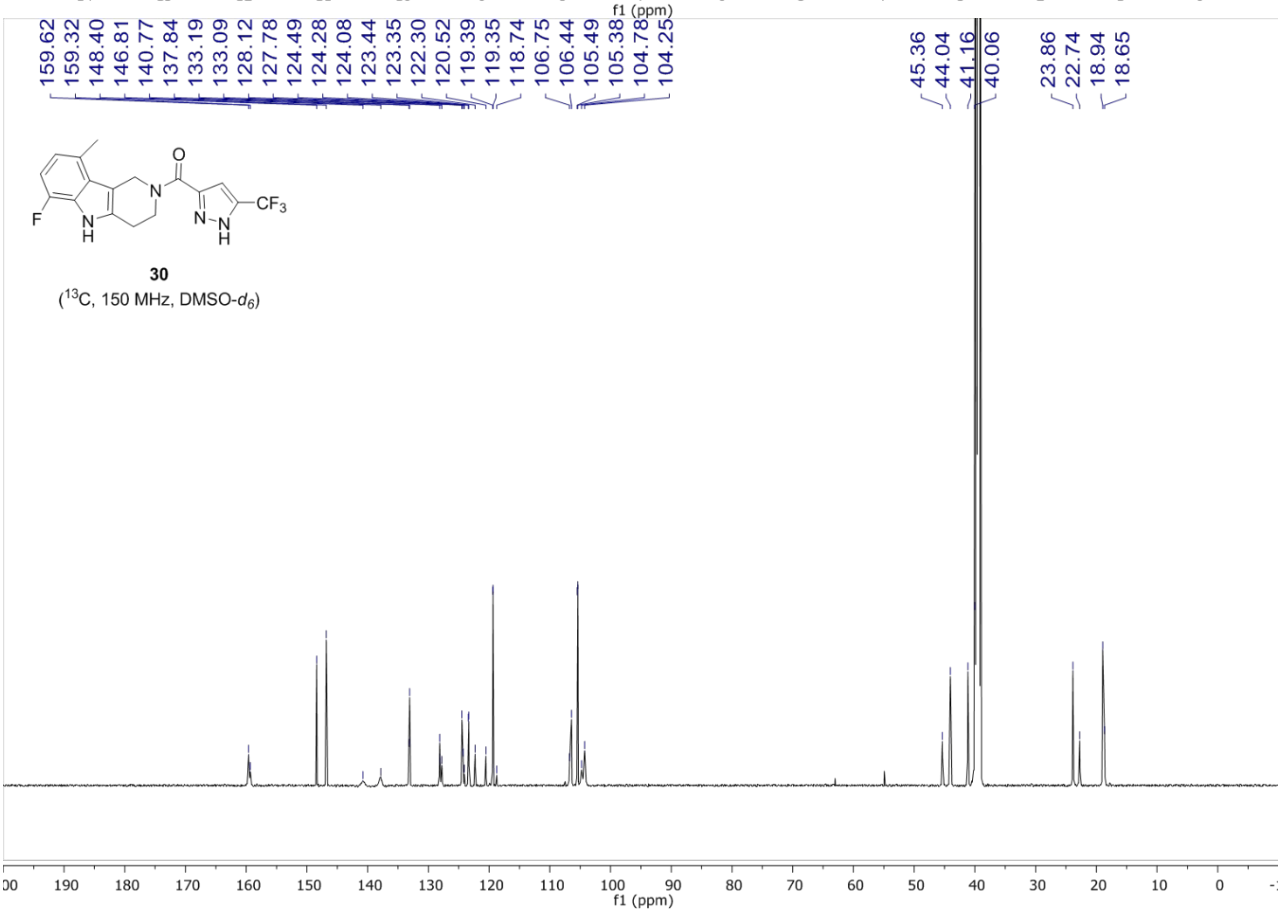
159.62
159.32
148.40
146.81
140.77
137.84
133.19
133.09
128.12
127.78
124.49
124.28
124.08
123.44
123.35
122.30
120.52
119.39
119.35
118.74
106.75
106.44
105.49
105.38
104.78
104.25
45.36
44.04
41.16
40.06
23.86
22.74
18.94
18.65
30
(13C, 150 MHz, DMSO-d6)
f1 (ppm)

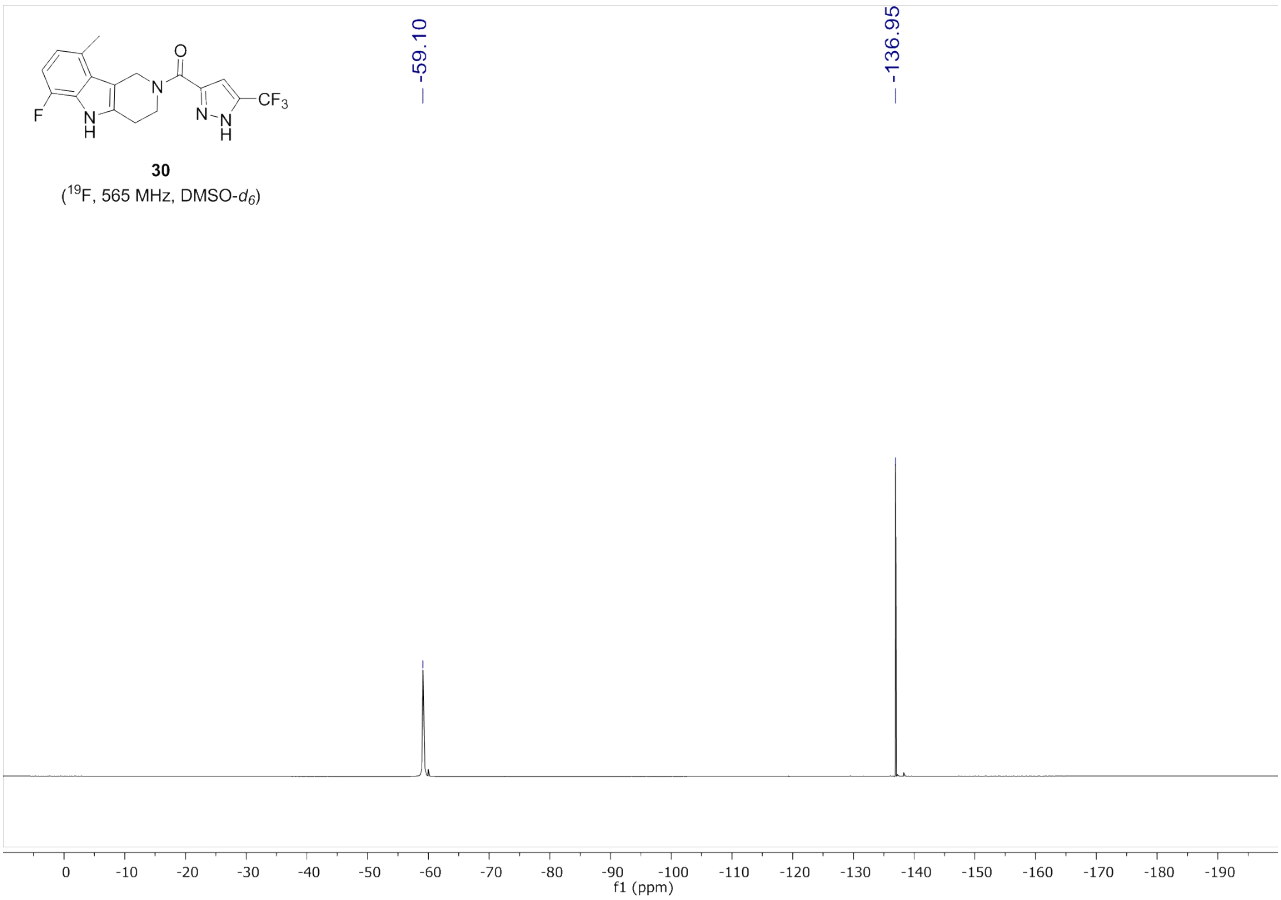

CF3
F
N
H
N
O
N
N
H
30
(19F, 565 MHz, DMSO-d6)
-59.10
-136.95
0
-10
-20
-30
-40
-50
-60
-70
-80
-90
-100
-110
-120
-130
-140
-150
-160
-170
-180
-190
f1 (ppm)

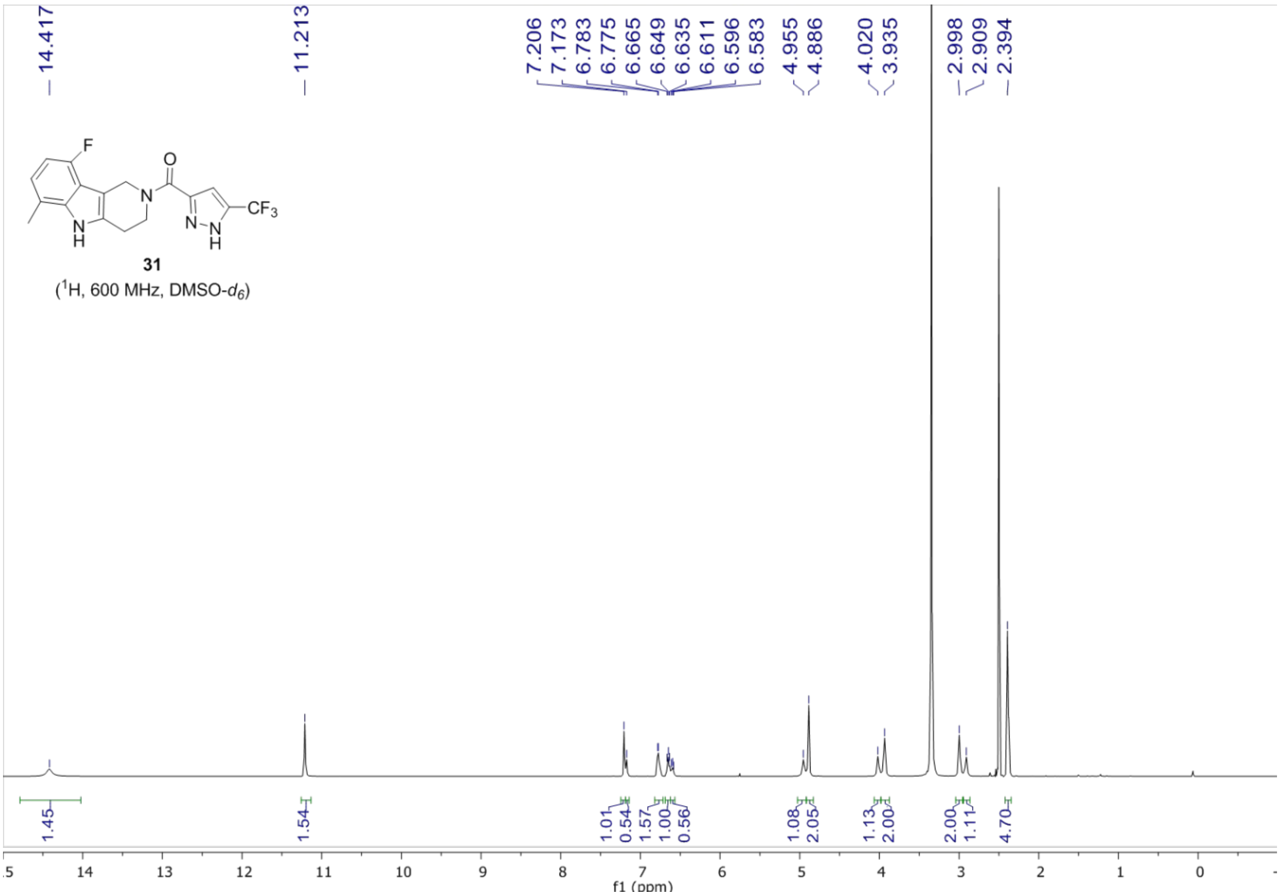
14.417
11.213
7.206 7.173 6.783 6.775 6.665 6.649 6.635 6.611 6.596 6.583
4.955 4.886
4.020 3.935
2.998 2.909 2.394
F
O
N
CF3
NH
N
N H
31
(1H, 600 MHz, DMSO-d6)
1.45
1.54
1.01 0.54 1.57 1.00 0.56
1.08 2.05
1.13 2.00
2.00 1.11
4.70
f1 (ppm)

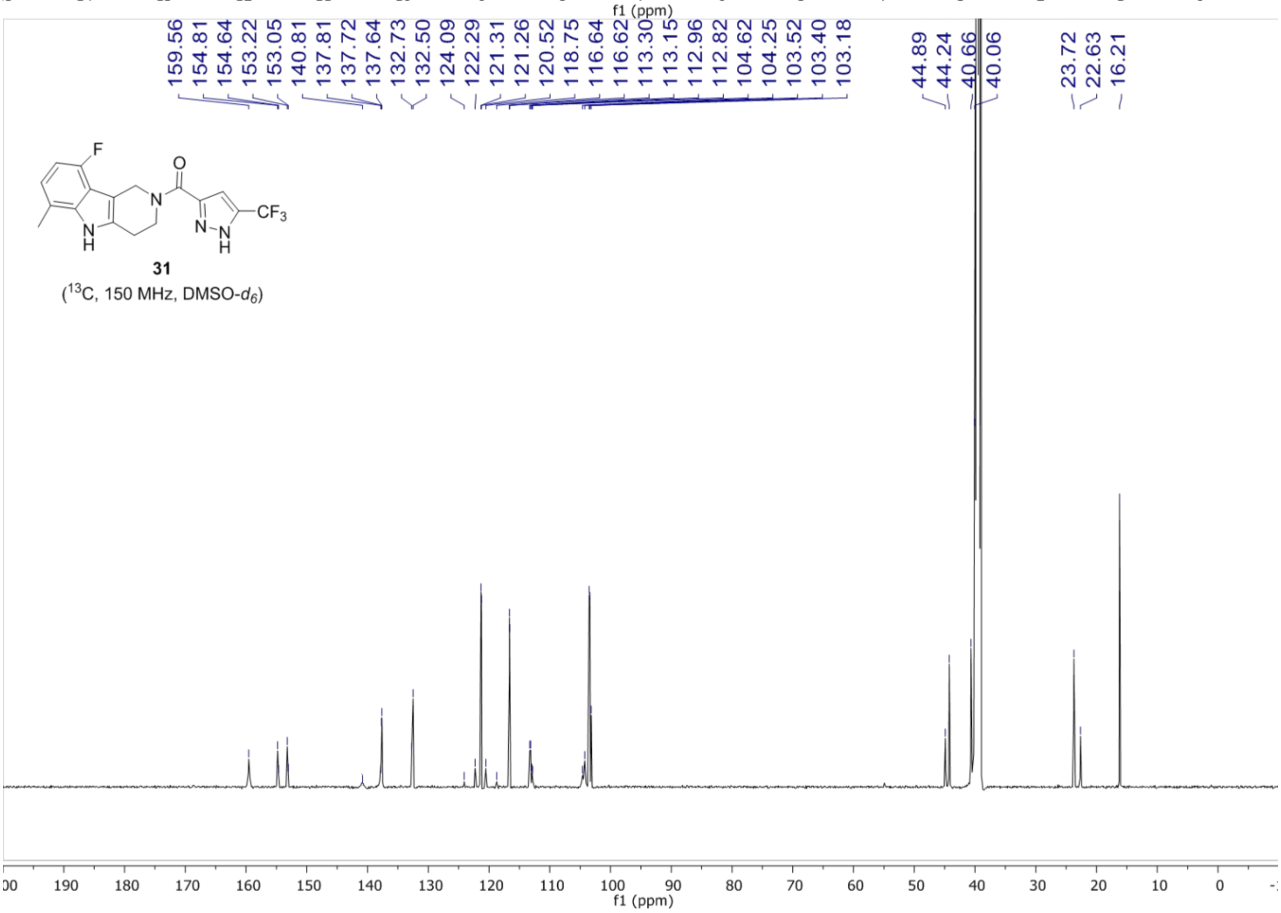
159.56 154.81 154.64 153.22 153.05 140.81 137.81 137.72 137.64 132.73 132.50 124.09 122.29 121.31 121.26 120.52 118.75 116.64 116.62 113.30 113.15 112.96 112.82 104.62 104.25 103.52 103.40 103.18
44.89 44.24 40.66 40.06
23.72 22.63 16.21
F
O
N
CF3
NH
N
N H
31
(13C, 150 MHz, DMSO-d6)
f1 (ppm)

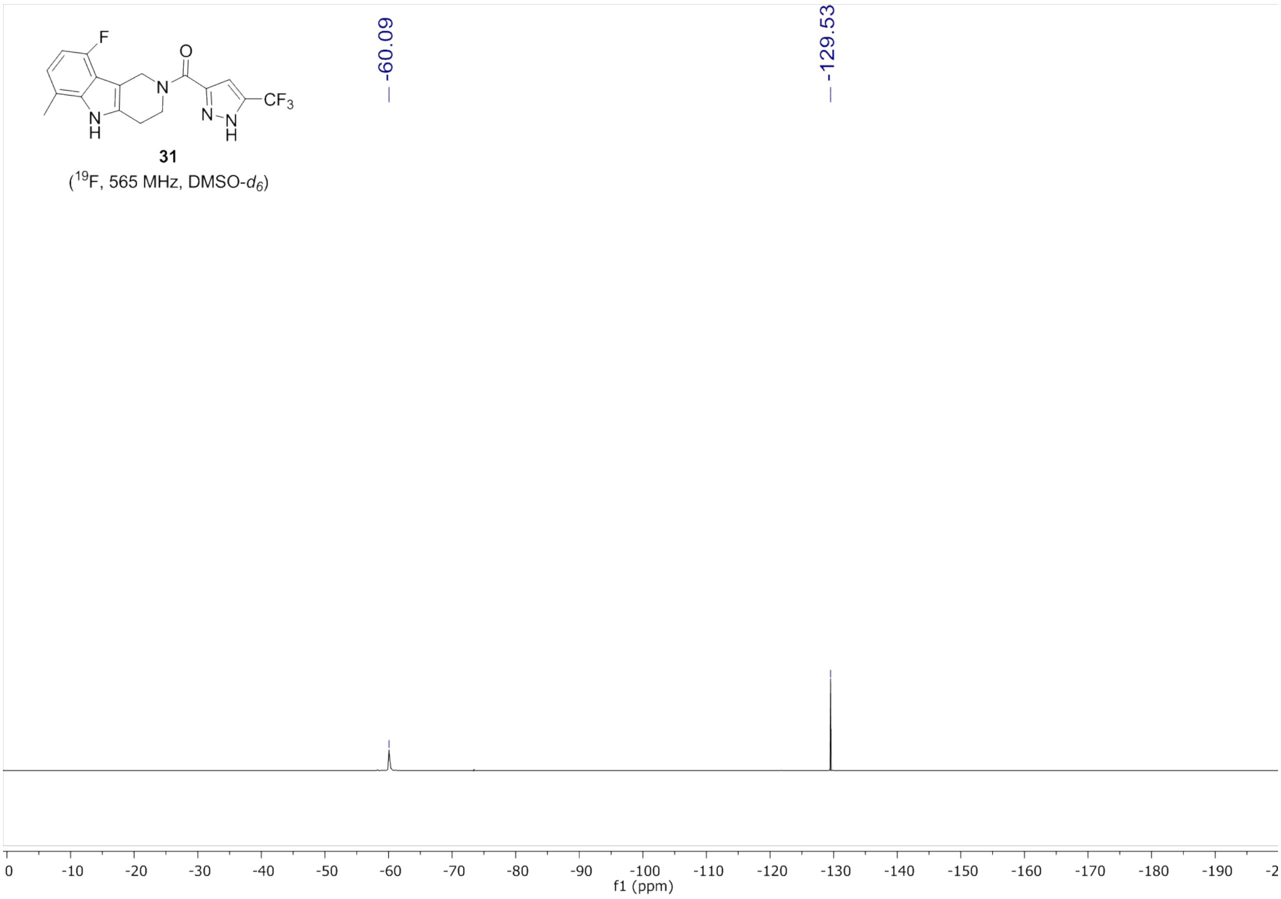
F
O
N
N
N
H
CF3
N
H
31
(19F, 565 MHz, DMSO-d6)
-60.09
-129.53
0
-10
-20
-30
-40
-50
-60
-70
-80
-90
-100
-110
-120
-130
-140
-150
-160
-170
-180
-190
-2
f1 (ppm)

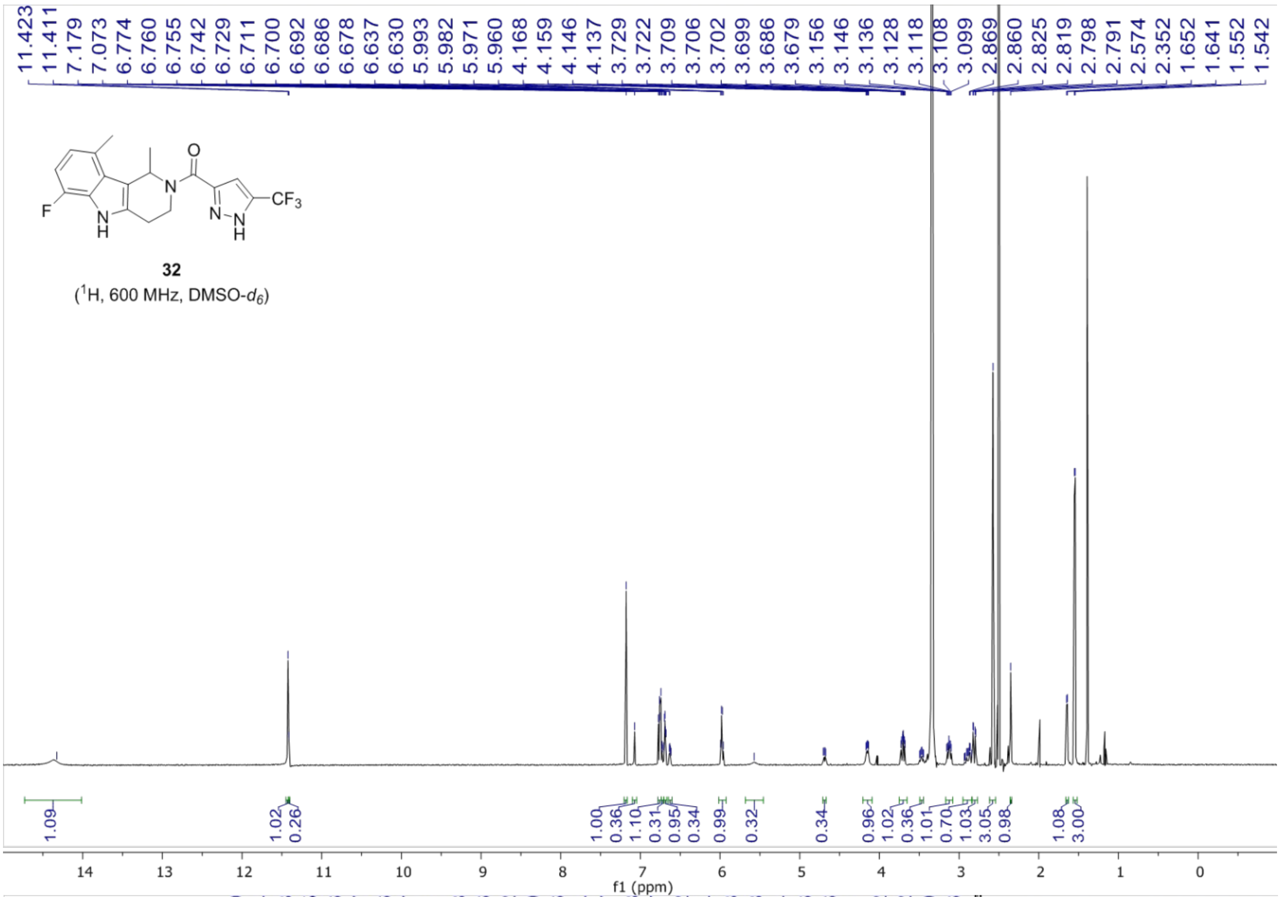
11.423 11.411 7.179 7.073 6.774 6.760 6.755 6.742 6.729 6.711 6.700 6.692 6.686 6.678 6.637 6.630 5.993 5.982 5.971 5.960 4.168 4.159 4.146 4.137 3.729 3.722 3.709 3.706 3.702 3.699 3.686 3.679 3.156 3.146 3.136 3.128 3.118 3.108 3.099 2.869 2.860 2.825 2.819 2.798 2.791 2.574 2.352 1.652 1.641 1.552 1.542
F
N
H
O
N
N
N
H
CF3
32
(1H, 600 MHz, DMSO-d6)
1.09 1.02 0.26 1.00 0.36 1.10 0.31 0.95 0.34 0.99 0.32 0.34 0.96 1.02 0.36 1.01 0.70 1.03 3.05 0.98 1.08 3.00
14 13 12 11 10 9 8 7 6 5 4 3 2 1 0
f1 (ppm)

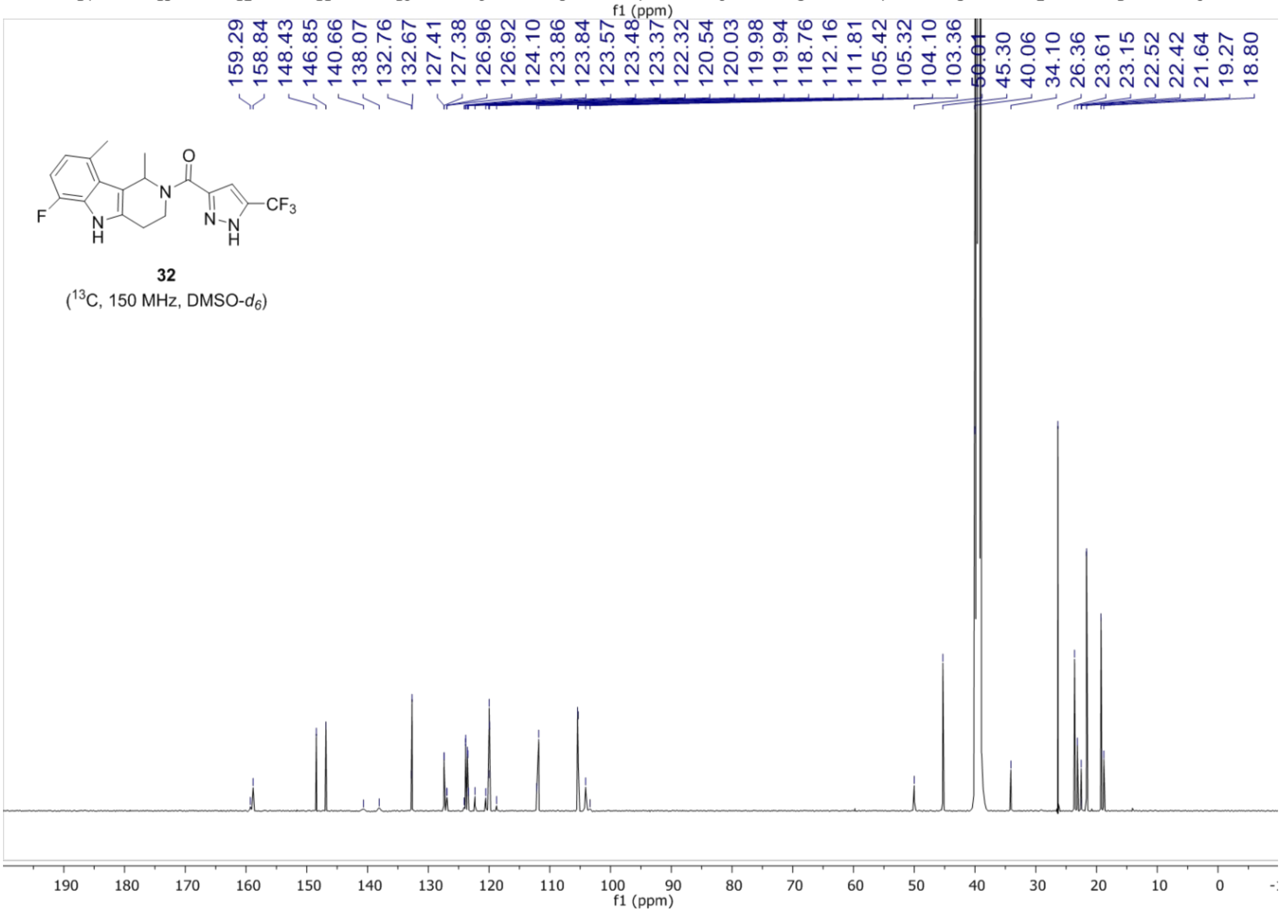
159.29 158.84 148.43 146.85 140.66 138.07 132.76 132.67 127.41 127.38 126.96 126.92 124.10 123.86 123.84 123.57 123.48 123.37 122.32 120.54 120.03 119.98 119.94 118.76 112.16 111.81 105.42 105.32 104.10 103.36 50.01 45.30 40.06 34.10 26.36 23.61 23.15 22.52 22.42 21.64 19.27 18.80
F
N
H
O
N
N
N
H
CF3
32
(13C, 150 MHz, DMSO-d6)
190 180 170 160 150 140 130 120 110 100 90 80 70 60 50 40 30 20 10 0
f1 (ppm)

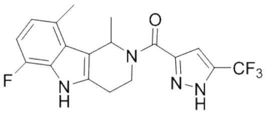

32

(^{19}F, 565 MHz, DMSO-d_6)

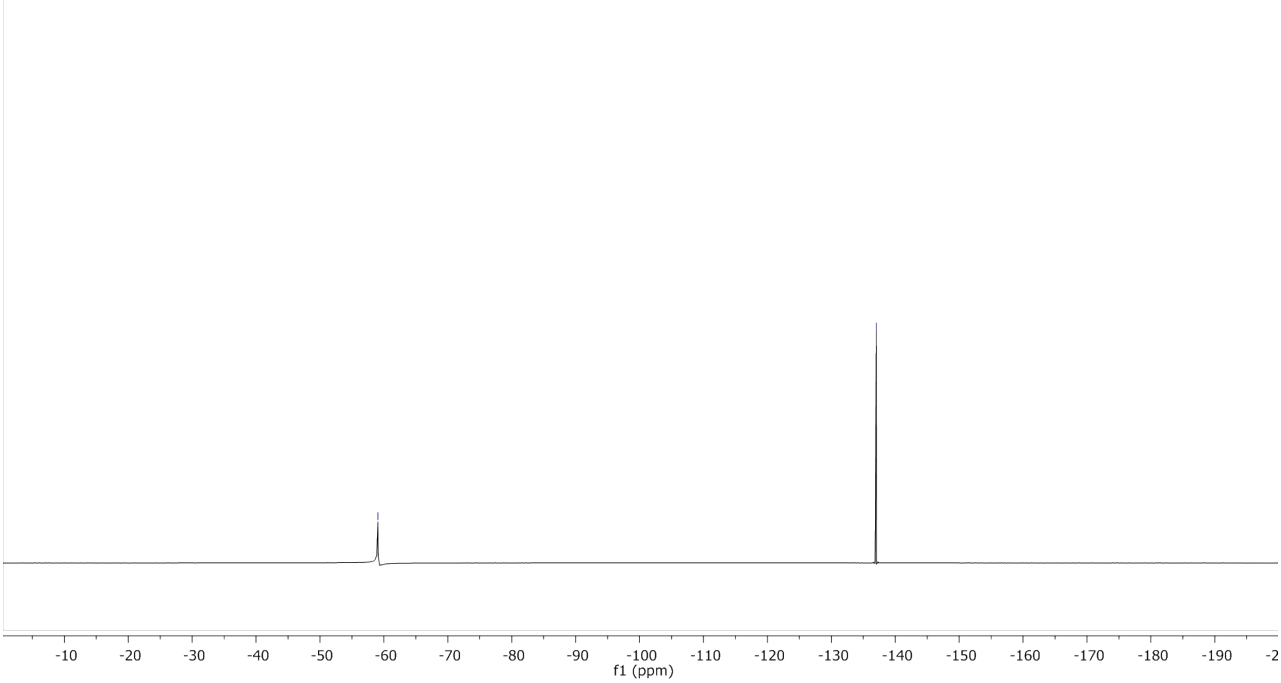

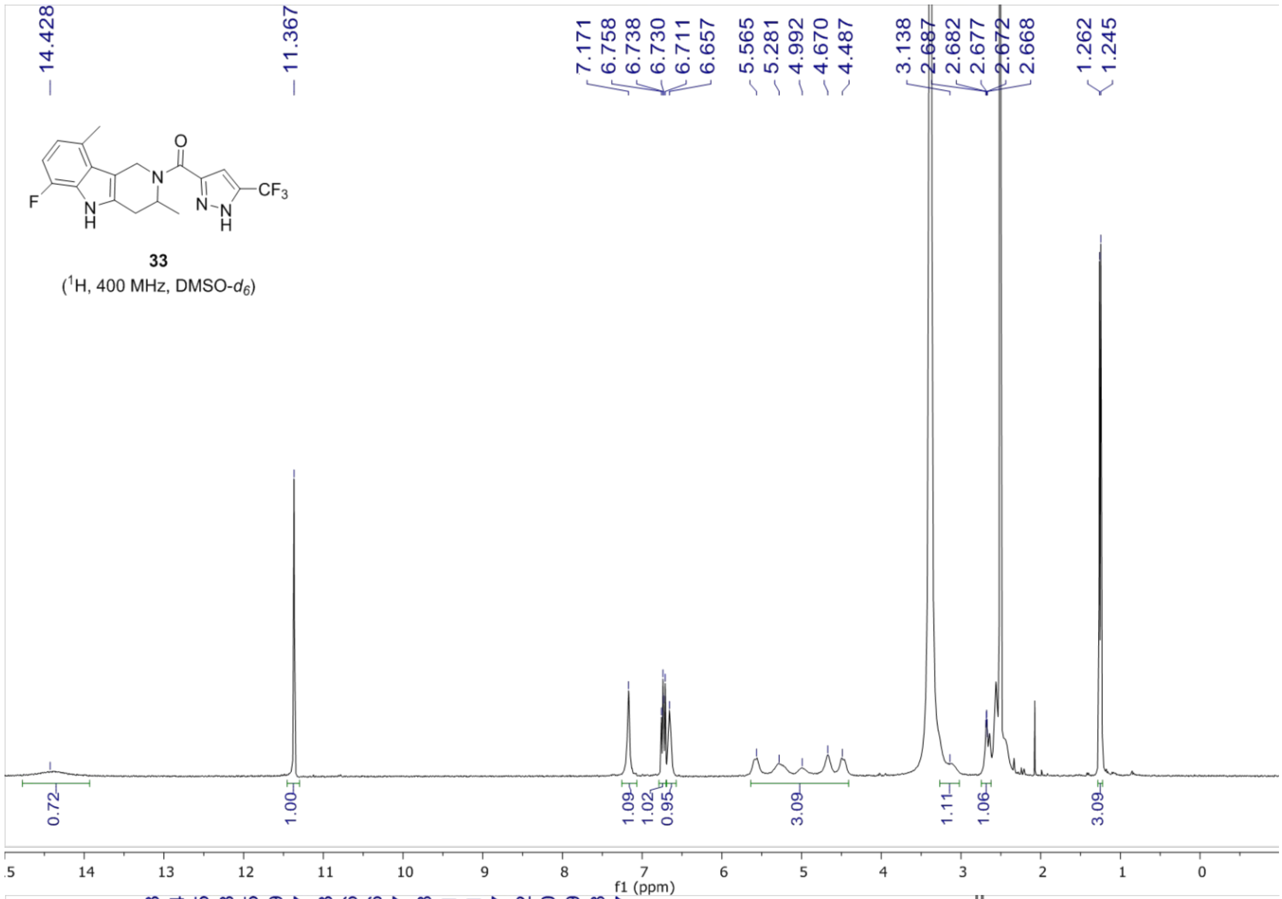

14.428
11.367
7.171
6.758
6.738
6.730
6.711
6.657
5.565
5.281
4.992
4.670
4.487
3.138
2.687
2.682
2.677
2.672
2.668
1.262
1.245
F
N
H
N
O
N
N
H
CF3
33
(1H, 400 MHz, DMSO-d6)
0.72
1.00
1.09
1.02
0.95
3.09
1.11
1.06
3.09
f1 (ppm)

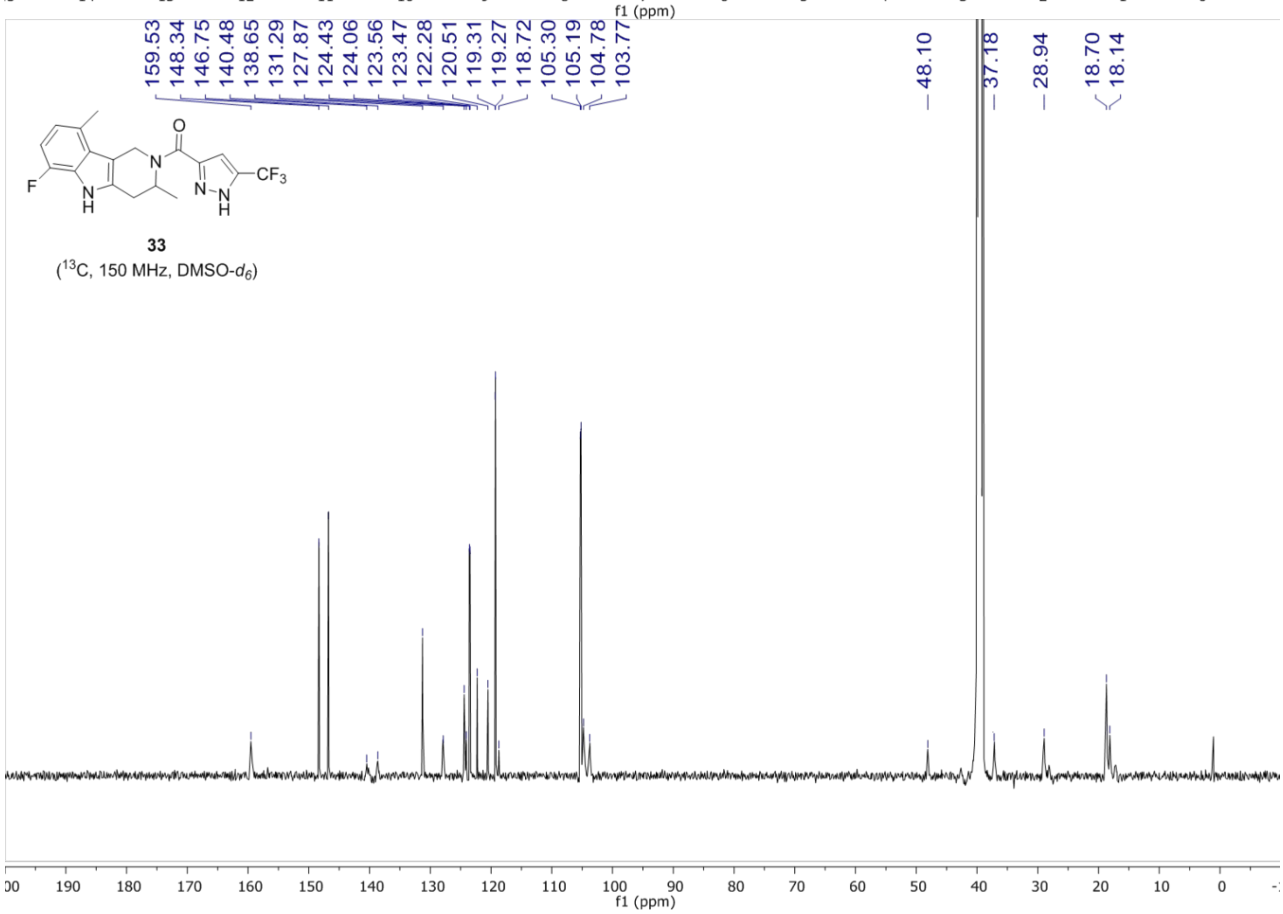

159.53
148.34
146.75
140.48
138.65
131.29
127.87
124.43
124.06
123.56
123.47
122.28
120.51
119.31
119.27
118.72
105.30
105.19
104.78
103.77
48.10
37.18
28.94
18.70
18.14
33
(13C, 150 MHz, DMSO-d6)
f1 (ppm)

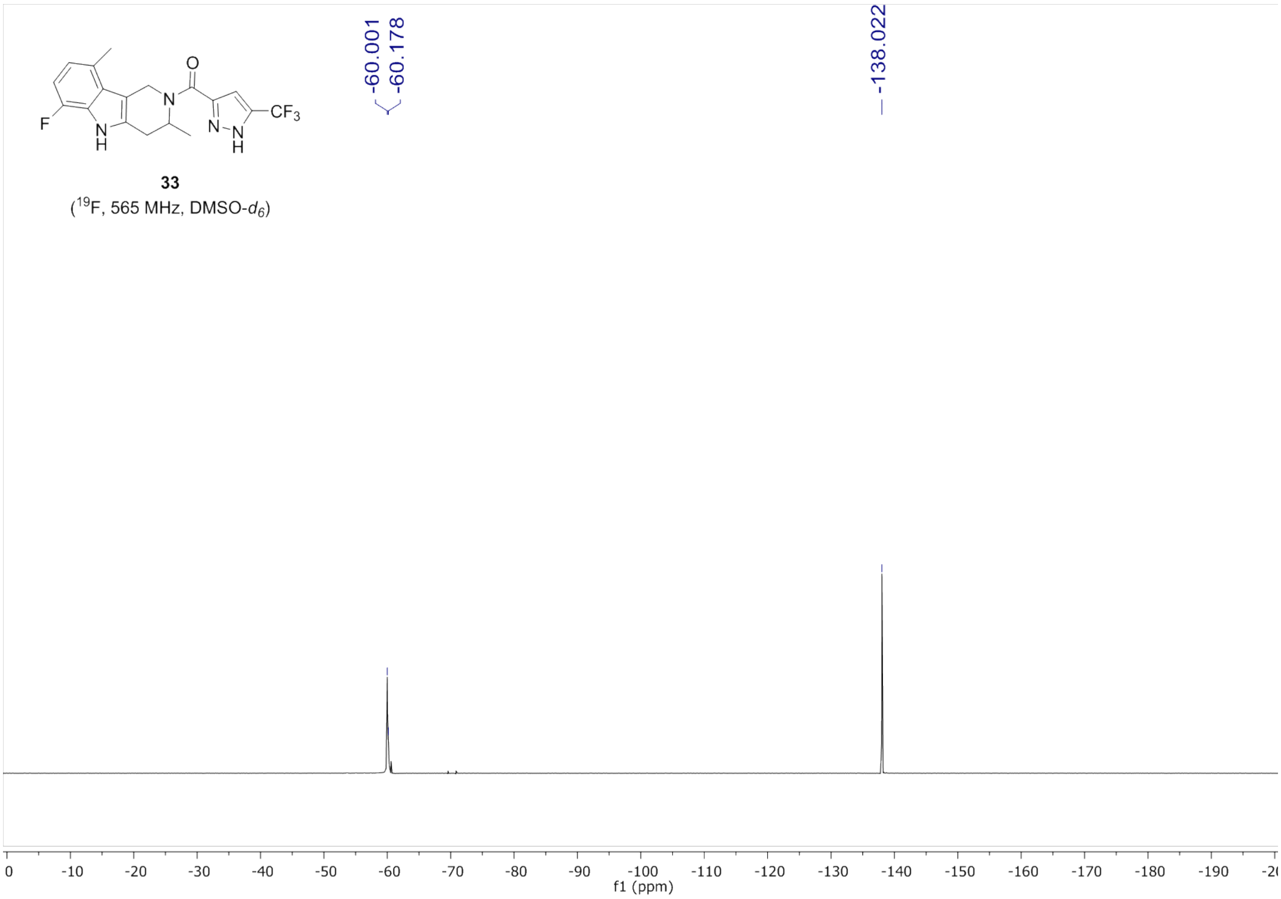
33
(19F, 565 MHz, DMSO-d6)
-60.001
-60.178
-138.022
0
-10
-20
-30
-40
-50
-60
-70
-80
-90
-100
-110
-120
-130
-140
-150
-160
-170
-180
-190
f1 (ppm)

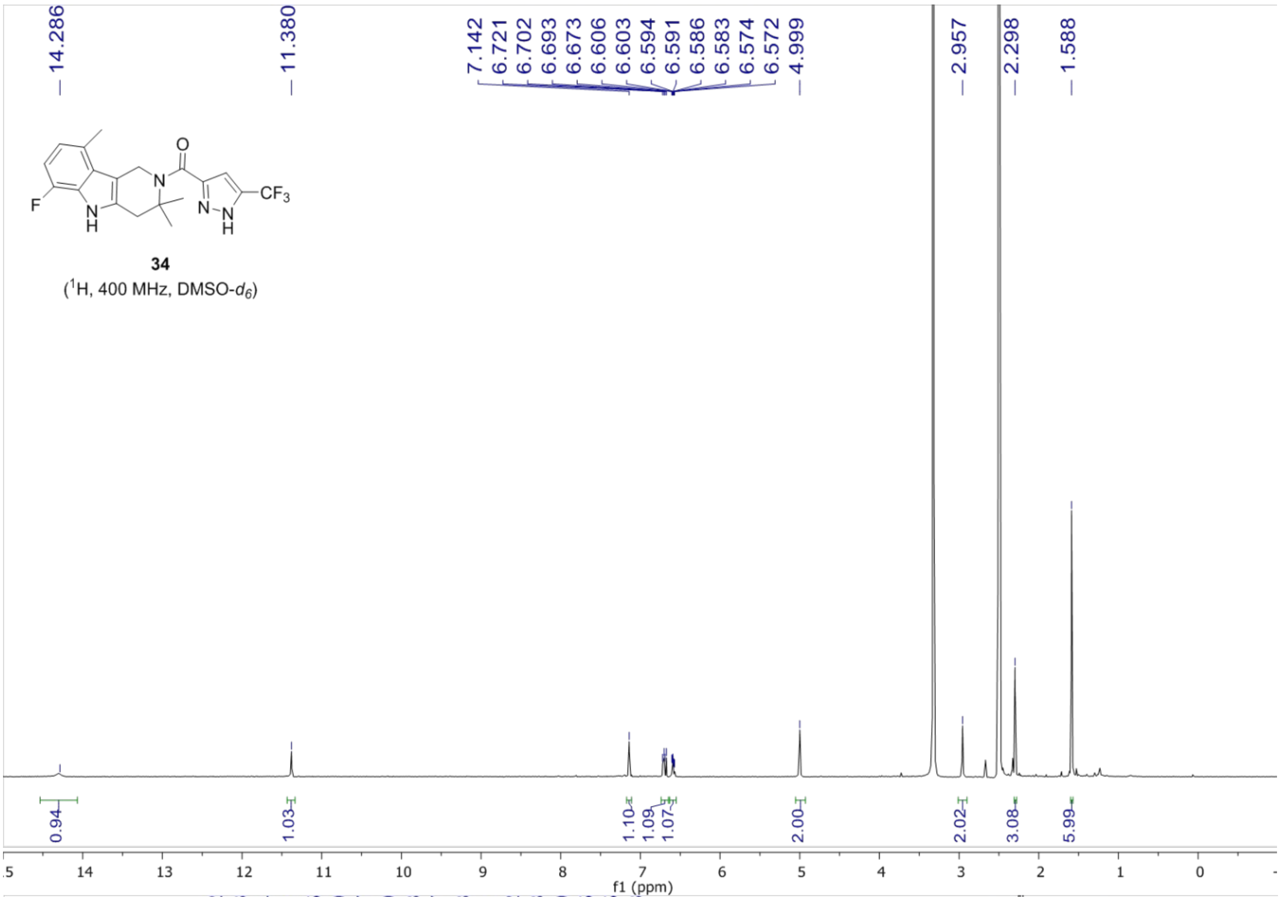
14.286
11.380
7.142
6.721
6.702
6.693
6.673
6.606
6.603
6.594
6.591
6.586
6.583
6.574
6.572
4.999
2.957
2.298
1.588
O
N
F
N
H
N
N
H
CF3
34
(1H, 400 MHz, DMSO-d6)
0.94
1.03
1.10
1.09
1.07
2.00
2.02
3.08
5.99
f1 (ppm)

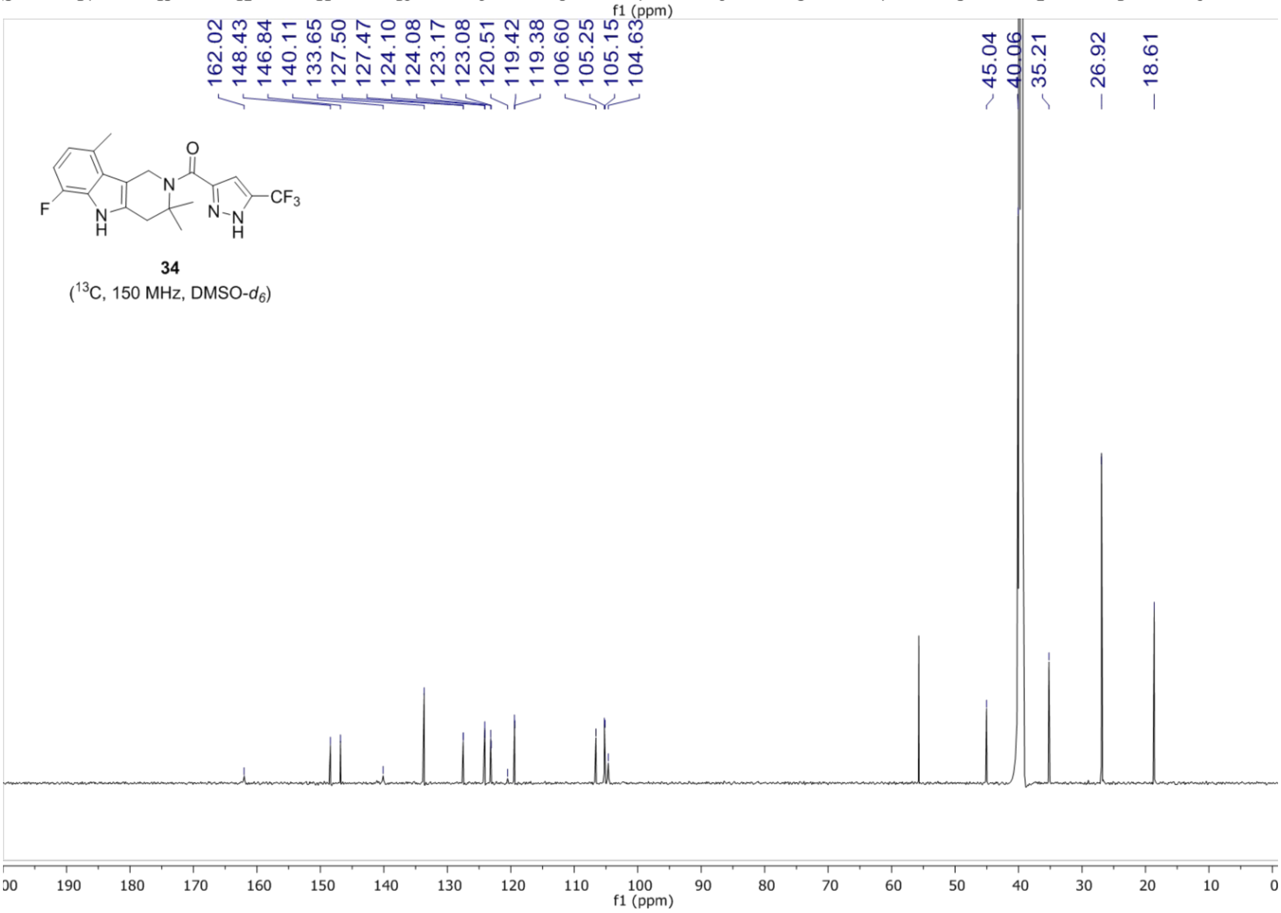
162.02
148.43
146.84
140.11
133.65
127.50
127.47
124.10
124.08
123.17
123.08
120.51
119.42
119.38
106.60
105.25
105.15
104.63
45.04
40.06
35.21
26.92
18.61
O
N
F
N
H
N
N
H
CF3
34
(13C, 150 MHz, DMSO-d6)
f1 (ppm)

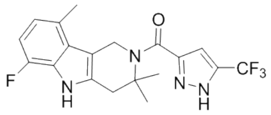

34
(^{19}F, 565 MHz, DMSO-d_6)

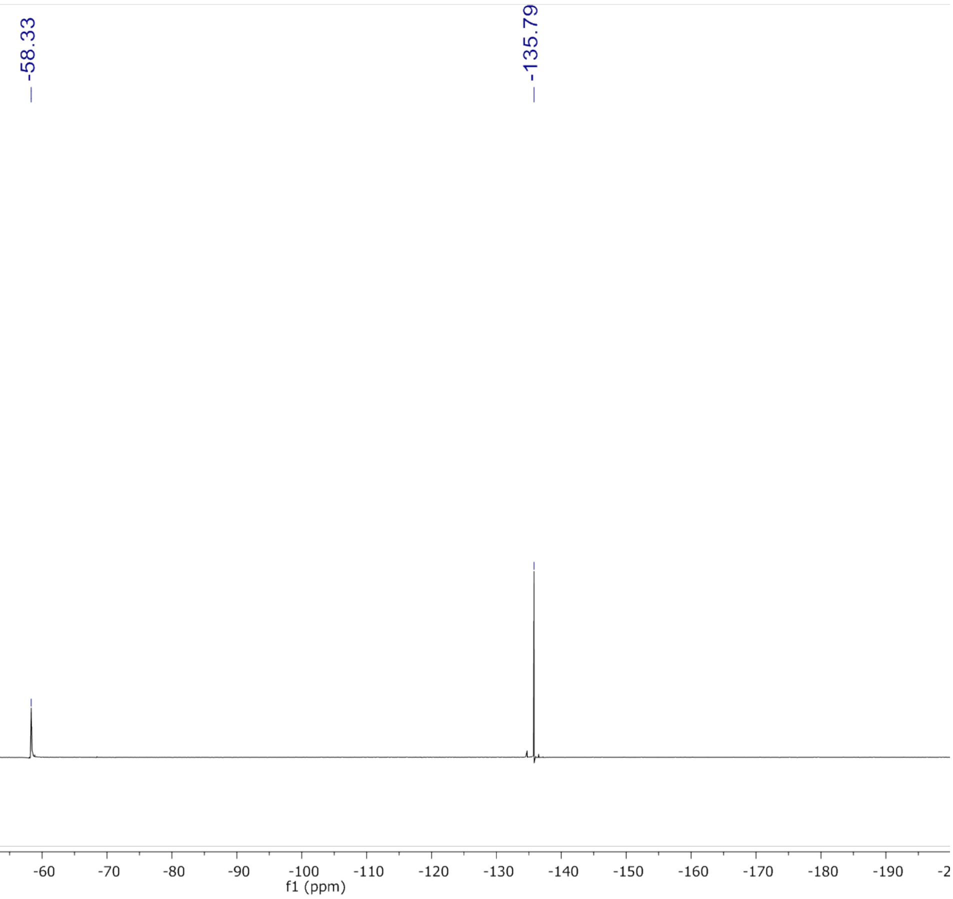

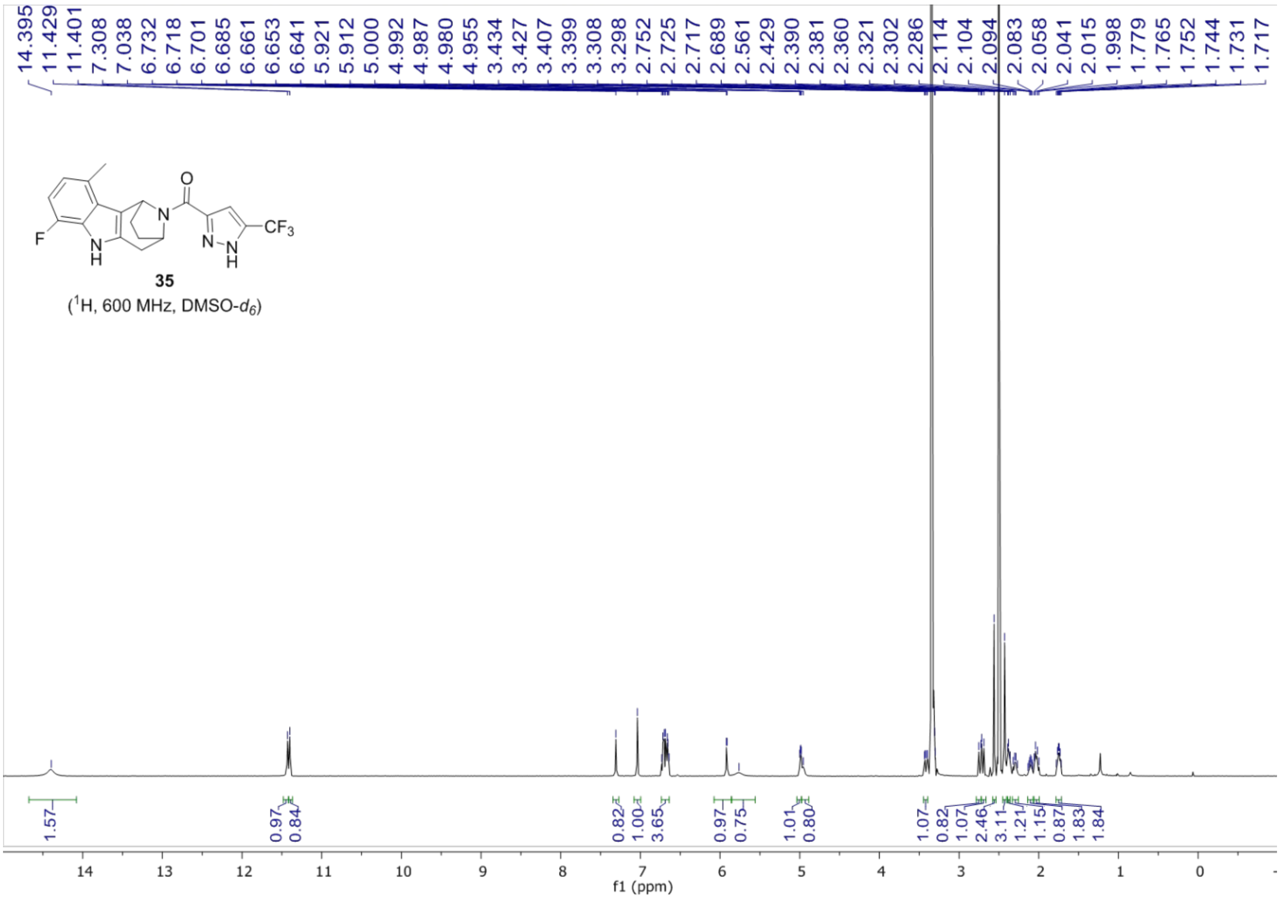
14.395
11.429
11.401
7.308
7.038
6.732
6.718
6.701
6.685
6.661
6.653
6.641
5.921
5.912
5.000
4.992
4.987
4.980
4.955
3.434
3.427
3.407
3.399
3.308
3.298
2.752
2.725
2.717
2.689
2.561
2.429
2.390
2.381
2.360
2.321
2.302
2.286
2.114
2.104
2.094
2.083
2.058
2.041
2.015
1.998
1.779
1.765
1.752
1.744
1.731
1.717
F
N
H
N
O
N
N
H
CF3
35
(1H, 600 MHz, DMSO-d6)
1.57
0.97
0.84
0.82
1.00
3.65
0.97
0.75
1.01
0.80
1.07
0.82
1.07
2.46
3.11
1.21
1.15
0.87
1.83
1.84
14
13
12
11
10
9
8
7
6
5
4
3
2
1
0
f1 (ppm)

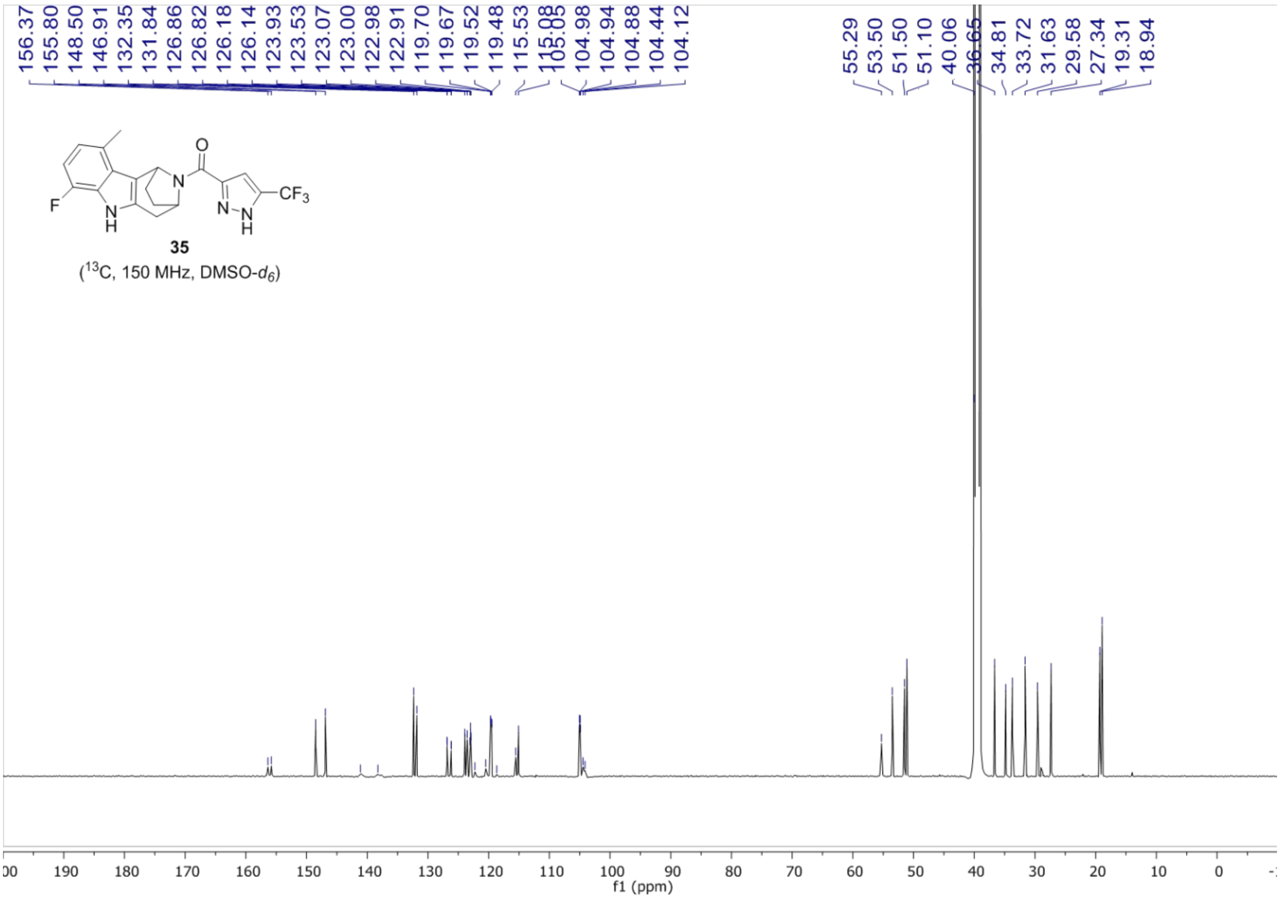
156.37
155.80
148.50
146.91
132.35
131.84
126.86
126.82
126.18
126.14
123.93
123.53
123.07
123.00
122.98
122.91
119.70
119.67
119.52
119.48
115.53
115.08
105.05
104.98
104.94
104.88
104.44
104.12
55.29
53.50
51.50
51.10
40.06
36.65
34.81
33.72
31.63
29.58
27.34
19.31
18.94
F
N
H
N
O
N
N
H
CF3
35
(13C, 150 MHz, DMSO-d6)
190
180
170
160
150
140
130
120
110
100
90
80
70
60
50
40
30
20
10
0
f1 (ppm)

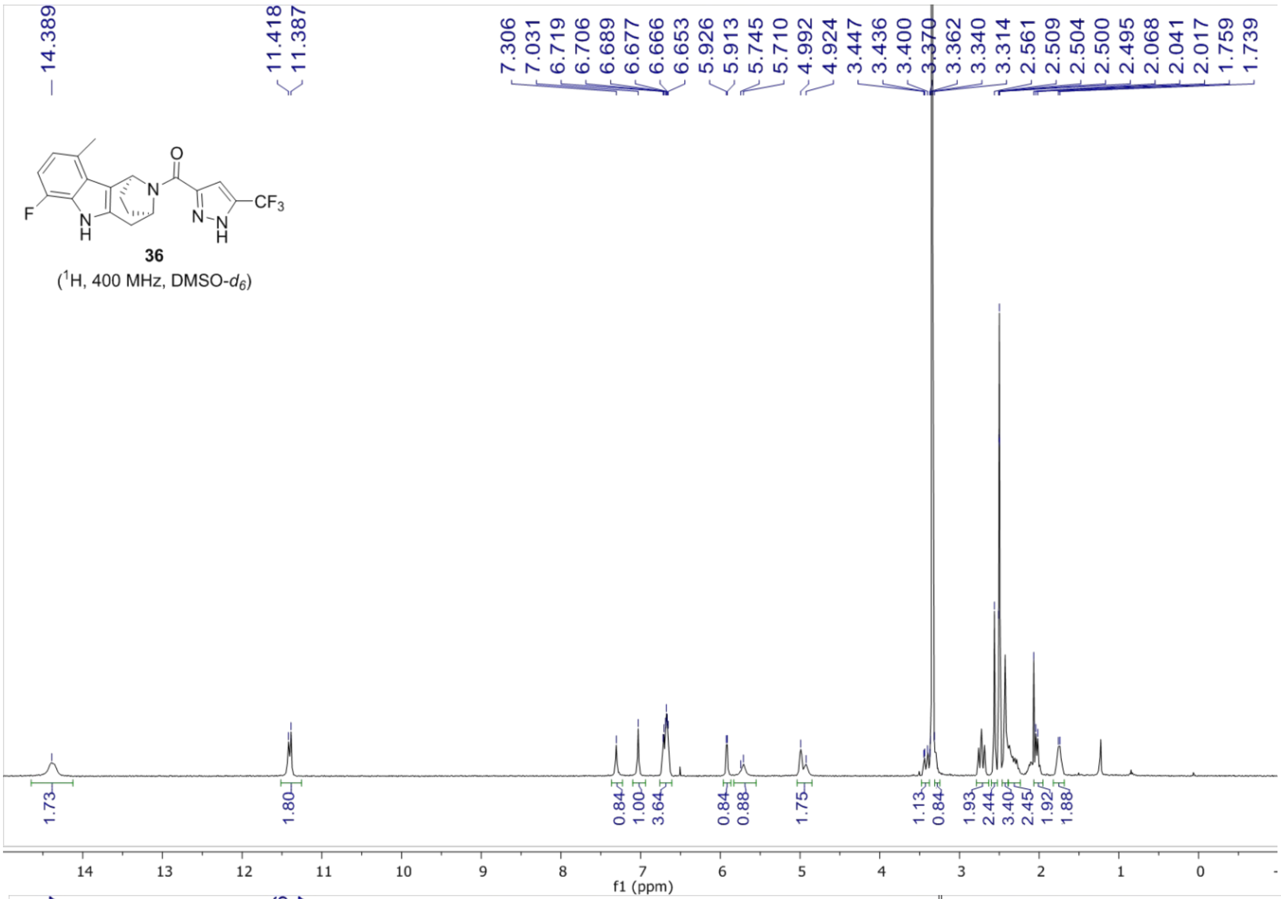
14.389
11.418
11.387
7.306 7.031 6.719 6.706 6.689 6.677 6.666 6.653 5.926 5.913 5.745 5.710 4.992 4.924 3.447 3.436 3.400 3.370 3.362 3.340 3.314 2.561 2.509 2.504 2.500 2.495 2.068 2.041 2.017 1.759 1.739
F
N
H
O
N
N
N
H
CF3
36
(1H, 400 MHz, DMSO-d6)
1.73
1.80
0.84 1.00 3.64
0.84 0.88
1.75
1.13 0.84
1.93 2.44 3.40 2.45 1.92 1.88
14 13 12 11 10 9 8 7 6 5 4 3 2 1 0
f1 (ppm)

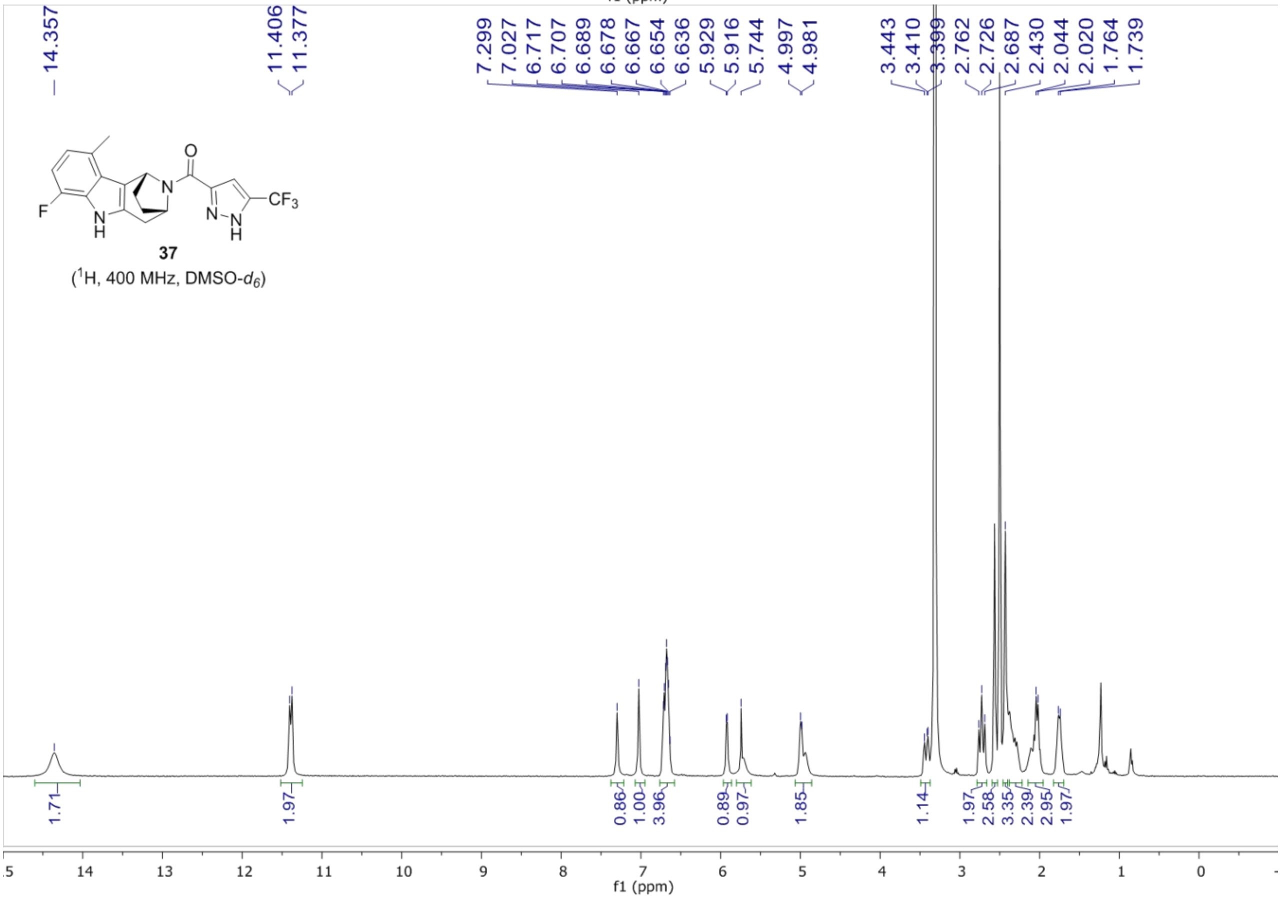
14.357
11.406
11.377
7.299 7.027 6.717 6.707 6.689 6.678 6.667 6.654 6.636 5.929 5.916 5.744 4.997 4.981
3.443 3.410 3.399 2.762 2.726 2.687 2.430 2.044 2.020 1.764 1.739
F
N
H
O
N
N
N
H
CF3
37
(1H, 400 MHz, DMSO-d6)
1.71
1.97
0.86 1.00 3.96
0.89 0.97
1.85
1.14
1.97 2.58 3.35 2.39 2.95 1.97
14 13 12 11 10 9 8 7 6 5 4 3 2 1 0
f1 (ppm)

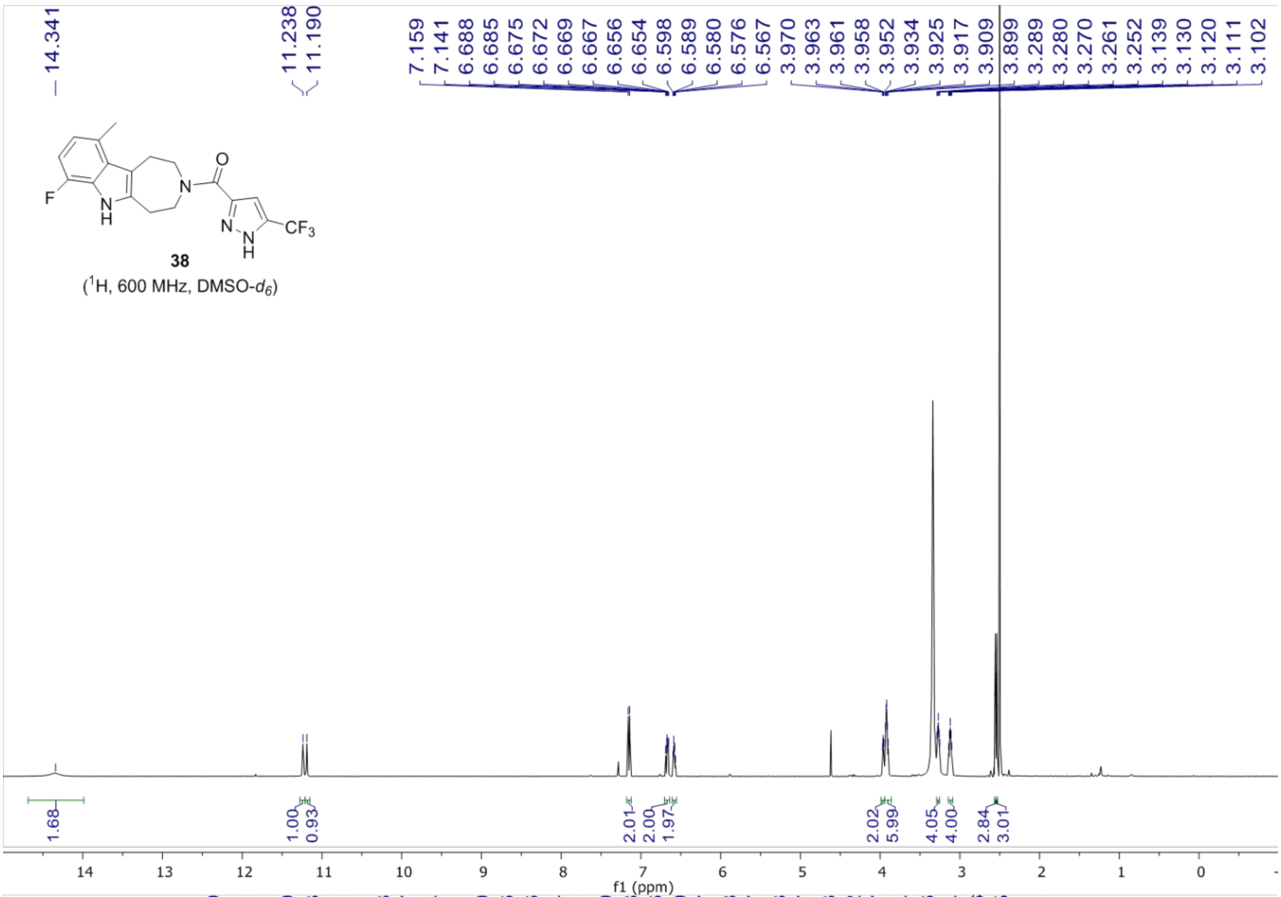
14.341
11.238
11.190
7.159
7.141
6.688
6.685
6.675
6.672
6.669
6.667
6.656
6.654
6.598
6.589
6.580
6.576
6.567
3.970
3.963
3.961
3.958
3.952
3.934
3.925
3.917
3.909
3.899
3.289
3.280
3.270
3.261
3.252
3.139
3.130
3.120
3.111
3.102
F
NH
N
O
N
N
H
CF3
38
(1H, 600 MHz, DMSO-d6)
1.68
1.00
0.93
2.01
2.00
1.97
2.02
5.99
4.05
4.00
2.84
3.01
f1 (ppm)

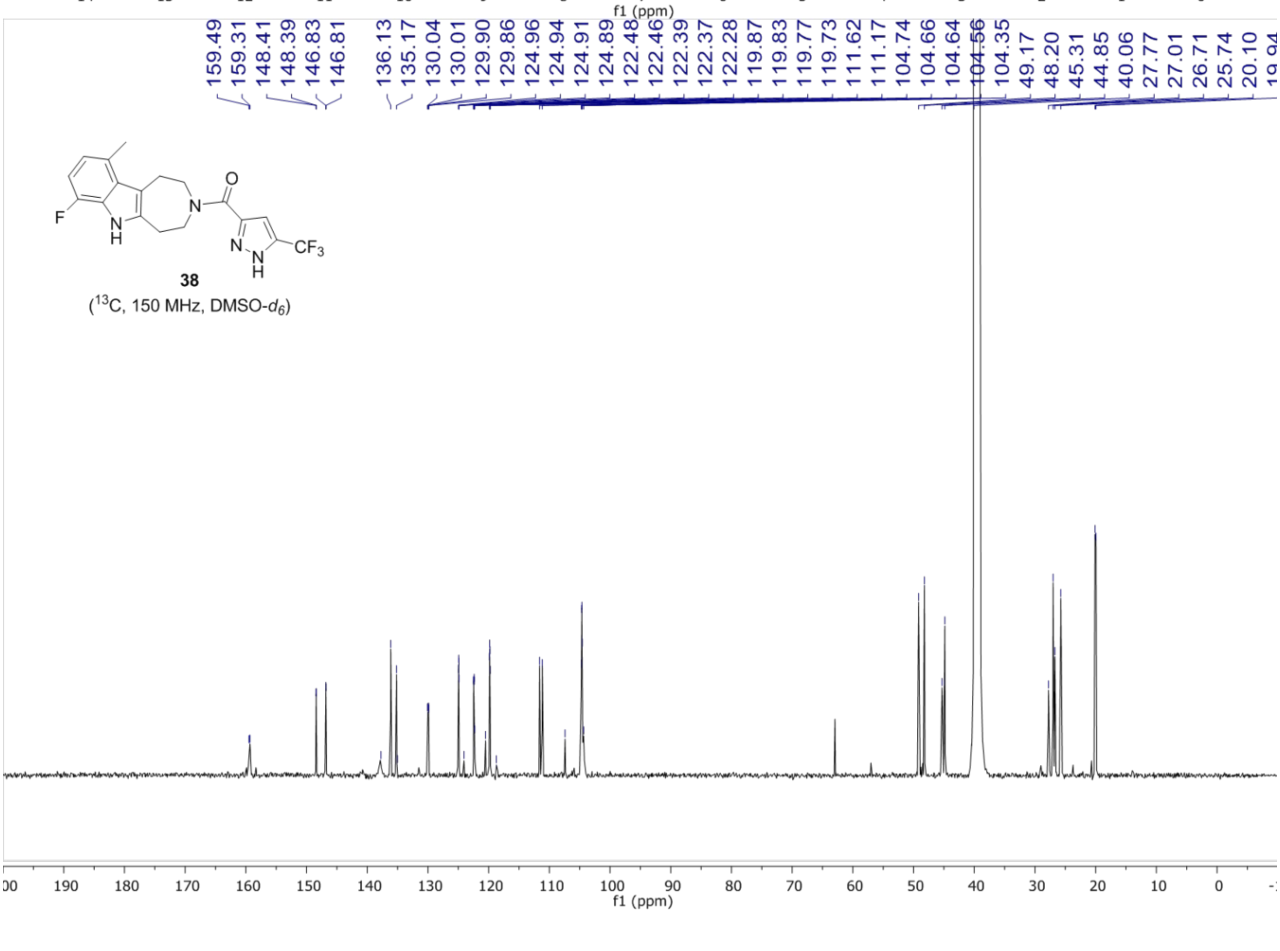
159.49
159.31
148.41
148.39
146.83
146.81
136.13
135.17
130.04
130.01
129.90
129.86
124.96
124.94
124.91
124.89
122.48
122.46
122.39
122.37
122.28
119.87
119.83
119.77
119.73
111.62
111.17
104.74
104.66
104.64
104.56
104.35
49.17
48.20
45.31
44.85
40.06
27.77
27.01
26.71
25.74
20.10
F
NH
N
O
N
N
H
CF3
38
(13C, 150 MHz, DMSO-d6)
f1 (ppm)

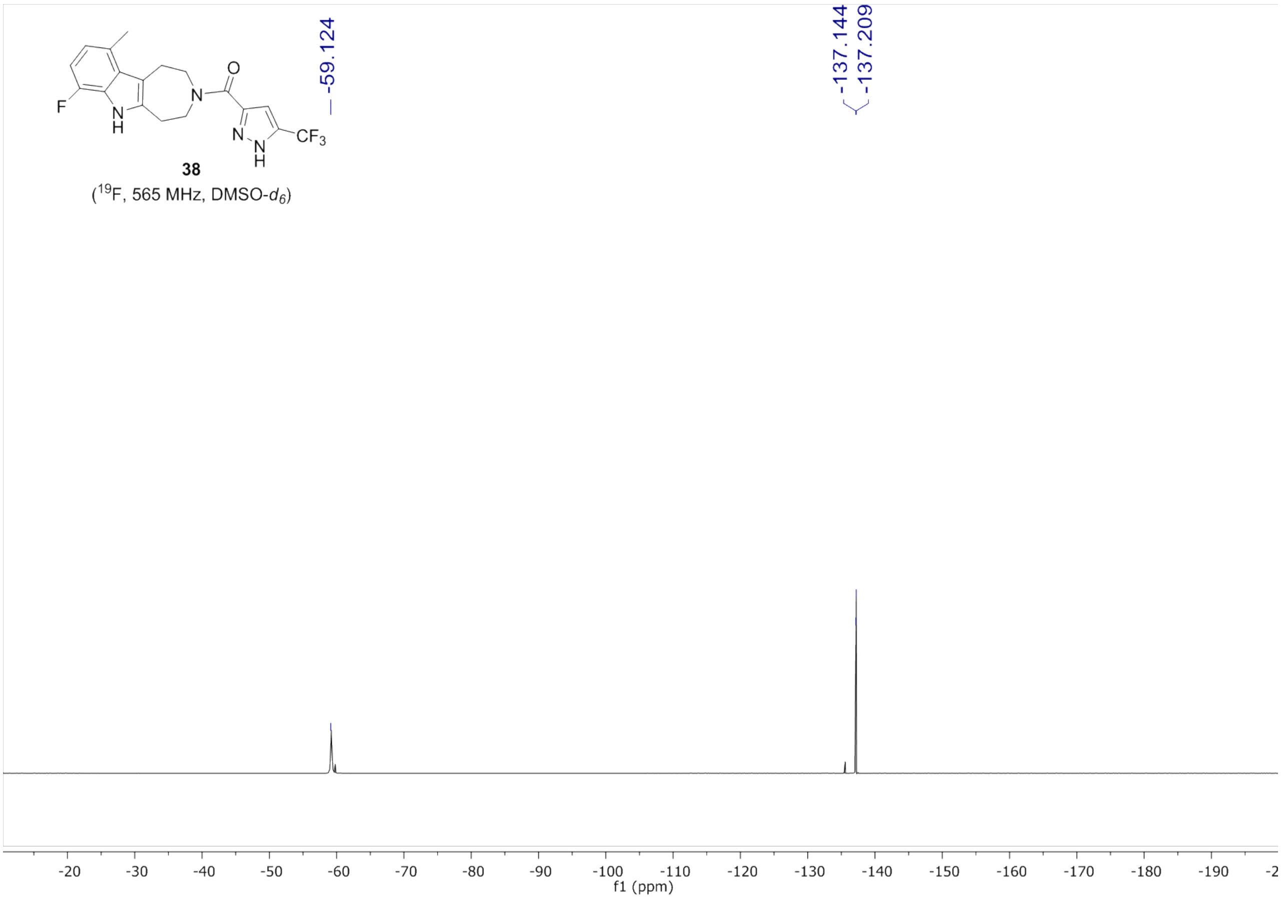

-59.124
-137.144
-137.209
F
H
N
O
N
N
H
CF3
38
(19F, 565 MHz, DMSO-d6)
-20 -30 -40 -50 -60 -70 -80 -90 -100 -110 -120 -130 -140 -150 -160 -170 -180 -190
f1 (ppm)

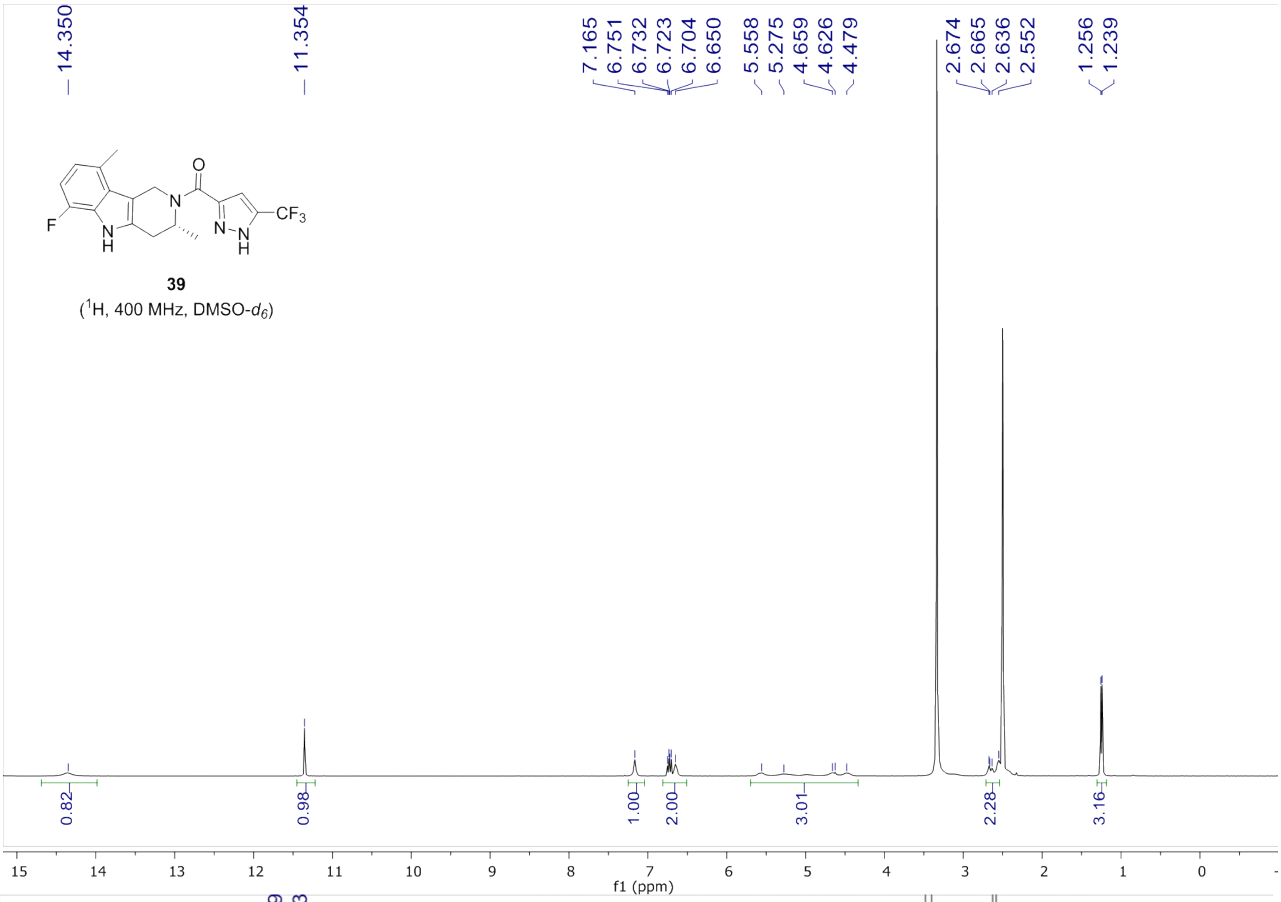

39
(^{1}H, 400 MHz, DMSO-d_6)

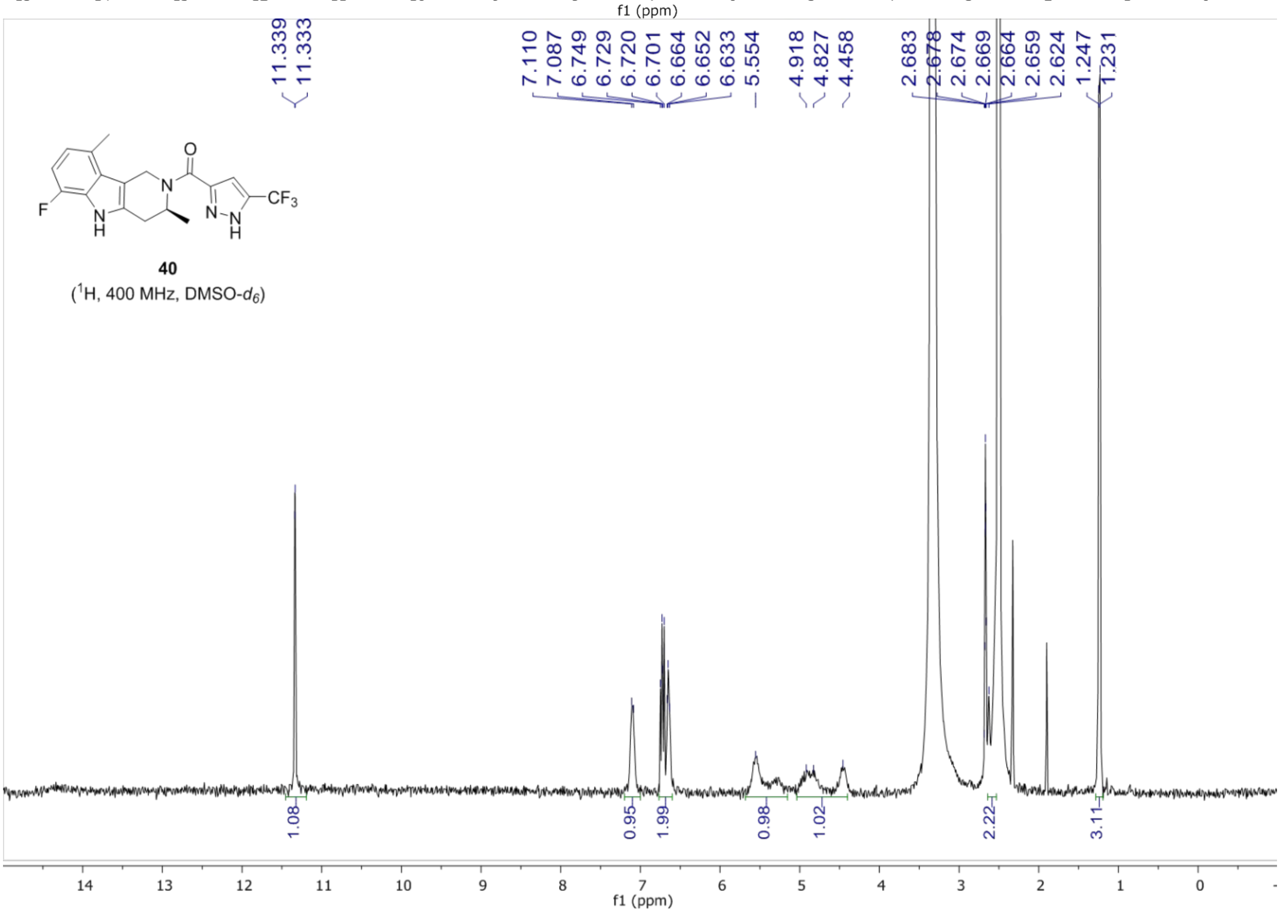

40
(^{1}H, 400 MHz, DMSO-d_6)

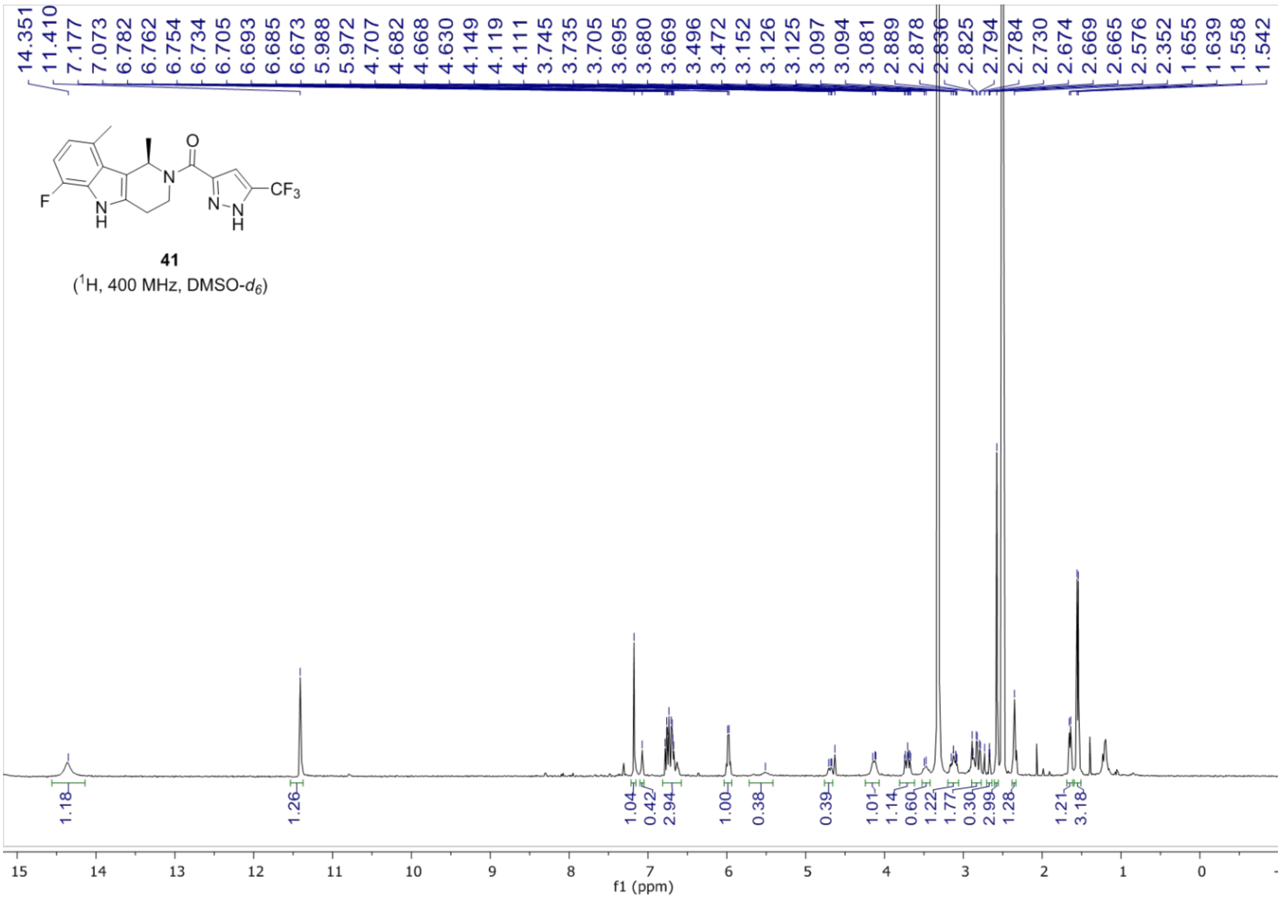
14.351 11.410 7.177 7.073 6.782 6.762 6.754 6.734 6.705 6.693 6.685 6.673 5.988 5.972 4.707 4.682 4.668 4.630 4.149 4.119 4.111 3.745 3.735 3.705 3.695 3.680 3.669 3.496 3.472 3.152 3.126 3.125 3.097 3.094 3.081 2.889 2.878 2.836 2.825 2.794 2.784 2.730 2.674 2.669 2.665 2.576 2.352 1.655 1.639 1.558 1.542
F
NH
N
O
N
N
H
CF3
41
(1H, 400 MHz, DMSO-d6)
1.18 1.26 1.04 0.42 2.94 1.00 0.38 0.39 1.01 1.14 0.60 1.22 1.77 0.30 2.99 1.28 1.21 3.18
15 14 13 12 11 10 9 8 7 6 5 4 3 2 1 0
f1 (ppm)

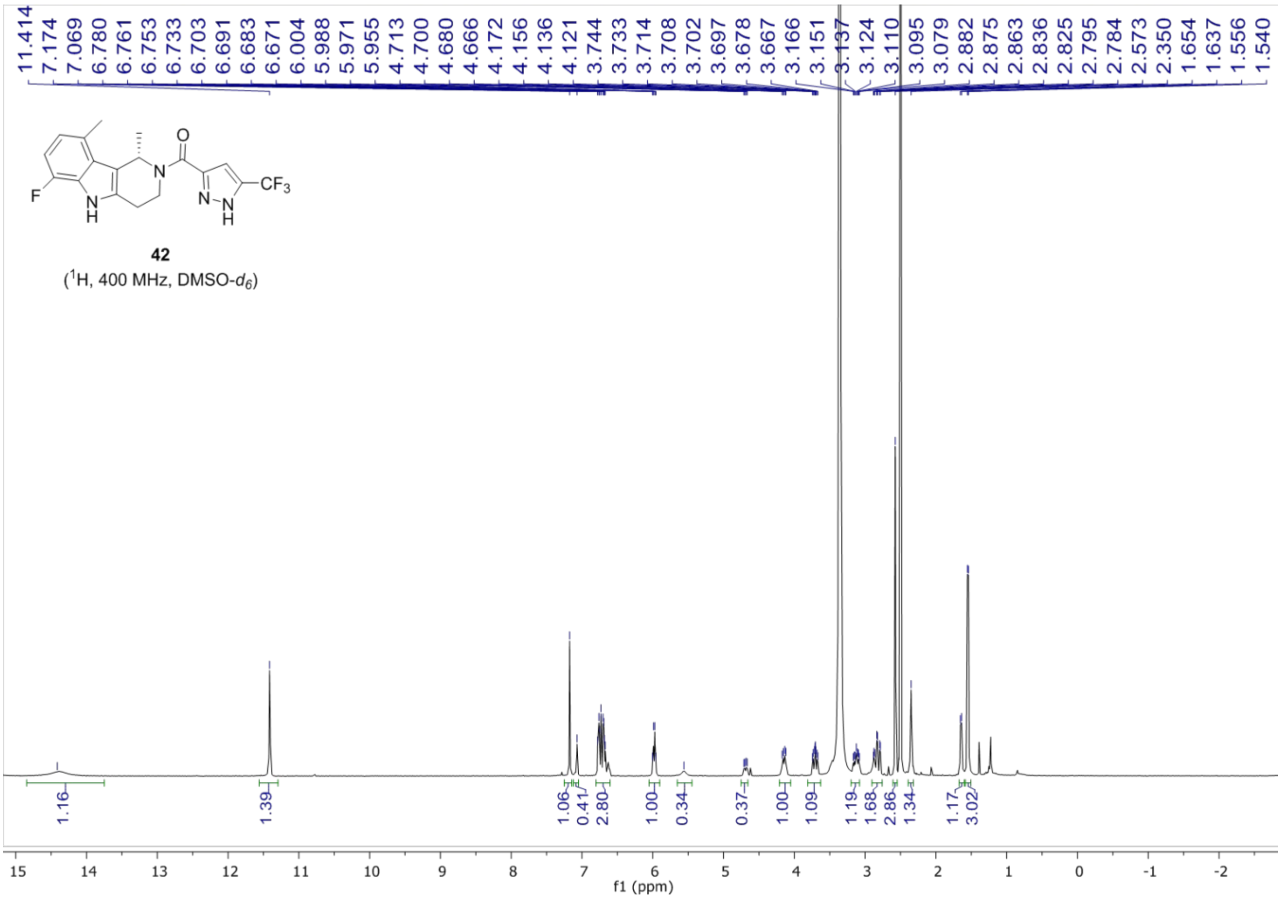
11.414 7.174 7.069 6.780 6.761 6.753 6.733 6.703 6.691 6.683 6.671 6.004 5.988 5.971 5.955 4.713 4.700 4.680 4.666 4.172 4.156 4.136 4.121 3.744 3.733 3.714 3.708 3.702 3.697 3.678 3.667 3.166 3.151 3.137 3.124 3.110 3.095 3.079 2.882 2.875 2.863 2.836 2.825 2.795 2.784 2.573 2.350 1.654 1.637 1.556 1.540
F
NH
N
O
N
N
H
CF3
42
(1H, 400 MHz, DMSO-d6)
1.16 1.39 1.06 0.41 2.80 1.00 0.34 0.37 1.00 1.09 1.19 1.68 2.86 1.34 1.17 3.02
15 14 13 12 11 10 9 8 7 6 5 4 3 2 1 0 -1 -2
f1 (ppm)

2. LC/MS analyses of Hit 1-6 and final compounds 1-42

10mM stock solution of test compound was prepared in DMSO-d_6 and further diluted 20-fold with CH_3CN-H_2O (1:1) for analysis. The QC analyses were performed on a Waters ACQUITY UPLC/MS system consisting of a SQD (Single Quadrupole Detector) Mass Spectrometer equipped with an Electrospray Ionization interface and a Photodiode Array Detector. Electrospray ionization in positive and negative mode was applied in the mass scan range 100-500Da. The PDA range was 210-400nm. The analyses were run on an ACQUITY UPLC BEH C_{18} column (100x2.1mmID, particle size 1.7μm) with a VanGuard BEH C_{18} pre-column (5x2.1mmID, particle size 1.7μm). The mobile phase was 10mM NH_4OAc in H_2O at pH 5 adjusted with AcOH (A) and 10mM NH_4OAc in CH_3CN-H_2O (95:5) at pH 5 (B) with 0.5mL/min as flow rate. A linear gradient was applied: 0-0.2min: 10%B, 0.2-6.2min: 10-90%B, 6.2-6.3min: 90-100%, 6.3-7.0min: 100%B. All initial hits and final compounds displayed ≥95% purity as determined by UPLC/MS analysis (unless otherwise indicated).

Hit-1

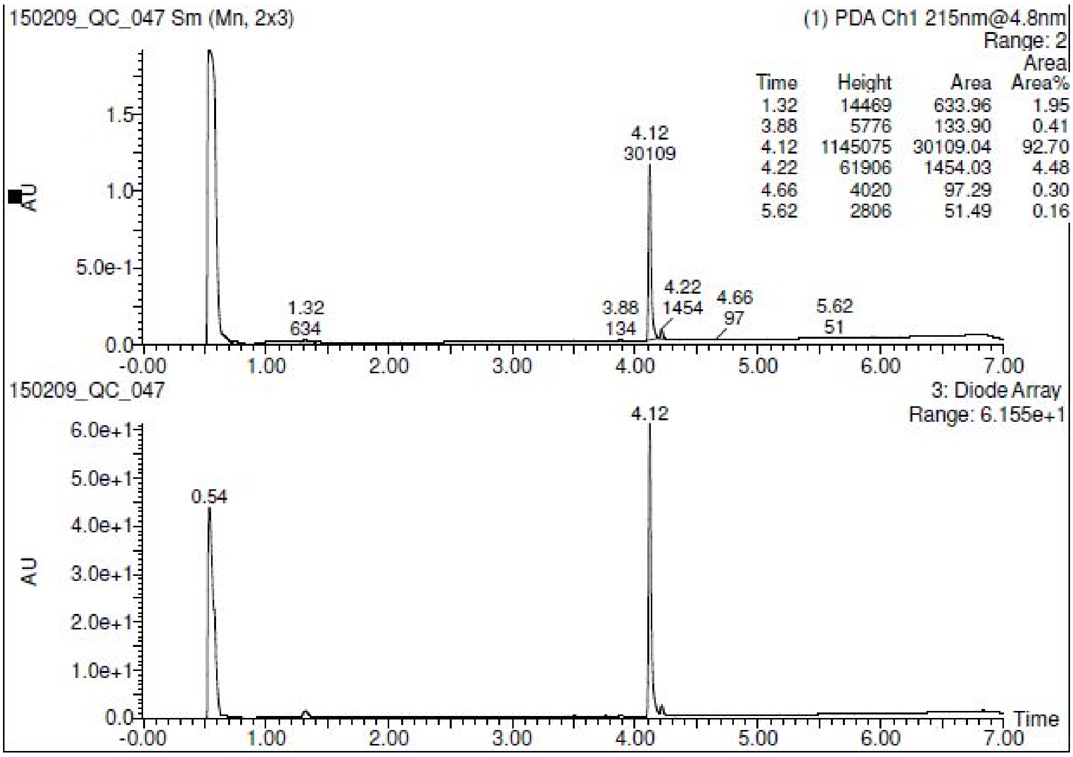

Hit-2

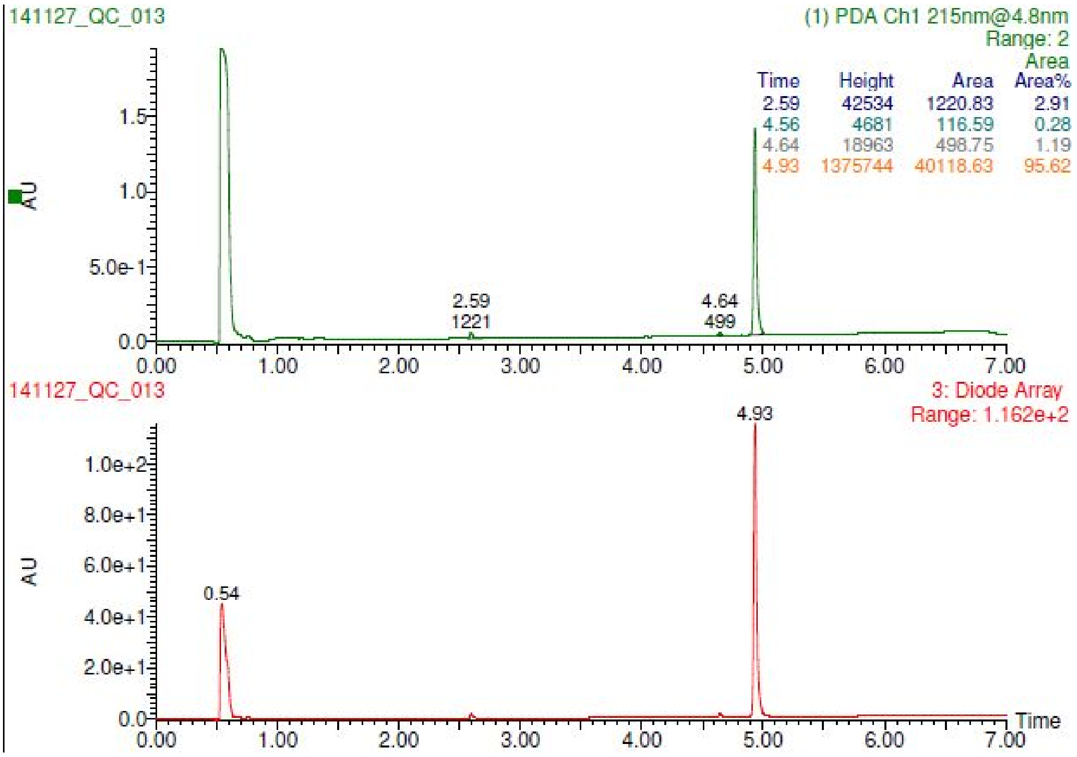

141127_QC_013
(1) PDA Ch1 215nm@4.8nm
Range: 2
Time Height Area Area%
2.59 42534 1220.83 2.91
4.56 4681 116.59 0.28
4.64 18963 498.75 1.19
4.93 1375744 40118.63 95.62
AU
2.59
1221
4.64
499
141127_QC_013
3: Diode Array
Range: 1.162e+2
4.93
0.54
Time

Hit-3

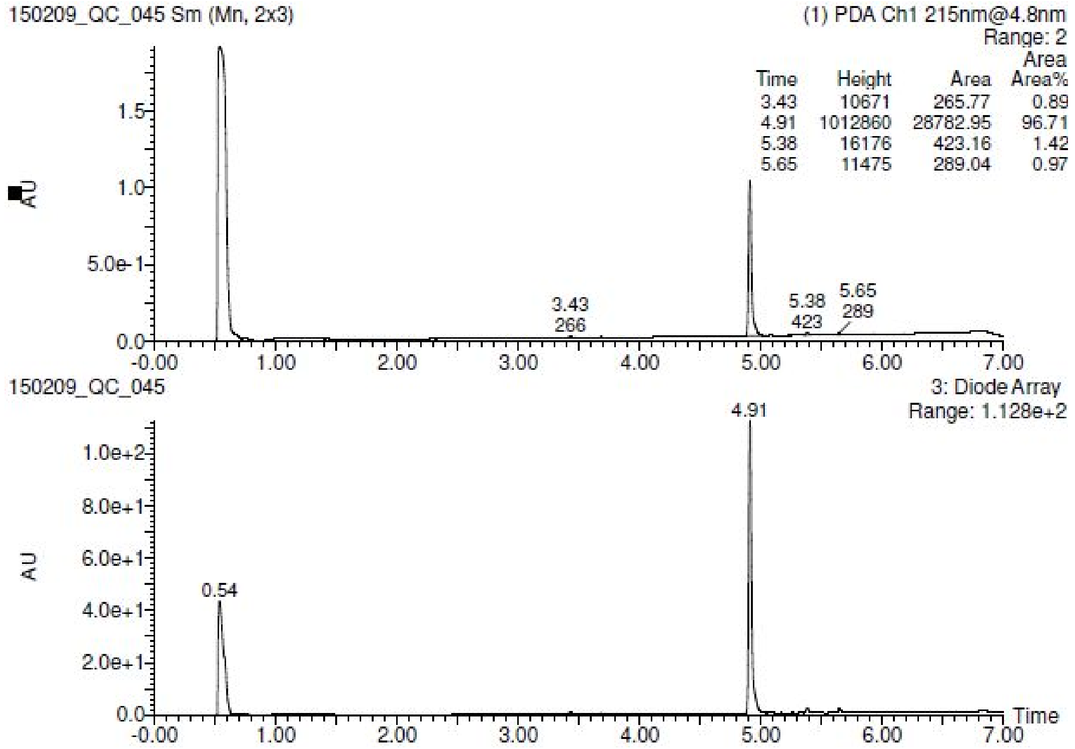

150209_QC_045 Sm (Mn, 2x3)
(1) PDA Ch1 215nm@4.8nm
Range: 2
Time Height Area Area%
3.43 10671 265.77 0.89
4.91 1012860 28782.95 96.71
5.38 16176 423.16 1.42
5.65 11475 289.04 0.97
AU
3.43
266
5.38
423
5.65
289
150209_QC_045
3: Diode Array
Range: 1.128e+2
4.91
0.54
Time

Hit-4

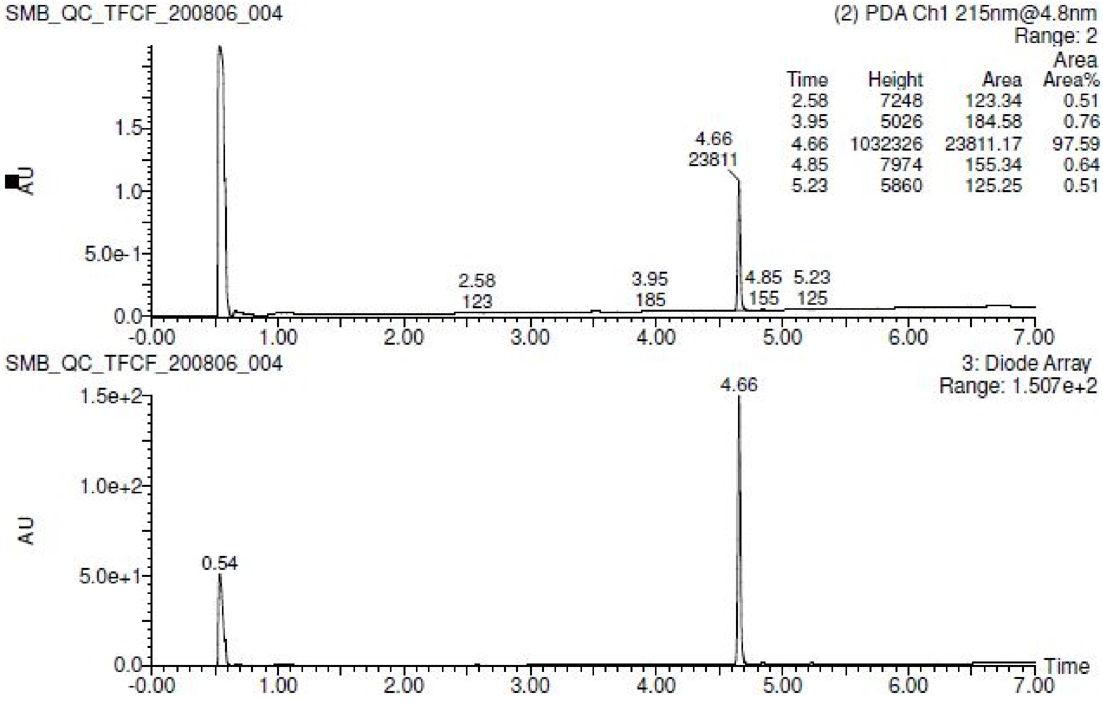
SMB_QC_TFCF_200806_004
(2) PDA Ch1 215nm@4.8nm
Range: 2
Time Height Area Area%
2.58 7248 123.34 0.51
3.95 5026 184.58 0.76
4.66 1032326 23811.17 97.59
4.85 7974 155.34 0.64
5.23 5860 125.25 0.51
AU
SMB_QC_TFCF_200806_004
3: Diode Array
Range: 1.507e+2
AU
Time

Hit-5

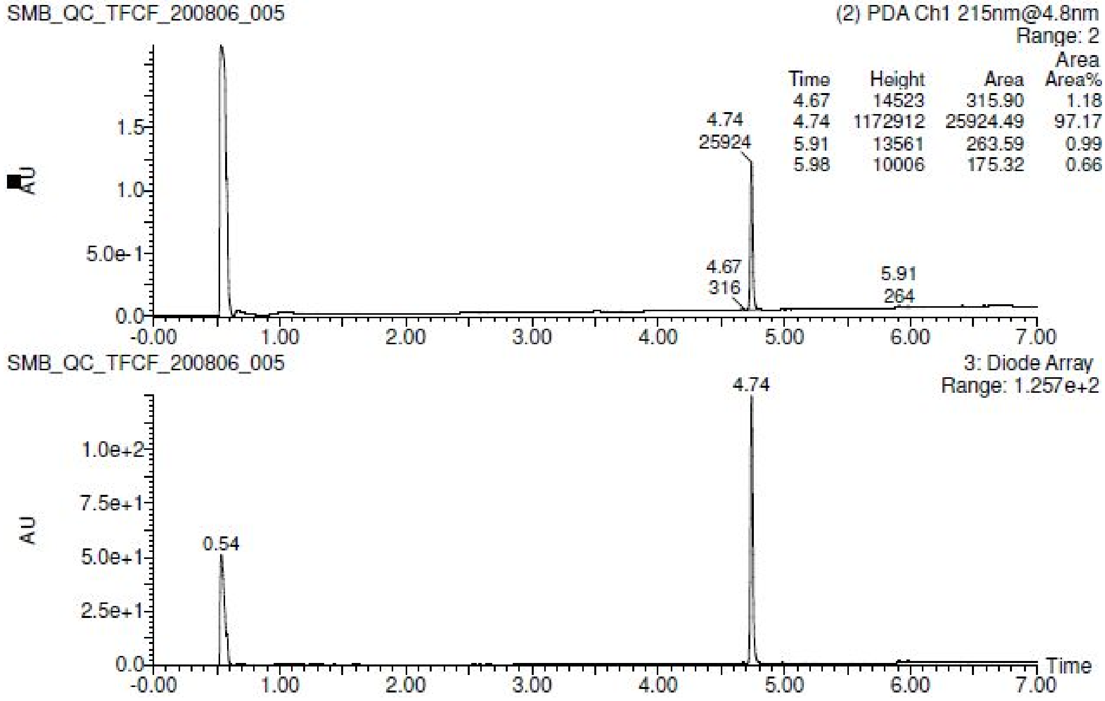
SMB_QC_TFCF_200806_005
(2) PDA Ch1 215nm@4.8nm
Range: 2
Time Height Area Area%
4.67 14523 315.90 1.18
4.74 1172912 25924.49 97.17
5.91 13561 263.59 0.99
5.98 10006 175.32 0.66
AU
SMB_QC_TFCF_200806_005
3: Diode Array
Range: 1.257e+2
AU
Time

Hit-6

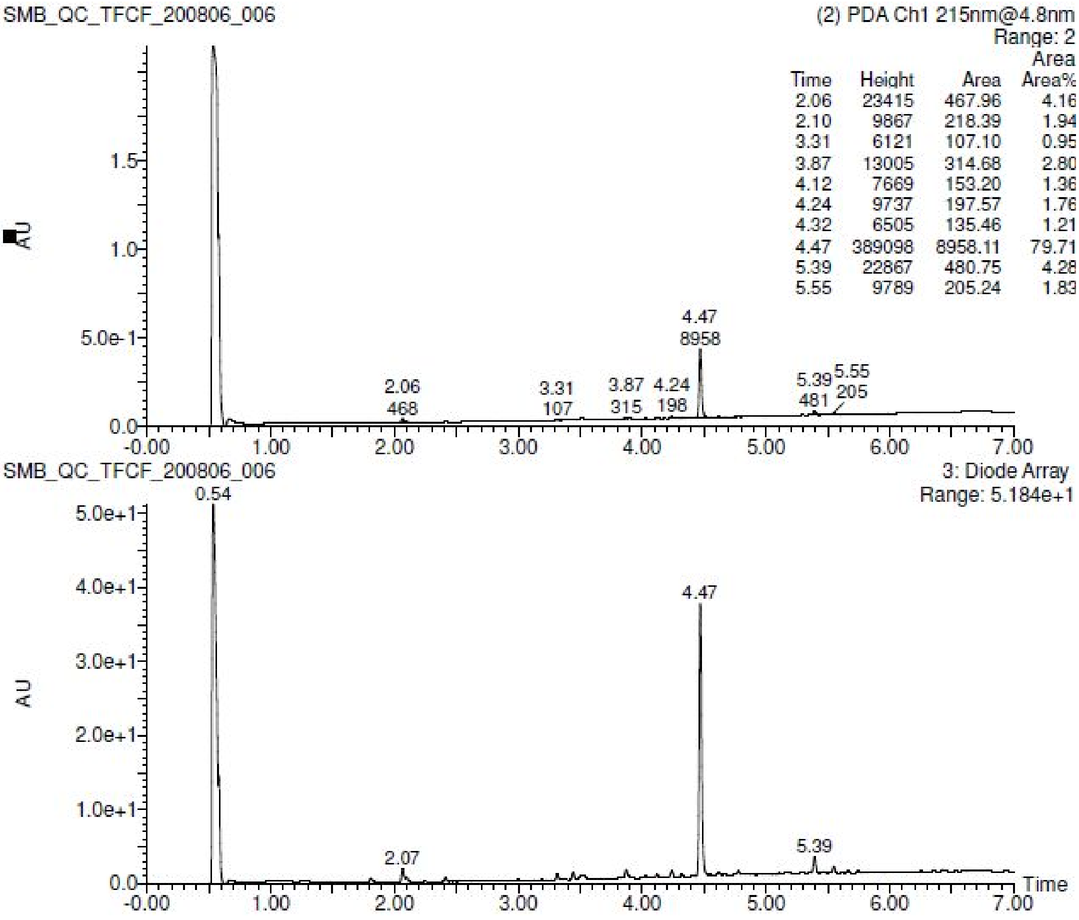

Compound **1**

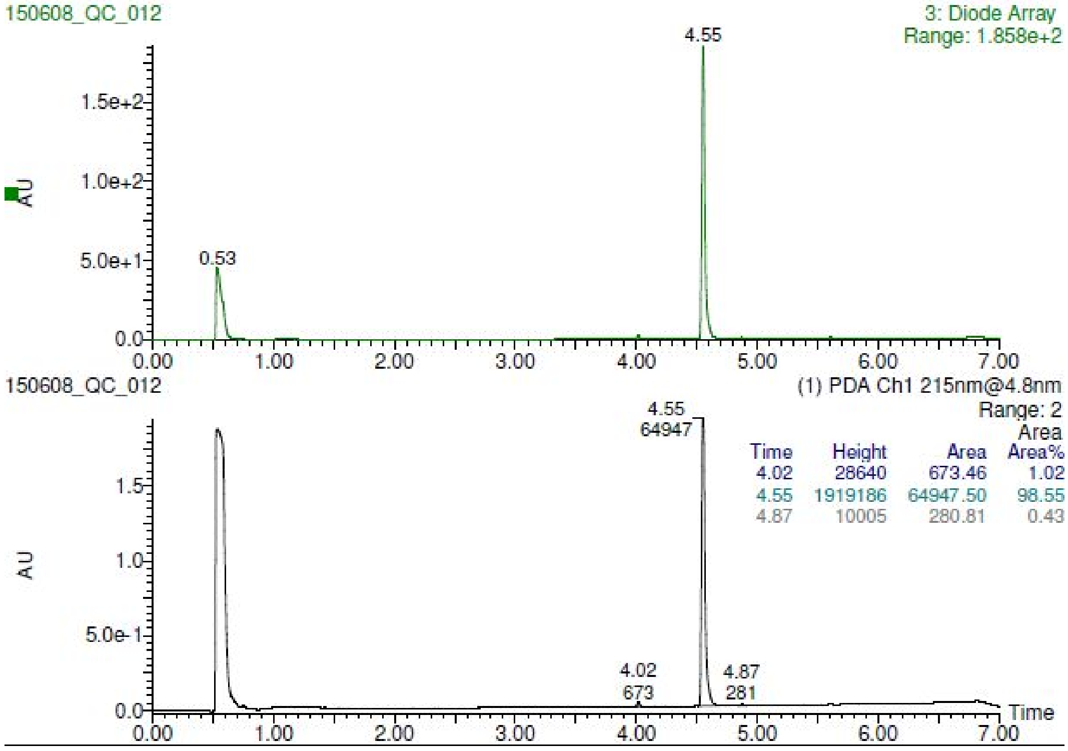

Compound **2**

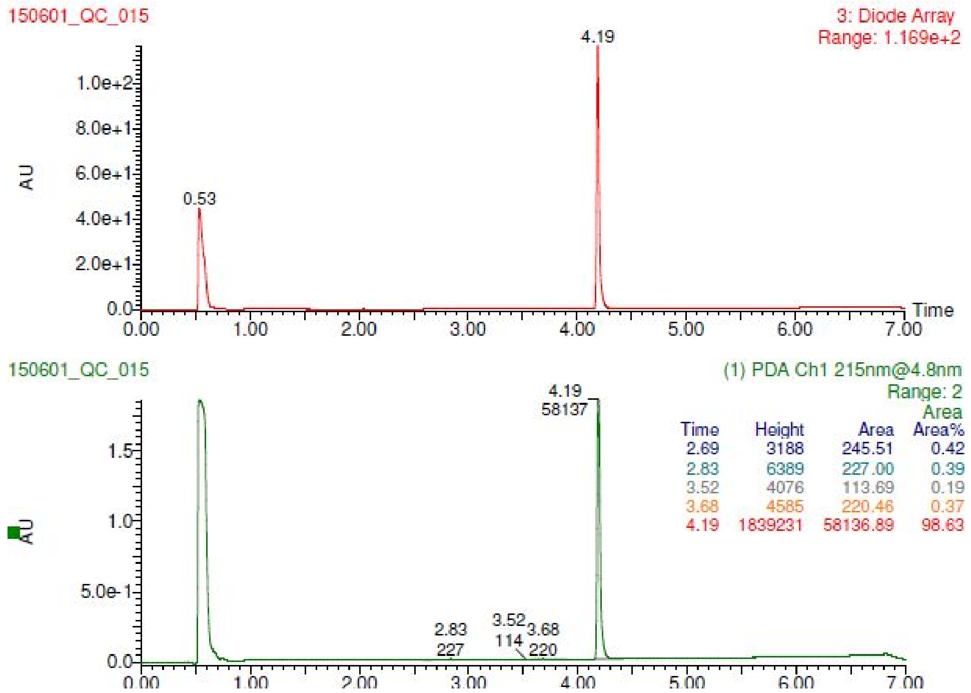

Compound **3**

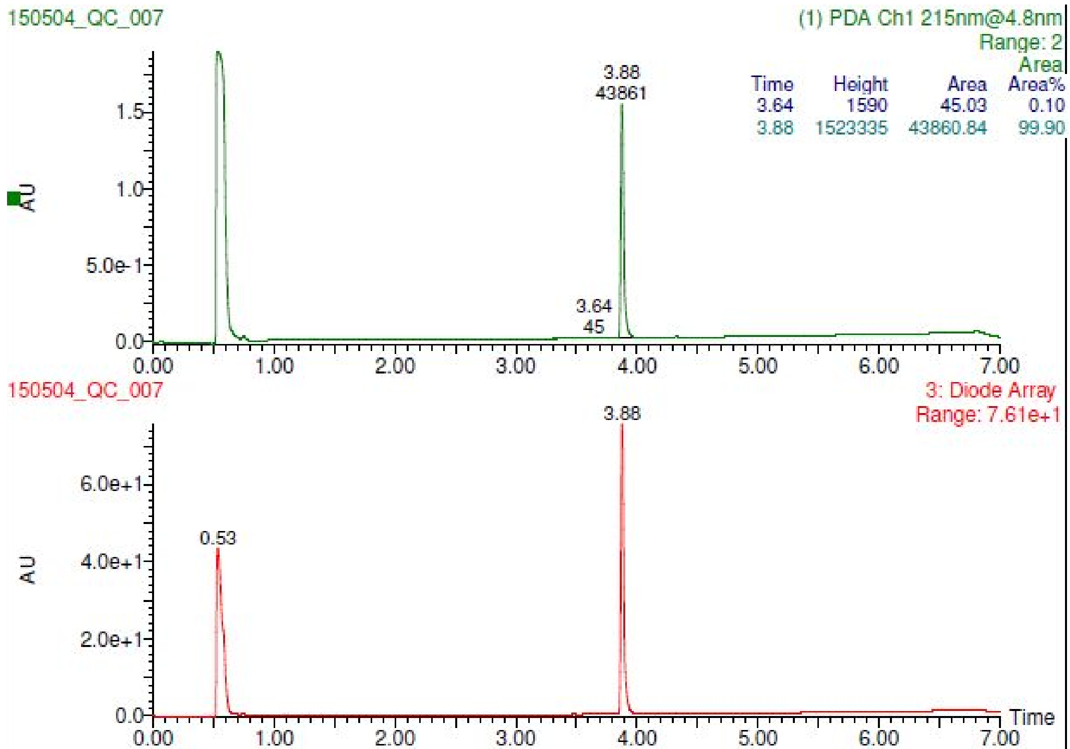

Compound **4**

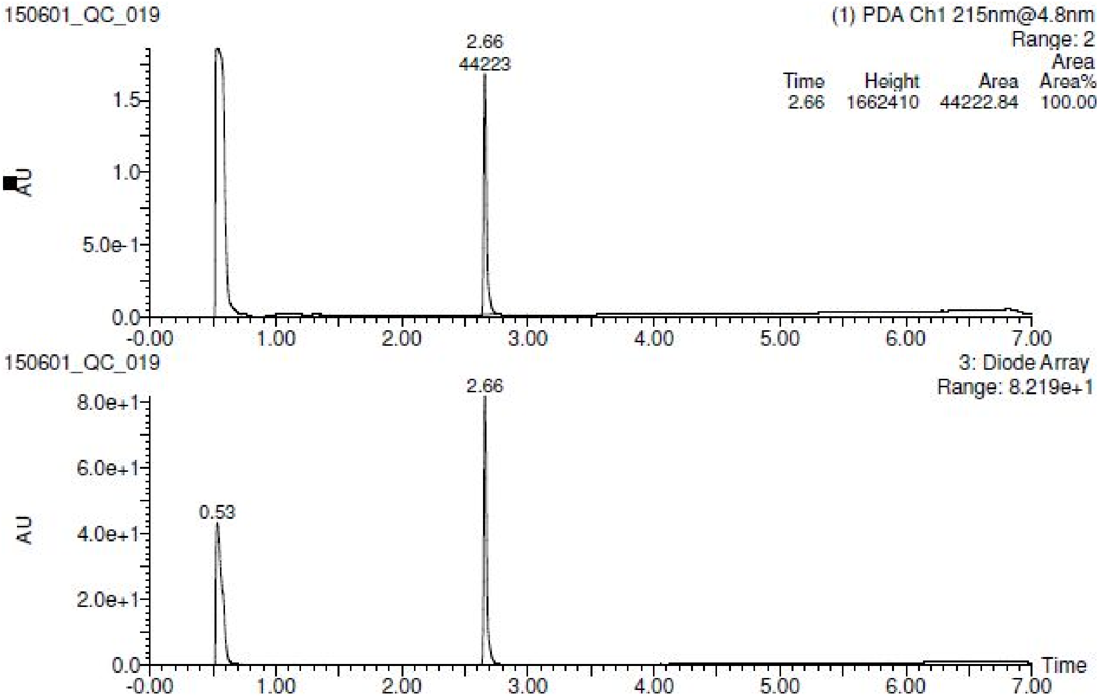

Compound **5**

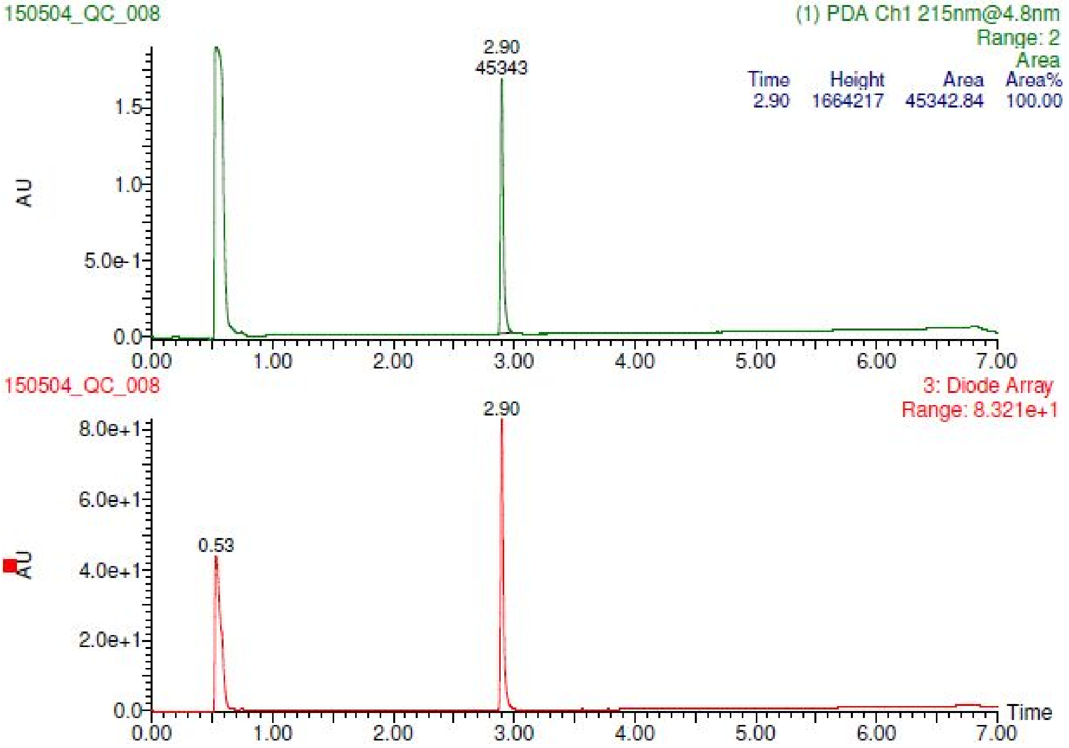

Compound **6**

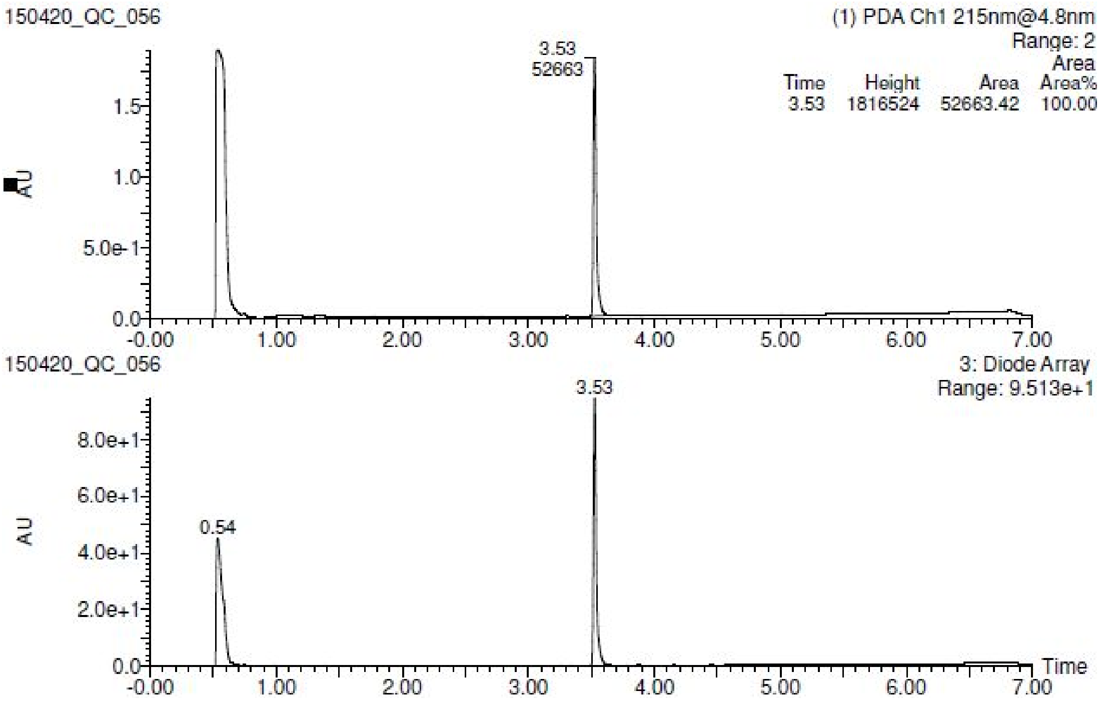

Compound **7**

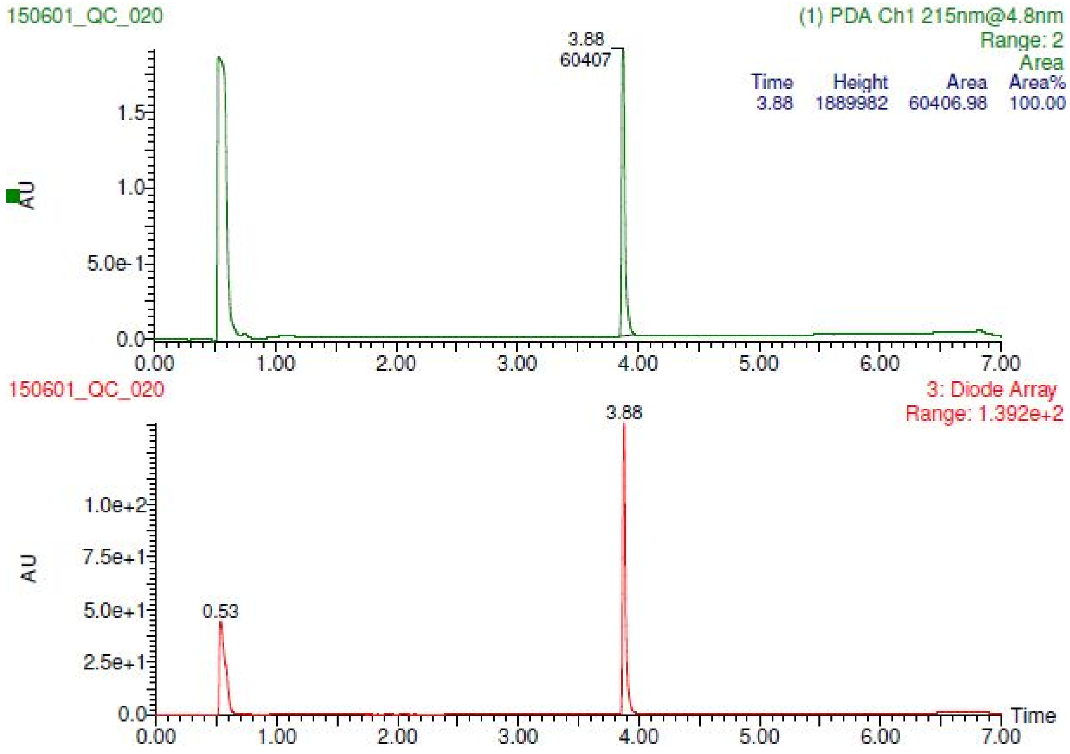

Compound **8**

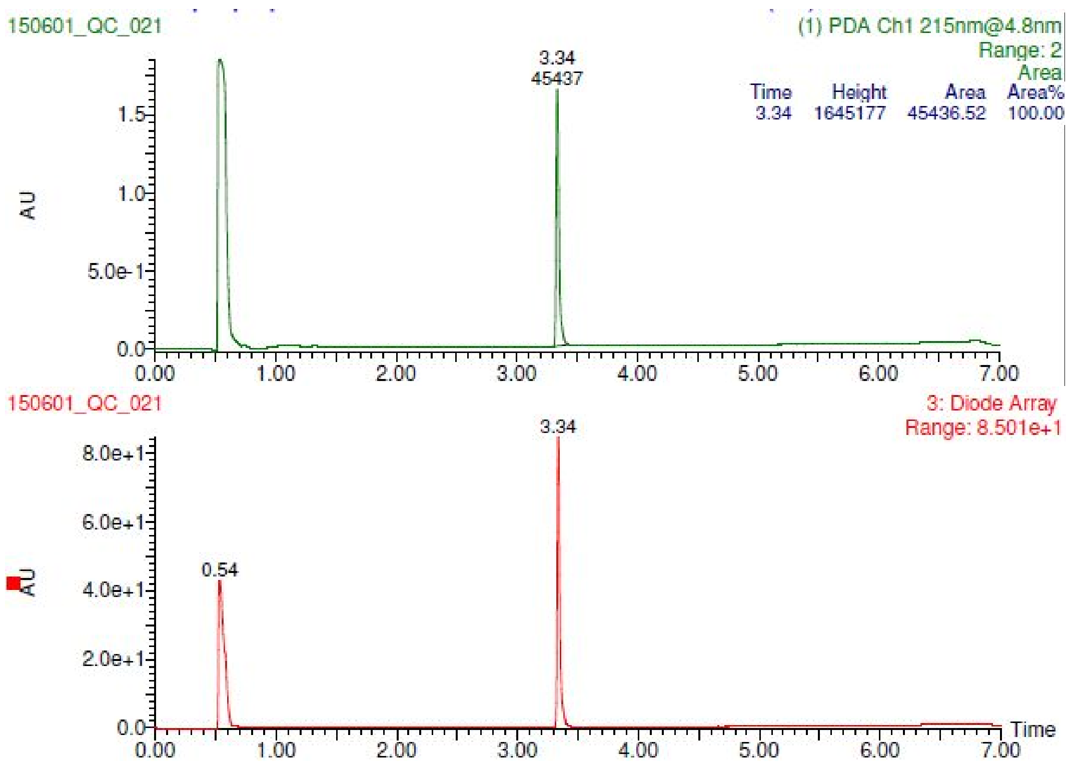

Compound **9**

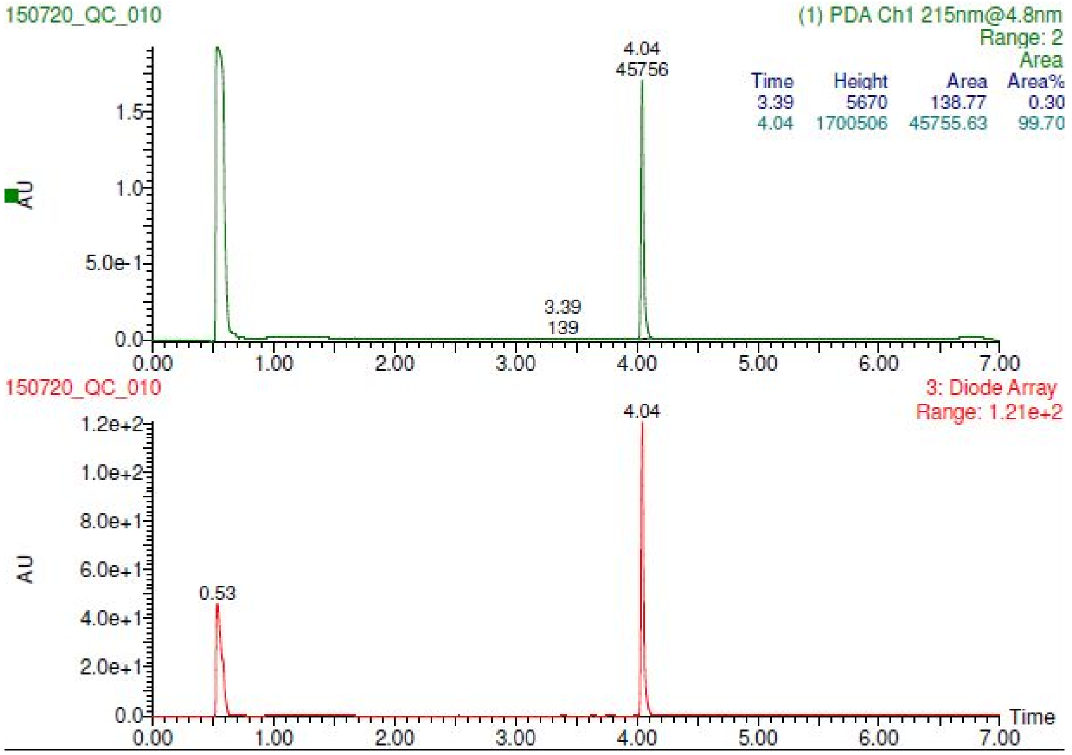

Compound **10**

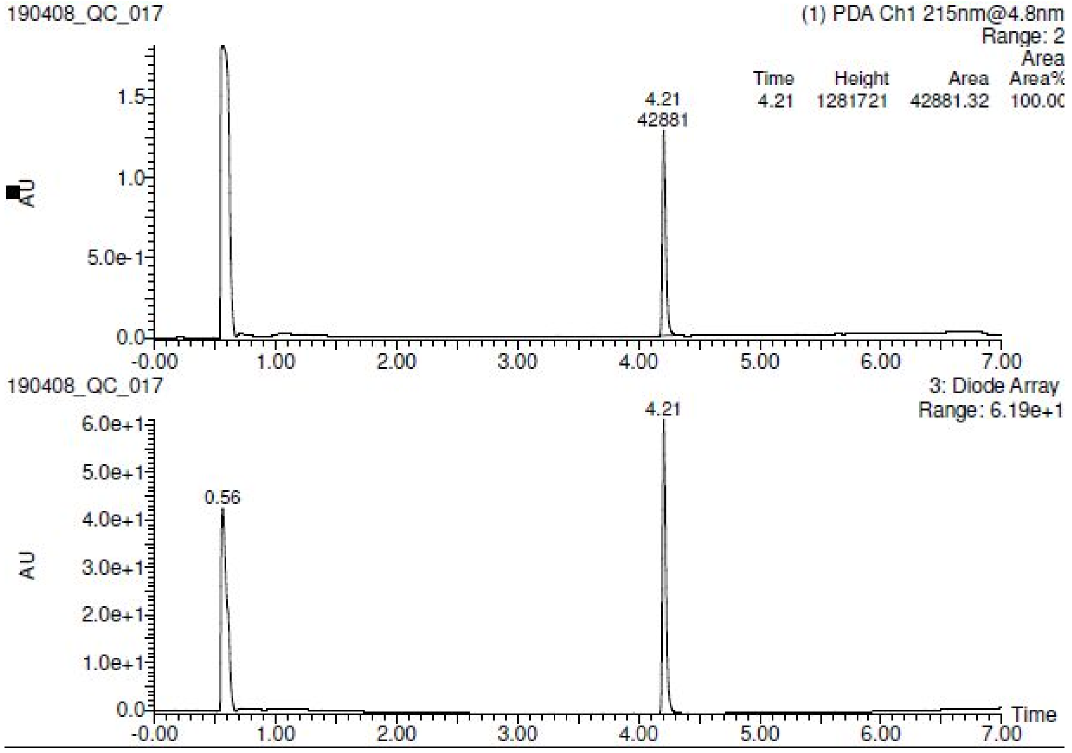

Compound **11**

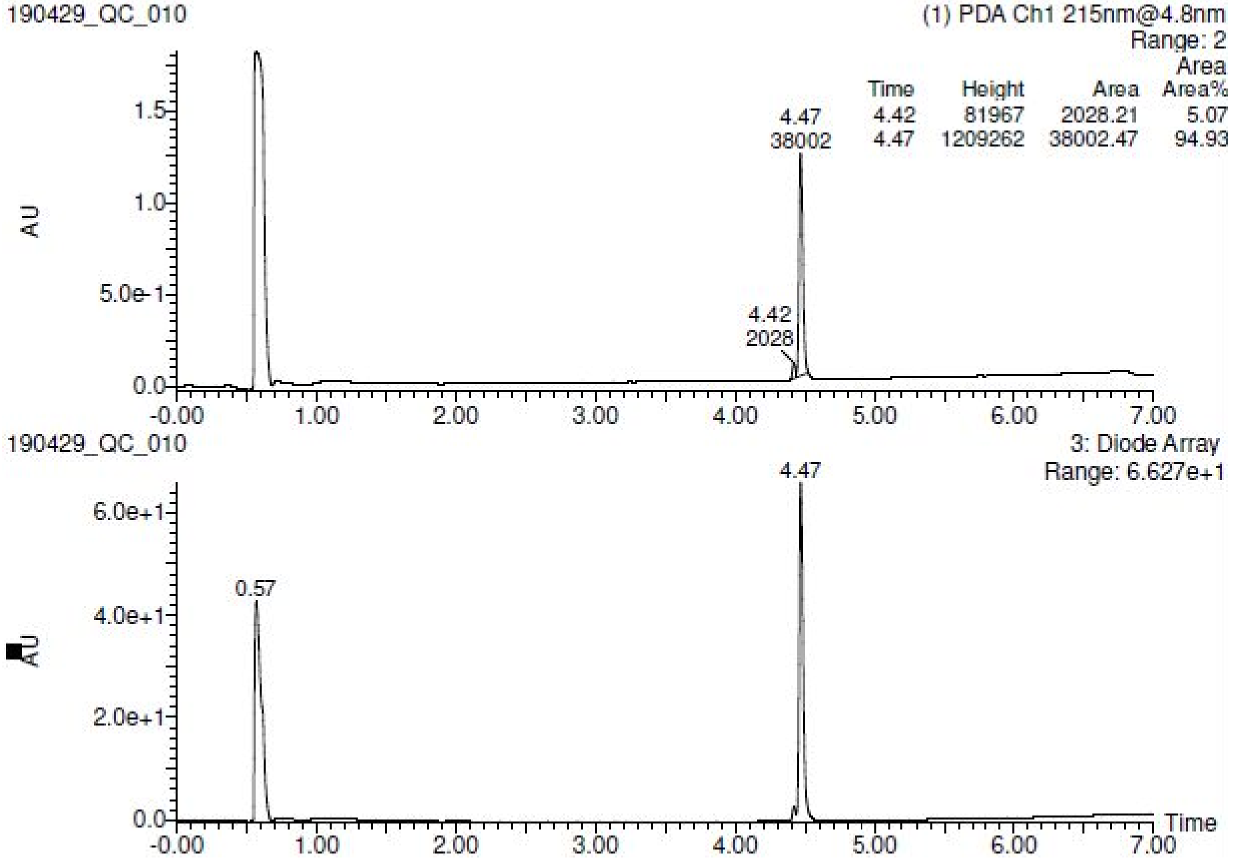

Compound **12**

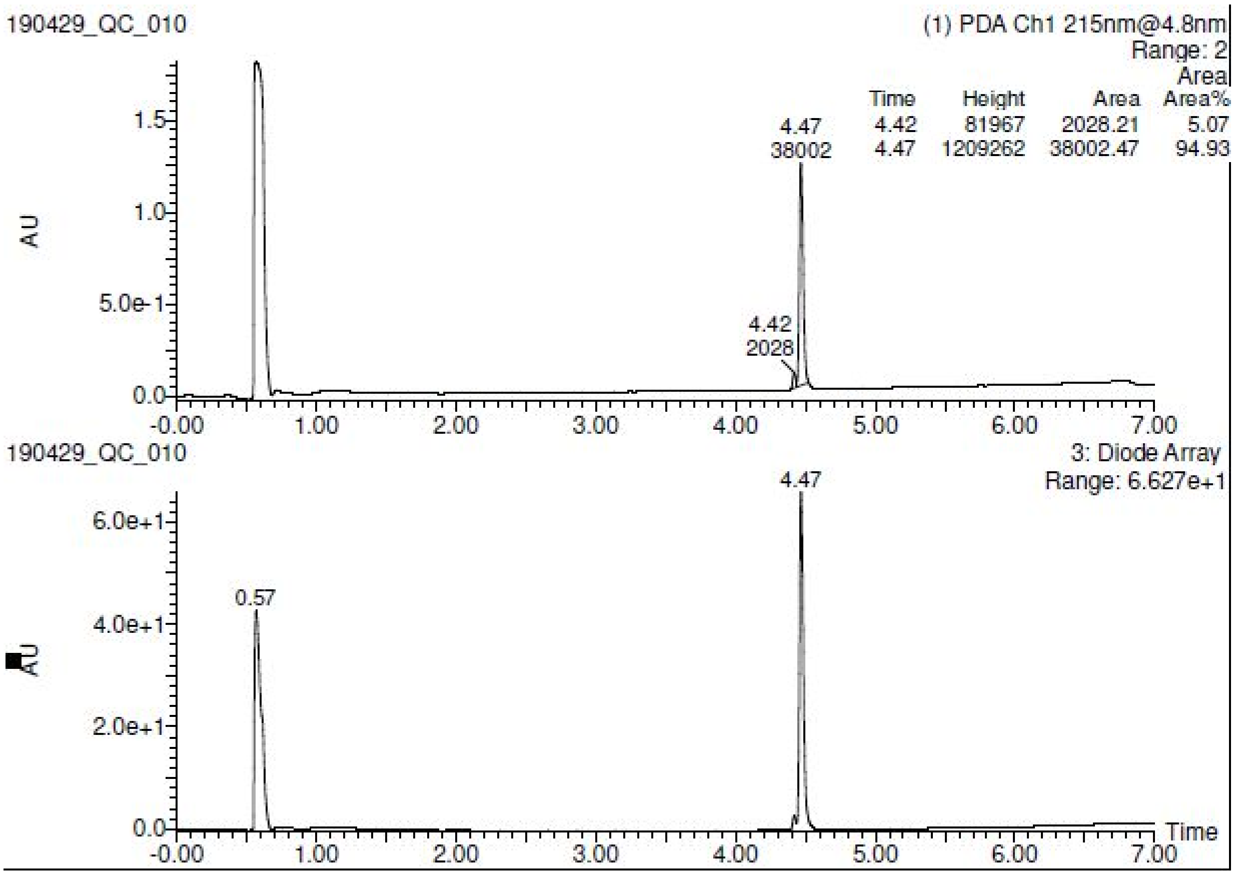

Compound **13**

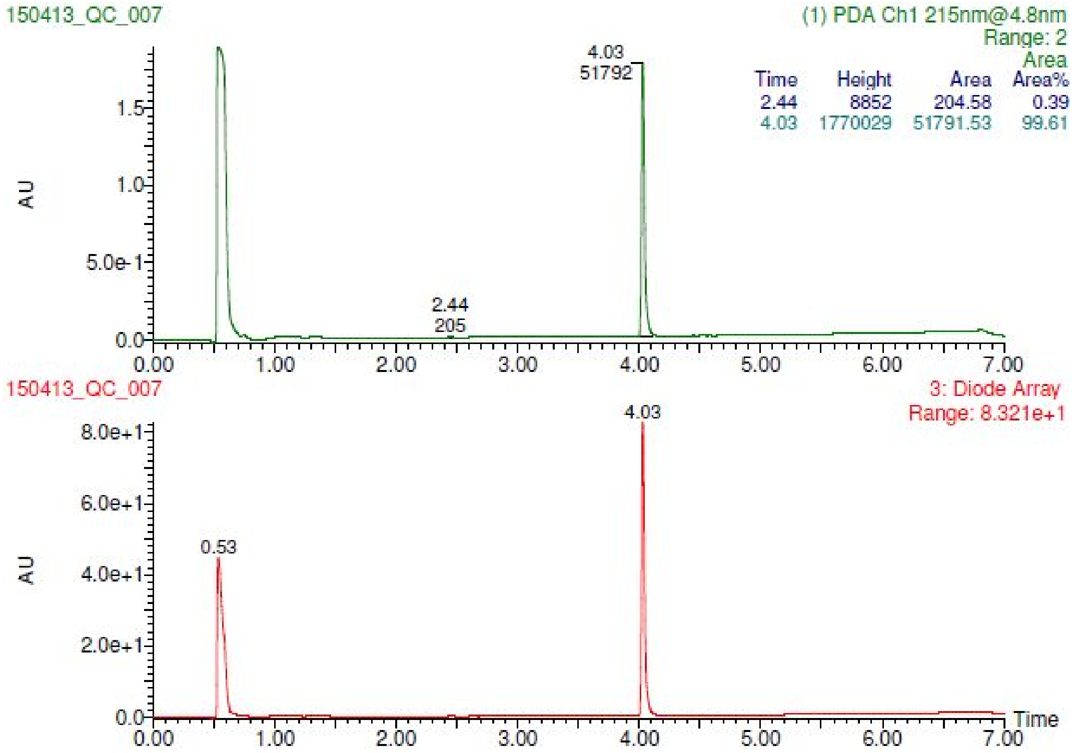

Compound **14**

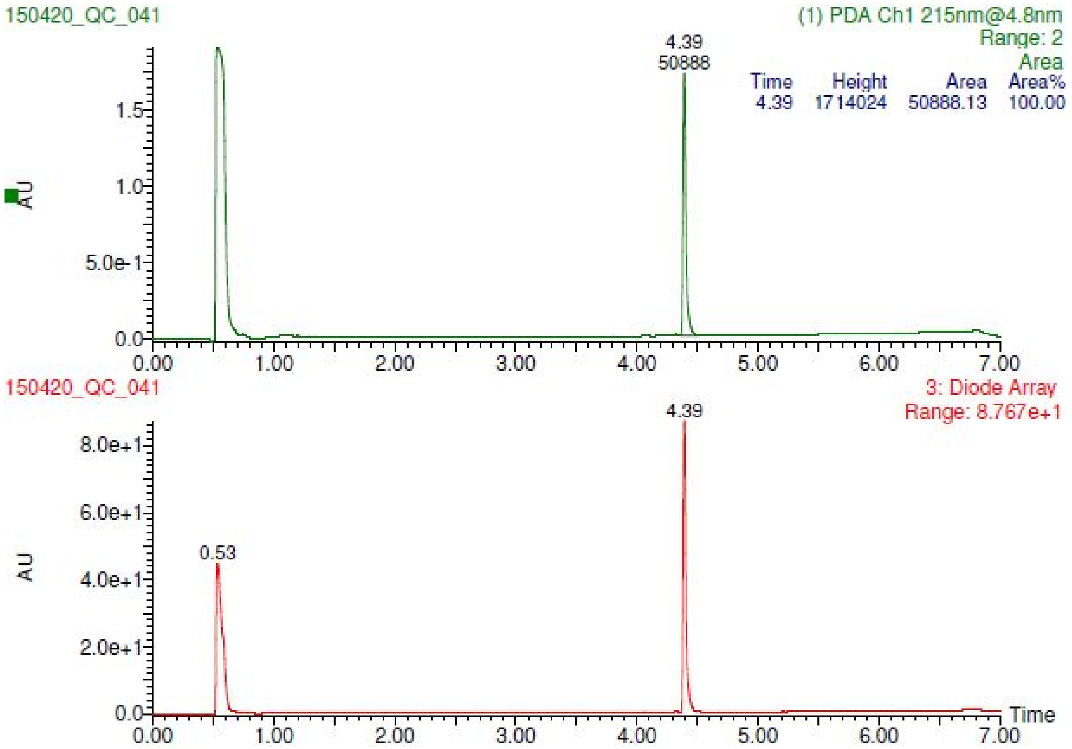

Compound **15**

Final compound **15** displayed ca. 93% purity as determined by UPLC/MS analysis.

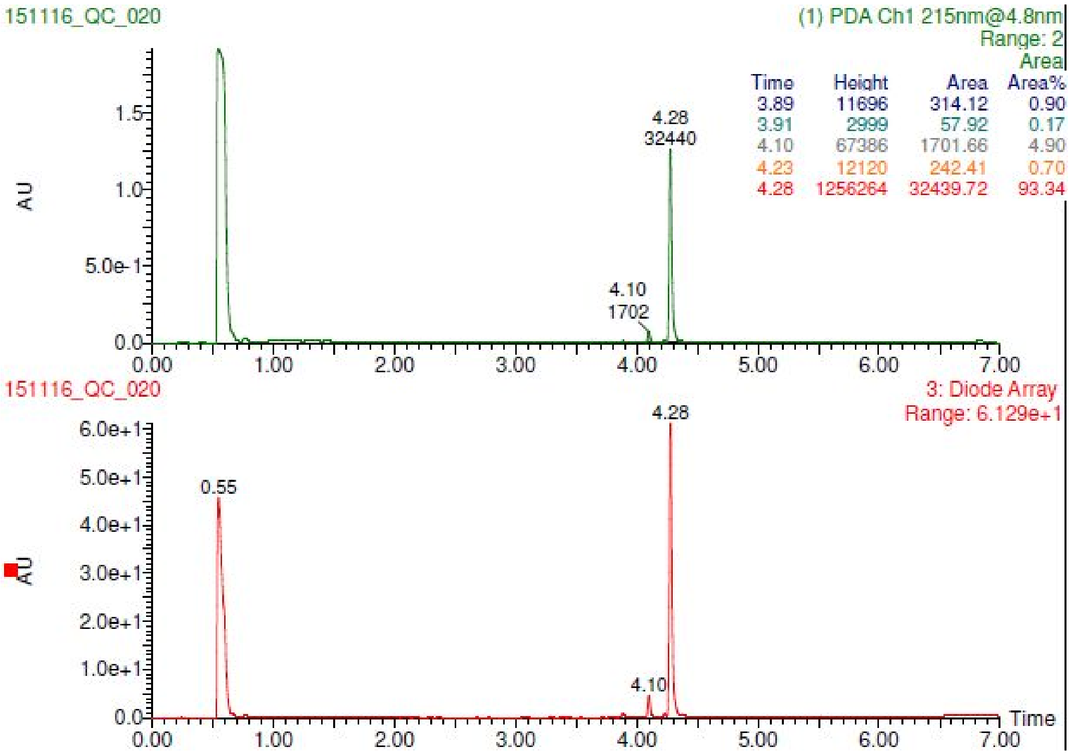

Compound **16**

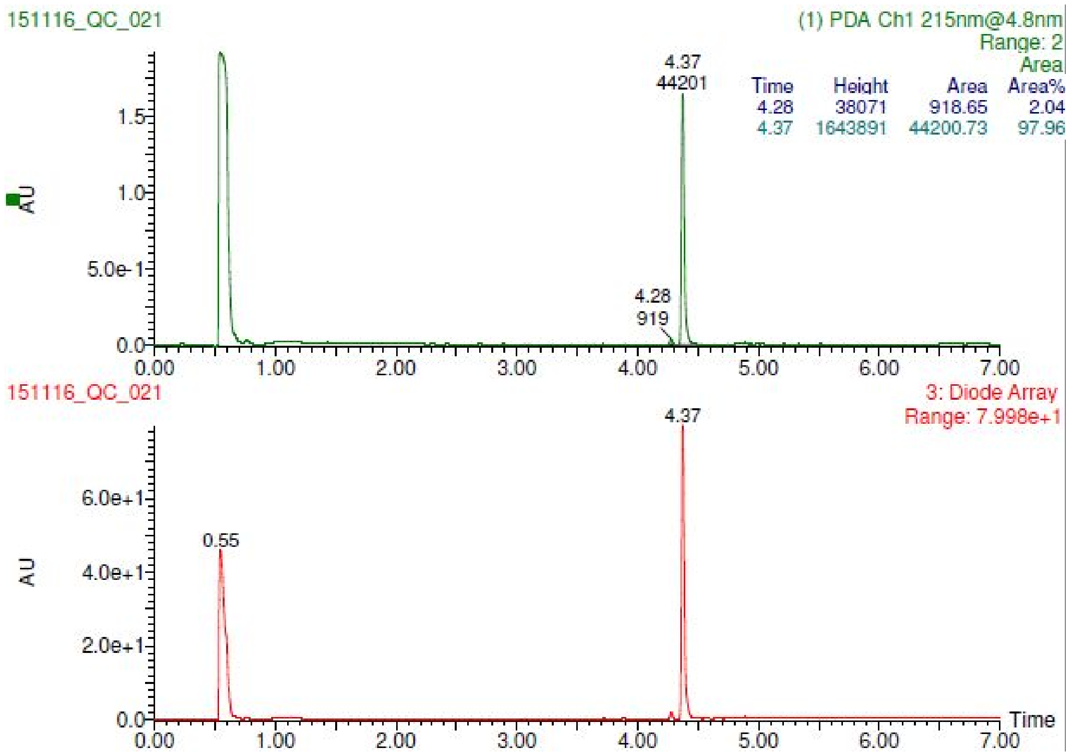

Compound **17**

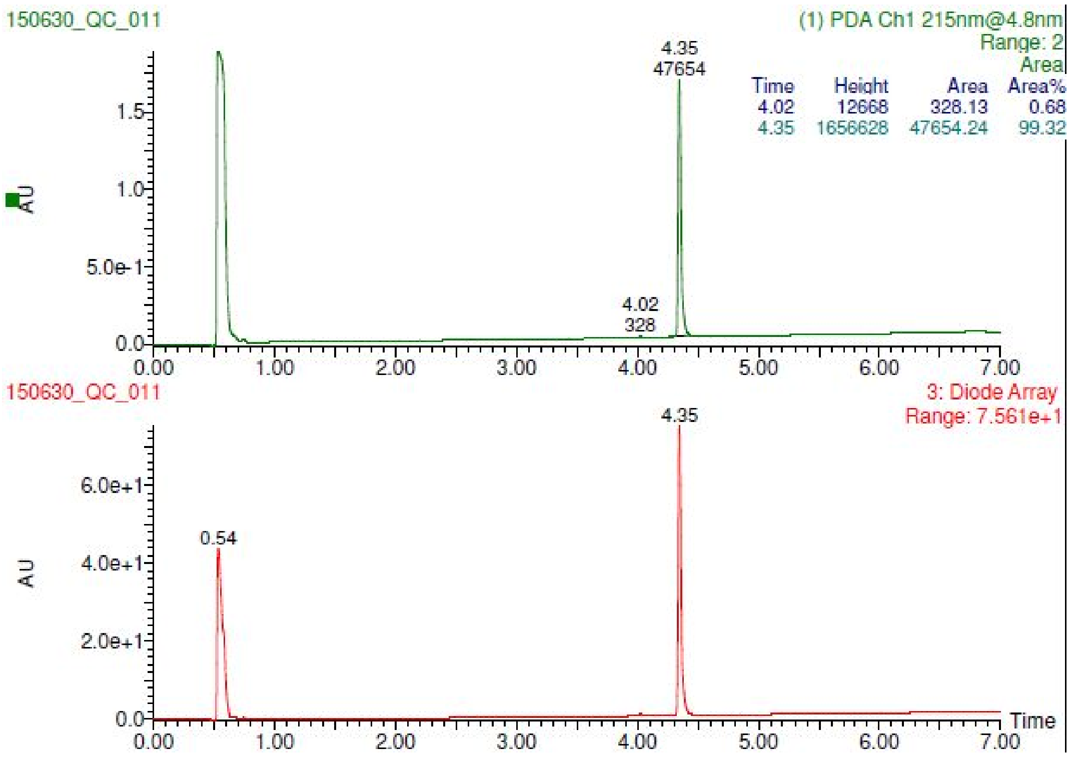

Compound **18**

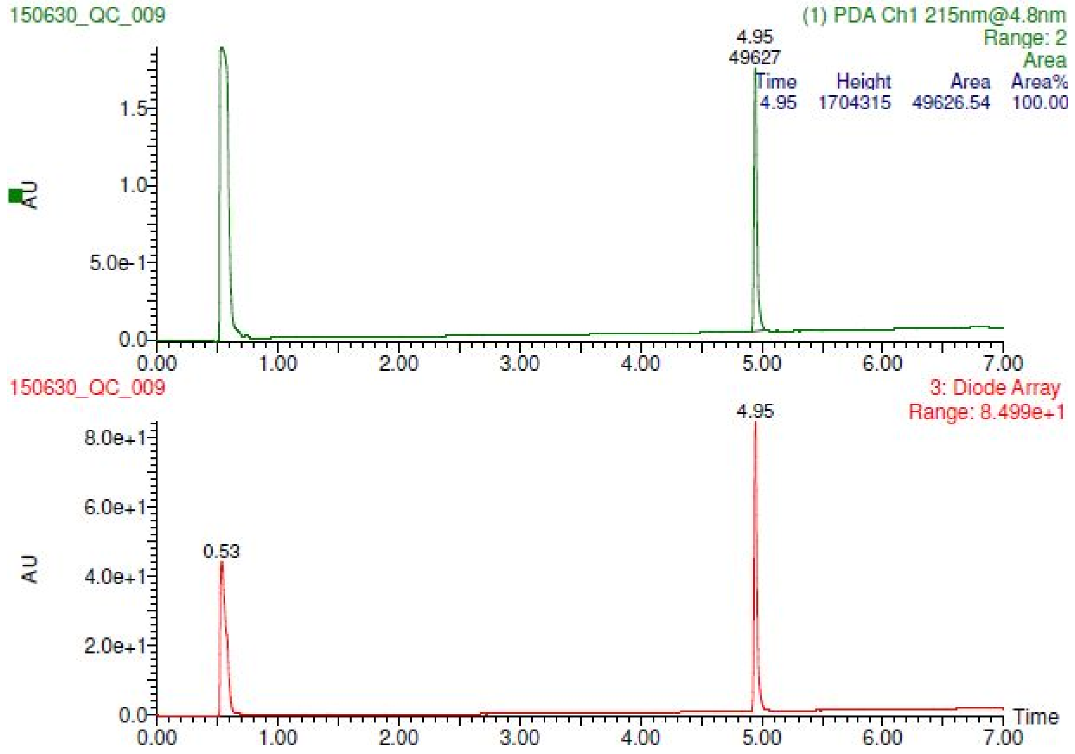

Compound **19**

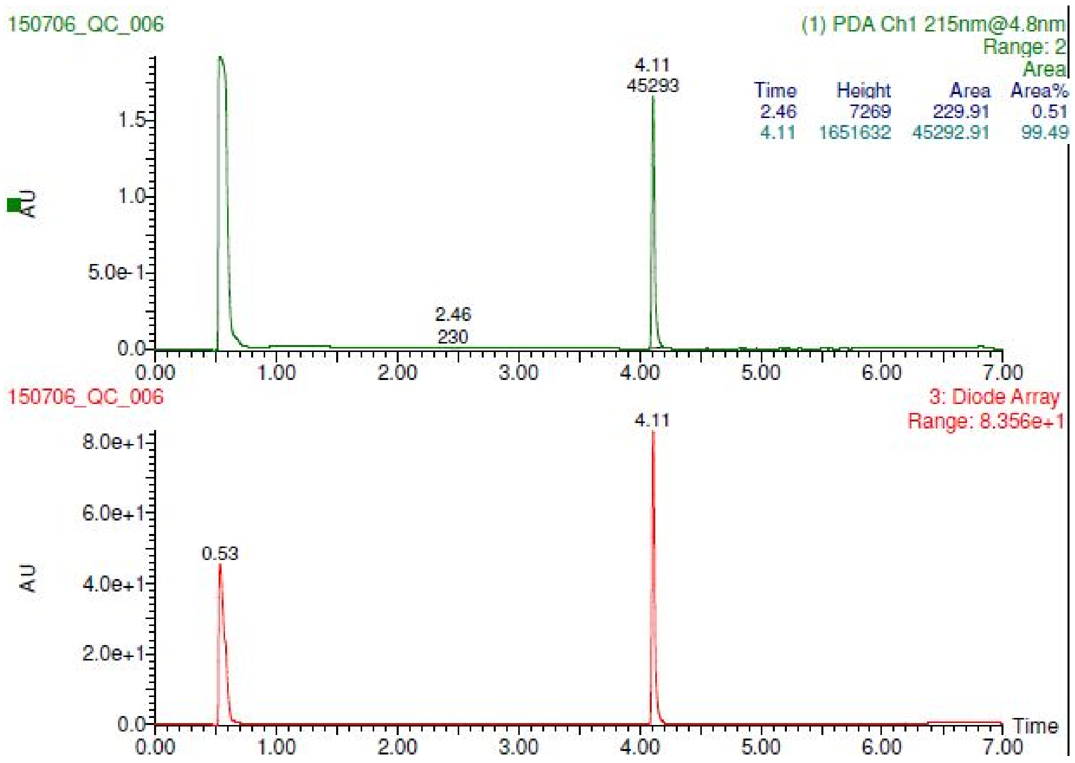

Compound **20**

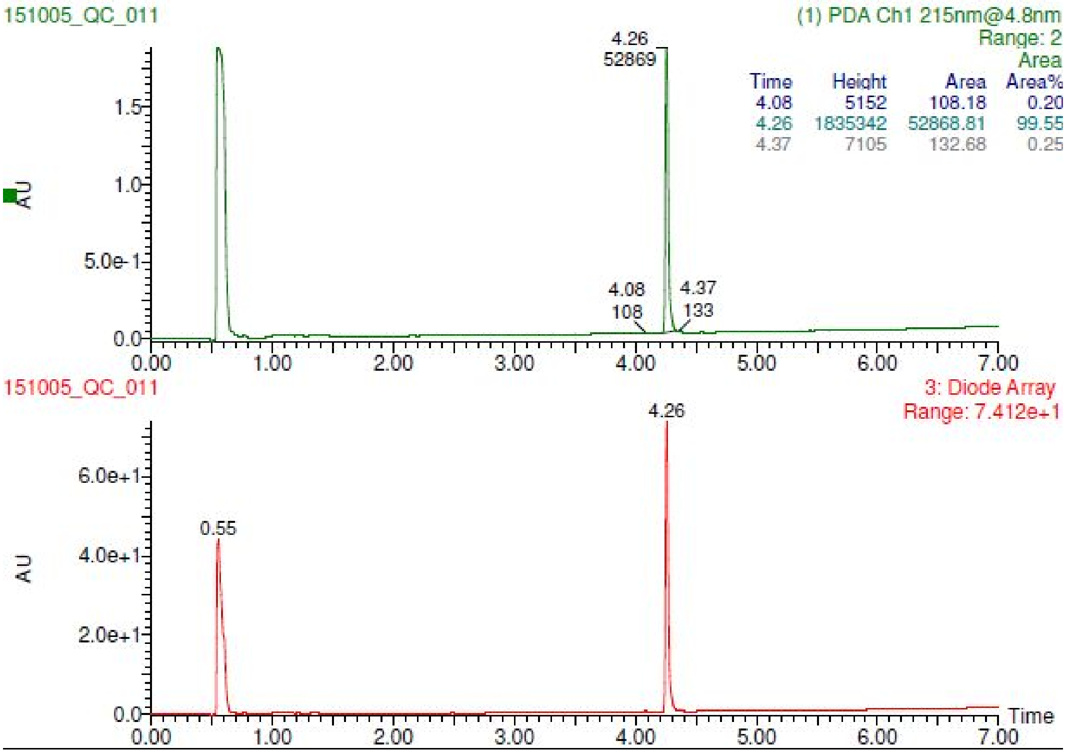

Compound **21**

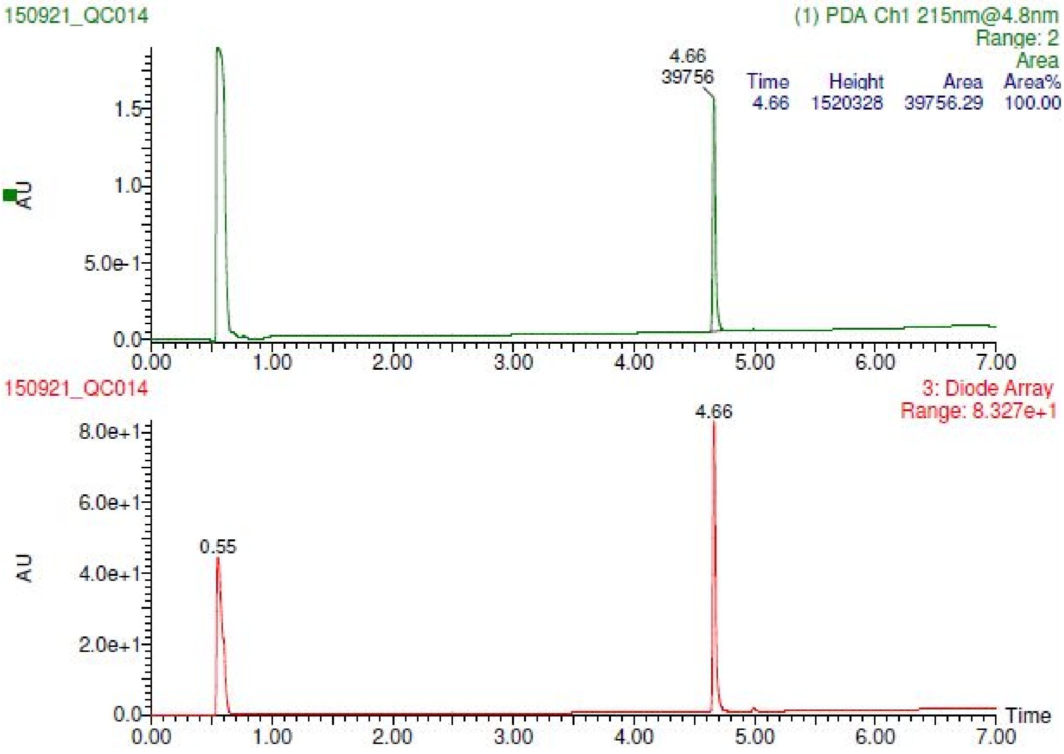

Compound **22**

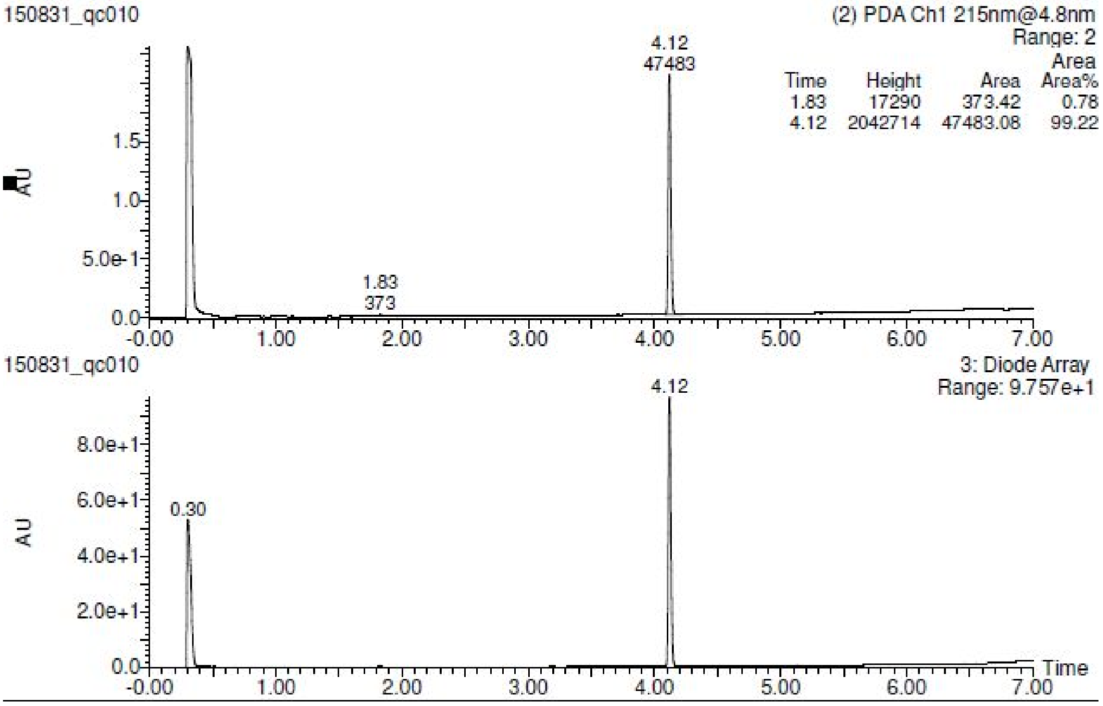

Compound **23**

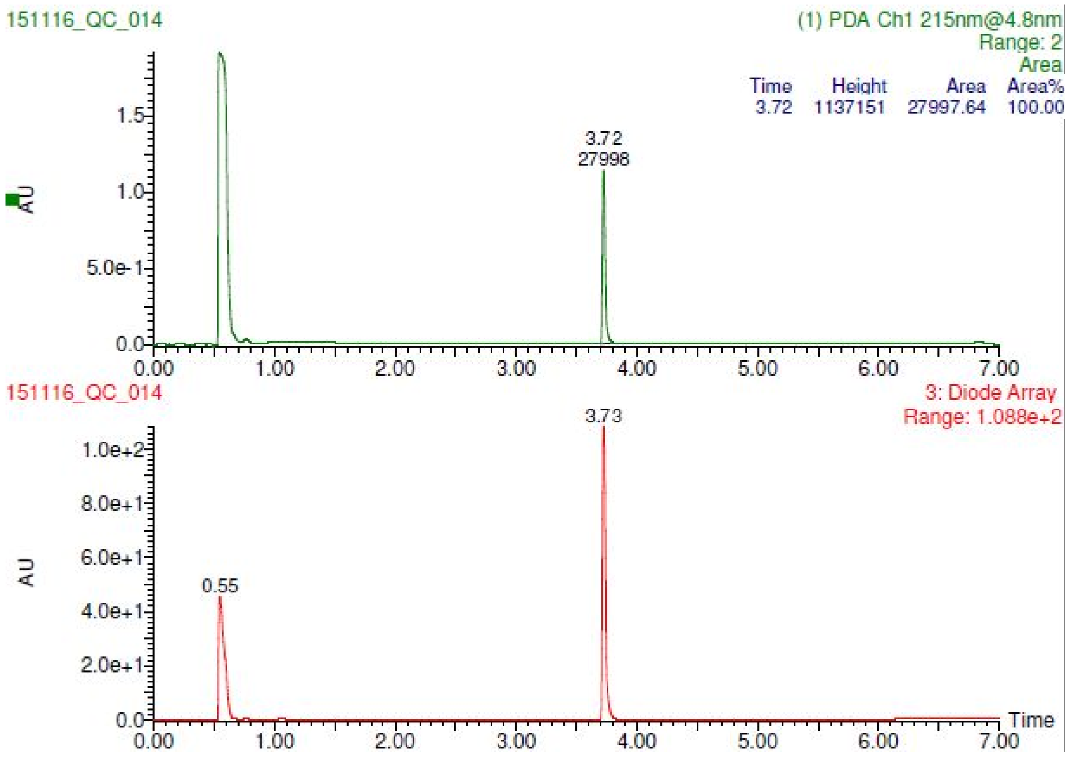

Compound **24**

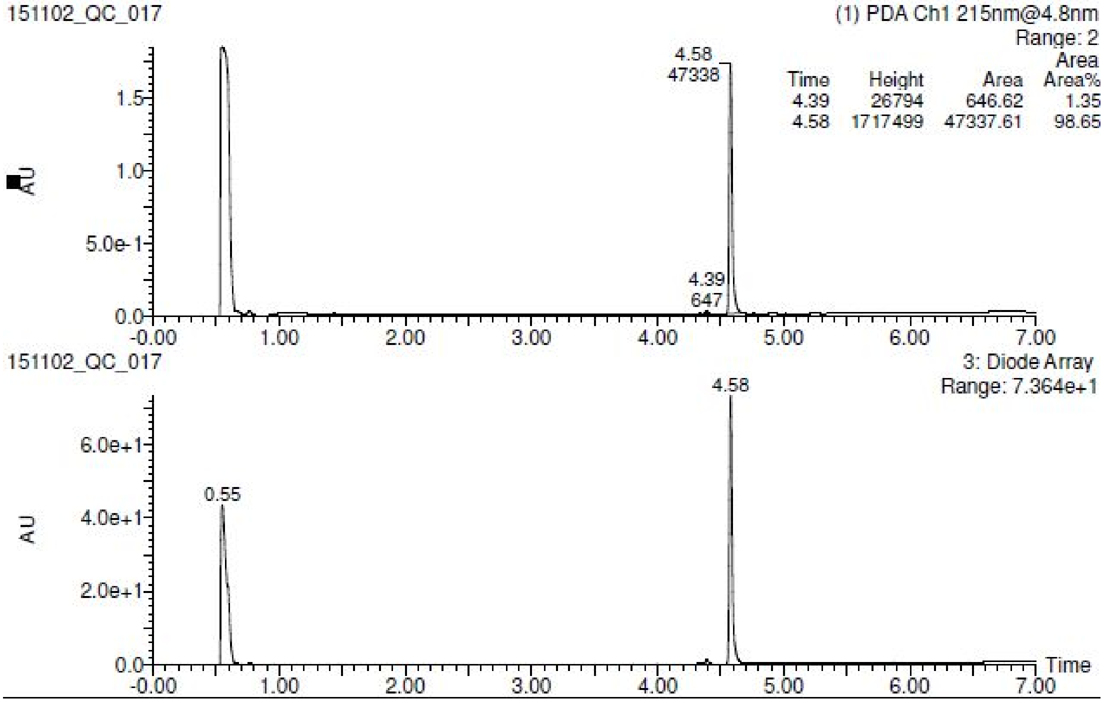

Compound **25**

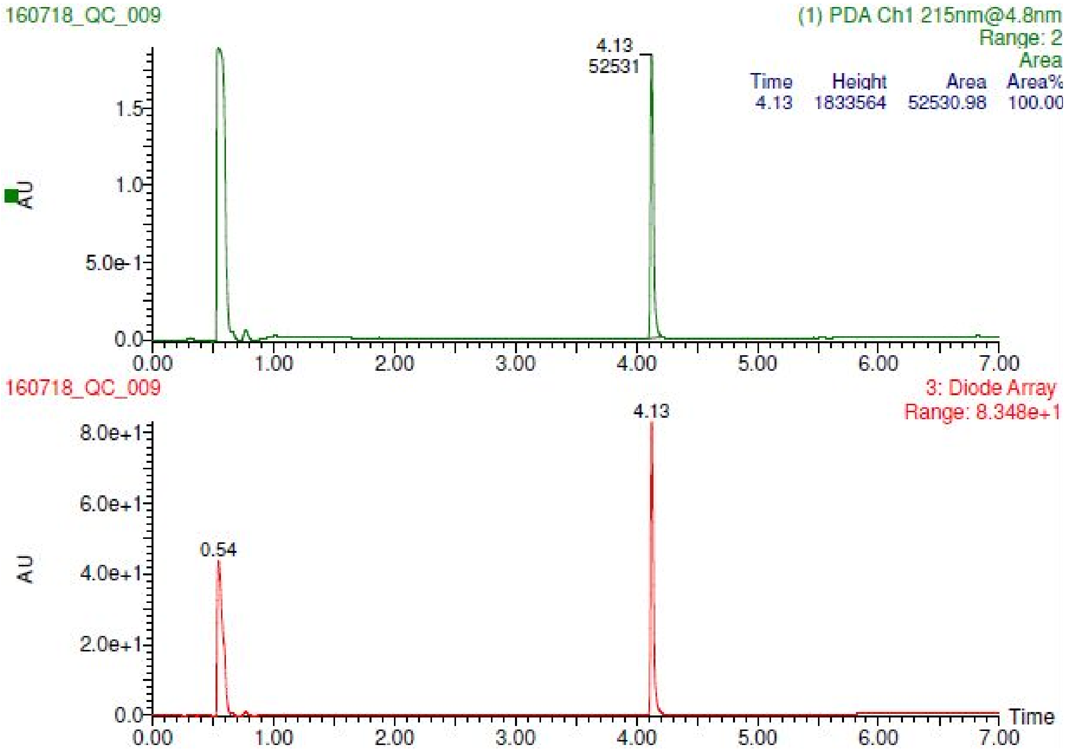

Compound **26**

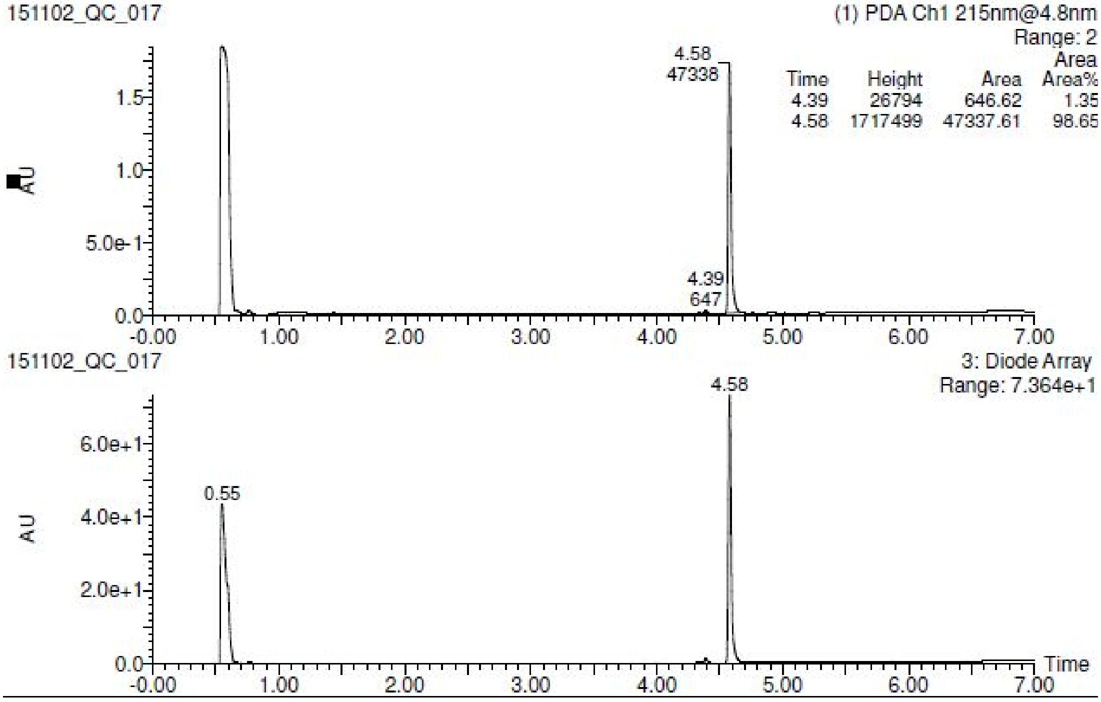

Compound **27**

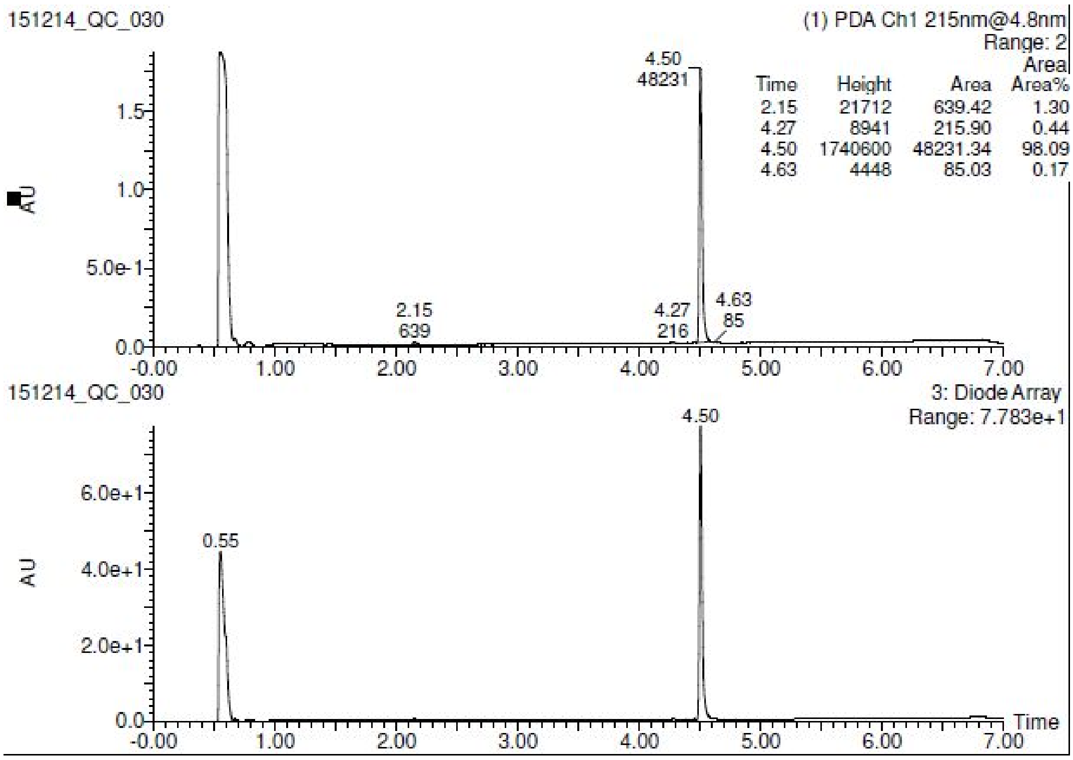

Compound **28**

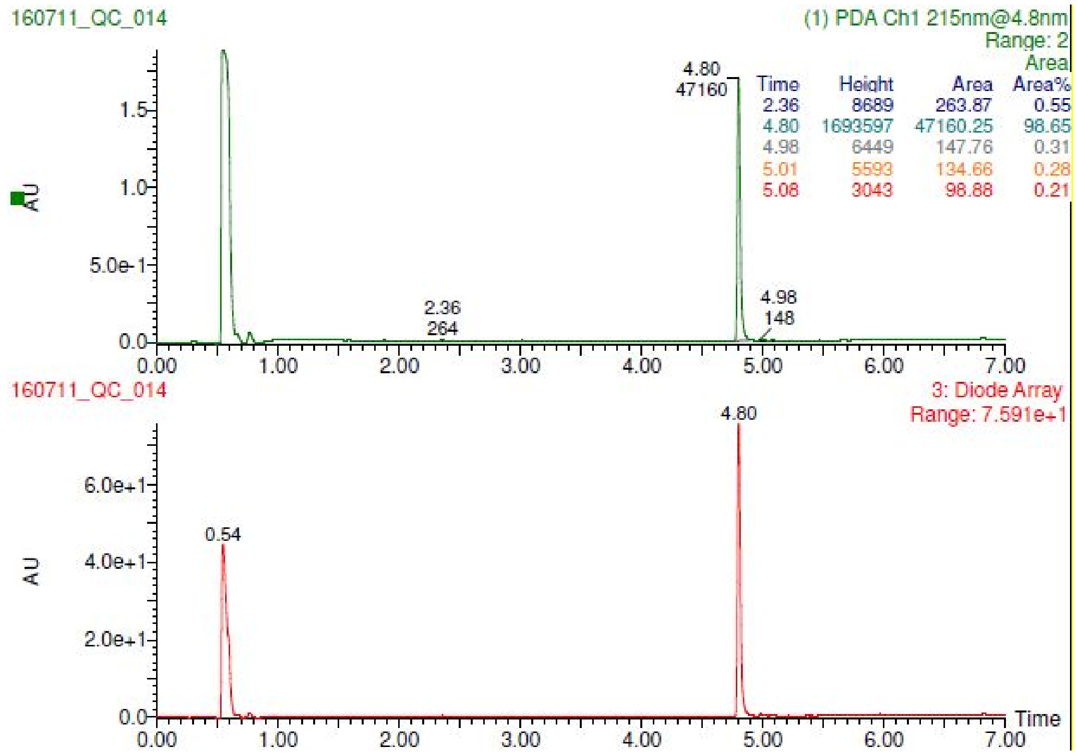

Compound **29**

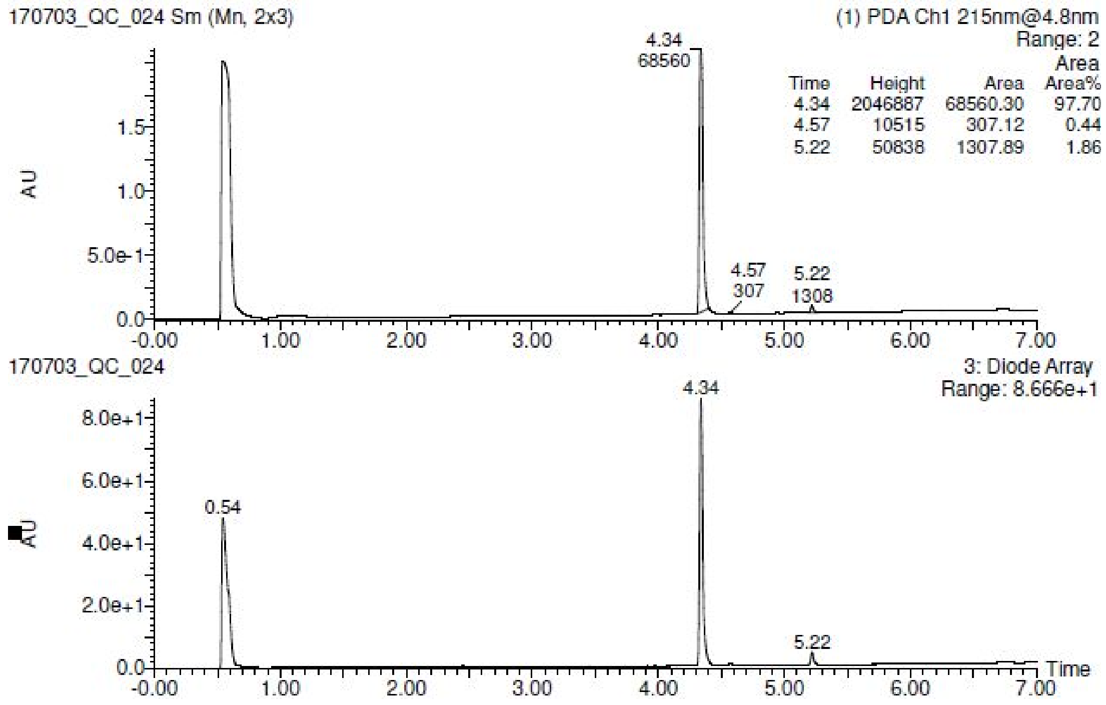

Compound **30**

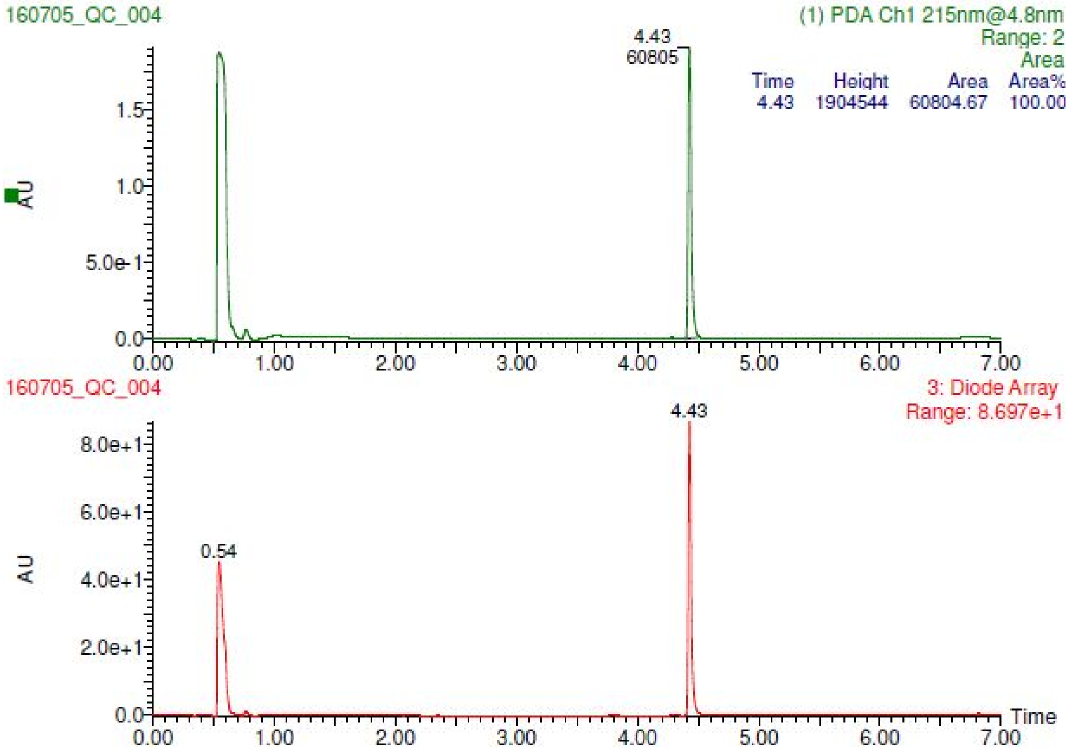

Compound **31**

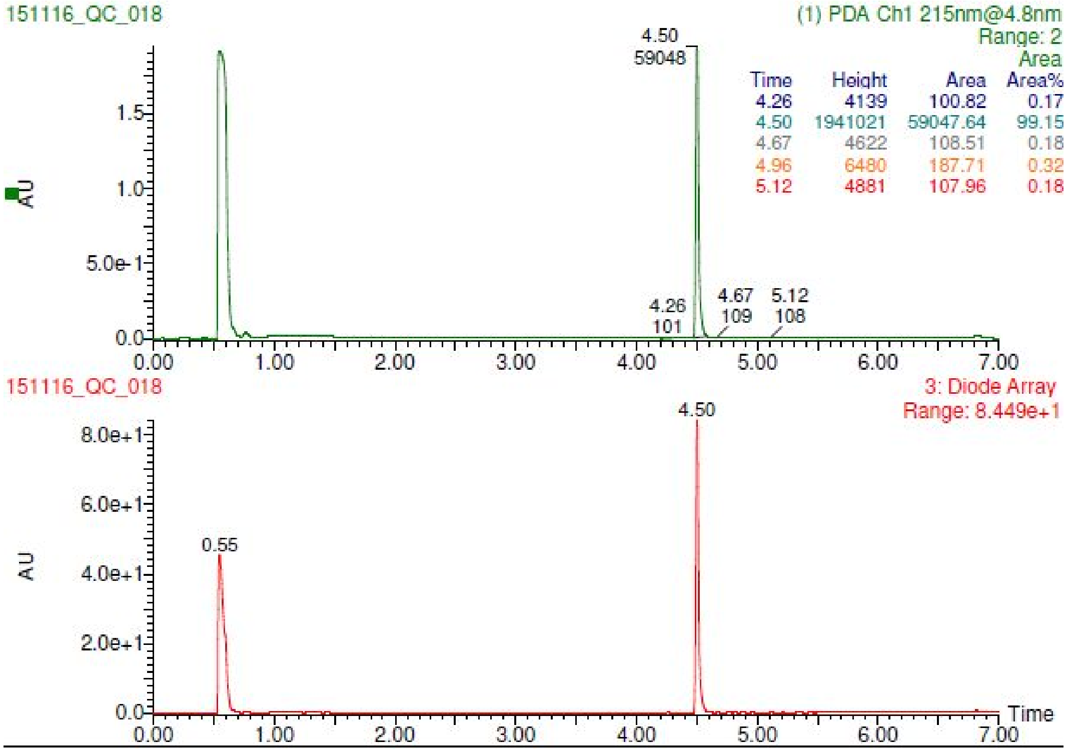

Compound **32**

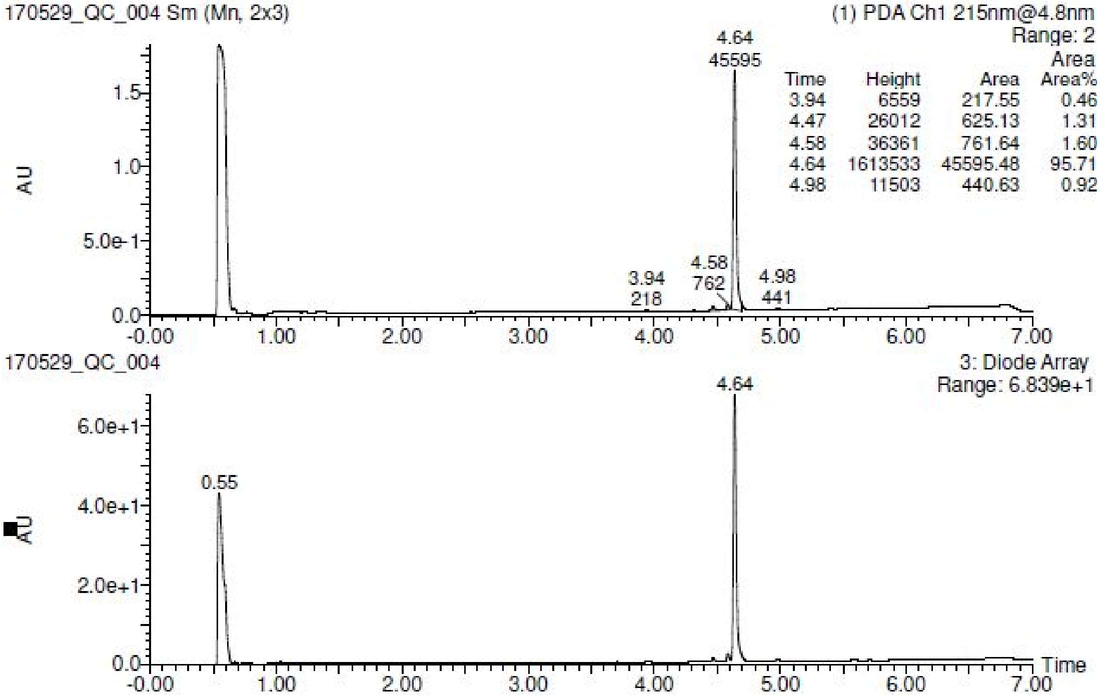

Compound **33**

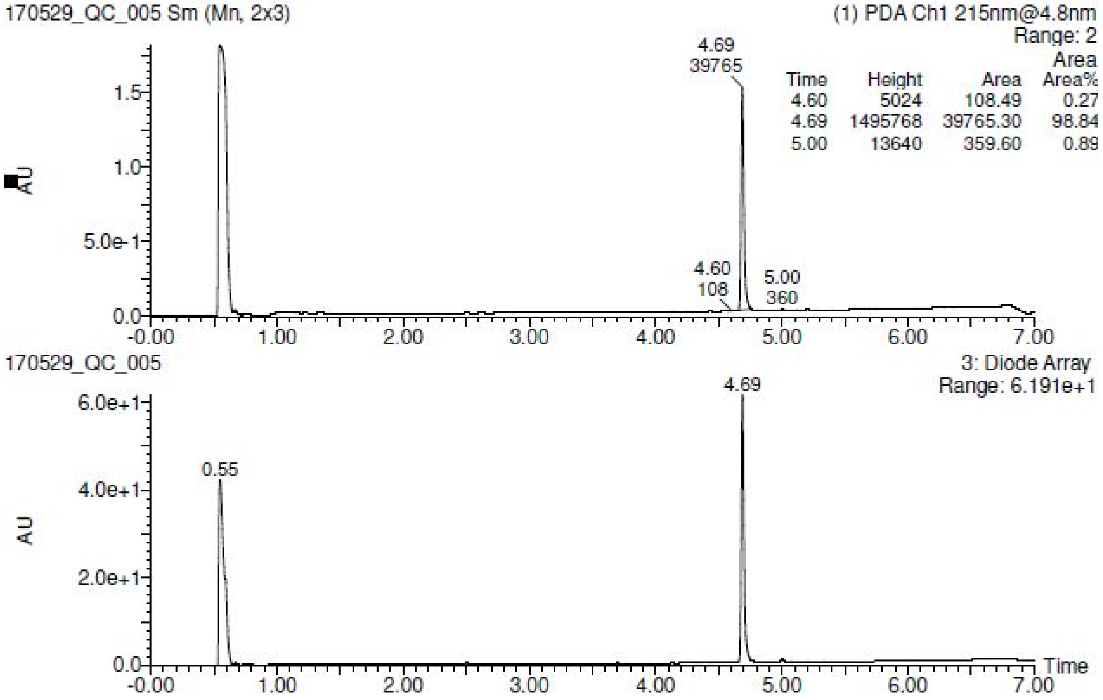

Compound **34**

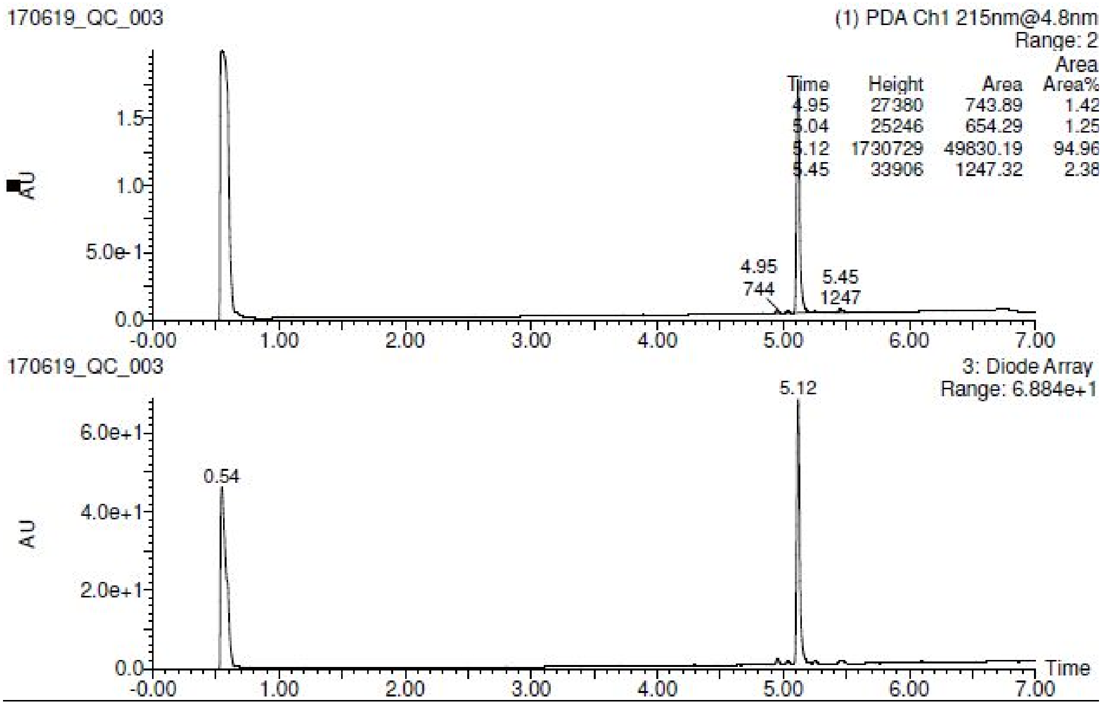

Compound **35**

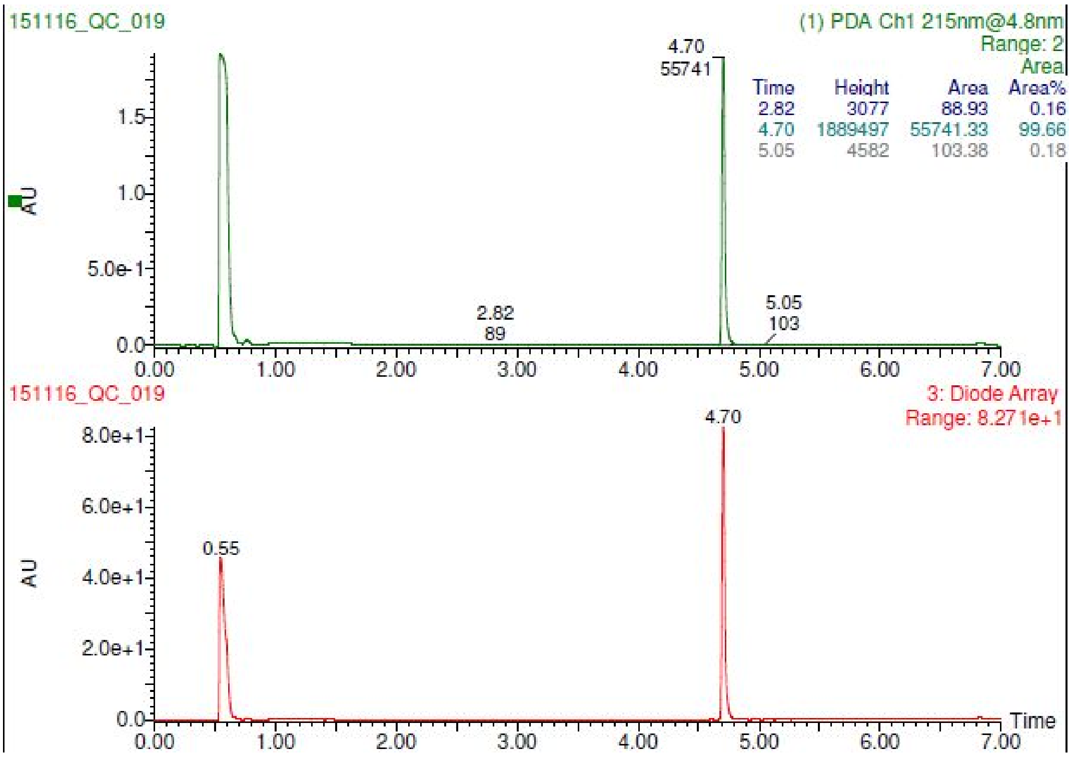

Compound **36**

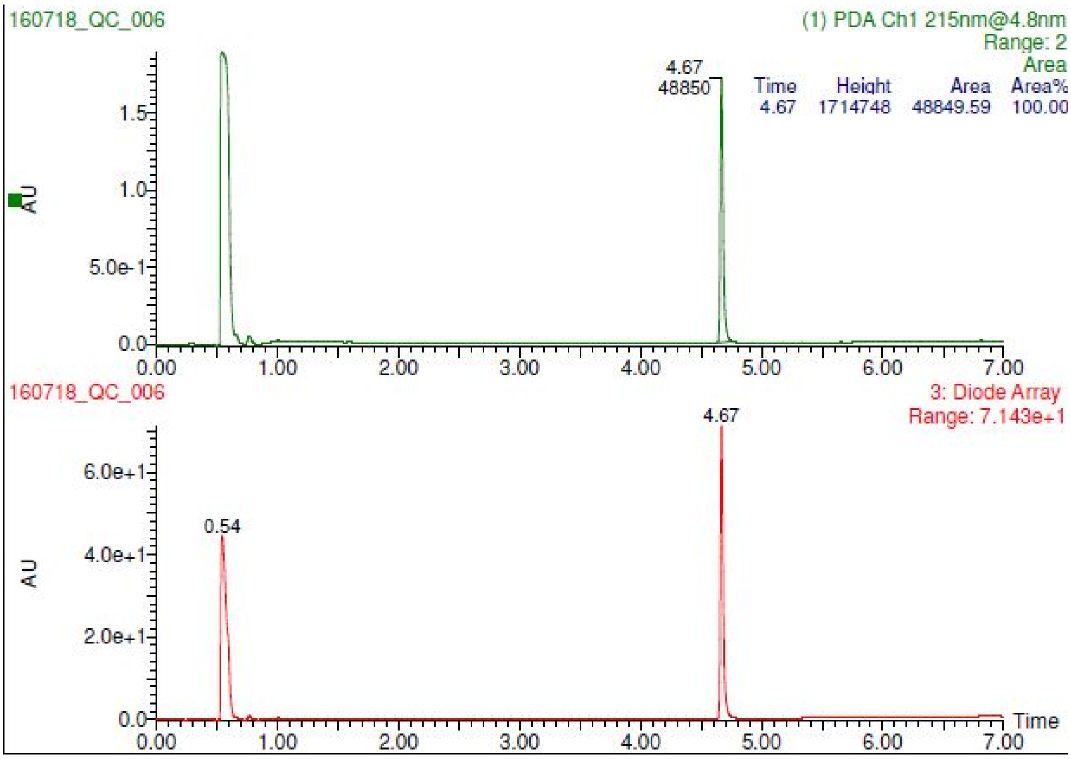

Compound **37**

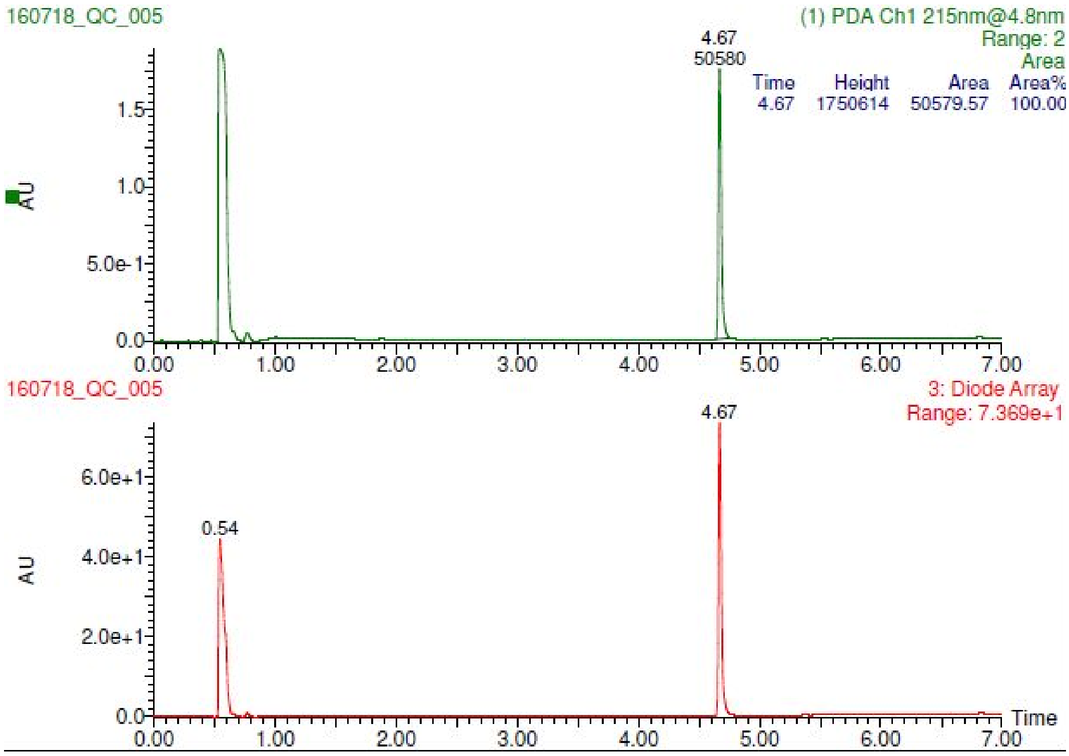

Compound **38**

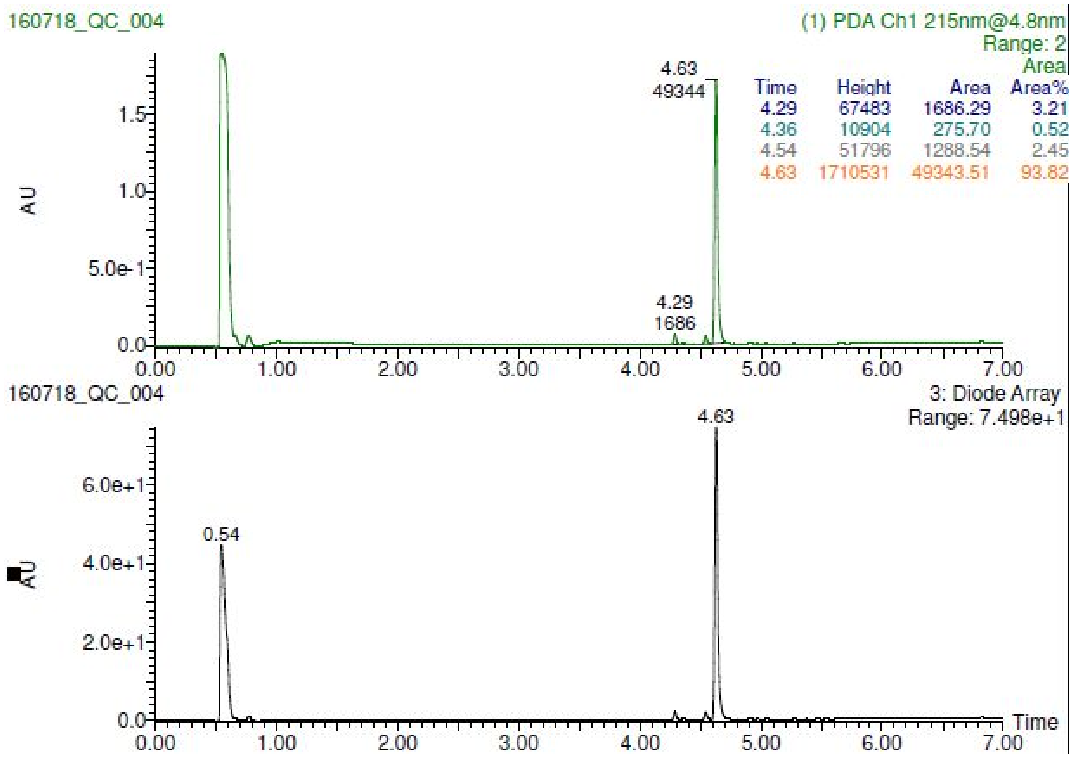

Compound **39**

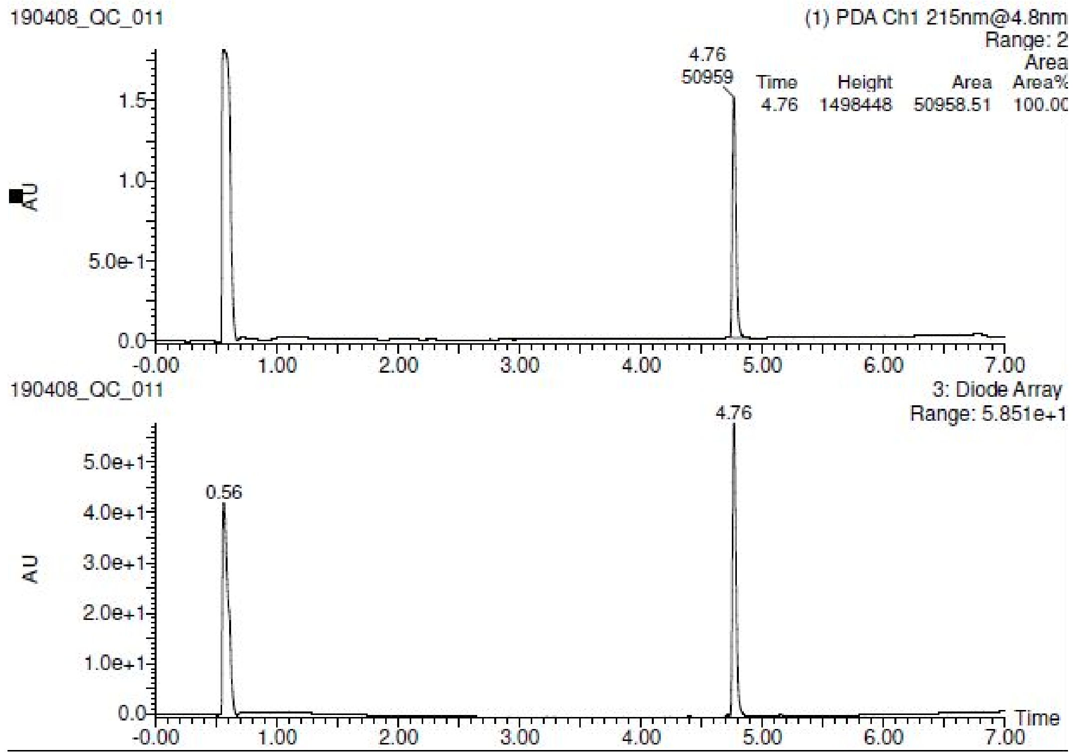

Compound **40**

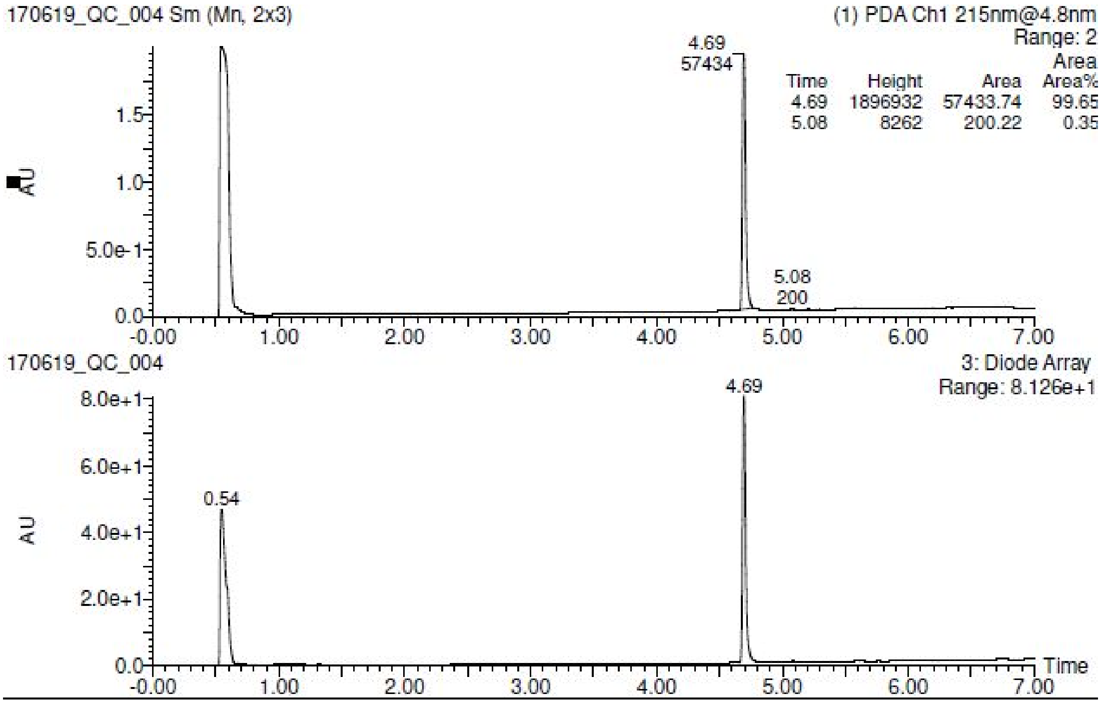

Compound **41**

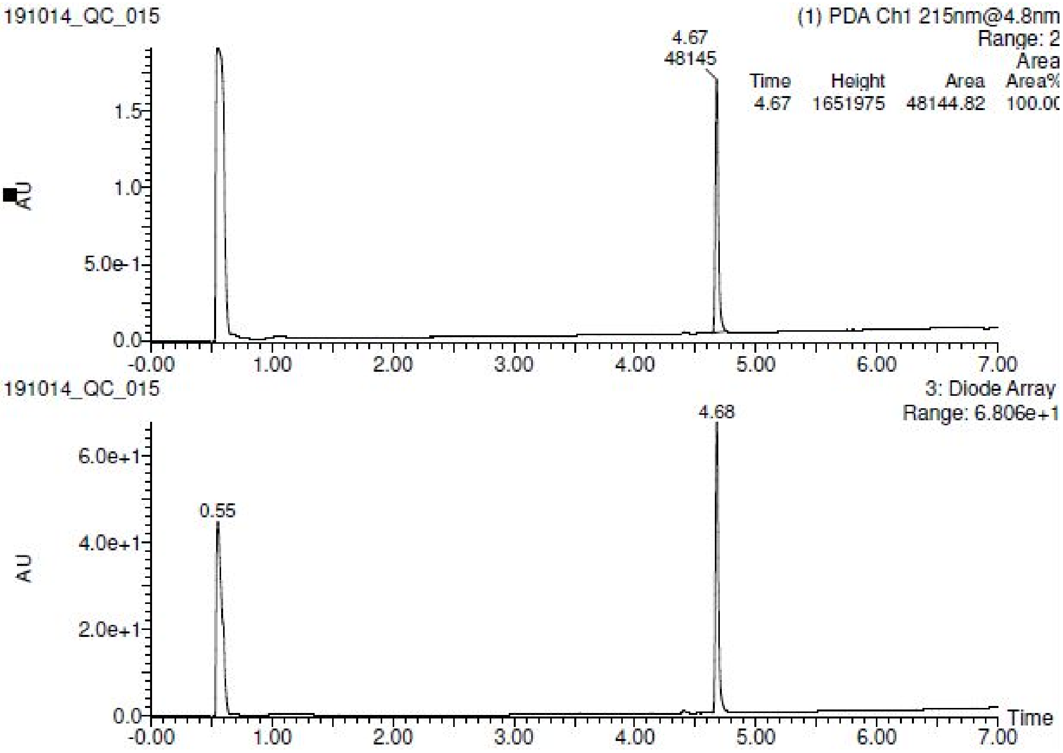

Compound **42**

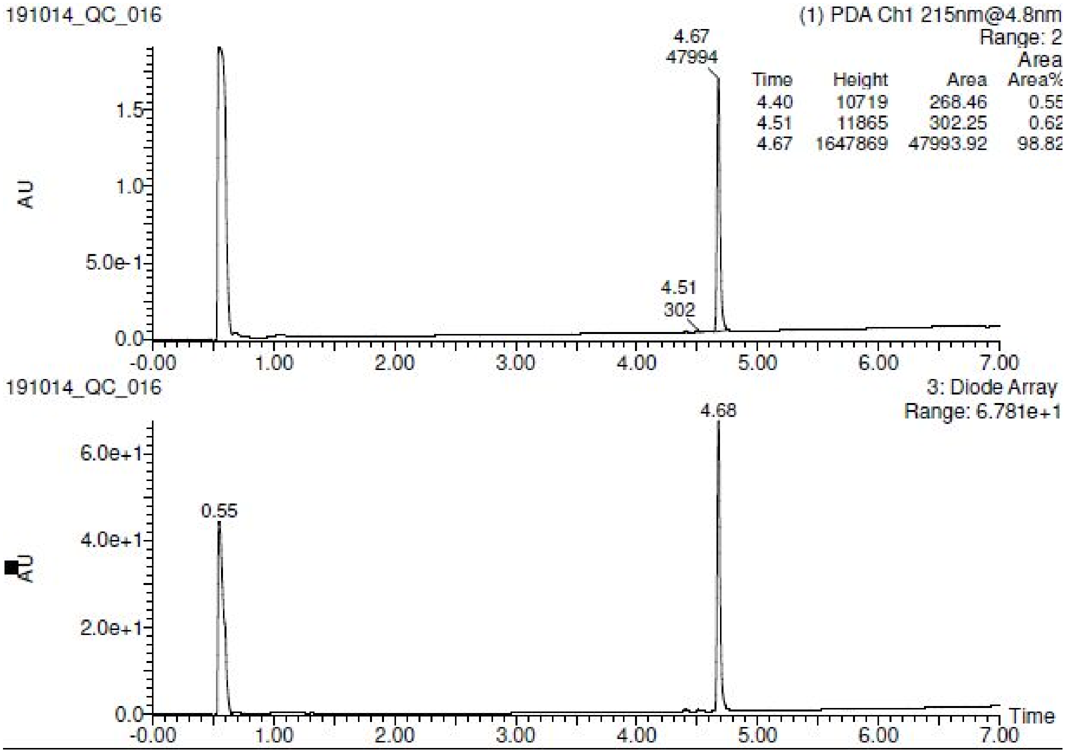

191014_QC_016
(1) PDA Ch1 215nm@4.8nm
Range: 2
4.67
47994
Time Height Area Area%
4.40 10719 268.46 0.55
4.51 11865 302.25 0.62
4.67 1647869 47993.92 98.82
4.51
302
AU
1.5
1.0
5.0e-1
0.0
-0.00 1.00 2.00 3.00 4.00 5.00 6.00 7.00
191014_QC_016
3: Diode Array
Range: 6.781e+1
4.68
0.55
6.0e+1
4.0e+1
2.0e+1
0.0
AU
Time
-0.00 1.00 2.00 3.00 4.00 5.00 6.00 7.00

3. Chiral HPLC analyses of final compounds 32, 33, 35-37, 39-42

The analytical chiral separations were performed on a Waters Alliance HPLC instrument consisting of an e2695 Separation Module and a 2998 Photodiode Array Detector. The PDA range was 210-400nm. The analyses were run in isocratic mode on Daicel ChiralPak AD column (250x4.6mmID, particle size 10µm) with a flow rate =1.0 mL/min.

Chiral HPLC analyses of final tetrahydro-γ-carboline ***32, 41*** *and* ***42***

The separation was run on a Daicel ChiralPak AD column (250x4.6mmID, particle size 10µm) at room temperature, using Heptane/2-Propanol (95:5) as mobile phase.

Data for racemate **32**: *R*t_E1: 118.94 min; *R*t_E2: 156.42 min

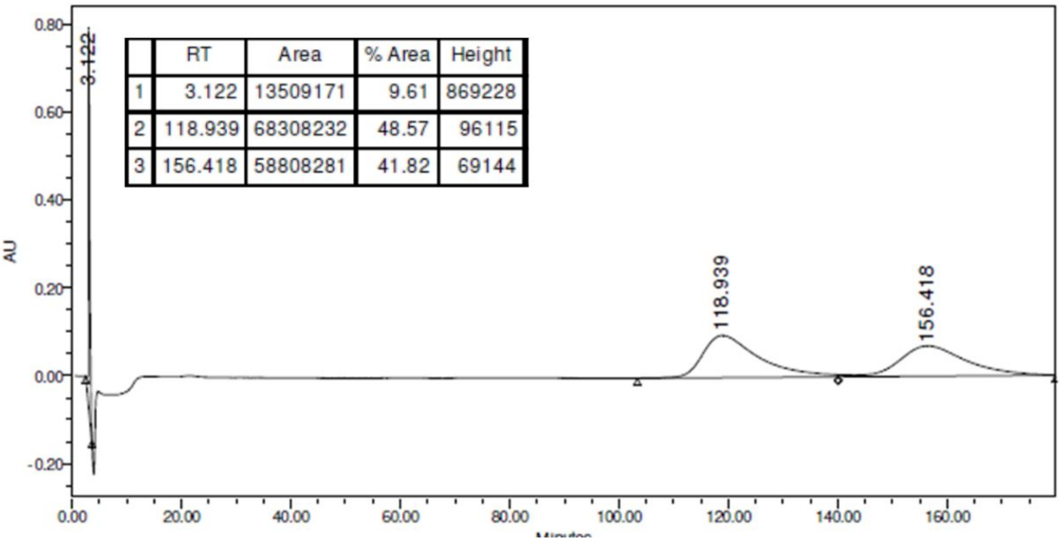

Data for enantioenriched compound **41**: *R*t_E1: 111.08 min (E1_major); *R*t_E2: 137.79 min (E2_minor); (97% ee, least value due to peak-tailing).

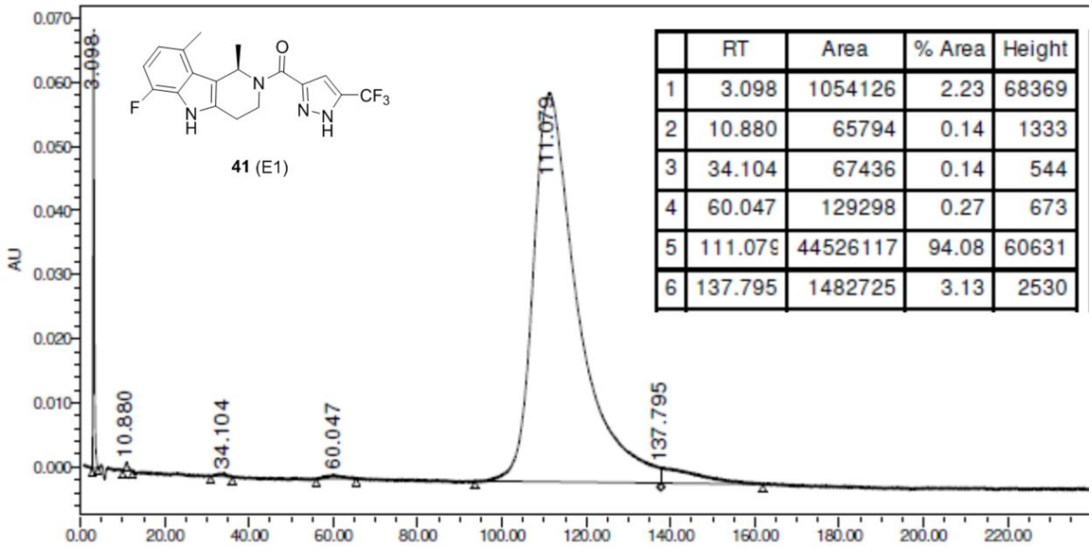

	RT	Area	% Area	Height
1	3.098	1054126	2.23	68369
2	10.880	65794	0.14	1333
3	34.104	67436	0.14	544
4	60.047	129298	0.27	673
5	111.079	44526117	94.08	60631
6	137.795	1482725	3.13	2530

Data for for enantioenriched compound **42**: *R*t_E1: 115.76 min (E1_minor); *R*t_E2: 151.53 min (E2_minor); (63% ee, least value due to peak-tailing)

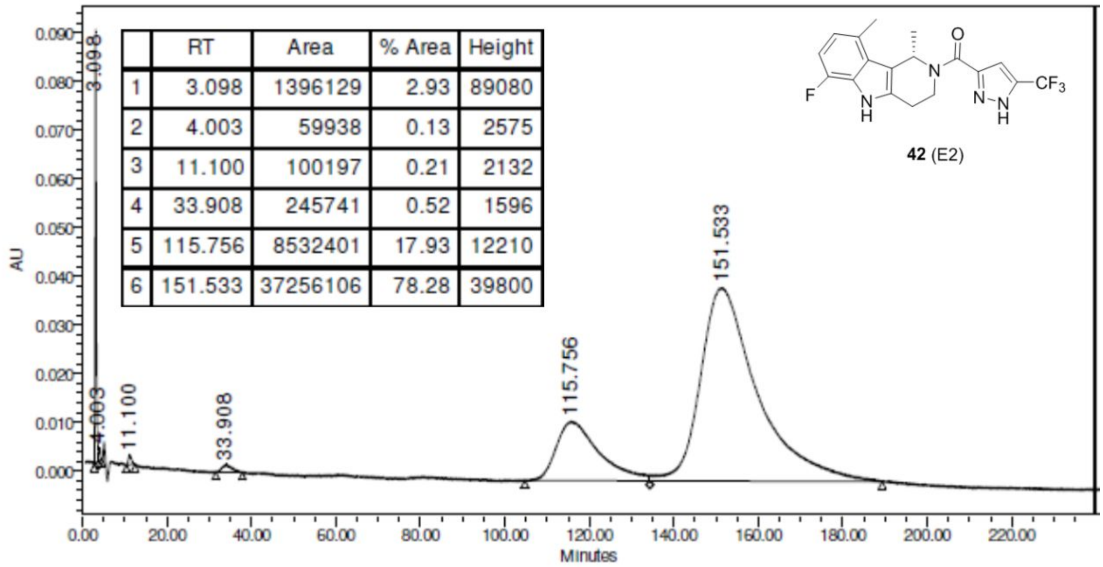

	RT	Area	% Area	Height
1	3.098	1396129	2.93	89080
2	4.003	59938	0.13	2575
3	11.100	100197	0.21	2132
4	33.908	245741	0.52	1596
5	115.756	8532401	17.93	12210
6	151.533	37256106	78.28	39800

*Chiral HPLC analyses of final tetrahydro-γ-carboline **33**, **39** and **40***

The separation was run on a Daicel ChiralPak AD column (250x4.6mmID, particle size 10μm) at room temperature, using Heptane/2-Propanol (95:5) + 0.1% TFA as mobile phase.

Data for racemate **33**: *R*t_E1: 104.74 min; *R*t_E2: 122.24 min

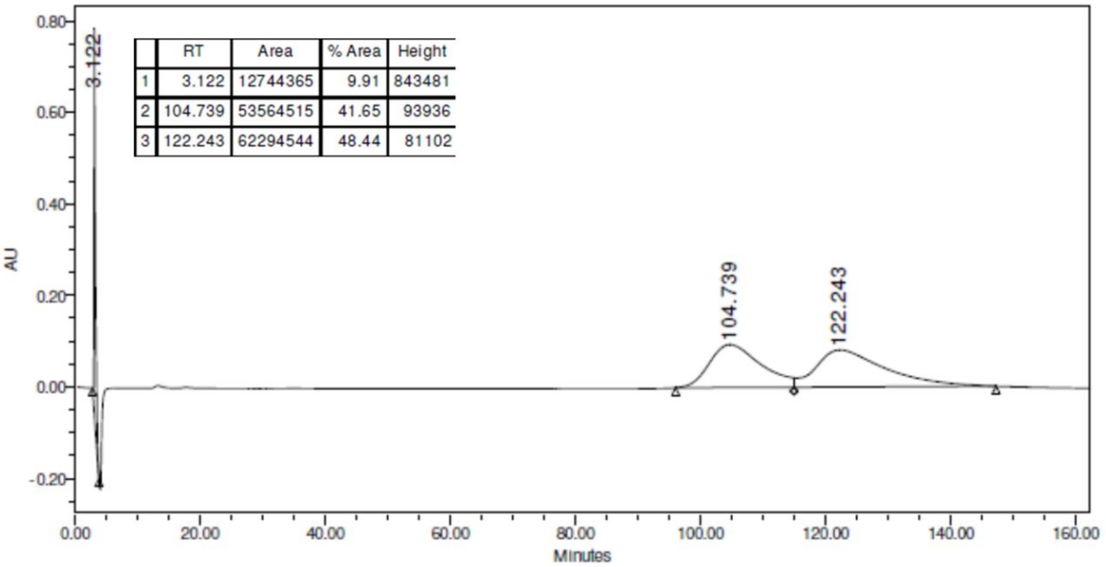

Data for enantioenriched compound **39**: *R*t_E1: 99.38 min (E1_minor); *R*t_E2: 112.75 min (E2_major); (>98% ee)

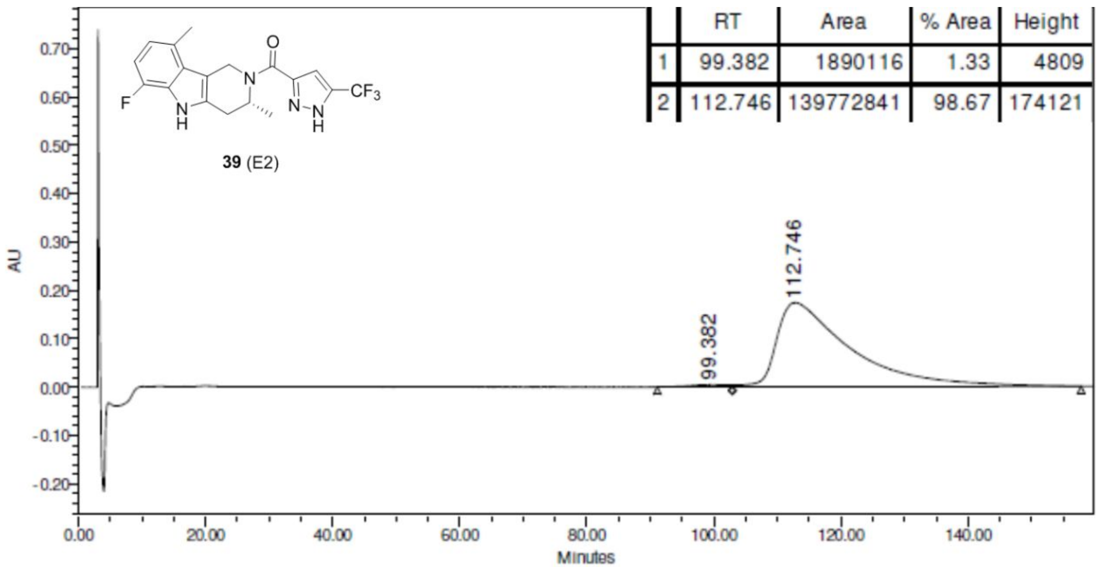

Data for enantioenriched compound **40**: *R*t_E1: 104.40 min (E1_major); *R*t_E2: 119.50 min (E2_minor); (78% ee, least value due to peak-tailing)

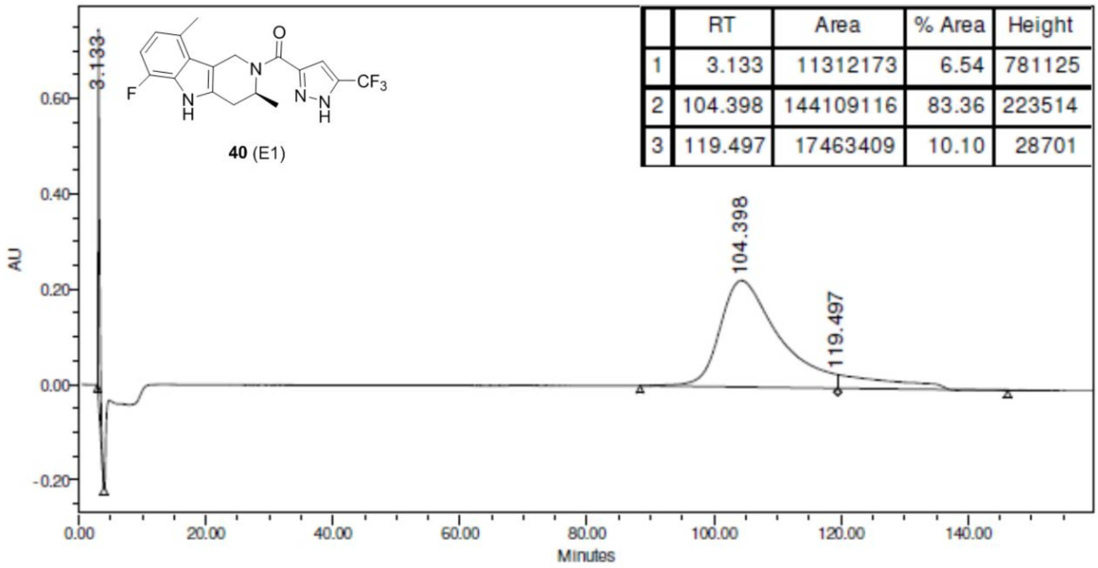

	RT	Area	% Area	Height
1	3.133	11312173	6.54	781125
2	104.398	144109116	83.36	223514
3	119.497	17463409	10.10	28701

*Chiral HPLC analyses of final tetrahydro-γ-carboline **35**, **36** and **37***

The separation was run on a Daicel ChiralPak AD column (250x4.6mmID, particle size 10µm) at room temperature, using Heptane/EtOH (75:25) as mobile phase.

Data for racemate **35**: *R*t_E1: 7.03 min; *R*t_E2: 17.85 min

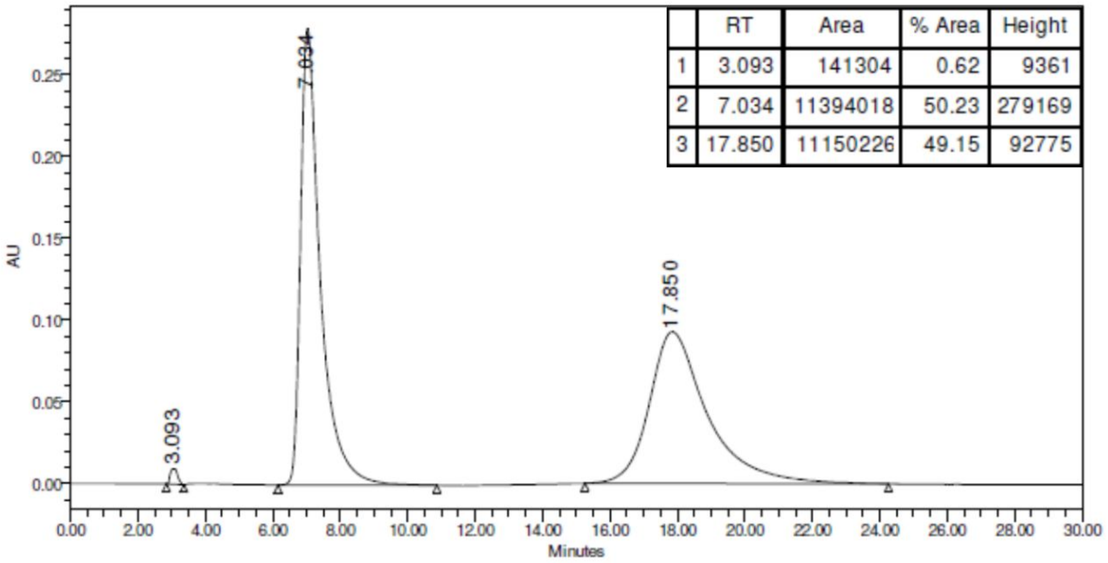

	RT	Area	% Area	Height
1	3.093	141304	0.62	9361
2	7.034	11394018	50.23	279169
3	17.850	11150226	49.15	92775

Data for enantioenriched compound **36**: *R*t_E1: 7.03 min (E1_major); (> 99% ee)

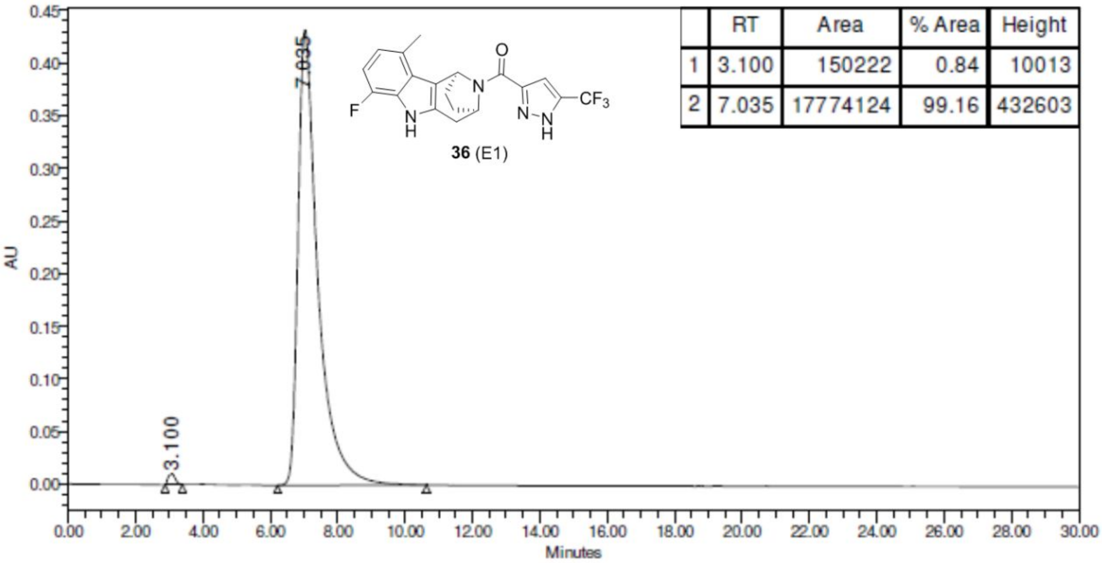

	RT	Area	% Area	Height
1	3.100	150222	0.84	10013
2	7.035	17774124	99.16	432603

Data for enantioenriched compound **37**: *R*t_E2: 17.81 min (E2_major); (> 99% ee)

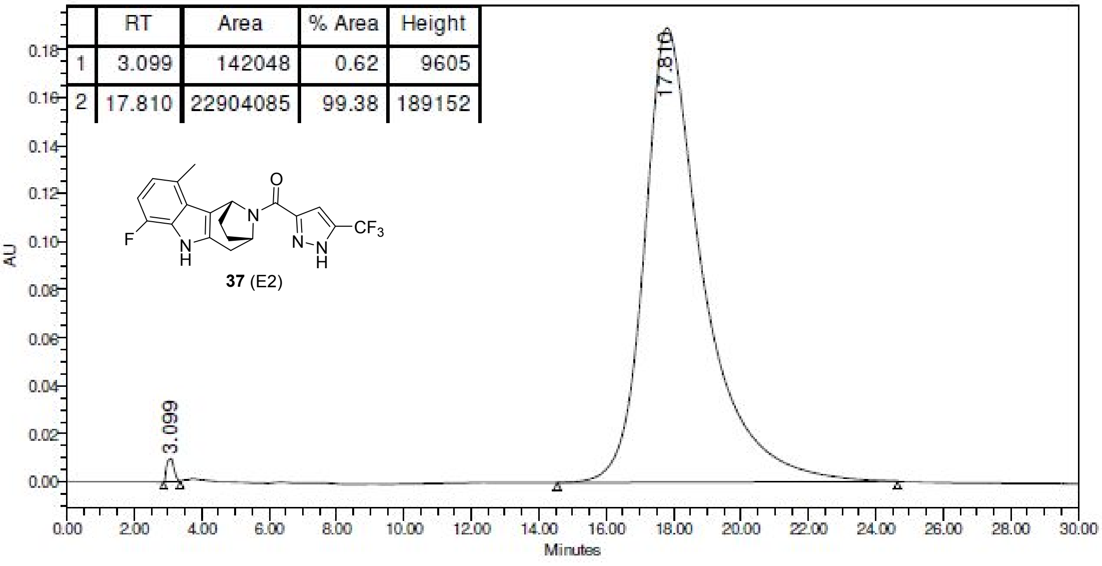

	RT	Area	% Area	Height
1	3.099	142048	0.62	9605
2	17.810	22904085	99.38	189152

4. *In vivo* characterization of compound 39

Pharmacokinetics Methods. Compound **39** was administered intravenously (i.v.) and orally (p.o.) to cannulated male Sprague-Dawley rats at 3 and 10 mg/kg dose (n = 3), respectively. Vehicle was PEG400/Tween 80/saline solution at 10/10/80 % in volume, respectively. Three animals per dose were treated. Blood samples at 0, 15, 30, 60, 90, 120, 240, and 360 min after administration were collected for p.o. arm. Blood samples at 0, 5, 15, 30, 60, 90, 120, and 240 min after administration were collected for i.v. arm. Plasma was separated from blood by centrifugation for 15 min at 3500 rpm a 4 °C, collected in an Eppendorf tube, and frozen (-80 °C). Control animals treated with vehicle only were also included in the experimental protocol.

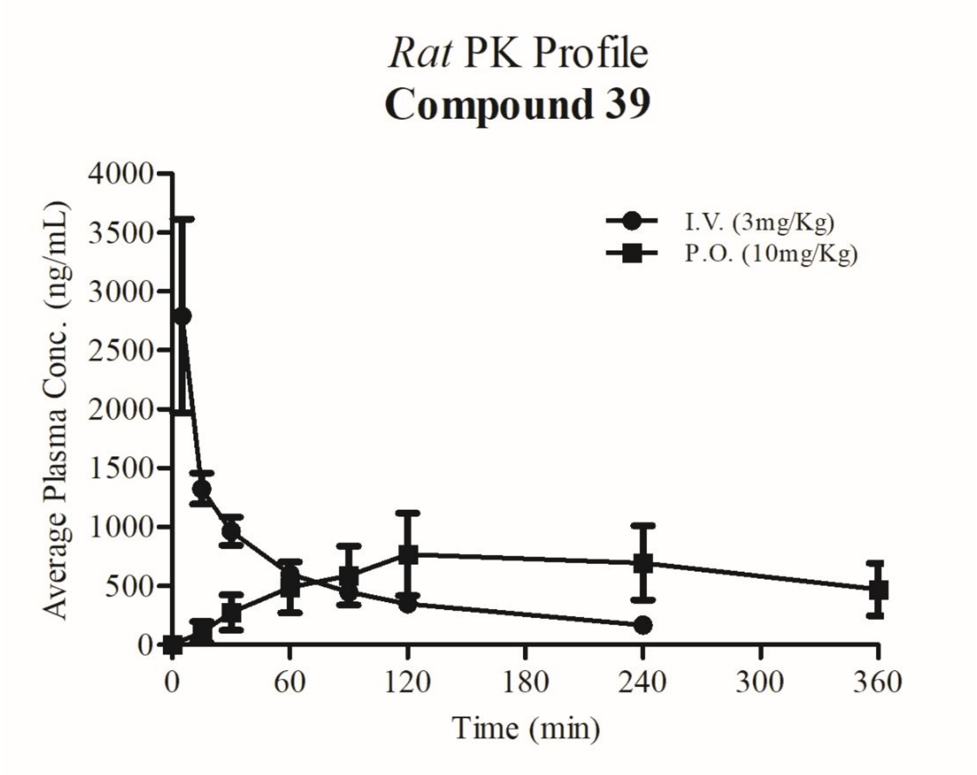

Figure S1. Pharmacokinetic profile of compound **39** after p.o. and i.v. administration in male Sprague Dawley rats (n = 3 per dose).

Sample Preparation for Lung Exposure Analysis. Three animals per dose and timing were treated. Compound **39** was dissolved in PEG400/Tween80/saline solution at 10/10/80 % in volume, respectively, and administered orally at the dose of 10 mg/kg. After 120 and 240 minutes, rats were sacrificed and lungs were immediately dissected, frozen on dry ice, and stored at -80 °C until analysis.

Lung samples were homogenized in RIPA buffer (150 mM NaCl, 1.0% Triton X-100, 0.5% sodium deoxycholate, 0.1% sodium dodecyl sulfate, 50 mM Tris, pH 8.0) and were then split into two aliquots kept at -80 °C until analysis. An aliquot was used for compound lung level evaluations. The second aliquot was kept for protein content evaluation using the bicinchoninic acid (BCA) assay (Thermo Scientific, Rockford, IL, USA).

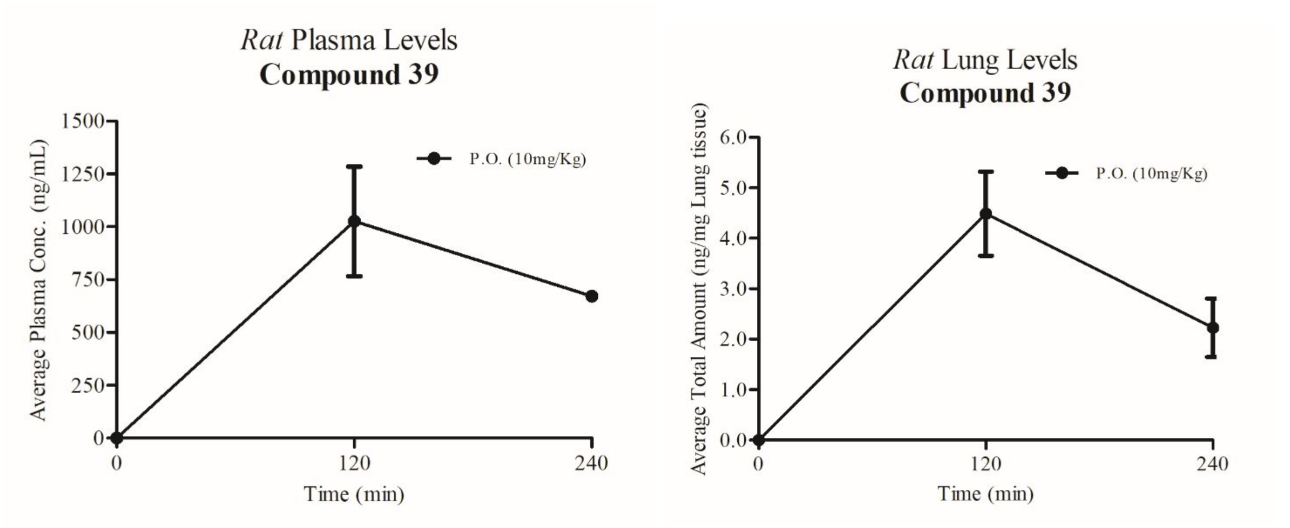

Figure S2. The mean concentration vs. time profiles of compound **39** in plasma and lung tissue after p.o. administration in male Sprague Dawley rats (n = 3).

5. References

1. Wu, P. S.; Otting, G. Rapid Pulse Length Determination in High-Resolution NMR. *J. Magn. Reson.* **2005,** *176*, 115-119.
2. Wider, G.; Dreier, L. Measuring Protein Concentrations by NMR Spectroscopy. *J. Am. Chem. Soc.* **2006,** *128*, 2571-2576.
